# Characterization of the Deleted in Autism 1 Protein Family: Implications for Studying Cognitive Disorders

**DOI:** 10.1371/journal.pone.0014547

**Published:** 2011-01-19

**Authors:** Azhari Aziz, Sean P. Harrop, Naomi E. Bishop

**Affiliations:** Department of Microbiology, La Trobe University, Bundoora, Australia; Deutsches Krebsforschungszentrum, Germany

## Abstract

Autism spectrum disorders (ASDs) are a group of commonly occurring, highly-heritable developmental disabilities. Human genes *c3orf58* or *Deleted In Autism-1* (*DIA1*) and *cXorf36* or *Deleted in Autism-1 Related* (*DIA1R*) are implicated in ASD and mental retardation. Both gene products encode signal peptides for targeting to the secretory pathway. As evolutionary medicine has emerged as a key tool for understanding increasing numbers of human diseases, we have used an evolutionary approach to study *DIA1* and *DIA1R*. We found *DIA1* conserved from cnidarians to humans, indicating *DIA1* evolution coincided with the development of the first primitive synapses. Nematodes lack a *DIA1* homologue, indicating *Caenorhabditis elegans* is not suitable for studying all aspects of ASD etiology, while zebrafish encode two *DIA1* paralogues. By contrast to *DIA1*, *DIA1R* was found exclusively in vertebrates, with an origin coinciding with the whole-genome duplication events occurring early in the vertebrate lineage, and the evolution of the more complex vertebrate nervous system. Strikingly, *DIA1R* was present in schooling fish but absent in fish that have adopted a more solitary lifestyle. An additional *DIA1*-related gene we named *DIA1-Like* (*DIA1L*), lacks a signal peptide and is restricted to the genomes of the echinoderm *Strongylocentrotus purpuratus* and cephalochordate *Branchiostoma floridae*. Evidence for remarkable *DIA1L* gene expansion was found in *B. floridae*. Amino acid alignments of *DIA1* family gene products revealed a potential Golgi-retention motif and a number of conserved motifs with unknown function. Furthermore, a glycine and three cysteine residues were absolutely conserved in all *DIA1*-family proteins, indicating a critical role in protein structure and/or function. We have therefore identified a new metazoan protein family, the DIA1-family, and understanding the biological roles of DIA1-family members will have implications for our understanding of autism and mental retardation.

## Introduction

Autism spectrum disorder (ASD) is a neurodevelopmental condition commonly diagnosed in early childhood. ASD is characterized by deficits in verbal and non-verbal communication, social interaction, and by displays of restricted and/or repetitive behaviours (Mendelian Inheritance in Man accession number 209850). In the absence of definitive neuropathological markers, these deficits remain the sole diagnostic indicators of autism. ASD has the greatest heritable basis of any developmental cognitive disorder, based on twin and family studies, with heritability estimates of around 90% [1]–[9]. In addition, spontaneous genetic alterations cause around 10% of cases [10]–[12]. As expected, many of the genes implicated in ASD have direct or indirect roles in synapse formation and function [13]–[19]. The secretory pathway plays a key role in neuron function, and abnormalities in secretion and secretory cargo have been found in increasing numbers of ASD patients [20]–[23]. Genes affecting secretory pathway traffic are also reported to be affected in those with ASD [12], [24]. Furthermore, post-translational modification of proteins in the secretory pathway via phosphorylation and sulphation are abnormal in ASD patients, as are genes involved in glycosylation events within the Golgi apparatus lumen [12], [25]–[28].

The wide phenotypic presentation of ASD is reflected in the increasing number of different gene changes identified in those affected [29]. Many genes implicated in ASD are of known function, while for other genes a role in brain function is yet to be identified. Many of the characterized and uncharacterized genes have orthologues in model organisms, which can be used to study biological function. The mouse model is the most widely used to experimentally manipulate candidate genes for ASD susceptibility [30]. Zebrafish, which have highly complex social behaviours, are also emerging as useful models for vertebrate neurodevelopment and autism research [31]–[34]. Increasing use is also being made of smaller organisms to further our understanding of neurobiology and neurological disorders. For example, the fruit fly *Drosophila melanogaster* encodes many homologues of human neuronal genes, including significant numbers implicated in neurological disease [35], [36]. Indeed, the first specific therapeutic treatment for a condition with co-morbid ASD symptoms was reported using a *Drosophila* model of fragile X syndrome [37]. This study [37] demonstrated for the first time that genetic defects such as ASD and mental retardation might be treatable after birth using drugs, rather than more complex gene therapy-based approaches.

Recently, in a study of consanguineous families, an uncharacterized gene was implicated in the etiology of ASD [38]. Deletion of this gene, known as *c3orf58*, was hemizygous in the unaffected parents and an unaffected sibling, but homozygous in a child with ASD. The gene was therefore renamed *DIA1*, for *Deleted In Autism 1* [38]. We have recently identified a second human gene, closely related to *DIA1*, which we have named *DIA1R*, for *DIA1-Related* [39]. *DIA1R* mutations and deletions are associated with X-linked mental retardation and/or ASD-like syndromes [39]. In order to further understand the biological role of *DIA1* and *DIA1R*, we have used an *in silico* approach to study the wider *DIA1* gene family. We report that *DIA1* is restricted to metazoans, while the closely-related homologue, *DIA1R*, is restricted to vertebrates, with the latter being strikingly absent from fish with a solitary lifestyle. An additional *DIA1* family member was found in echinoderm and cephalochordate genomes, which we name *DIA1L* (*DIA1-Like*). By contrast to the *DIA1* and *DIA1R* gene products, which encode signal peptides, *DIA1L* gene products are predicted to be cytosolic. Unexpectedly, *Caenorhabditis elegans* lacks an identifiable *DIA1* homologue, suggesting gene loss in the nematode lineage. Homologues of *DIA1* could not be detected prior to evolution of the Cnidaria, coinciding with the development of the first primitive synapses [40]. These findings provide us with evolutionary support for a role of *DIA1* homologues in neuronal function. We have therefore identified a new gene family, the *DIA1*-family, where there is increasing evidence two members, *DIA1* and *DIA1R*, play a vital role in normal human brain function.

## Results

### Identification of *DIA1* orthologues

The human (*Homo sapiens*) gene *c3orf58*, at chromosome position 3q24, has recently been renamed *DIA1* on the basis of its deletion in ASD [38]. Human *DIA1* has known orthologues in the genome of ten animal species [39], [41]. To characterize the *DIA1* family in detail, we used Basic Local Alignment Search Tool (BLAST) and keyword searches (search term: c3orf58) of publicly-available databases to identify further sequences orthologous to human *DIA1*. In total, thirty five full-length (Tables 1, S1, and S3) and forty five partial (Table S2) *DIA1* orthologues were identified. ‘Partial *DIA1* orthologues’ are those where the full-length gene or cDNA sequence is currently incomplete (partial), due to gaps in genomic sequence or because of the limited read-length of expressed sequence tag (EST) sequencing data. Protein length varied from 345 (*Drosophila willistoni* and *D. yakuba* DIA1) to 477 (*Ciona intestinalis* DIA1) amino acids, equating to between 40 and 54 kDa in predicted molecular mass (Tables 1 and S3). The average length of DIA1 proteins identified was 376 amino acids, with insect gene products, especially those of *Drosophila* species, being shorter than those from most other species (Table 1 and Table S3). Accession numbers of all *DIA1* orthologues are provided in Tables S1 and S2.

**Table 1:** Physical characteristics of DIA1 proteins and similarity to orthologues from key species.

				BLASTP^d^ similarity to^e^:						
Metazoan species^a^	Length protein (amino acids)	pI^b^	Molecular mass^c^ (kDa)	*H. sapiens*	*G. gallus*	*D. rerio (a)*	*C. intestinalis*	*S. purpuratus*	*D. melanogaster*	*N. vectensis*
**Cnidaria**										
*Nematostella vectensis*	402	5.6	44.7	8e-46	4e-44	6e-49	1e-15	6e-47	7e-08	-
**Echinodermata**										
*Strongylocentrotus purpuratus*	431	7.9	49.7	6e-80	3e-81	5e-84	3e-17	-	2e-08	2e-42
**Arthropoda**										
Hexapoda										
*Aedes aegypti*	395	6.0	45.7	6e-16	6e-15	2e-14	1e-04	5e-16	6e-14	5e-15
*Anopheles gambiae*	412	5.4	47.3	2e-06	3e-08	9e-09	0.10	5e-12	4e-10	0.018
*Culex pipiens*	400	5.1	45.2	2e-13	4e-13	8e-16	5e-04	1e-15	2e-13	2e-18
*Drosophila melanogaster*	348	5.3	40.0	1e-06	7e-07	2e-08	>10	2e-08	-	5e-08
*Nasonia vitripennis*	404	5.1	46.2	3e-53	7e-53	2e-54	1e-11	9e-47	2e-08	4e-36
**Chordata**										
Urochordata										
*Ciona intestinalis*	477	9.3	54.5	2e-29	1e-31	3e-34	-	3e-17	>10	4e-14
Cephalochordata										
*Branchiostoma floridae*	398	5.2	46.1	3e-101	5e-98	9e-104	2e-31	5e-81	7e-12	6e-43
Vertebrata										
Neopterygii										
*Danio rerio* (a)	432	8.6	50.0	0.0	0.0	-	6e-32	8e-82	2e-07	1e-46
*Danio rerio* (b)	429	8.7	49.4	0.0	0.0	0.0	6e-31	4e-80	2e-06	3e-44
*Gasterosteus aculeatus*	434	8.9	50.0	0.0	0.0	0.0	6e-33	5e-81	3e-06	2e-44
*Oryzias latipes*	434	8.8	50.1	0.0	0.0	0.0	2e-32	3e-83	3e-06	2e-44
*Takifugu rubripes*	435	8.8	50.1	0.0	0.0	0.0	3e-34	8e-84	1e-07	6e-47
*Tetraodon nigroviridis*	425	8.7	49.2	0.0	0.0	0.0	6e-32	6e-82	1e-05	9e-45
Tetrapoda										
*Gallus gallus*	429	8.6	49.0	0.0	-	0.0	3e-29	4e-79	7e-06	9e-42
*Xenopus tropicalis*	429	8.7	49.3	0.0	0.0	0.0	7e-32	3e-80	1e-06	7e-45
*Bos taurus*	430	8.8	49.5	0.0	0.0	0.0	1e-29	6e-80	2e-06	3e-41
*Canis familiaris*	430	8.8	49.5	0.0	0.0	0.0	1e-29	6e-80	2e-06	3e-41
*Homo sapiens*	430	8.8	49.4	-	0.0	0.0	1e-29	7e-80	2e-06	3e-41
*Macaca mulatta*	430	8.8	49.5	-	0.0	0.0	1e-29	7e-80	2e-06	3e-41
*Monodelphis domestica*	430	8.8	49.3	0.0	0.0	0.0	9e-30	6e-80	8e-07	2e-42
*Mus musculus*	430	8.9	49.5	0.0	0.0	0.0	2e-30	5e-79	9e-06	8e-41
*Pan troglodytes*	430	8.8	49.5	0.0	0.0	0.0	1e-29	6e-80	2e-06	3e-41
*Pongo pygmaeus*	430	8.8	49.5	0.0	0.0	0.0	1e-29	7e-80	2e-06	3e-41
*Pteropus vampyrus*	430	8.9	49.5	0.0	0.0	0.0	4e-29	7e-80	2e-05	8e-41
*Rattus norvegicus*	430	8.9	49.5	0.0	0.0	0.0	2e-30	5e-79	9e-06	8e-41
*Tursiops truncatus*	430	8.8	49.5	0.0	0.0	0.0	1e-29	7e-80	2e-06	3e-41

a DIA1 homologues were restricted to the Metazoa, but were not found in the phylum Porifera or Nematoda. Partial homologues, including those found in the phylum Mollusca and Annelida (Table S2), are not shown. See Table S1 for accession numbers of full length DIA1 proteins. Only a single *Drosophila* species DIA1 is included here. Table S3 is an expanded version of this Table.

b Isoelectric point calculated using the assumption that all residues have pKa values equivalent to that of isolated residues, and so may not accurately represent the value for the folded protein.

c Isotopically-averaged molecular weight prediction in kiloDaltons.

d The BLASTP E-value measures the statistical significance threshold for protein sequence matches. The smaller the number, the better the match. Computer shorthand nomenclature is used to present E-values when values are small. For example, 5e-01 = 0.5 and 5e-04 = 0.0005. Values below 1e-250 are indicated as zero, and details of those greater than 10 are not provided. A dash is used when protein alignments have 100% identity.

e Proteins were compared to DIA1from *H. sapiens*, *C. intestinalis*, *S. purpuratus*, or *D. melanogaster*, or *N. vectensis* by protein BLAST. The DIA1 proteins used for comparison were chosen as representives from Class Mammalia, Class Aves, and Class Neopterygii within the subphylum Vertebrata, subphylum Urochordata, phylum Echinodermata, phylum Arthropoda, and phylum Cnidaria, respectively.

**Figure 1:**
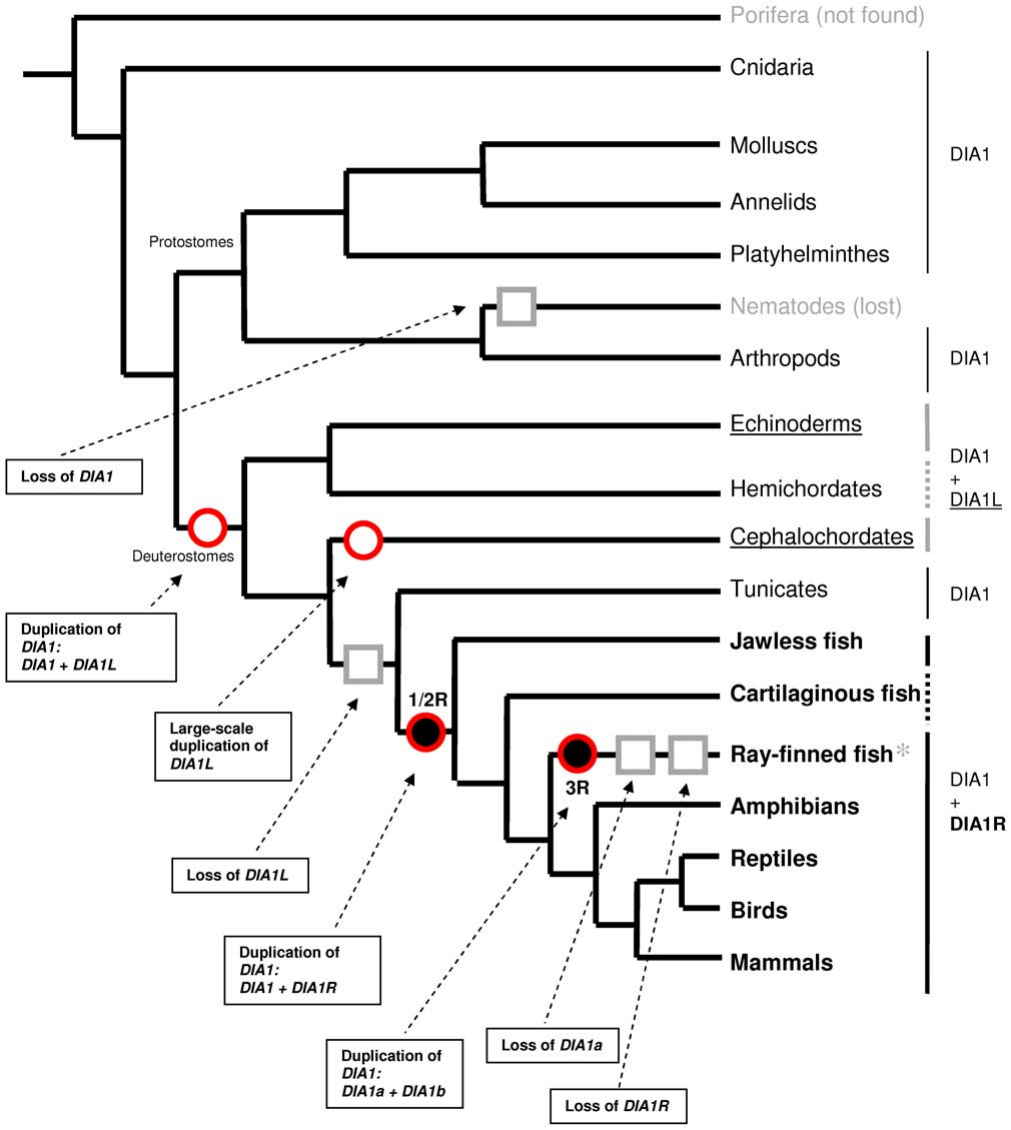
*DIA1*-family superimposed on a simplified metazoan phylogeny. *DIA1* is absent from the genome sequences of nematodes (grey font) as well as fungi, plants, amoebozoa and chromalveolates (not shown). Due to a paucity of sequence data, it is unclear whether a *DIA1* homologue is absent from the Porifera (grey font). *DIA1L* was exclusively found in echinoderm and cephalochordate genomes (underlined), which also encode *DIA1*. *DIA1L* is absent from tunicates, but a current dearth of sequence data precludes evaluation of hemichordate genomes for *DIA1L* homologues (indicated by a dotted bold grey line on right hand side, and a lack of underline). A bold dotted black line (right-hand side) indicates that the presence of *DIA1R* has been confirmed in cartilaginous fish but, probably due to a lack of sequence data, *DIA1* has yet to be identified in this class of chordates. Both a *DIA1* and *DIA1R* gene are present in vertebrate genomes (bold font), with a notable absence of *DIA1R* in acanthopterygian fish (asterisk). Furthermore, two *DIA1* paralogues were identified in the genomes of fish from the superorder Ostariophysi, but not in fish from other superorders (see Figure 3). Data for the schematic metazoan phylogeny were from numerous sources [157]–[163]. Proposed rounds of whole-genome duplication (WGD) are indicated by filled black spheres, where two WGDs occurred early in the vertebrate lineage (1R/2R) and a third WGD (3R) in the ray-finned fish lineage before the diversification of teleosts [43], [164], [165]. Proposed duplications of *DIA1*-family genes are indicated by red circles, and ‘loss’ of *DIA1*-family genes by grey squares. Dashed arrows are used to annotate events occurring in our current model of *DIA1*-family evolution. Further details of two different models of *DIA1*-family duplication and ‘loss’ events in the fish lineage (*) can be found in Figure 3, where some fish species encode *DIA1* paralogues, while others lack *DIA1R*. Accession numbers of *DIA1*, *DIA1R*, and *DIA1L* sequences can be found in Tables S1-S5, Table S7 and Table S9.

Species containing *DIA1* orthologues were restricted to a single eukaryotic supergroup: the Opisthokonta (Tables 1, S1-S3). *DIA1* orthologues were not found in any other eukaryotic supergroup (Plantae, Amoebozoa, Chromalveolata, Excavata, or Rhizaria) despite the availability of complete genome sequences from all supergroups, except the Rhizaria. Within the Opisthokonta, *DIA1* orthologues were not found in any fungal species and were restricted to the genomes of metazoan species. Within the Metazoa, a *DIA1* orthologue was not detected in sequence from the phylum Placozoa or phylum Porifera, possibly due to a scarcity of data. By contrast, orthologues were identified in species within the phylum Cnidaria, Mollusca, Annelida, Platyhelminthes, Echinodermata, Arthropoda, and Chordata (Tables S1 and S2). Within the phylum Chordata, *DIA1* orthologues were present in the subphylum Urochordata (sea squirt, *C. intestinalis*), Cephalochordata (lancelet, *Branchiostoma floridae*) and Vertebrata. *DIA1* orthologues were strikingly absent from the phylum Nematoda, including the completed genomes of *Caenorhabditis elegans* and *C. briggsae*. These findings are summarized in Figure 1.

### *DIA1* paralogues in zebrafish

The genome of each metazoan species encode only a single *DIA1* gene, with the exception of the zebrafish (*Danio rerio*) and minnow (*Pimephales promelas*) genomes, which encode two closely related *DIA1* genes (Tables 1, S1 and S2). We will refer to the closely related *DIA1* paralogues in these two fish species as *DIA1a* and *DIA1b*. ESTs for both *DIA1* paralogues were detected in the *D. rerio* and *P. promelas* EST databases (Tables S1 and S2), indicating expression of both paralogues, and arguing against one copy being a pseudogene of the other. By contrast, the ‘completed’ genomes sequence of pufferfish *Takifugu rubripes* (Fugu), *Tetraodon nigroviridis*, and the medaka *Oryzias latipes*, encode only a single *DIA1* orthologue (Tables S1 and S2).

**Figure 2:**
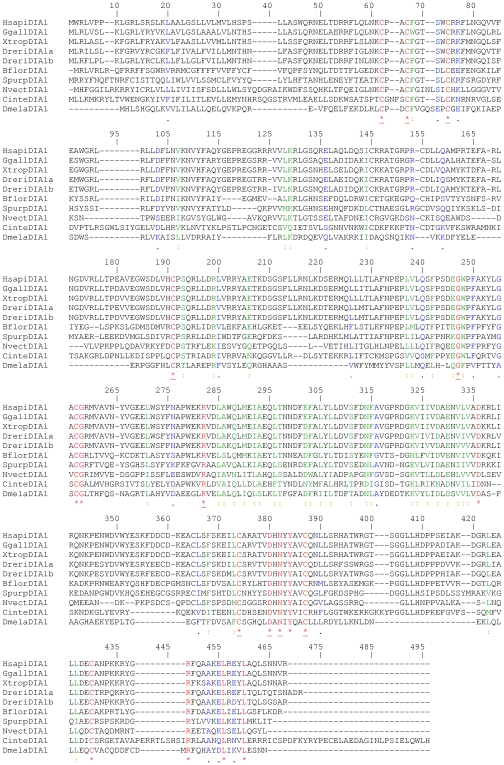
Amino acid sequence comparison of DIA1 from key species. The sequence alignment was generated using CLUSTALW [47]. Identical amino acids are highlighted in red font and indicated below the alignment with an asterisk (*). Strongly similar amino acids are highlighted in green font and indicated below the alignment with a colon (:). Weakly similar amino acids are highlighted in blue font and indicated below the alignment with a full stop (.). Dissimilar amino acids are in black font. Amino acids conserved in all DIA1 proteins, as determined by alignment of DIA1 gene products from all species (Figure S2), are underlined (*). Amino acid numbering is provided above the alignment. Gaps required for optimal alignment are indicated by dashes. Standard single-letter amino acid abbreviations are used. Organism abbreviations use the first letter of the genus name, followed by the first four letters of the species (e.g. *Homo sapiens* DIA1 is abbreviated to HsapiDIA1). The two *D. rerio* DIA1 paralogues are abbreviated as DreriDIA1a and DreriDIA1b. Full species names and accession numbers can be found in Table S1.

**Figure 3:**
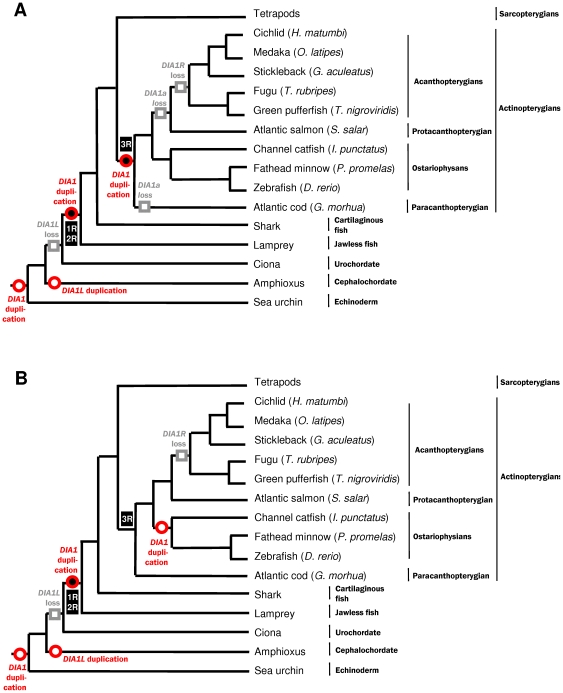
Fish-centric models of *DIA1*-family evolution. In both models (A and B), the genome of the hypothetical chordate ancestor encodes two *DIA1*-family genes: *DIA1* and *DIA1L*. The *DIA1L* gene has been ‘lost’ in the urochordate/vertebrate lineage, preceding the 1/2R whole genome duplications (WGDs). A duplicated copy of *DIA1*, which we have called *DIA1R*, was retained subsequent to the 1/2R WGD event, with both *DIA1* and *DIA1R* identified in lamprey, fish, and tetrapod genomes. In the fish lineage, however, two different models, (A) and (B), could account for our current knowledge of *DIA1* family members. In model (A), the *DIA1* duplication generating *DIA1a* and *DIA1b* coincides with the 3R WGD. Two lineage-specific ‘losses’ of *DIA1a* have then occurred: the first in the *G. morhua* lineage, and the second in the Protacanthopterygian/Acathopterygian lineage. There are too few data available to determine whether the channel catfish encodes *DIA1a*, *DIA1b*, both, or neither. In model (B), the *DIA1* duplication leading to *DIA1a* and *DIA1b* in ostariophysans does not coincide with 3R but, instead, is specific to the ostariophysan lineage. Both model (A) and (B) both predict *DIA1R* gene loss in the acanthopterygian lineage. Proposed rounds of WGD [43], [164], [165] are indicated by filled black spheres: numbering of the WGDs is provide in black boxes: those occurring early in the vertebrate lineage marked as 1R/2R and that in the ray-finned fish lineage marked as 3R. Proposed duplications of *DIA1*-family genes are indicated by red circles, and ‘loss’ of *DIA1*-family genes by grey squares. Data for the schematic fish phylogeny were from numerous sources [162], [166]–[169].

Amino acid alignments of the *D. rerio DIA1a* and *DIA1b* gene products reveal an overall 88/98% amino acid identity/similarity (Figures 2 and S1). At the nucleotide level, the mRNA sequences are 77% identical (Table S1). While only partial sequence is available for the *P. promelas DIA1* paralogues, amino acid alignments of the available sequences (see Table S2) reveal that *DIA1a* of *P. promelas* has greater similarity to *DIA1a* from *D. rerio* (than to *DIA1b* from *D. rerio*), while *DIA1b* of *P. promelas* is more similar to *DIA1b* from *D. rerio* (than to *DIA1a* from *D. rerio*). The available data support a model where ostariophysan fish, but not fish from other superorders, encode two functional, closely related *DIA1* paralogues. These findings, superimposed on a simplified fish phylogeny, are summarized in Figure 3.

### Comparison of DIA1 proteins

To compare DIA1 proteins with each other, we used three methods: (i) BLAST analyses, (ii) amino acid alignments, and (iii) phylogenetic analyses (see later). First, we used pair-wise protein BLAST analyses to generate ‘expect values’ (E-values) as a means of comparing the similarity between DIA1 orthologues (Table S3). All full-length *DIA1* gene products were compared to DIA1 from five key species. The DIA1 sequences used for comparison of all identified DIA1 proteins were those from a species representative of the phyla Cnidaria (*Nematostella vectensis*), Echinodermata (*Strongylocentrotus purpuratus*), Arthropoda (*D. melanogaster*), and Chordata, where a representative of both the subphyla Urochordata (*C. intestinalis*) and Vertebrata (*H. sapiens*) were included, with a representative from the class Mammalian, class Aves, and class Neopterygii for the latter subphylum. When proteins are compared using this method, the smaller the E-value, the greater the similarity between two compared proteins. E-values of greater than 1.0 are generally considered insignificant. Our analyses found significant pair-wise E-values between all of the *DIA1* gene products examined (Table S3). The only exceptions (where E-values were greater than 1.0) were in pair-wise comparisons between DIA1 from *C. intestinalis* and *Drosophila* species. By contrast, significant similarity (less than 1.0) was found between *C. intestinalis* DIA1 and DIA1 from all other insect species. We evaluate and discuss these similarities and differences in more detail later. Our comparisons therefore support the hypothesis that the DIA1 orthologues form a metazoan protein family.

Secondly, amino acid alignments were employed to compare *DIA1* gene products at the amino acid level. While in pair-wise comparisons vertebrate DIA1 proteins were between 78–100% identical and 97–100% similar (Table S4), an amino acid alignment of DIA1 proteins from all species revealed only 10 absolutely conserved amino acids (Figure S2): 3 cysteines in the amino-terminal portion; a cysteine glycine and arginine in the central region; and an asparagine, an aspartate and two further cysteines in the carboxy-terminal portion. Blocks of highly conserved regions of amino acids within the *DIA1* gene products were also identified, with the extreme amino-terminal region showing the greatest diversity (Figures 2 and S2). These alignments provide evidence that conserved regions of DIA1 are vital to the core biological function of the protein, and support the notion that this biological role is conserved in all metazoan species with a *DIA1* orthologue.

### Identification of *DIA1R* orthologues

The human gene currently annotated as *cXorf36* is closely related to *DIA1*, and has recently been renamed *DIA1R* [39]. The human *DIA1R* gene is X-linked, located on the short arm of the X chromosome at position Xp11.3, and has orthologues in at least four vertebrate species [39]. We carried out BLAST searches using the human DIA1R sequence and keyword searches (search term: cXorf36) to identify further *DIA1R* orthologues. We use the term ‘orthologue’, rather than ‘paralogue’, to describe those genes most similar to *DIA1* or to *DIA1R*, as *DIA1R* and *DIA1* proteins differ substantially at the amino acid level, and are predicted to have evolved specific, yet-related, cellular functions. Reciprocal BLAST searches were used to confirm that newly identified DIA1R sequences had greatest similarity to DIA1R, rather than to DIA1. Reciprocal BLAST searches also ensured all currently available DIA1 and DIA1R sequences were identified, and no false positives were included. Our findings are highlighted below.

Unlike *DIA1* orthologues, which were found in most metazoan phyla, *DIA1R* orthologues were restricted to the phylum Chordata. Furthermore, within the Chordata, *DIA1R* expression is restricted to the subphylum Vertebrata, including the early-branching lamprey species *Petromyzon marinus* (Tables S5 and S6). *DIA1R* was not found in subphylum Urochordata or Cephalochordata genomes. No evidence for *DIA1R* pseudogenes or closely-related paralogues was detected in any of the available sequence databases. All genomes encoding a *DIA1R* gene also had a *DIA1* gene, with the exception of the EST databases for cartilaginous fish species and a single ostariophysan species (*Ictalurus punctatus*), where partial *DIA1R* sequences (Table S6), but not *DIA1* sequence(s) was detected. Due to limited EST and genomic data for these species, we currently assume this absence is due to a lack of sequence data, rather than gene loss. Together, these data indicate that *DIA1R* arose from a *DIA1* gene duplication event occurring prior to the expansion of the vertebrates, which diverged from the cephalochordates around 520 million years ago [42], and this coincides with the well-documented whole-genome duplication events in the ancestral vertebrate, prior to vertebrate expansion [43].

### Absence of *DIA1R* in acanthopterygian fish

While *DIA1R* was detected in several fish species (Tables S5 and S6), there was a striking absence of *DIA1R* orthologues from the ‘completed’ genomes of acanthopterygian fish species including the genomes of *Gasterosteus aculateus* (stickleback), *O. latipes* (medaka fish), *T. nigroviridis* and *T. rubripes* (both pufferfish). By contrast, *DIA1R* was found in the genomes of fish from the superorders Ostariophysi (*D. rerio* and *I. punctatus*) and Protacanthopterygii (*Salmo salar*), although current EST data does not support the presence of *DIA1R* in the genome of another ostariophysan species, *P. promelas*. Conversely, while a *DIA1R* EST from the channel catfish (*I. punctatu*s) was identified, a corresponding *DIA1* EST was not found in this species. At present, it is mostly likely that the apparent absence of *DIA1R* in *P. promelas*, and *DIA1* in *I. punctatus*, is due to limitations in EST data, rather than because of gene loss. Therefore, two paralogues of *DIA1* are present in ostariophysan fish, but only a single *DIA1R* orthologue; while in the protacanthopterygian fish, we only find evidence for a single *DIA1* and *DIA1R* gene (Tables S1, S2, S5 and S6). By contrast, in acanthopterygian genomes, only a single *DIA1* gene is detected and *DIA1R* is absent. The timing of the *DIA1* gene duplication (to form *DIA1a* and *DIA1b*) and the loss of *DIA1R* in acanthopterygians can only be further delineated when additional fish sequence data becomes available. Nonetheless, these data provide evidence for both *DIA1* gene duplication and *DIA1R* gene loss events during evolution of teleost fish. Two models for *DIA1* and *DIA1R* evolution in teleost fish, are discussed later, and are summarized in Figure 3.

### Comparison of DIA1R proteins

To compare DIA1R proteins with each other, we used three methods: (i) BLAST analyses, (ii) amino acid alignments, and (iii) phylogenetic analyses. First, we used pair-wise protein BLAST analyses to generate ‘expect values’ (E-values) to assess the similarity between DIAR1 orthologues (Table 2). All full-length *DIA1R* gene products were compared with DIA1R from three key vertebrate species. The DIA1R sequences used for comparison with all identified full-length DIA1Rs were representative species from the subphylum Vertebrate; one each from Class Mammalia, Class Aves, and Class Neopterygii. Our analyses found significant pair-wise similarity (i.e. E-values less than 1.0) between the all of *DIA1R* gene products examined (Table 2). The highest pair-wise E-value (indicating the least similarity) was obtained when DIA1R from *S. salar* (Atlantic salmon) was compared to that of *Gallus gallus* (chicken), and was a value of 7e-77. As expected, each DIA1R protein was more similar to other DIA1R proteins, than to DIA1 proteins from the same species (Table 2). For example, the pair-wise E-value for DIA1R from *S. salar* compared to DIA1 from *G. gallus* was 1e-44, indicating less similarity to DIA1 than to DIA1R from the same species. Our comparisons therefore support the existence of a DIA1R-subfamily within the DIA1-family, where the subfamily genes are exclusive to the subphylum Vertebrata. Comparison of DIA1 to DIA1R gene products is discussed in detail in the section below.

**Table 2:** Physical characteristics of DIA1R proteins and similarity to orthologues from key species.

				BLASTP^d^ similarity to^e^:					
				DIA1R from-			DIA1 from-		
Species^a^	Length (amino acids)	pI^b^	Molecular mass^c^ (kDa)	*H. sapiens*	*G. gallus*	*D. rerio*	*H. sapiens*	*G. gallus*	*D. rerio (a)*
**METAZOA** f									
**Chordata**									
Vertebrata									
Neopterygii									
*Danio rerio*	417	8.7	47.2	1e-105	7e-81	-	2e-42	3e-44	1e-43
*Salmo salar*	453	8.7	50.5	6e-101	7e-77	1e-130	8e-41	1e-44	1e-42
Tetrapoda									
Aves									
*Gallus gallus*	430	8.7	49.0	3e-142	-	2e-85	1e-34	6e-35	1e-35
Mammalia									
*Bos taurus*	433	8.4	48.1	0.0	9e-128	8e-108	3e-40	5e-43	1e-43
*Dipodomys ordii*	434	7.8	48.5	0.0	2e-127	5e-102	2e-40	5e-42	1e-43
*Equus caballus*	433	8.8	48.7	0.0	3e-132	9e-109	2e-41	2e-42	3e-43
*Homo sapiens*	433	8.1	48.6	-	8e-136	9e-106	3e-41	2e-44	7e-45
*Macaca mulatta*	433	8.1	48.6	0.0	4e-134	1e-104	7e-42	4e-44	4e-45
*Monodelphis domestica*	432	8.6	49.0	0.0	1e-139	1e-110	8e-46	2e-47	1e-48
*Ornithorhynchus anatinus*	432	8.9	48.8	2e-168	3e-131	5e-103	8e-40	5e-40	7e-44
*Mus musculus*	435	8.1	49.0	0.0	4e-129	2e-104	4e-38	3e-40	3e-40
*Rattus norvegicus*	435	8.5	48.8	0.0	6e-129	4e-106	4e-42	2e-44	1e-44
*Sorex araneus*	431	8.8	48.1	0.0	6e-123	1e-100	2e-38	7e-41	1e-42

a See Table S5 for accession numbers of full length DIA1R proteins.

b Isoelectric point calculated using the assumption that all residues have pKa values equivalent to that of isolated residues, so may not accurately represent the value for the folded protein.

c Isotopically averaged molecular weight prediction in kiloDaltons.

d The BLASTP E-value (Expect value) measures the statistical significance threshold for protein sequence matches. The smaller the number, the better the match. Computer shorthand nomenclature is used to present E-values when values are small. For example, 5e-01 = 0.5 and 5e-04 = 0.0005. Values lower than 1e-250 are treated as zero. A dash is used when alignments have 100% identity.

e Proteins were compared to DIA1 (or DIA1a when paralogues were present) and DIA1R from *Homo sapiens, Gallus gallus* or *Danio rerio* by BLASTP. The proteins used for comparison were chosen as representatives from the Class Mammalia, Class Aves, and Class Neopterygii within the subphylum Vertebrata.

f DIA1R orthologues are only found in the subphylum Vertebrata and not in other subphyla or phyla. For example, DIA1R orthologues are not found in the phylum Nematoda, Platyhelminthes, Cnidaria, Echinodermata, or Arthropoda.

Secondly, we carried out amino acid alignments of all DIA1R orthologues (Figure S3 and Table S4). This comparison revealed a high level of amino acid identity and similarity between DIA1R proteins from all species, with an overall 27% amino acid identity and 53% similarity across gene products from all species. Poorest conservation was within the extreme amino-terminal portion, which forms a signal peptide (see later). Comparison between DIA1R gene products in a pair-wise manner revealed 45–97% identity and 76–97% similarity between each pair (Table S4). Further comparison of DIA1R proteins was carried using phylogenetic analyses and this data will be presented and discussed later.

### Comparison of DIA1 and DIA1R proteins

To compare DIA1R orthologues with their DIA1 counterparts, three approaches were used: (i) pair-wise amino acid alignments, (ii) amino acid alignments of all full length DIA1 and DIA1R proteins identified, and (iii) phylogenetic analyses. The phylogenetic analyses are provided later.

First, a detailed pair-wise comparison of DIA1 and DIA1R gene products was performed, using amino acid sequence from all species where both genes had been identified (Table S4). These analyses lead to two notable findings. (i) All DIA1R amino acid sequences are around 30% identical and 60% similar to their DIA1 counterpart of the same species (Table S4). (ii) The pair-wise amino acid identity between DIA1 homologues of two given species was always greater than that of the DIA1R proteins of those same species (Table S4). For example, while DIA1 from *H. sapiens* and *Macaca mulatta* (rhesus macaque) are 100% identical at the amino acid level, the DIA1R proteins from these same species are only 97% identical. Similarly, DIA1 from *Rattus norvegicus* (rat) and *Mus musculus* (mouse) are 100% identical at the amino acid level, while the DIA1R proteins from these same species are only 91% identical. The greater divergence of DIA1R, compared to DIA1, is more apparent when sequences from more evolutionary distant species are compared. For example, DIA1 from *H. sapiens* and *Gallus gallus* (fowl) are 90% identical (96% similar), but DIA1R proteins from these two species are only 65% identical (87% similar). These findings indicate greater evolutionary pressures favouring the conservation of *DIA1*, compared to *DIA1R*. More rapid evolution of one copy of a duplicated gene is a well-documented phenomenon [44], [45] and greater DIA1R divergence, than DIA1 divergence, is therefore expected.

**Figure 4:**
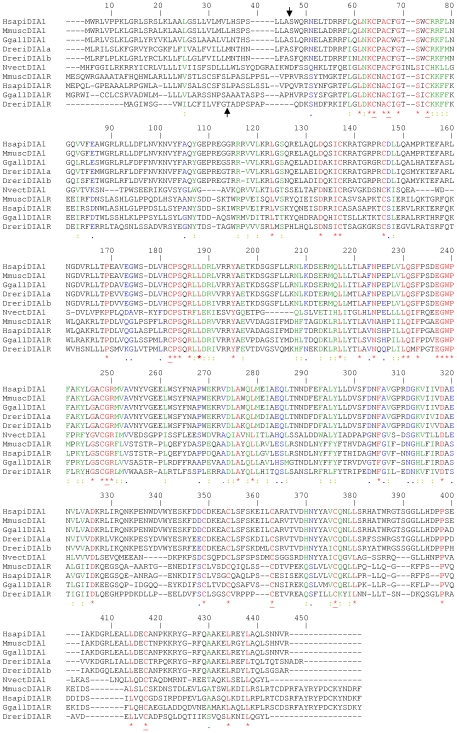
Amino acid sequence alignment of DIA1 and DIA1R proteins from key species. Gene products from species with known full-length *DIA1* and *DIA1R* orthologues were aligned using CLUSTALW [47], with DIA1 from the cnidarian species *Nematostella vectensis* (NvectDIA1), included for comparative purposes. Identical amino acids are highlighted in red font and indicated below the alignment with an asterisk (*). Strongly similar amino acids are highlighted in green font and indicated below the alignment with a colon (:). Weakly similar amino acids are highlighted in blue font and indicated below the alignment with a full stop (.). Dissimilar amino acids are in black font. Amino acids conserved in all DIA1 and DIA1R proteins, as determined by alignment of the DIA1 and DIA1R gene products from all species (Figure S4), are underlined (*). Amino acid numbering is provided above the alignment. Gaps required for optimal alignment are indicated by dashes. Standard single-letter amino acid abbreviations are used. Organism abbreviations use the first letter of the genus name, followed by the first four letters of the species (e.g. *Homo sapiens* DIA1R is abbreviated to HsapiDIA1R). Full species names and accession numbers can be found in Tables S1 and S4. Predicted signal peptide cleavage sites for human DIA1 and DIA1R (Figure S5) are indicated by arrows above or below the alignment, respectively.

Secondly, an amino acid alignment of all DIA1 and DIA1R proteins was created (Figure S4). This comparison found a total of eight amino acids that were absolutely conserved in DIA1 and DIA1R from all species, and these are highlighted in Figure 4. The conserved residues are: three cysteine residues in the amino-terminal portion, a cysteine and glycine residue in the central portion, and three cysteine residues in the C-terminal portion. Overall, there was greater amino acid conservation between DIA1 and DIA1R proteins in the central portion, which suggests this region mediates a key, conserved protein function.

### Tunicate DIA1 is similar to both DIA1 and DIA1R from other species

On examination of amino acid alignments of DIA1 and DIA1R gene products, it was noticeable that the *C. intestinalis* (a tunicate) sequence contained frequent amino acid insertions of various lengths, when compared to the DIA1 and DIA1R sequence from all other species (Figure S4). These insertions were most prominent in the amino- and carboxy-terminal portions of the *C. intestinalis* DIA1 protein, rather than the central region. We therefore investigated the relationship of *C. intestinalis* DIA1 with other DIA1 family members in greater detail.

First, pair-wise amino acid alignments of DIA1 of *C. intestinalis* with DIA1 and DIA1R of key vertebrate species (*H. sapiens*, *M. musculus*, *G. gallus*, *D. rerio*), were carried out (Table S7). These analyses revealed that *C. intestinalis* DIA1 was only marginally more similar to DIA1 than to DIA1R from other species, being on average 23.5% identical (55% similar) to the representative vertebrate DIA1 proteins, and 22% identical (55% similar) to the DIA1R proteins.

Secondly, reciprocal pair-wise E-values between *C. intestinalis* DIA1, and DIA1 and DIA1R of the same key vertebrate species (*H. sapiens*, *M. musculus*, *G. gallus*, *D. rerio*), were generated (Table S7). These analyses revealed that *C. intestinalis* DIA1 showed greater similarity (i.e. lower E-values) to DIA1, than DIA1R orthologues using the BLAST algorithm (Table S7), with E-values of around e-32 when compared to the vertebrate DIA1 proteins, but only e-25 when compared to the DIA1R proteins from the same species. These findings suggest that *C. intestinalis* DIA1 indels are causing difficulties when comparisons are made with other protein family members. Close examination of the *C. intestinalis DIA1* gene structure did not reveal any annotation errors contributing to the indels in the *C. intestinalis* DIA1 sequence, nor were any pseudogenes, or other *DIA1*-related sequences, detected in the *C. intestinalis* genome (data not shown). These results highlight the differing results that can be obtained using different alignment algorithms, such as BLAST [46] or CLUSTALW [47], and the intrinsic difficulties in obtaining optimal amino acid alignments for sequences with insertions. Our conclusions are that *C. intestinalis* contains a single copy of a divergent *DIA1* (not a *DIA1R*) gene, and that this divergence will impact on the phylogenetic relationships we later derive.

### Signal peptides in DIA1 and DIA1R orthologues

Human DIA1 and DIA1R, have amino-terminal signal peptides for protein targeting to the secretory pathway [39], [48]. Signal peptide functionality is supported by localization of DIA1 to the lumen of the Golgi apparatus [48]. We therefore analyzed all available *DIA1* and *DIA1R* gene products for signal peptides, and predicted signal peptide cleavage sites, using three of the best-performing algorithms [49], [50]. The methods used were: (i) the neural network algorithm of SignalP v3.0 [51], (ii) the hidden Markov model of SignalP v3.0 [51], and (iii) the Sigcleave algorithm at EMBOSS [52]. Use of multiple algorithms also gives us the maximal possible confidence about the results, if they concur. No trans-membrane domains were predicted, nor ER-retrieval or retention motifs detected (data not shown).

Our analyses of 35 full length *DIA1* orthologues revealed signal peptides (SPs) in gene products from all species, using all three prediction methods (Figure S5). Furthermore, concordant SP cleavage sites were predicted by all three methods for DIA1 proteins from 6 species, with very similar cleavage sites predicted for DIA1s from a further 11 species (i.e. where 2 of the 3 methods predicted a cleavage site aligning with the concordant site, and the remaining method predicted the adjacent amino acid as the cleavage site). As similar cleavage site prediction results were obtained for DIA1 proteins from all tetrapod species, the cleavage site for tetrapod DIA1 cleavage is predicted to be conserved, and to occur after alanine-37 (using *H. sapiens* DIA1 numbering). By contrast, the signal peptide cleavage site for DIA1 from *Drosophila* species is predicted to be after proline-24 (using *D. melanogaster* DIA1 numbering). While there is a consensus for SP presence in DIA1s from other species, there was no consensus for the cleavage site (Figure S5). Less concordance in prediction of the cleavage site for these species may reflect a current lack of information about the SP recognition process in such species. The majority of model proteins on which the prediction algorithms are based are from tetrapods, and less is known about cleavage of signal peptides in proteins from other species.

Our analyses of DIA1R orthologues likewise revealed the presence of SPs in *DIA1R* gene products from all species using all three prediction methods (Figure S5). While there was a consensus for SP presence in all DIA1Rs, there was no consensus for the position of the cleavage site, as only two species showed concordance in cleavage sites prediction from all three methods. Based on our data, the best prediction for the SP cleavage site for mammalian DIA1R is after serine-31 (using *H. sapiens* DIA1R numbering). Overall, we show SPs are predicted for both DIA1 and DIA1R from all species, however the exact amino acid composition of luminal DIA1 and DIA1R gene products will require experimental validation. Such data will be of benefit for refining the algorithms used for signal peptide cleavage sites, particularly in non-tetrapod species. Our data indicate that translocation into the ER and transportation to the Golgi apparatus will be a common property of all *DIA1* and *DIA1R* gene products.

### *DIA1*-like homologues in amphioxus and echinoderm

BLAST searches of the non-redundant (NR) database revealed the presence of additional, *DIA1*-like genes in some species, which we refer to as *DIA1L*, for *DIA1-like* gene. These gene products were restricted to the genomes of *S. purpuratus* (an echinoderm) and *B. floridae* (a cephalochordate). Initially, we thought these products may be due to annotation errors or be pseudogenes. Indeed, analysis of the annotated *S. purpuratus DIA1L* gene did reveal a splicing error in the gene model, which we corrected (Table S8 and Figure S10). The EST database provides evidence that the *S. purpuratus DIA1L* gene is expressed (Table S8).

By contrast to the echinoderm genome, which has a single *DIA1L* gene, three full-length *DIA1L* paralogues were identified in the *B. floridae* genome, which we have called *DIA1La*, *DIA1Lb*, and *DIA1Lc* (Table S8). Of the three full-length *B. floridae DIA1L* genes, two contain introns, indicating they are not processed pseudogenes (Table S8). EST data supports the expression of *DIA1Lb* and the intron-less *B. floridae DIA1Lc* gene (Table S8). For the latter, this indicates the lack of introns is due to intron-loss events, rather than *DIA1Lc* being a pseudogene. For *B. floridae*, EST sequencing is still very much a work in progress, and this may explain the current lack of expression data for the amphioxus *DIA1La* gene. Together these data indicate a duplication of *DIA1* early in the deuterostome lineage, generating what is now *DIA1L* (Figure 1). However, we cannot rule out an alternative hypothesis: that the duplication event generating *DIA1L* preceded protostome-deuterostome divergence, and that *DIA1L* was lost early in the protostome lineage. Strikingly, while the echinoderm genome encodes a single copy of *DIA1* and of *DIA1L*, the cephalochordate genome encodes a single copy of *DIA1* and multiple copies of *DIA1L*. This indicates cephalochordate lineage-specific duplication of *DIA1L*. A lack of *DIA1L* in later-branching deuterostomes, indicates *DIA1L* homologues were ‘lost’ prior to tunicate divergence.

### Comparison of *DIA1L* and *DIA1* gene products

Amino acid alignments reveal *S. purpuratus* DIA1 and DIA1L have approximately 15% identical amino acids while, overall, 40% of aligned amino acids are similar (Table S9 and Figure S7). The corrected *S. purpuratus DIA1L* gene product is 636 amino acids in length (Table S8), compared to 431 residues for the homologous *S. purpuratus DIA1* gene product (Table 1; Table S1). The *B. floridae DIA1La* gene product is 483 amino acids in length and the *B. floridae DIA1Lb* gene product 539 residues, again both longer than DIA1 (418 amino acids) of the same species. By contrast, *B. floridae* DIA1Lc is shorter (398 amino acids) than the parental *DIA1* gene product. *B. floridae* DIA1La, DIA1Lb and DIA1Lc are all approximately 20% identical (55% similar) to each other at the amino acid level (Figure S7; Table S9) and *S. purpuratus DIA1L* is most similar to *B. floridae DIA1Lc* (∼60% similar/20% identical). By contrast to the variability between *DIA1L* gene products, the *DIA1* gene products of *S. purpuratus* and *B. floridae* show greater similarity to each other (∼75% similar/40% identical). This finding indicates greater evolutionary pressure favouring the conservation of DIA1 sequences, compared to that favouring sequence conservation in the duplicated *DIA1L* gene(s), over the same evolutionary time-period.

### Conservation within the DIA1 protein family

**Figure 5:**
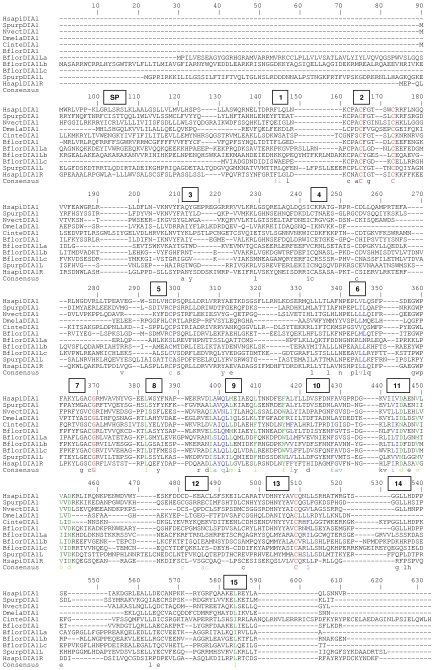
Amino acid sequence alignment of DIA1-family proteins from key species. All full-length *DIA1*, *DIA1R*, and/or *DIA1L* gene products were aligned using CLUSTALW (Figure S7), and this figure represents excerpts from this master alignment, where proteins from the following phyla only are represented: Cnidaria (*N. vectensis* DIA1: NvectDIA1), Arthopoda (D. *melanogaster* DIA1: DmelaDIA1), Echinodermata (*S. purpuratus* DIA1 and DIA1L: SpurpDIA1 and SpurpDIA1L), Cephalochordata (*B. floridae* DIA1 and DIA1L paralogues: BflorDIA1, BflorDIA1La, b, and c), and Chordata. The latter includes representatives of the subphylum Urochordata (*C. intestinalis* DIA1: CinteDIA1) and subphylum Vertebrata (*H. sapiens* DIA1 and DIA1R: HsapiDIA1 and HsapiDIA1R). Amino acid numbering from the master alignment (Figure S7) is provided above the alignment. Gaps required for optimizing the master alignment (Figure S7) are indicated by dashes. Standard single-letter amino acid abbreviations are used. Organism abbreviations use the first letter of the genus name, followed by the first four letters of the species (e.g. *Homo sapiens* DIA1R is abbreviated to HsapiDIA1R). Full species names and accession numbers can be found in Tables S1, S4 and S7. The predicted location of the DIA1 and DIA1R signal peptides (SP) are indicated above the alignment (Figure S5). Conserved amino acid motifs detected in the master alignment (Table 3, Figure S7) are indicated in numbered boxes above the alignment. Consensus amino acids for each motif are indicated below the alignment. Amino acids absolutely conserved across the whole DIA1-family are indicated in red upper-case letters, those strongly conserved across the whole DIA1-family are in green, and those weakly conserved across the whole DIA1-family in blue (Figure S7). In addition, black lower-case letters indicate amino acids conserved in over 80% of DIA1-family sequences (Figure S8), while grey lower-case letters indicate conservation in 50–80% of DIA1-family sequences (Figure S9).

To determine key amino acids conserved across all members of the DIA1 protein family, amino acid alignments of *DIA1*, *DIA1R*, and *DIA1L* gene products were performed (Figure S7). Absolute conservation of four residues across the whole DIA1-protein family was found. These residues were: two cysteines in the amino-terminal region, a central glycine residue, and a further cysteine in the carboxy-terminal region of the proteins (Figure S7). In total, 15 highly conserved motifs, can be delineated (Table 3), however hydrophobic motif 1 is weakly conserved in DIA1L proteins and *Drosophila* DIA1 proteins, motif 12 is absent from DIA1L proteins, motifs 4 and 14 are absent from *Drosophila* DIA1 proteins, and motif 14 is only weakly conserved in DIA1R and DIA1L proteins (Figures S7–S9). We are currently investigating further the structure and function of the identified DIA1-family motifs. Overall, greatest amino acid similarity is found in the central portion of the extended DIA1 family (Figure 5), indicating a core role for this region in function, not only of DIA1 and DIA1R, but also of DIA1L. The relationship between DIA1-family gene products is examined further below, using phylogenetic methods.

**Table 3:** DIA1-family motifs.

Motif*	Alignment consensus**	Human DIA1 sequence***	Comment
1	F-L-x-L	FLqL	This motif is conserved in DIA1 and DIA1R proteins, but less-strongly conserved in DIA1L proteins (which are not targeted to the secretory pathway). The penultimate leucine in the motif is absent from *Drosophila* DIA1 proteins.
2	C-x-A-C-x-G-x(3,5)-C	CpACfGtswC	Motif contains two of the absolutely conserved DIA1-family residues.
3	A-x-Y-x(6,15)-L	AqYgepreggrrrvvklrL	The tyrosine is conserved in 73% of DIA1-family members. In some family members it is replaced by a tryptophan or phenylalanine, or there is an adjacent (or nearby) residue that is a tyrosine.
4	I-C-x(8,10)-C	ICkratgrprC	Absent from DIA1 from *Drosophila* species.
5	V-x(4,11)-C-x-S-x(6,10)-Y-x-E	VegwsdlvhCpSqrlldrlvrrYaE	The tyrosine is conserved in 85% of DIA1-family proteins.
6	L-x(3)-L-x-x-N-x-x-P-L-V-L-Q	LlltLafNpePLVLQ	The proline and glutamine residues of this motif are absent from DIA1L proteins from amphioxus.
7	G-W-P-x(5)-G-x-C-G	GWPfakylGaCG	Motif contains one of the absolutely conserved DIA1-family residues.
8	L-x-x-Y	LwsY	The tyrosine is conserved in 77% of DIA1-family members. In some family members it is replaced by a tryptophan or phenylalanine, or there is an adjacent (or nearby) tyrosine residue.
9	R-x-D-L-A-x-Q-L-M-x-I-x(3)-L	RvDLAwQLMeIaeqL	The first amino acid (R) of this motif is poorly conserved in DIA1R proteins.
10	F-x-L-Y-x-x-D-x(5)-F-A-V	FaLYlldvsfdnFAV	The tyrosine is conserved in 73% of DIA1-family members. In some family members it is replaced by a tryptophan or phenylalanine, or there is an adjacent (or nearby) residue that is a tyrosine. The aspartate of this motif is absent from DIA1R proteins.
11	K-V-x-I-D-x-E-x-V-x-V-x-D	KViIvDaEnVlVaD	The central glutamate is not conserved in DIA1R proteins.
12	C-x(3,4)-A-C-x(6,8)-C	CdkeAClsfskeilC	The final cysteine is well-conserved, but the remainder of motif is poorly conserved in insect and tunicate DIA1. Motif absent from DIA1L proteins.
13	[D-x-N-x-Y-x-x]-C-x-x-L-L	[DhNyYav]CqnLL	Motif contains one of the absolutely conserved DIA1-family residues. An expanded, tyrosine-containing motif [in square brackets] is found in this position in DIA1 and DIA1L, but not DIA1R, proteins (see Figures S8 and S9).
14	G-x-L-H-x(3,4)-E	GlLHDPPsE	Motif found in DIA1 only, with the exception of *Drosophila* DIA1 proteins. Absent from DIA1R and DIA1L.
15	L-x-E-x(16,18)-L	LdEcanpkkrygrfqaakeL	Consensus is conserved in more than 80% of DIA1 family but, while the final leucine is highly conserved, the first leucine is absent from 75% of DIA1R proteins. The charged residue is poorly conserved in DIA1 of insects.

*Motifs numbered in amino- to carboxy-terminal direction.

**Consensus motif is that from the Boxshade consensus line using 80% similarity threshold (Figure S8), unless otherwise indicated. Underlined residues  = 100% conserved. Standard single-letter amino acid abbreviations are used, where x = any amino acid, and x(6,8) indicates 6, 7, or 8 poorly/non-conserved amino acids present in that position.

***Motif-conforming residues are in upper case; poorly or non-conserved amino acids in lower case.

### DIA1L proteins lack signal peptides

By contrast with both *DIA1* and *DIA1R* gene products (Figure S5), DIA1L proteins do not encode predicted signal peptides (data not shown). As for DIA1 and DIA1R, we analyzed *DIA1L* gene products for the presence of trans-membrane domains and, similarly, none were detected (data not shown). We therefore conclude that while DIA1 and DIA1R can enter the endoplasmic reticulum, DIA1L proteins cannot, and will fulfil a cytosolic function. The altered sub-cellular localization of DIA1L compared to the parental DIA1 may contribute to the sequence divergence of DIA1L proteins compared to that of DIA1 proteins (Table S9). Subcellular relocalization has previously been described as a mechanism for duplicate gene retention [53]–[55] and as a factor contributing to asymmetric sequence divergence [56].

### Identification of further *DIA1L* homologues in amphioxus

While a single *DIA1L* homologue was found in the echinoderm genome, and three full-length *DIA1L* homologues in the cephalochordate *B. floridae* (Table S8), we also found evidence that further *DIA1L* homologues may exist in the *B. floridae* genome. In the first assembly (August, 2009) of the *B. floridae* genome, 14 incomplete *DIA1L* genes were annotated. In the most recent assembly (October, 2009), we found evidence for five partial *DIA1L* genes (Table S10), in addition to the 3 full-length amphioxus *DIA1L* homologues. Our analyses (below) suggest the number of *DIA1L* homologues, however, will be eight, as some of the partial *DIA1L* genes represent redundant allelic copies (Table S10). Further analysis is hampered by gaps in the genomic sequence.

The currently annotated five incomplete *DIA1L* genes in the *B. floridae* genome were identified by BLAST searches of the NR database (Table S10). We have numbered the partial (*DIA1L*-pt) genes *DIA1L-*pt1–*DIA1L-*pt5, for discussion purposes. Two of the *DIA1L* partial genes, *DIA1L*-pt3 and *DIA1L*-pt4 are similar to each other and share synteny (Table S10), and will most likely prove to be alleles of each other (and be re-annotated in a future assembly of the *B. floridae* genome). Expression of *DIA1L*-pt3 and its proposed allele *DIA1L*-pt4 is supported by EST data (Table S10) but, at present, expression data for *DIA1L*-pt5 is lacking. *DIA1L*-pt1 and *DIA1L*-pt2 are annotated as adjacent genes encoded on opposite DNA strands. A single EST with 93% identity to both *DIA1L*-pt1 and *DIA1L*-pt2 suggests expression of at least one of these genes (Table S10). However, the significance of a lack of expression data for *DIA1L*-pt5 is unclear, as it must be appreciated that, for *B. floridae*, EST sequencing is still limited. Therefore, while a duplication of *DIA1* early in the deuterostome lineage generated *DIA1L*, there has been large-scale lineage-specific expansion of the *DIA1L* in the cephalochordate lineage. Strikingly, despite the retention of *DIA1L* homologues in echinoderms and cephalochordates subsequent to this gene duplication event, *DIA1L* is absent in all later-branching deuterostomes, indicating *DIA1L* was ‘lost’ or diverged dramatically (precluding detection) prior to tunicate divergence (see Figure 1).

### A non-processed *DIA1* pseudogene in the mosquito *C. pipiens*?

The *C. pipiens* genome contains a second *DIA1*-like gene (Table S1) classified as putatively translated on the NR-database (accession number XP_001867819). However, there is some evidence for re-classification of the *C. pipiens* gene as a non-processed pseudogene (Table S1) and we are currently investigating this possibility in more detail. The presence of non-processed pseudogene provides evidence of a past lineage-specific *DIA1* gene duplication event in *C. pipiens*. No non-processed or processed pseudogenes were detected in other species using genomic BLAST searches.

### *DIA1* family phylogeny

**Figure 6:**
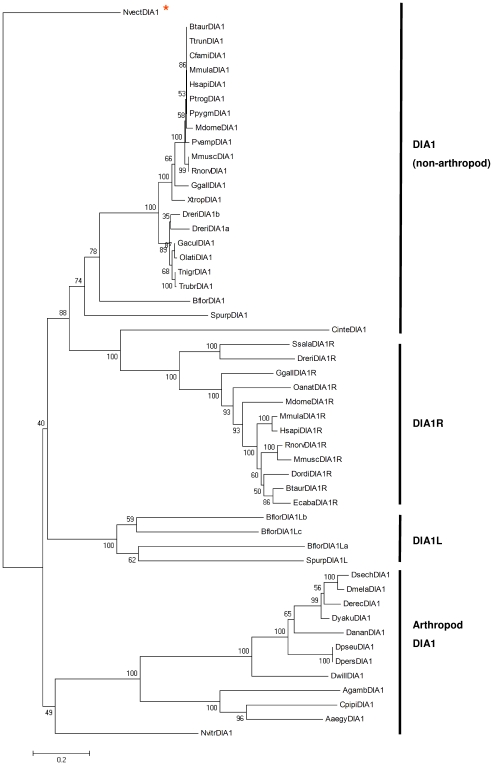
Evolutionary relationships between DIA1-family members. The evolutionary history of the DIA1 family was inferred using the neighbour-joining method [57]. The optimal tree is shown, with statistical reliability of branching assessed using 1000 bootstrap replicates [155], where percentage values are shown next to the branches. The tree is drawn to scale, with branch lengths in the same units as those of the evolutionary distances used to infer the phylogenetic tree. The evolutionary distances were computed using the Poisson correction method [170] and units are the number of amino acid substitutions per site. All positions containing gaps were eliminated from the dataset (Figure S10). There were a total of 258 positions in the final dataset. Phylogenetic analyses were conducted in MEGA4 [150]. The tree was rooted on the cnidarian *N. vectensis* DIA1 sequence (NvectDIA1), as highlighted with an asterisk. Organism abbreviations use the first letter of the genus name, followed by the first four letters of the species. Full species names and accession numbers can be found in Tables S1, S4 and S7.

To assess the evolutionary relatedness of the *DIA1* family genes, a phylogenetic tree, based on the alignment of the amino acid sequences of all *DIA1* homologues (Figure S7) was generated, using a distance-based neighbour-joining method [57]. Figure 6 illustrates our current knowledge of the evolutionary relationship between *DIA1*, *DIA1R*, and *DIA1L*. Four key features were highlighted by these analyses. (i) *DIA1R* orthologues cluster with each other, supporting the hypothesis of a gene duplication, and subsequent divergence of the duplicated gene. (ii) Evidence for a second *DIA1* duplication in the teleost lineage is highlighted by the clustering of the two *DIA1* paralogues from *D. rerio* first with each other, rather than with *DIA1* from other vertebrates. By contrast, the *D. rerio DIA1R* orthologue clusters with *DIA1R* of other vertebrates, not with the *D. rerio DIA1* paralogues. (iii) Clustering of the *DIA1L* gene products of sea urchin (*S. purpuratus*) and amphioxus (*B. floridae*) together, supports the scenario of a *DIA1* gene duplication early in the deuterostome lineage, followed by a lineage-specific expansion of *DIA1L* in *B. floridae*. (iv) DIA1 from the sea squirt, *C. intestinalis,* did not branch in the position expected from the known phylogenetic relationship of species (Figure 2). Our neighbourhood-joining analysis found *C. intestinalis* DIA1 clustering with the DIA1R orthologues, rather than the DIA1 orthologues, of other species (Figure 6). However, *C. intestinalis* does not encode a *DIA1R* gene, and has only one *DIA1* gene, although the resulting gene product has characteristics intermediate to DIA1 and DIA1R of other species (see above). The unusual branching of *C. intestinalis* DIA1 may be due to one or more of the following factors: loss of a duplicated gene, convergent evolution, and/or limitations in the phylogenetic reconstruction method. Use of other amino acid alignment methods (data not shown) and different methods of phylogenetic reconstruction, such as maximum-likelihood (Figure S11) or Bayesian analysis (Figure S12), did not alter the branching of the *C. intestinalis DIA1* gene to that consistent with the current evolutionary model of species.

In summary (Figures 1 and 6), the earliest-branching metazoan in which *DIA1* was detected was the cnidarian, *N. vectensis*. The origin of *DIA1L* can be traced back to early in the deuterostome lineage, where a *DIA1* gene duplication event and subsequent divergence occurred. In the cephalochordate lineage, large-scale, lineage-specific duplication of *DIA1L* has occurred, leading to an estimated 7 copies of *DIA1L* in the *B. floridae* genome. *DIA1L* loss (or divergence precluding detection) then occurred prior to urochordate branching, and *DIA1L* homologues are not detected in urochordates or vertebrates. The origin of *DIA1R* (via *DIA1* duplication) coincides with the two WGDs occurring early in vertebrate evolution, and humans and other vertebrates have two *DIA1* homologues: *DIA1* and *DIA1R*. By contrast, further *DIA1* gene duplication has occurred in the fish lineage which may have coincided with the teleost WGD, or alternatively an event specific to the ostariophysan lineage (Figure 3). Loss of *DIA1R* has occurred in the acanthopterygian fish lineage. Together, our data support the hypothesis that multiple gene duplication and gene ‘loss’ events have occurred during the evolution of the *DIA1* family. Three subfamilies of expressed extant genes exist that share a number of common motifs: *DIA1*, *DIA1R*, and *DIA1L*. The *DIA1* and *DIA1R* gene products from all species are targeted to the lumen of the secretory pathway and *DIA1L* gene products to the cytoplasm. Animal models may be useful in understanding why defective *DIA1* and *DIA1R* gene products cause ASD and/or mental retardation.

## Discussion

Defective human *DIA1* and *DIA1R* genes, despite their ubiquitous tissue expression, are implicated in the etiology of autism, autism-like syndromes, and/or mental retardation [38], [39]. Little is known about *DIA1* or *DIA1R*, except that the genes are ubiquitously expressed and they encode signal peptides for targeting to the secretory pathway, with DIA1 localizing to the lumen of the Golgi apparatus [39], [48]. Here we confirmed the presence of a signal peptide for secretory pathway targeting in *DIA1* and *DIA1R* gene products from all species, and have studied the evolutionary history of the *DIA1* gene, finding its emergence coincided with the development of the early nervous system. By contrast, the closely related *DIA1R* gene is exclusive to vertebrate genomes. We further identify a related gene *DIA1L* in echinoderm and amphioxus genomes only, where the gene products are predicted to be cytoplasmically-targeted. Therefore, the recently identified human *DIA1* gene is part of a larger, evolutionarily-related gene family.

### *DIA1* evolution coincided with early nervous tissue development

We found *DIA1* to be conserved from cnidarians to humans, but we found no evidence for *DIA1* in the currently available poriferan sequence databases. Cnidarians have a basic nervous system, but are constructed on principles similar to those of complex metazoans, including the formation of organized nerve networks [58]. The cnidarian apical pole is considered a primitive head, and it is currently thought that nervous tissue first evolved in cnidarians or a closely-related ancestor [58], [59]. Therefore, the detection of *DIA1* coincides with the evolution of a neuronal network, providing circumstantial evidence for a key role of DIA1 in neuronal function.

### Absence of *DIA1* in nematodes

Unexpectedly, *DIA1* was absent in nematodes, including the completed genomes of *C. elegans* and *C. briggsae*. There are two possible explanations for this finding: (i) nematodes have lost the *DIA1* gene and have no need for an equivalent gene; or (ii) a nematode *DIA1* is still present, but it is undetectable due to evolutionary divergence. This apparent nematode-specific gene loss is not without precedent: it has been found that, while 20% of *S. purpuratus* genes are found in fruit fly, only 15% are present in nematode. Furthermore, while *S. purpuratus* has members of 97% of the human kinase subfamilies, while *Drosophila* lacks 20%, and nematodes 32% [60]. Indeed, more than half of the putative nematode genes are unique to the phylum, with 23% being species-specific [61]. Even within the nematode phylum, organisms only share only around 60% of their genes [61]. Finally, a study of ESTs from the Cnidarian *Acropora millepora* showed that over 10% of the *Acropora* ESTs with clear human homologs, have no representatives in the *Drosophila* or *Caenorhabditis* genomes [62]. Clearly, secondary gene loss in the nematode lineage is becoming an increasingly well-documented phenomenon.

The hypothesis that the lack of detection of *DIA1* in nematode genomes is due to divergence, however, is also supported by the rapid evolutionary rates reported in the nematode lineage [63]–[66], with genome rearrangements occurring approximately four times faster in the worm than in the fly [67] and 50-fold the rate in vertebrates [68]. Our finding of *DIA1* in arthropod genomes, but its absence in nematode genomes, is not without precedent as only 35% of genes in *C. elegans* and *D. melanogaster* are considered orthologous [69]. Therefore, while nematodes have provided insights into many areas of neurobiology, there are also nematode-specific phenomena. A lack of a *DIA1* homologue in nematodes may be related to some of the differences in neurobiology found in nematodes compared to other metazoans [70]–[74]. Our overall model is that *DIA1* evolved in an ancestor of the cnidarians and, despite being ubiquitously expressed in mammalian tissues [39], [41], [75], gene loss has a marked impact on neurological function in humans, thereby probably causing the symptoms of ASD. Whatever the secretory-pathway role of *DIA1* is, it is now defunct or significantly different, in nematodes.

### Evolution of the *DIA1*-related gene *DIA1R*

We have recently described a ubiquitously-expressed *DIA1*-related gene in the human genome, *DIA1R*, where deletion or mutation is linked to ASD-like syndromes and/or X-linked mental retardation [39]. Unlike *DIA1*, which we found present in the genome of most metazoans, we found *DIA1R* to be restricted to the subphylum Vertebrata within the phylum Chordata, and *DIA1R* is absent from urochordate and cephalochordate genomes. Therefore, the emergence of *DIA1R* coincides with the timing of the known two whole genome duplication (WGD) events, which occurred early in the vertebrate lineage around 500 million years ago [43], [76]–[79]. These WGDs preceded the dramatic rise of vertebrate life that occurred during the Cambrian explosion and coincided with the development of larger brains, a neural crest, and cranial placodes. Indeed, genome duplications often precede species-expansion, although links between genome duplication and increased species diversity remains correlative [80]. Certain genes were preferentially maintained after the vertebrate WGD events including transporters and kinases, with many being involved in signal transduction and development [81]–[83]. This provides circumstantial evidence for the involvement of *DIA1*-family genes in such processes. Together, these data indicate that *DIA1R* is a post-2R gene with a role in brain function.

### Identification of a cytosolic homologue of *DIA1*: *DIA1L*

*DIA1*-like homologues, termed *DIA1L*, were detected in the genomes of *S. purpuratus* (an echinoderm) and *B. floridae* (a cephalochordate). Our data suggest an origin of *DIA1L* by *DIA1* duplication early in the deuterostome lineage. Other gene families known to have diversified by gene duplication subsequent to the protostome-deuterostome divergence include the myosin light chain family [84], and the ST8Sia family of sialyltransferases [85]. Unlike both *DIA1* and *DIA1R*, *DIA1L* proteins were unexpectedly found to lack signal peptides, and are therefore predicted to have a cytosolic location and function. There are a number of possible explanations for the restriction of *DIA1L* to echinoderm and cephalochordate genomes: (i) *S. purpuratus* and *B. floridae* are unique in their requirement for a DIA1-family role in both the cytoplasm and secretory pathway, resulting in subsequent loss of the gene in other species; (ii) Other species require cytoplasmic DIA1-family activity and achieve this by currently uncharacterized splicing events; or (iii) The *DIA1L* gene diversified rapidly after divergence of cephalochordates and is no longer recognizable as a *DIA1L* homologue in extant chordate genomes. While we currently cannot differentiate between these hypotheses, we favour the latter hypothesis, as the central portion of *B. floridae DIA1La* shows weak amino acid similarity to an as-yet-uncharacterized human gene product (data not shown).

### Intensive gene duplication pre-dating vertebrate *DIA1* duplication

While the echinoderm, *S. purpuratus*, encodes a single *DIA1* and a single *DIA1L* gene, the amphioxus genome encodes a single *DIA1* gene, and multiple *DIA1L* genes. While three of the *DIA1L* genes in *B. floridae* are well-documented, the current genome assembly also provides evidence for a further 5 *DIA1L* genes (although two of these may be allelic). These data provide evidence for a large-scale expansion of *DIA1L* specific to the amphioxus lineage. The genome of *B. floridae* was previously considered relatively unduplicated [86], [87], however more recent evidence documents large-scale duplication of genes from certain functional categories in the amphioxus lineage. These include nuclear hormone receptors [88], opsins [42], tyrosine-kinase superfamily genes [89], and receptors and receptor-adaptors of the innate immune system [90]. It has therefore been suggested that the vertebrate WGD events, occurring subsequent to amphioxus divergence, were symptoms of a pre-existing predisposition toward genomic structural change [78]. Our data support the hypothesis that the early vertebrate WGDs were preceded by remarkable gene-family expansion and genome rearrangements.

### Duplication and loss of *DIA1-*family genes in teleost fish

Two closely-related *DIA1* paralogues were found in the genome of the ostariophysan fish *D. rerio* and *P. promelas: DIA1a* and *DIA1b*. There are two possible explanations for this finding: (i) origin of the duplicated *DIA1* gene during the fish-specific WGD (also known as ‘3R’), with retention in the ostariophysan lineage, but loss of the duplicated in gene in both the paracanthopterygian and acanthopterygian/protanthopterygian fish lineages, or (ii) a *DIA1* gene duplication event occurring early after divergence of the ostariophysan lineage, with maintenance of the duplicated gene in this lineage. The two possible evolutionary scenarios are superimposed on a fish-centric phylogenetic tree in Figure 3. There are current precedents for complicated evolutionary pathways for other teleost genes. Both the Elopomorpha, Ostariophysi, Salmoniformes, and Acanthipterygii have lineage-specific duplications of the vitellogenin (*Vtg*) genes occurring after the 3R WGD event (our unpublished data; [91]). By contrast, two independent losses of the androgen receptor-B (*AR-B*) gene, subsequent to the 3R WGD, have occurred, one at the base of the Otophysi and another at the base of the Salmoniformes [92]. The precise evolutionary history of the *DIA1* paralogues in fish cannot be reconstructed until significantly more fish sequence data become available.

Why ostariophysan fish have retained three *DIA1*-family genes is unclear. Features specific to ostariophysan fish include: small, horny projections called unculi; a bony Weberian apparatus; the release of a pheromone known as the alarm substance, when frightened; and highly social behaviour [93]. As we propose the *DIA1* and *DIA1R* gene products play a ubiquitous role in the secretory pathway [39], it is possible that *DIA1*-family homologues play a role in generating these ostariophysan-specific features. Zebrafish are a widely-used model organism for studying vertebrate development in both normal and pathologic conditions [33] and are also being used to study the etiology of neurological disorders including Alzheimer disease [94] and schizophrenia [34], [95]–[97]. Indeed, due to overlapping genes, risk factors and neurological findings, methodology applicable to studies of schizophrenia is also highly relevant to autism [98]–[106]. The ability to manipulate the genome and quantitate effects on behavioural phenotypes, including social skills, makes the zebrafish an attractive model organism to study the etiology of autism [31], [107], [108].

Another unexpected finding was the absence of a *DIA1R* gene from the genomes of acanthopterygian fish, including the ‘completed’ genomes sequence of pufferfishes *T. rubripes* (Fugu) and *T. nigroviridis*, and the medaka *O. latipes.* It would be unusual to have gaps encompassing the same gene in all three species, and unprecedented to also have the same gene unrepresented in the EST databases of all acanthopterygian fish, unless it is not encoded by those species. It is possible *DIA1R* is present and functional, but has diverged beyond recognition in acanthopterygian fish. However, there is a precedent for ‘loss’ of other genes during acanthopterygian fish evolution, and therefore we consider this a more likely phenomenon. For example, loss of Hox-family genes, and melanocortin receptor genes have occurred in pufferfish when compared to zebrafish [109], [110]. Indeed, while 5,918 orthologous genes are found between the medaka and pufferfish, only 1,365 are found between medaka and zebrafish [111].

Given the proposed ubiquitous role of *DIA1R* in secretion, with specific effects on brain function [39], loss of *DIA1R* in acanthopterygian fish would be expected to relate to structural and/or functional differences in acanthopterygian fish compared to fish from other superorders. Indeed, differences in brain structure and function have been reported between species from the superorder Ostariophysi and Acanthopterygii [112]–[116]. Of possible relevance to the etiology of ASD, the fish lacking *DIA1R* are considered solitary in nature, while those with *DIA1R* are considered schooling fish [117].

### Evolution of urochordate *DIA1*

Comparative analyses of the *DIA1* gene product of the urochordate *C. intestinalis* revealed both DIA1- and DIA1R-like characteristics. The unusual phylogenetic placement of *C. intestinalis* DIA1 is not without precedent. It is well-known that the *Ciona* lineage is fast-evolving, making topologies unreliable [118]. Indeed, it has been reported that both the sea urchin and amphioxus genomes are more representative of the ancestral deuterostome than that of *C. intestinalis*, due to its considerable evolutionary changes [42]. In the future, the use of further sequence data from other urochordate species may resolve this issue. The unique characteristics of tunicate *DIA1* and its sequence divergence may relate to traits specific to that lineage. For example, tunicates are the only animals capable of producing cellulose, which is a major component of the characteristic ‘tunic’ which is a defining feature of the subphylum Tunicata [119]. Evolutionary divergence of *DIA1*, and modification of its role within the secretory pathway, may be required for tunic secretion. We are currently investigating the evolution of *C. intestinalis DIA1* in more detail.

### Conservation within the DIA1 protein family

Amino acid sequence alignments revealed a number of amino acids conserved across the DIA1 protein family. Absolutely conserved amino acids found were: two cysteine residues in the amino-terminal region, a centrally-located glycine residue, and a further cysteine residue in the carboxy-terminal region. Conserved cysteine residues have been implicated in the dimerisation of some proteins [120] and can be essential components of metal- or calcium-ion binding sites [121]–[124]. However, none of the known consensus sequences for such motifs are found in DIA1 or DIA1R. We identified 15 unique motifs characteristic of the DIA1-family, and suggest that hydrophobic motif-1 may be a Golgi-retention motif in DIA1 and DIA1R proteins. While the retention of Golgi-resident proteins with TM domains is well-studied [125], retention of fully-luminal Golgi proteins is sparse. However, an amino-terminal leucine-rich region is essential for Golgi retention of the NEFA/NUC family of Ca^2+^-binding EF-hand/leucine zipper proteins [126]. None of the remaining DIA1-family motifs have a predicted function in proteins localized to the lumen of the ER or Golgi apparatus.

Arthropod DIA1 proteins were found to differ from those from other species and form their own phylogenetic clade. DIA1-motif 1 was poorly conserved in arthropod DIA1 proteins, while motifs 4 and 14 were absent in DIA1 from *Drosophila* species. As a result of these differences, most of the *Drosophila* DIA1 proteins did not have significant similarity to that of the divergent *C. intestinalis* DIA1 by BLAST, although there was sufficient similarity to have significant matches to DIA1 proteins from all other species. By contrast, DIA1 from the wasp species *Nasonia vitripennis* was the most similar to non-arthropod DIA1 proteins. This finding was not unexpected, as around 25% of *Nasonia* genes are more similar to human genes than to their *Drosophila* counterparts, reflecting the derived nature of many *Drosophila* genes [127]. Overall, amino acid similarity was greatest in central part of the DIA1 family sequences, compared to the amino- and carboxy-terminal portions, indicating the central part of DIA1 contains the core functional domain of this protein.

### Conclusion

We have demonstrated that *DIA1* and *DIA1R*, but not *DIA1L*, encode signal peptides for targeting to the secretory pathway. Together, with the finding that *DIA1* or *DIA1R* mutations occur in patients with ASD and/or mental retardation [38], [39], a role for DIA1 and DIA1R within the secretory pathway of cells is suggested as causative. While we propose that both *DIA1* and *DIA1R* encode a hydrophobic amino-terminal Golgi-retention motif, in the absence of known functional protein motifs and domains, ongoing studies on human *DIA1* and *DIA1R* are required to determine the exact role(s) of these genes in cellular function, particularly the effects on cognitive function.

## Materials and Methods

### Detection of *DIA1*-family homologues

The human DIA1 amino acid sequence [38], [48] or human DIA1R amino acid sequence [39] were retrieved from the National Center for Biotechnology Information (NCBI) Entrez Protein database, and used in BLAST and keyword searches (search terms: c3orf58 or cXorf36) of the NR protein database at the NCBI [46], [128], [129]. EST and genomic databases at the NCBI were searched using the TBLASTN algorithm [46]. Additional BLAST and keyword searches were carried out on: the Ensembl database, including preliminary genome assemblies [130], [131]; the Joint Genome Institute (JGI) database [132], including the placozoan species *Trichoplax adhaerens* [133]; and SilkDB, the silkworm sequence database [134]. Databases were last searched in August 2009, with the exception of *B. floridae*, where information was updated using assembly data from October 2009.

### Protein alignments and analyses

Protein sequence alignments were generated with CLUSTALW (version 1.8) [47] at NPS@ [135] with manual alignment of some positions. Boxshade (version 3.21), available at EMBnet [136]–[137], was used to format some amino acid alignments. The ExPASy Compute pI/MW tool was used to calculate theoretical molecular weights and isoelectric points [138]. Three trans-membrane prediction methods were used to analyze protein sequences: TMpred [139], TMAP [140], and HMMTOP version 2.0 [141]. Signal peptides were evaluated using SignalP version 3.0 [51], [142] or the SigCleave algorithm [52], which is part of the EMBOSS software suite [143]. Amino acid motifs and domains were investigated using the following resources: MOTIF at GenomeNet [144]; PSORT-II [145]; the Conserved Domain Database at the NCBI, which also contains data from Pfam, SMART and COG [146]; and the ELM resource [147].

### Phylogeny

Gblocks version 0.91b was used to eliminate poorly aligned positions and divergent regions in aligned protein sequences [148], [149]. The evolutionary history of the DIA1 family was inferred using the neighbour-joining method [57] in MEGA4 [150], or using the PhyML maximum-likelihood algorithm [151]–[152] or Bayesian inference [153] via the ‘Phylogeny.fr’ web-server [154]. Statistical reliability of branching was assessed using either bootstrap replicates [155] or approximate likelihood ratio testing [156].

## Supporting Information

Table S1 Taxa, accession numbers, and chromosome location of *DIA1* orthologues.
(0.05 MB PDF)

Table S2 Taxa, accession numbers and chromosome location of partial *DIA1* orthologues.
(0.04 MB PDF)

Table S3 Physical characteristics of all available full-length DIA1 proteins and their similarity to orthologues from key species.
(0.04 MB PDF)

Table S4 Pairwise comparison of DIA1 and DIA1R proteins.
(0.02 MB PDF)

Table S5 Taxa, accession numbers and chromosome location of full-length *DIA1R* orthologues.
(0.03 MB PDF)

Table S6 Taxa, accession numbers and chromosome location of partial *DIA1R* orthologues.
(0.03 MB PDF)

Table S7 Comparison of *Ciona intestinalis* DIA1 to DIA1 and DIA1R from other species.
(0.02 MB PDF)

Table S8 Taxa and accession numbers of full-length *DIA1L* genes.
(0.03 MB PDF)

Table S9 Amino acid comparisons between DIA1L and DIA1 proteins.
(0.02 MB PDF)

Table S10 Taxa, accession numbers and chromosome location of partial *DIA1L* paralogues.
(0.03 MB PDF)

Figure S1 Amino acid sequence comparison of DIA1a and DIA1b from zebrafish. The sequence alignment was generated using CLUSTALW [47]. Identical amino acids are highlighted in red font and indicated below the alignment with an asterisk (*). Strongly similar amino acids are highlighted in green font and indicated below the alignment with a colon (:). Weakly similar amino acids are highlighted in blue font and indicated below the alignment with a full stop (.). Dissimilar amino acids are in black font. Amino acid numbering is provided above the alignment. Gaps are indicated by dashes. The alignment shows 88% identical amino acids, and a further 10% similar amino acids, providing an overall similarity of 98%. Standard single-letter amino acid abbreviations are used. Organism abbreviation uses the first letter of the genus name, followed by the first four letters of the species (i.e., *Danio rerio* DIA1a is abbreviated to DreriDIA1a). Accession numbers can be found in Table S1.
(0.01 MB PDF)

Figure S2 Amino acid sequence comparison of DIA1 proteins. The sequence alignment of all full-length DIA1 proteins was generated using CLUSTALW [47]. A consensus amino acid sequence is presented below the alignment, where uppercase letters indicate absolutely conserved amino acids. Regions of greater than 50% conservation are shaded (identical amino acids are in black boxes and similar amino acids in grey boxes). Amino acid numbering is provided on the left-hand side of the alignment. Gaps required for optimal alignment are indicated by dashes. The alignment reveals that 10 amino acids are absolutely conserved (2% identity), with a further 6% amino acids being similar, providing an overall similarity of 8%. Standard single-letter amino acid abbreviations are used. Organism abbreviation uses the first letter of the genus name, followed by the first four letters of the species (e.g., *Strongylocentrotus purpuratus* DIA1 is abbreviated to SpurpDIA1). Accession numbers and full species names can be found in Table S1.
(0.08 MB PDF)

Figure S3 Amino acid sequence comparison of DIA1R proteins. The sequence alignment was generated using CLUSTALW [47]. Identical amino acids are highlighted in red font and indicated below the alignment with a red asterisk (*). Strongly similar amino acids are highlighted in green font and indicated below the alignment with a green colon (:). Weakly similar amino acids are highlighted in blue font and indicated below the alignment with a blue full stop (.). Dissimilar amino acids are in black font. Amino acid numbering is provided above the alignment. Gaps are indicated by dashes. The alignment shows 124 identical amino acids (27% identity), with a further 27% similar amino acids, providing an overall similarity of 54%. Standard single-letter amino acid abbreviations are used. A consensus line is provided below the alignment where the number “2” is inserted when no consensus amino acid is found. Organism abbreviations use the first letter of the genus name, followed by the first four letters of the species (e.g., *Monodelphis domestica* DIA1R is abbreviated to MdomeDIA1R). Accession numbers and full species names can be found in Table S5.
(0.03 MB PDF)

Figure S4 Amino acid sequence alignment of DIA1 and DIA1R proteins. The sequence alignment of all full-length DIA1 and DIA1R proteins was generated using CLUSTALW [47]. A consensus amino acid sequence is presented below the alignment, where uppercase letters indicate absolutely conserved amino acids. Regions of greater than 50% conservation are shaded (identical amino acids are in black boxes and similar amino acids in grey boxes) and 56% of aligned positions showed conservation in >50% of sequences. Only 8 amino acids are absolutely conserved (∼2% identity), with a further 23 amino acids being similar (∼5%) in all DIA1 and DIA1R proteins. Amino acid numbering is provided on the left-hand side of the alignment. Gaps required for optimal alignment are indicated by dashes. Standard single-letter amino acid abbreviations are used. Organism abbreviations use the first letter of the genus name, followed by the first four letters of the species (e.g., *Gallus gallus* DIA1R is abbreviated to GgallDIA1R). Accession numbers and full species names can be found in Tables S1 and S5.
(0.13 MB PDF)

Figure S5 Signal peptide localization in DIA1 and DIA1R proteins. The sequence alignment and consensus sequence of the amino terminal region of all full-length DIA1 and DIA1R proteins is from Figure S4. Abbreviations are as in Figure S4. DIA1R proteins were grouped together in the top portion of the figure, with aligned DIA1 proteins below, where arthropod sequences are placed above, nonvertebrate/nonarthropod sequences below, and vertebrate DIA1 sequences in the middle (see annotation on right-hand side of alignment). Initiation methiones were not manually aligned. Bold red amino acids represent the last amino acid of the amino-terminal signal peptide predicted using the NN algorithm [51]. Bold blue amino acids represent the last amino acid of the signal peptide predicted using the HMM prediction method [51]. Bold purple amino acids represent the last amino acid of the signal peptide predicted by both NN and HMM prediction methods. Bold underlined amino acids represent the last amino acid of the signal peptide predicted by the Sigcleave algorithm [52]. Lack of an underlined residue indicates no signal peptide cleavage site was predicted within the aligned region by Sigcleave, and lack of a red residue (or purple) indicates no signal peptide cleavage site predicted by the NN algorithm. The most commonly predicted site for cleavage of DIA1R signal peptides or vertebrate and arthropod DIA1 signal peptides are indicated with arrows above the alignment.
(0.03 MB PDF)

Figure S6 Amino acid sequence comparison of DIA1L proteins. A single DIA1L protein from *S. purpuratus* (SpurpDIA1L) and three DIA1L paralogues from *B. floridae* (BflorDIA1La, BflorDIA1Lb, BflorDIA1Lc) were aligned using CLUSTALW [47]. Identical amino acids shared between all four proteins are highlighted in red font and indicated below the alignment with a red asterisk (*). Strongly similar amino acids are highlighted in green font and indicated below the alignment with a green colon (:). Weakly similar amino acids are highlighted in blue font and indicated below the alignment with a blue full stop (.). Dissimilar amino acids are in black font. Amino acid numbering is provided above the alignment. Gaps are indicated by dashes. The alignment shows 7% identical amino acids, with a further 20% similar amino acids, providing an overall similarity of 27%. Amino acid similarity and identity shared at the amino-terminal ends of the three longer DIA1L proteins are indicated using the coloured fonts described above, but the corresponding annotations (*, : and .) are in grey font below the alignment. Standard single-letter amino acid abbreviations are used. Accession numbers can be found in Table S8.
(0.02 MB PDF)

Figure S7 Amino acid sequence comparison of DIA1-family proteins. The sequence alignment of all full-length DIA1, DIA1R and DIA1L proteins was generated using CLUSTALW [47]. Identical amino acids are highlighted in red font and indicated below the alignment with a red asterisk (*). Strongly similar amino acids are highlighted in green font and indicated below the alignment with a green colon (:). Weakly similar amino acids are highlighted in blue font and indicated below the alignment with a blue full stop (.). Dissimilar amino acids are in black font. Amino acid numbering is provided above the alignment. Gaps are indicated by dashes. The alignment shows only 4 identical amino acids and 18 similar amino acids conserved across the entire protein family (3% similarity). Standard single-letter amino acid abbreviations are used. Organism abbreviations use the first letter of the genus name, followed by the first four letters of the species (e.g., *Pteropus vampyrus* DIA1 is abbreviated to PvampDIA1). Accession numbers and full species names can be found in Tables S1, S5 and S8.
(0.05 MB PDF)

Figure S8 Amino acids conserved in at least 80% of DIA1-family members. The sequence alignment of all full-length DIA1, DIA1R and DIA1L proteins was generated using CLUSTALW [47]. A consensus amino acid sequence is presented below the alignment, where uppercase letters indicate absolutely conserved amino acids. Regions of greater than 80% conservation are shaded (identical amino acids are in black boxes and similar amino acids in grey boxes), and 10% of aligned positions showed conservation in >80% of sequences. Amino acid numbering is provided on the left-hand side of the alignment. Gaps required for optimal alignment are indicated by dashes. Standard single-letter amino acid abbreviations are used. Organism abbreviations use the first letter of the genus name, followed by the first four letters of the species (e.g., *Salmo salar* DIA1R is abbreviated to SsalaDIA1R). Accession numbers and full species names can be found in Tables S1, S5 and S8.
(0.10 MB PDF)

Figure S9 Amino acids conserved in at least 50% of DIA1-family members. The sequence alignment of all full-length DIA1, DIA1R and DIA1L proteins was generated using CLUSTALW [47]. A consensus amino acid sequence is presented below the alignment, where uppercase letters indicate absolutely conserved amino acids. Regions of greater than 50% conservation are shaded (identical amino acids are in black boxes and similar amino acids in grey boxes), and 38% of aligned positions showed conservation in >50% of sequences. Amino acid numbering is provided on the left-hand side of the alignment. Gaps required for optimal alignment are indicated by dashes. Standard single letter amino acid abbreviations are used. Organism abbreviations use the first letter of the genus name, followed by the first four letters of the species (e.g., *Tursiops truncatus* DIA1 is abbreviated to TtrunDIA1R). Accession numbers and full species names can be found in Tables S1, S5 and S8.
(0.14 MB PDF)

Figure S10 DIA1-family amino acid sequences in FASTA format. Each DIA1-family amino acid sequence starts with a “>” (greater-than) symbol followed by the species abbreviation and protein type (e.g., *Oryzias latipes* DIA1 is abbreviated to OlatiDIA1 and *Pongo pygmaeus* DIA1R to PpygmDIA1R). Following the initial title line is the actual amino acid sequence, in standard single-letter code. Accession numbers, full species names, and differences to current database sequence data (due to corrections) can be found in Tables S1, S5 and S8.
(0.04 MB PDF)

Figure S11 Maximum-likelihood tree of DIA1-family proteins. Proteins encoded by each full-length *DIA1*-family gene were aligned using CLUSTALW [47] and subjected to maximum-likelihood analysis [151] using PhyML phylogeny software [135]. Approximate likelihood-ratio test for branch-support statistics [156] was carried out, and percentage values are shown next to branches. Branch lengths are proportional to the number of amino acid substitutions per site (see scale bar). G-blocks were used to eliminate poorly aligned positions and divergent regions, since they may not be homologous or may have been saturated by multiple substitutions [148]. The tree was rooted on the cnidarian *N. vectensis* DIA1 sequence (NvectDIA1), as highlighted with an asterisk. Organism abbreviations use the first letter of the genus name, followed by the first four letters of the species. Full species names and accession numbers can be found in Tables S1, S4 and S7.
(0.12 MB PDF)

Figure S12 Phylogeny of the DIA1-family reconstructed using a Bayesian phylogenetic approach. A subset of DIA1-family gene products were aligned using CLUSTALW [47] and subjected to Bayesian inference of phylogeny using the MrBayes programme [153]. The number above each branch refers to the Bayesian posterior probability of the node, given as a percentage (e.g., 77 represents a posterior probability of 0.77). Branch lengths are proportional to the number of amino acid substitutions per site (see scale bar). Gblocks were used to curate the alignment [148]. The tree was rooted on the cnidarian *N. vectensis* DIA1 sequence (NvectDIA1), as highlighted with an asterisk. Organism abbreviations use the first letter of the genus name, followed by the first four letters of the species. Full species names and accession numbers can be found in Tables S1, S4 and S7.
(0.07 MB PDF)
