# Supplementary material for: Characterization of the Deleted in Autism 1 Protein Family: Implications for Studying Cognitive Disorders
Source: PLoS One. 2011 Jan 19;6(1):e14547. doi: 10.1371/journal.pone.0014547 (PMC3023760; doi:10.1371/journal.pone.0014547)
Supplement: Table S1 — Taxa, accession numbers, and chromosome location of DIA1 orthologues. (0.05 MB PDF) [file pone.0014547.s001.pdf]

Table S1. Taxa, accession numbers, and chromosome location of *DIA1* orthologues.

Species[a]	Protein accession number	mRNA (or EST) accession number	Genomic DNA accession number	Chrom[b]	Intron(s)[c]	Comments
**METAZOA**						
**Cnidaria**						
Nematostella vectensis	XP_001637923	XM_001637873	NW_001834388	-	Yes	4 exons
**Echinodermata**						
Strongylocentrotus purpuratus (a)	XP_001190365 (XP_782541)	XM_001190365 (XM_777448)	NW_001465662	-	Yes	Two accession numbers representing alleles same gene (ID 577204) are provided on the NR database. Annotated as incomplete at 5' end of mRNA sequence. However, by similarity to other DIA1 sequences the sequence *is* complete, and amino acid 13 of the current database protein sequence is the predicted initiation methionine. This protein sequence has been used in our subsequent analyses. However, a Kozak consensus for echinoderms is yet to be elucidated, and there are 2 methionines close by that are possible alternatives.
**Arthropoda**						
Aedes aegypti	XP_001660317	XM_001660267	NW_001811157	-	Yes	4 exons
Anopheles gambiae	XP_001689408	XM_001689356	NT_078267	3L	Yes	3 exons
Culex pipiens	XP_001864190	XM_001864155	NW_001887202	-	Yes	3 exons<br>*C. pipiens* also encodes a second *DIA1*-like gene with a single intron (GeneID: 6051368) that may be a non-processed pseudogene (current accession numbers: XP_001867819 and XM_001867784). The proposed pseudogene is truncated at the C-terminus, compared to the parental gene. EST sequences are not identical to either predicted mRNA (around 90% identical to each).
Drosophila ananassae	XP_001960192	XM_001960156	NW_001939294	-	No	
Drosophila erecta	XP_001975165	XM_001975129	NW_001956550	-	No	
Drosophila melanogaster	NP_726172	NM_166516	NT_033778	2R	No	Is a proposed intron in the 5'NCR. Chromosome 2R is also known as Muller

						element C. Protein has predicted ATP binding and ATP hydrolysis motifs.
Drosophila persimilis	XP_002026638	XM_002026602	NW_001985990	-	No	Expected to be located on chromosome 3 (*D. persimilis* Muller element C).
Drosophila pseudoobscura	XP_001362056	XM_001362019	NC_009006	3	No	Incorrectly annotated has having 2 exons, leading the current predicted protein sequence having an extra 12 amino acids compared to DIA1 of all other *Drosophila* species. We found the first exon was not supported by EST sequence. We have used sequence supported by EST sequence and based on comparison to DIA1 from other *Drosophila* DIA1 gene models, and have therefore used amino acid 12 of the currently annotated database protein sequence as the initiation methionine for the genuine protein for all our analyses (Figure S10). This leads to a gene model where a single exon encodes the DIA1 protein.<br>Chromosome 3 corresponds to Muller element C.
Drosophila sechellia	XP_002039872	XM_002039836	NW_001999698	-	No	Expected to be located on chromosome 2R (*D. sechellia* Muller element C).
Drosophila willistoni	XP_002062789	XM_002062753	NW_002032469	-	No	Expected to be located on chromosome 2L (*D. willistoni* Muller element C).
Drosophila yakuba	XP_002092384	XM_002092348	NT_167063	-	No	Expected to be located on chromosome 2R or 2L (*D. yakuba* Muller element C locations).
Nasonia vitripennis	XP_001606587	XM_001606537	NW_001820126	-	Yes	The current database annotation predicts 5 exons, however this is incorrect. By comparison to EST sequences the first annotated exon is wrong, and the start codon is actually found in sequence 5' to the currently annotated exon 2. The remainder of the gene annotation is correct. Our model is supported by EST sequences, and our resulting gene model has 4 exons only. The corrected amino acid sequence (Figure S10) has been used in all analyses.

**Chordata**						
Urochordata						
Ciona intestinalis	XP_002120404	XM_002120368	NW_001954917	-	Yes	8 exons
Cephalochordata						
Branchiostoma floridae	XP_002593078	XM_002593032	NW_003101409	-	Yes	BRAFLDRAFT_72848<br>Gene ID 7229409<br>4 exons
Vertebrata						
Actinopterygii						
Danio rerio (a)	NP_001007437	NM_001007436	NC_007113	2	Yes	3 exons (with another with 5'NCR only)<br>98% similar and 88% identical to *D. rerio* DIA1b (see below) at the amino acid level, 77% identical at the mRNA level, and 42% identical at the genomic level (including intronic sequence, but not 5' or 3' non-coding sequences).
Danio rerio (b)	NP_001108398	NM_001114926	NC_007135	24	Yes	3 exons annotated.<br>98% similar and 88% identical to *D. rerio* DIA1a (see above) at the amino acid level, and 77% identical at the mRNA level, and 42% identical at the genomic level (including intronic sequence, but not 5' or 3' non-coding sequences).
Gasterosteus aculeatus	ENSGACP 00000004897	ENSGACT 00000004911	ENSGACG 00000003735	-	Yes	Incorrect gene model currently on ENSEMBL database with 4 exons (one encoding only 2 amino acids). The model has been corrected to a model with 3 exons, which matches that of other DIA1 genes. In addition, two amino acids have been added to C-terminus of database protein sequence, which then reaches the stop codon of gene, which is not present in the current annotation. The correct sequence can be found in Figure S10.
Oryzias latipes	ENSORLP 00000012699	ENSORLT 00000012700	ENSORLG 00000010126	20	Yes	ENSEMBL identifiers.<br>Two amino acids have been added to C-terminus of database protein sequence, to reach stop codon of gene.<br>The correct sequence can be found in Figure S10.

Takifugu rubripes	ENSTRUP 00000045614	ENSTRUT 00000045768	ENSTRUG 00000017793	-	Yes	ENSEMBL identifiers
Tetraodon nigroviridis	CAG04280	-	CAAE01014737	6	Yes	3 exons
Tetrapoda						
Aves						
Gallus gallus	XP_422591	XM_422591	NW_001471743	9	Yes	3 exons
Amphibia						
Xenopus tropicalis	NP_001120404	NM_001126932	-	-	-	
Mammalia						
Bos taurus	XP_586239	XM_586239	NW_001493763	1	Yes	3 exons
Canis familiaris	XP_854190	XM_849097	NW_876276	23	Yes	3 exons
Homo sapiens	NP_775823	NM_173552	NC_000003	3q24	Yes	Annotated as isoform 'a'. A shorter splice variant also exists, and is currently under further investigation.
Macaca mulatta	XP_001111676	XM_001111676	NW_001112571	2	Yes	3 exons
Monodelphis domestica	XP_001372012	XM_001371975	NW_001581995	7	Yes	3 exons
Mus musculus	NP_001028317	NM_001033145	NC_000075	9 E3.3	Yes	3 exons
Pan troglodytes	XP_516799	XM_516799	NW_001232838	3	Yes	3 exons
Pongo pygmaeus	ENSPPYP 00000015862	ENSPPYT 00000143058	ENSPPYG 00000014182	3	Yes	ENSEMBL identifiers
Pteropus vampyrus	ENSPVAP 00000008197	ENSPVAT 00000008685	ENSPVAG 00000008686	-	Yes	ENSEMBL identifiers
Rattus norvegicus	NP_001127944	NM_001134472	NC_005107	8q31	Yes	Reference database gene model available during this study had 5 exons and correlates to accession numbers XP_236403/XM_236493. This was not supported by EST sequence, had 2 extra exons, and an additional 184 N-terminal amino acids compared with DIA1 from all other mammals. We annotated a corrected gene model (Figure S10) based on the mRNA sequence given in NM_001134472, for which there is EST evidence, and which correlates with the gene model for other mammalian *DIA1* genes.<br>Just prior to submission of this paper the incorrect version was withdrawn from the database.

Tursiops truncatus	ENSTTRP 00000004302	ENSTTRT 00000004566	ENSTTRG 00000004571	-	Yes	ENSEMBL identifiers

[a]Orthologues are only present in Metazoa.
[b]Chromosome location (and map position if available).
[c]Intron(s) disrupting coding sequence (if genomic sequence was available).
