# Supplementary material for: Characterization of the Deleted in Autism 1 Protein Family: Implications for Studying Cognitive Disorders
Source: PLoS One. 2011 Jan 19;6(1):e14547. doi: 10.1371/journal.pone.0014547 (PMC3023760; doi:10.1371/journal.pone.0014547)
Supplement: Table S2 — Taxa, accession numbers and chromosome location of partial DIA1 orthologues. (0.04 MB PDF) [file pone.0014547.s002.pdf]

Table S2. Taxa, accession numbers and chromosome location of partial *DIA1* orthologues.

Species[a]	Protein accession number or identifier	mRNA (or EST) accession number or identifier	Genomic DNA accession number or identifier	Chrom[b]	Intron(s)[c]	Comments
**METAZOA**						
**Platyhelminthes**						
Schistosoma mansoni	-	CD093176 & BU770455	-	-	-	Non-overlapping ESTs.
**Hemichordata**						
Saccoglossus kowalevskii	-	FF496411	-	-	-	EST sequence.
**Mollusca**						
Lottia gigantea	-	FC757266	-	-	-	EST
Mytilus californianus	-	ES736823	-	-	-	EST
Venerupis decussatus	-	AM869986	-	-	-	EST
**Annelida**						
Helobdella robusta	HELRO10585	-	scaffold_18		Yes	JGI identifier.
Capitella sp.	-	EY608635	-	-	-	EST
Hirudo medicinalis	-	EY484180	-	-	-	EST
Tubifex tubifex	-	EY450728	-	-	-	EST
**Arthropoda**						
Apis mellifera	-	-	NW_001254902	-	-	Genomic sequence found by BLAST, correct gene yet to be annotated.
Bombyx mori	BGIBMGA 004346-PA	BGIBMGA 004346-TA	BGIBMGA 004346	-	Yes	Silkworm genome database identifiers.
Drosophila simulans			Chr2R_random: 2756013,2756201	2R	-	*Drosophila* species genome BLAST at http://insects.eugenes.org/species/blast/
Ixodes scapularis	EEC05416	-	DS703671	-	Yes	WGS sequence
Pediculus humanus	EEB19697	-	DS235874	-	Yes	WGS sequence
Triboleum castaneum	XP_001815292	XM_001815240	NW_001092881	-	Yes	Contains gene annotation errors. By similarity to all other *DIA1* genes, the sequence has regions missing.
**Chordata**						
Urochordata						
Ciona savignyi	-	-	reftig_11 Positions 606,676 to 606,885	-	-	Identifiers from the *Ciona savignyi* database at the Broad Institute

Halocynthia roretzi	-	DB634200	-	-	-	EST
Vertebrata						
Cephalaspidomorphi*						
Petromyzon marinus	-	-	Contig84862	-	-	ENSEMBL genomic BLAST<br>No further sequence available near start of contig to try to generate a gene model.
Actinopterygii						
Gadus morhua	-	EX722062	-	-	-	EST
Haplochromis matumbi	-	DB869638	-	-	-	EST
Pimephales promelas (a)	-	DT206727, DT232116 & DT131905	-	-	-	Overlapping 5' EST sequences<br>Homologue of *D. rerio DIA1a* (see data below):<br><br>Comparison of 127 amino acids translation product with *D. rerio* DIA1a gene product-<br>98% similar<br>98% identical<br>Protein BLAST E-value 1e-69<br><br>Comparison of 127 amino acids with *D. rerio* DIA1b gene product:<br>85% similar<br>97% identical<br>Protein BLAST E-value 1e-55
Pimephales promelas (b)	-	DT148813 & DT1248813	-	-	-	Overlapping 5' ESTs<br>Homologue of *D. rerio DIA1b*(see data below):<br><br>Comparison of 190 amino acids translation product with *D. rerio* DIA1b gene product-<br>98% similar<br>100% identical<br>Protein BLAST E-value 9e-109<br><br>Comparison of 190 amino acids with *D. rerio* DIA1a gene product:<br>90% similar<br>99% identical<br>Protein BLAST E-value 2e-100
Tetrapoda						
Aves						
Taeniopygia gutta	-	DV580069	-	-	-	EST
Reptilia						
Anolis carolinensis	-	FG707981	-	-	-	EST

Amphibia						
Xenopus laevis	NP_001089508	NM_001096039	-	-	-	Annotated as a full length protein, but by similarity to other DIA1 genes it is not full length, and lacks sequence encoding the C-terminal ~100 amino acids of DIA1.
Mammalia						
Cavia porcellus	ENSCPOP00000008356	ENSCPOT00000009393	ENSCPOG00000009308	-	Yes	ENSEMBL identifiers
Dasypus novemcinctus	ENSDNOP00000013657	ENSDNOT00000017609	ENSDNOG00000017610	-	Yes	ENSEMBL identifiers
Echinops telfairi						
Equus caballus	ENSECAP00000006978	ENSECAT00000009203	ENSECAG00000009060	-	16	ENSEMBL identifiers
Erinaceus europaeus						
Felis catus	ENSFCAP00000005560	ENSFCAT00000005987	ENSFCAG00000005985	-	Yes	ENSEMBL identifiers
Loxodonta africana	ENSLAFP00000003853	ENSLAFT00000004614	ENSLAFG00000004615	-	Yes	ENSEMBL identifiers
Microcebus murinus	ENSMICP00000011901	ENSMICT00000013056	ENSMICG00000013066	-	Yes	ENSEMBL identifiers<br>Is almost full length, except for what appears to be a single nucleotide deletion in exon 1. This is probably a sequencing error.
Mustela putorius	-	GD183634	-	-	-	EST
Myotis lucifugus	ENSMLUP00000002419	ENSMLUT00000002662	ENSMLUG00000002662	-	Yes	ENSEMBL identifiers
Ochotona princeps	ENSOPRP00000005737	ENSOPRT00000006256	ENSOPRG00000006267	-	Yes	ENSEMBL identifiers
Ornithorhynchus anatinus	ENSOANP00000017787	ENSOANT00000017790	ENSOANG00000011229	-	Yes	ENSEMBL identifiers
Oryctolagus cuniculus	ENSOCUP00000010859	ENSOCUT00000012614	ENSOCUG00000012616	-	Yes	ENSEMBL identifiers
Otolemur garnetti	ENSOGAP00000010287	ENSOGAT00000011491	ENSOGAG00000011489	-	Yes	ENSEMBL identifiers
Papio anubis	-	FC112759	-	-	-	EST
Pongo abelii	-	CR856552	-	-	-	EST
Procavia capensis	ENSPCAP00000010296	ENSPCAT00000011030	ENSPCAG00000011077		Yes	ENSEMBL identifiers.<br>Would be full length except for what appears to be a single nucleotide deletion in exon 1. This is probably a sequencing error.
Spermophilus tridecemlineatus	ENSSTOP00000014040	ENSSTOT00000015685	ENSSTOG00000015680	-	Yes	ENSEMBL identifiers
Sus scrofa	-	DN130313	-	-	-	EST sequence.
Tupaia belangeri	ENSTBEP00000013350	ENSTBET00000015387	ENSTBEG00000015399	-	Yes	ENSEMBL identifiers

[a]Orthologues are only present in the Metazoa.
[b]Chromosome location.
[c]Intron(s) disrupting coding sequence (if genomic sequence was available).
*Classification of extant lamprey as Cephalaspidomorphi (Nelson JS, 1994[¶]) is controversial, and more recently lampreys have been classified in the class Petromyzontida or Hyperoartia [93].
[¶]Nelson JS (1994) *Fishes of the World* (3rd edition). New York: John Wiley and Sons.
