# Supplementary material for: Characterization of the Deleted in Autism 1 Protein Family: Implications for Studying Cognitive Disorders
Source: PLoS One. 2011 Jan 19;6(1):e14547. doi: 10.1371/journal.pone.0014547 (PMC3023760; doi:10.1371/journal.pone.0014547)
Supplement: Table S3 — Physical characteristics of all available full-length DIA1 proteins and their similarity to orthologues from key species. (0.04 MB PDF) [file pone.0014547.s003.pdf]

Table S3. Physical characteristics of all available full-length DIA1 proteins and their similarity to orthologues from key species.

Species[a]	Length (amino acids)	pI[b]	Molecular mass[c] (kDa)	BLASTP[d] similarity to[e]:						
				H. sapiens	*G. gallus*	*D. rerio* (a)	*C. intestinalis*	*S. purpuratus*	*D. melanogaster*	*N. vectensis*
**METAZOA**[f]										
**Cnidaria**										
Nematostella vectensis	402	5.6	44.7	8e-46	4e-44	6e-49	1e-15	6e-47	7e-08	-
**Echinodermata**										
Strongylocentrotus purpuratus	431	7.9	49.7	6e-80	3e-81	5e-84	3e-17	-	2e-08	2e-42
**Arthropoda**										
Hexapoda										
Aedes aegypti	395	6.0	45.7	6e-16	6e-15	2e-14	1e-04	5e-16	6e-14	5e-15
Anopheles gambiae	412	5.4	47.3	2e-06	3e-08	9e-09	0.10	5e-12	4e-10	0.018
Culex pipiens	400	5.1	45.2	2e-13	4e-13	8e-16	5e-04	1e-15	2e-13	2e-18
Drosophila ananassae	347	5.0	40.0	4e-04	3e-04	1e-06	>10	2e-06	1e-134	6e-09
Drosophila erecta	346	5.5	40.0	6e-07	2e-07	3e-08	>10	5e-09	2e-171	5e-10
Drosophila melanogaster	348	5.3	40.0	1e-06	7e-07	2e-08	>10	2e-08	-	5e-08
Drosophila persimilis	351	5.5	40.0	7e-09	2e-08	9e-10	>10	6e-08	3e-135	2e-06
Drosophila pseudoobscura	351	5.4	40.0	1e-07	2e-08	1e-09	>10	6e-08	8e-135	2e-06
Drosophila sechellia	350	5.7	40.3	5e-07	2e-07	7e-09	5.0	1e-08	0.0	5e-07
Drosophila willistoni	345	5.2	40.2	3e-10	3e-07	3e-07	4.6	4e-09	2e-92	1e-09
Drosophila yakuba	345	5.8	40.0	9e-09	3e-09	9e-11	>10	4e-07	3e-175	4e-10
Nasonia vitripennis	404	5.1	46.2	3e-53	7e-53	2e-54	1e-11	9e-47	2e-08	4e-36
**Chordata**										
Urochordata										
Ciona intestinalis	477	9.3	54.5	2e-29	1e-31	3e-34	-	3e-17	>10	4e-14
Cephalochordata										
Branchiostoma floridae	398	5.2	46.1	3e-101	5e-98	9e-104	2e-31	5e-81	7e-12	6e-43
Vertebrata										
Neopterygii										
Danio rerio (a)	432	8.6	50.0	0.0	0.0	-	6e-32	8e-82	2e-07	1e-46
Danio rerio (b)	429	8.7	49.4	0.0	0.0	0.0	6e-31	4e-80	2e-06	3e-44
Gasterosteus aculeatus	434	8.9	50.0	0.0	0.0	0.0	6e-33	5e-81	3e-06	2e-44
Oryzias latipes	434	8.8	50.1	0.0	0.0	0.0	2e-32	3e-83	3e-06	2e-44
Takifugu rubripes	435	8.8	50.1	0.0	0.0	0.0	3e-34	8e-84	1e-07	6e-47
Tetraodon nigroviridis	425	8.7	49.2	0.0	0.0	0.0	6e-32	6e-82	1e-05	9e-45
Tetrapoda										
Aves										
Gallus gallus	429	8.6	49.0	0.0	-	0.0	3e-29	4e-79	7e-06	9e-42
Amphibia										
Xenopus tropicalis	429	8.7	49.3	0.0	0.0	0.0	7e-32	3e-80	1e-06	7e-45
Mammalia										
Bos taurus	430	8.8	49.5	0.0	0.0	0.0	1e-29	6e-80	2e-06	3e-41
Canis familiaris	430	8.8	49.5	0.0	0.0	0.0	1e-29	6e-80	2e-06	3e-41
Homo sapiens	430	8.8	49.4	-	0.0	0.0	1e-29	7e-80	2e-06	3e-41
Macaca mulatta	430	8.8	49.5	-	0.0	0.0	1e-29	7e-80	2e-06	3e-41

Monodelphis domestica	430	8.8	49.3	0.0	0.0	0.0	9e-30	6e-80	8e-07	2e-42
Mus musculus	430	8.9	49.5	0.0	0.0	0.0	2e-30	5e-79	9e-06	8e-41
Pan troglodytes	430	8.8	49.5	0.0	0.0	0.0	1e-29	6e-80	2e-06	3e-41
Pongo pygmaeus	430	8.8	49.5	0.0	0.0	0.0	1e-29	7e-80	2e-06	3e-41
Pteropus vampyrus	430	8.9	49.5	0.0	0.0	0.0	4e-29	7e-80	2e-05	8e-41
Rattus norvegicus	430	8.9	49.5	0.0	0.0	0.0	2e-30	5e-79	9e-06	8e-41
Tursiops truncatus	430	8.8	49.5	0.0	0.0	0.0	1e-29	7e-80	2e-06	3e-41

[a]See Table S1 for accession numbers of full length DIA1 proteins.
[b]Isoelectric point calculated using the assumption that all residues have pKa values equivalent to that of isolated residues, and so may not accurately represent the value for the folded protein.
[c]Isotopically averaged molecular weight prediction in kiloDaltons.
[d]The BLASTP E-value (Expect value) measures the statistical significance threshold for protein sequence matches. The smaller the number, the better the match. Computer shorthand nomenclature is used to present E-values when values are small. For example, 5e-01 = 0.5 and 5e-04 = 0.0005. Values lower than 1e-250 are indicated as zero. When E-values were greater than 10, details are not provided. A dash is used when protein alignments have 100% identity.
[e]Proteins were compared to DIA1 from *Homo sapiens*, *Ciona intestinalis*, *Strongylocentrotus purpuratus*, or *Drosophila melanogaster,* or *Nematostella vectensis* by protein BLAST. The DIA1 proteins used for comparison were chosen as representives from class Mammalia, class Aves, and class Neopterygii within the subphylum Vertebrata, subphylum Urochordata, phylum Echinodermata, phylum Arthropoda, and phylum Cnidaria, respectively.
[f]DIA1 homologues were not found in the phylum Porifera or Nematoda. Partial homologues, including those found in the phylum Mollusca and Annelida (Table S2), are not shown.
