# Supplementary material for: Characterization of the Deleted in Autism 1 Protein Family: Implications for Studying Cognitive Disorders
Source: PLoS One. 2011 Jan 19;6(1):e14547. doi: 10.1371/journal.pone.0014547 (PMC3023760; doi:10.1371/journal.pone.0014547)
Supplement: Table S4 — Pairwise comparison of DIA1 and DIA1R proteins. (0.02 MB PDF) [file pone.0014547.s004.pdf]

Table S4. Pairwise comparison of DIA1 and DIA1R proteins.

Percentage amino acid similarity[c]

Protein		DIA1									DIA1R							
	Species[a]	Hsapi[b]	Mmula	Btaur	Mmusc	Rnorv	Mdome	Ggall	Dr(a)	Dr(b)	Hsapi[b]	Mmula	Btaur	Mmusc	Rnorv	Mdome	Ggall	Dreri
DIA1																		
	Hsapi	-	100	99	98	98	97	90	78	82	28	29	28	26	26	29	28	27
	Mmula	100	-	99	98	98	97	90	78	82	28	29	28	26	26	29	28	27
	Btaur	100	100	-	98	98	97	90	78	82	28	29	28	26	26	29	28	27
	Mmusc	99	99	99	-	100	96	89	78	82	28	29	27	27	27	29	28	27
	Rnorv	99	99	99	100	-	96	89	78	82	28	29	27	27	27	29	28	27
	Mdome	99	99	100	99	99	-	88	77	82	28	28	28	26	27	27	29	27
	Ggall	96	96	96	99	96	96	-	78	82	29	29	28	27	27	28	28	29
	Dreri(a)	96	96	96	97	95	96	95	-	88	28	28	28	26	27	27	26	25
	Dreri(b)	97	97	97	97	97	97	97	98	-	28	28	28	25	26	28	29	26
DIA1R																		
	Hsapi	62	62	62	63	63	62	62	62	62	-	97	83	81	80	74	65	45
	Mmula	63	63	63	64	64	63	63	63	62	99	-	83	81	83	73	64	44
	Btaur	61	61	61	64	64	62	62	63	62	96	94	-	79	78	70	62	46
	Mmusc	63	63	63	64	64	62	64	63	62	94	95	93	-	91	71	63	45
	Rnorv	60	60	60	60	60	62	60	62	62	95	94	94	97	-	70	61	45
	Mdome	63	63	63	63	63	63	63	65	62	91	91	90	90	89	-	66	48
	Ggall	61	61	61	61	61	62	62	63	63	87	87	86	86	86	90	-	45
	Dreri	59	59	59	59	59	60	62	60	60	78	78	77	76	76	78	76	-

Percentage amino acid identity[c]

[a]Species with both a DIA1 *and* DIA1R orthologue (on the current databases) are presented.
[b]Species abbreviations are the first letter of the genus name, followed by the first four letters of the species name e.g. Homo sapiens = Hsapi. Full details of species names and protein accession numbers can be found in from Tables S1 and S5.
[c]The percent identity (above diagonal) and similarity (below diagonal) between proteins aligned pair-wise using CLUSTALW [47] are displayed.
