# Supplementary material for: Characterization of the Deleted in Autism 1 Protein Family: Implications for Studying Cognitive Disorders
Source: PLoS One. 2011 Jan 19;6(1):e14547. doi: 10.1371/journal.pone.0014547 (PMC3023760; doi:10.1371/journal.pone.0014547)
Supplement: Table S5 — Taxa, accession numbers and chromosome location of full-length DIA1R orthologues. (0.03 MB PDF) [file pone.0014547.s005.pdf]

Table S5. Taxa, accession numbers and chromosome location of full-length *DIA1R* orthologues.

Species[a]	Protein accession number	mRNA (or EST) accession number	Genomic DNA accession number or identifier	Chrom[b]	Intron(s)[c]	Comments
**METAZOA**						
**<u>Chordata</u>**						
Vertebrata						
Actinopterygii						
Danio rerio	XP_697696	XM_692604	NW_001879471	9	Yes	5 exons
Salmo salar	NP_001133357	NM_001139885	-	-	-	EST sequence
Tetrapoda						
Aves						
Gallus gallus	XP_416761	XM_416761	NW_001471534	1	Yes	Currently annotated with only 4 exons, but should have 5 exons, by comparison with other vertebrate *DIA1R* genes. This error has resulted in the incorrect 5' mRNA sequence and N-terminal protein sequence being placed on the reference database. Correct sequence is present in genomic sequence (and is not due to a gap in genomic sequence). All subsequent analyses have been done using the corrected sequence (see Figure S10).
Mammalia						
Bos taurus	NP_001030572	NM_001035495	NC_007331	X	Yes	5 exons
Dipodomys ordii	ENSDORP 00000008924	ENSDORT 00000009500	ENSDORG 00000009502	-	Yes	ENSEMBL identifiers
Equus caballus	ENSECAP 00000014058 (XP_001490801)	ENSECAT 00000017314 (XM_001490751)	ENSECAG 00000016492 (NW_001877040)	X	Yes	ENSEMBL identifiers (reference database accession numbers are in brackets, as these differ from the current ENSEMBL model, and are not correct). The current reference database protein has incorrect N-terminal protein sequence. Furthermore, the current reference assembly based on EquCab2, annotates two separate, but adjacent, DIA1R homologues each other on chromosome X. (The second has accession numbers XP_001917806 and XM_001917771.) The presence of two DIA1R homologues is not supported by an ENSEMBL genomic BLAST search (data not shown). Rather these two RefSeq entries and separate genes (LOC100050952 and LOC100147123) encode the N-terminal and C-terminal halves of the correct DIA1R gene product. A corrected gene model was created and a corrected gene product used in all analyses (see Figure S10).

Homo sapiens	NP_789789	NM_176819	NC_000023	Xp11.3	Yes	The accession numbers refer to the full length human DIA1R orthologue.<br>When this paper was being prepared, the eference database had only annotated a shorter splice variant, not the true DIA1R orthologue, which was obtained manually from the genomic sequence. Our non-annotated gene had 5 exons, and has since been placed on the database (annotated as isoform '1'), while the short gene (and only gene originally annotated) has only 3 exons (now referred to as isoform '2').<br>We are currently investigating splice variants of human DIA1R further.
Macaca mulatta	XP_001098371	XM_001098371	NW_001218116	X	Yes	5 exons
Monodelphis domestica	XP_001377879	XM_001377842	NW_001581956	4	Yes	5 exons
Ornithorhynchus anatinus	XP_001512494	XM_001512444	NW_001794129	-	Yes	5 exons
Mus musculus	NP_780437	NM_175228	NC_000086	X A1.3	Yes	5 exons
Rattus norvegicus	XP_576913	XM_576913	NW_048034	Xq12	Yes	5 exons<br>During our analyses, we found that the splice donor, intron phase, and site for intron 1 insertion within the coding sequence, is incorrect. This is by comparison with other mammalian DIA1R genes. This has been caused by gaps in database sequence available in this region (indicated by 'N's in this genomic region). This has lead to a phase 0 intron being annotated, rather than a phase 1, like other mammalian DIA1R genes. This error, however, has only resulted in one erroneous amino acid in the resulting protein on the database, and the corrected protein (see Figure S10) has been included in our analyses. The gene was not further included in our intronic analyses, due to this error.

[a]Orthologues were restricted to the phylum Metazoa.
[b]Chromosome location (and map position if available).
[c]Intron(s) disrupting coding sequence *only* were determined (if genomic sequence was available).
