# Supplementary material for: Characterization of the Deleted in Autism 1 Protein Family: Implications for Studying Cognitive Disorders
Source: PLoS One. 2011 Jan 19;6(1):e14547. doi: 10.1371/journal.pone.0014547 (PMC3023760; doi:10.1371/journal.pone.0014547)
Supplement: Table S6 — Taxa, accession numbers and chromosome location of partial DIA1R orthologues. (0.03 MB PDF) [file pone.0014547.s006.pdf]

Table S6. Taxa, accession numbers and chromosome location of partial *DIA1R* orthologues.

Species[a]	Protein accession number or identifier	mRNA (or EST) accession number or identifier	Genomic DNA accession number or identifier	Chrom[b]	Intron(s)[c]	Comments
**METAZOA**						
**Chordata**						
Vertebrata						
Cephalaspidomorphi						
Petromyzon marinus	-	-	Contig40181	-	Yes	ENSEMBL genomic BLAST
Chondrichthyes						
Leucoraja erinacea	-	EE990465	-	-	-	EST
Squalus acanthias	-	CX663166	-	-	-	EST
Actinopterygii						
Ictalurus punctatus	-	GH685824	-	-	-	EST
Tetrapoda						
Aves						
Taeniopygia guttata	ESTGUP00000006234	ESTGUT00000006296	ESTGUG00000006967	-	Yes	ENSEMBL identifiers<br>First exon appears incorrect on current draft.
Amphibia						
Ambystoma tigrinum	-	CN052543	-	-	-	EST sequence
Mammalia						
Canis familiaris	ENSCAFP00000031380 and ENSCAFP00000021608	ENSCAFT00000036049 and ENSCAFT00000023268	ENSCAFG00000023457 and ENSCAFG00000014656	X	Yes	The first ENSEMBL identifiers match the N-terminal region of DIA1R and the second identifiers match the C-terminal region (both on X chromosome scaffold). Gaps in genomic sequence have led to gene modelling errors.
Cavia porcellus	ENSCPO00000008663	ENSCPOT00000009737	ENSCPOG00000009650	-	Yes	ENSEMBL identifiers
Dasypus novemcinctus	ENSDNOP00000013850	ENSDNOT00000017850	ENSDNOG00000017852	-	Yes	ENSEMBL identifiers
Echinops telfairi	ENSETEP00000015464	ENSETET00000019037	ENSETEG00000019037	-	Yes	ENSEMBL identifiers
Erinaceus europaeus	ENSEEUP00000000288	ENSEEUT00000000324	ENSEEUG00000000323	-	Yes	ENSEMBL identifiers
Felis catus	ENSFCAP00000008763	ENSFCAT00000009451	ENSFCAG00000009449	-	Yes	ENSEMBL identifiers
Loxodonta africana	ENSLAFP00000005064	ENSLAFT00000006035	ENSLAFG00000006035	-	Yes	ENSEMBL identifiers
Felis catus	ENSFCAP00000008763	ENSFCAT00000009451	ENSFCAG00000009449	-	Yes	ENSEMBL identifiers
Microcebus murinus	ENSMICP00000013720	ENSMICT00000015048	ENSMICG00000015058	-	Yes	ENSEMBL identifiers

Myotis lucifugus	ENSMLUP00000001597	ENSMLUT00000001744	ENSMLUG00000001741	-	Yes	ENSEMBL identifiers
Ochotona princeps	ENSOPRP00000008092	ENSOPRT00000008840	ENSOPRG00000008844	-	Yes	ENSEMBL identifiers
Oryctolagus cuniculus	ENSOCUP00000012696	ENSOCUT00000014763	ENSOCUG00000014763	-	Yes	ENSEMBL identifiers
Otelemur garnettii	ENSOGAP00000001890	ENSOGAT00000002117	ENSOGAG00000002115	-	Yes	ENSEMBL identifiers
Pan troglodytes	ENSPTRP00000055446	ENSPTRT00000063901	ENSPTRG00000034190	-		ENSEMBL identifiers<br>On the reference database the equivalent sequence is currently annotated as a pseudogene (Accession number XR_020904).
Pongo pygmaeus	ENSPPYP00000022690	ENSPPYT00000023651	ENSPPYG00000029273	X	Yes	ENSEMBL identifiers
Pteropus vampyrus	ENSPVAP00000004149	ENSPVAT00000004383	ENSPVAG00000004383	-	Yes	ENSEMBL identifiers
Rattus norvegicus	XP_576913	XM_576913	NW_048034	X	Yes	A large gap in the current genomic sequence, means the reference database sequence does not correspond to the expected full length protein/mRNA/gene sequence and is incorrect.
Sorex araneus	ENSSARP00000004306	ENSSART00000004738	ENSSARG0000004729	-	Yes	ENSEMBL identifiers
Spermophilus tridecemlineatus	ENSSTOP00000002079	ENSSTOT00000002324	ENSSTOG00000002326	-	Yes	ENSEMBL identifiers<br>Current mRNA is predicted from 8 exons, rather than the expected 5 exons (as for most vertebrate DIA1R sequences). The most likely scenario is that there are several base mis-calls in the genomic sequence, which has led to prediction of 3 incorrect introns, which currently consist of 1, 2, or 2 bp only. This sequence has therefore not been used in our analyses.
Tarsius syrichta	ENSTSYP00000012353	ENSTSYT00000013464	ENSTSYG00000013470	-	Yes	ENSEMBL identifiers
Tupaia belangeri	ENSTBEP00000005669	ENSTBET00000006583	ENSTBEG00000006593	-	Yes	ENSEMBL identifiers
Tursiops truncatus	ENSTTRP00000006398	ENSTTRT00000006766	ENSTTRG00000006768	-	Yes	ENSEMBL identifiers

[a]Orthologues are only present in the phylum Metazoa.
[b]Chromosome location, if available.
[c]Intron(s) disrupting coding sequence, if genomic sequence was available.
