# Supplementary material for: Characterization of the Deleted in Autism 1 Protein Family: Implications for Studying Cognitive Disorders
Source: PLoS One. 2011 Jan 19;6(1):e14547. doi: 10.1371/journal.pone.0014547 (PMC3023760; doi:10.1371/journal.pone.0014547)
Supplement: Table S7 — Comparison of Ciona intestinalis DIA1 to DIA1 and DIA1R from other species. (0.02 MB PDF) [file pone.0014547.s007.pdf]

Table S7. Comparison of *Ciona intestinalis* DIA1 to DIA1 and DIA1R from other species.

	Protein[a]	DIA1				DIA1R			
	Species[b]	Hsapi	Mmusc	Ggall	Dreri(a)	Hsapi	Mmusc	Ggall	Dreri
BLAST results[c]	BLAST *with* Cinte DIA1	7e-31	7e-32	2e-31	8e-34	4e-28	9e-27	3e-22	1e-23
	BLAST *against* Cinte DIA1	7e-32	9e-33	9e-33	2e-34	3e-29	7e-29	2e-27	4e-24
CLUSTAL results[d]	*Identity* to Cinte DIA1	23%	23%	24%	24%	22%	22%	23%	21%
	Similarity to Cinte DIA1	55%	54%	54%	58%	54%	54%	56%	56%

[a]Comparisons were made between CintDIA1 (the *DIA1* gene product from *C. intestinalis*) and key vertebrate species where both full-length DIA1 and DIA1R sequences were available for comparison. For *D. rerio*, the DIA1a paralogue was used.

[b]Organism abbreviations use the first letter of the genus name, followed by the first four letters of the species (e.g. *Mus musculus* is abbreviated to Mmusc). Accession numbers and full species names can be found in Tables S1 and S5.

[c]E-values were generated by BLASTP and reciprocal BLASTP [46] and measure the statistical significance threshold for protein sequence matches. The smaller the E-value, the better the match, and the more similar the two proteins. Computer shorthand is used for E-values (e.g. 5e-01 = 0.5 and 5e-04 = 0.0005).

[d]Percentage of amino acids identical or similar to *C. intestinalis* DIA1 were determined by pairwise CLUSTALW alignments [47].
