# Supplementary material for: Characterization of the Deleted in Autism 1 Protein Family: Implications for Studying Cognitive Disorders
Source: PLoS One. 2011 Jan 19;6(1):e14547. doi: 10.1371/journal.pone.0014547 (PMC3023760; doi:10.1371/journal.pone.0014547)
Supplement: Table S8 — Taxa and accession numbers of full-length DIA1L genes. (0.03 MB PDF) [file pone.0014547.s008.pdf]

Table S8. Taxa and accession numbers of full-length *DIA1L* genes.

Species	Protein accession number	mRNA (or EST) accession number	Genomic DNA accession number	Intron(s)	Comments
**METAZOA**					
**Echinodermata**					
Strongylocentrotus purpuratus	XP_789827	XM_784734	NW_001348711	Yes	Expression of this gene is supported by EST data. However, our analyses suggest the current predicted splicing of this gene is erroneous. The two main problems with the current annotation are the presence of one very short (10bp) exon, which is followed by an intron using a non-consensus splice donor ('gc' not 'gt'). We therefore analysed the 'problematic' region in detail. This region was found to include duplicated sequence, which currently results in the duplication of a 102 amino acid sequence within the predicted protein sequence. The 'small' exon links these two duplicated regions.<br>The duplicated region is 97% identical at the amino acid level and 96% identical at the nucleotide level (13 nucleotide differences). We have made a new gene annotation that delineates the duplicated region by introns, based on the hypothesis that the duplicated region originated by exon duplication. This annotation creates a phase 1 intron in a position conserved in the *DIA1* gene from *C. intestinalis.* Alignment with the *B. floridae DIA1L* genes, and other DIA1 family proteins improved using the protein sequence predicted from our corrected model of this gene. Confirmation of our model will require further expression data for this gene, which currently does not cover the disputed region. The corrected protein sequence used in all subsequent analyses can be found in Figure S10.

**Chordata**					
Cephalochordata					
Branchiostoma floridae (a)	XP_002604567	XM_002604521	NW_003101509	Yes	BRAFLDRAFT_79443<br>No ESTs currently support expression.<br>A weak similarity of the central portion of this gene product to the uncharacterized human *c18orf51* gene product was noted, and is currently being investigated.
Branchiostoma floridae (b)	XP_002591705	XM_002591751	NW_003101395	Yes	BRAFLDRAFT_123514<br>Several ESTs support gene expression (e.g. GenBank accession number: FE557531). Has partial ATPase domain in C-terminal portion of predicted protein.
Branchiostoma floridae (c)	XP_002591751	XM_002591705	NW_003101395	No	BRAFLDRAFT_83524<br>While all other *B. floridae DIA1* and *DIA1L* genes contain introns (Table S1 and above) this homologous genomic region lacks introns, raising the possibility this sequence should be annotated as a processed pseudogene, not as a hypothetical protein.<br>A BLAST search of the EST database revealed two cDNA sequences with ~97% (Genbank accession number: FE550562) and 93% (Genbank accession number: FE550561) identity to the 5’ or 3’ regions of *DIA1Lc* (at the nucleotide level) respectively. These ESTs provide support for the annotation of *DIA1c* as an intron-less expressed gene, rather than a pseudogene.
