# Supplementary material for: Characterization of the Deleted in Autism 1 Protein Family: Implications for Studying Cognitive Disorders
Source: PLoS One. 2011 Jan 19;6(1):e14547. doi: 10.1371/journal.pone.0014547 (PMC3023760; doi:10.1371/journal.pone.0014547)
Supplement: Table S9 — Amino acid comparisons between DIA1L and DIA1 proteins. (0.02 MB PDF) [file pone.0014547.s009.pdf]

Table S9. Amino acid comparisons between DIA1L and DIA1 proteins.

**Species**		*S. purpuratus*		*B. floridae*			
	**Protein**	DIA1L	DIA1	DIA1La	DIA1Lb	DIA1Lc	DIA1
S. purpuratus	DIA1L	-	15%	18%	21%	16%	17%
	DIA1	42%	-	16%	19%	20%	38%
B. floridae	DIA1La	49%	49%	-	24%	22%	19%
	DIA1Lb	52%	46%	58%	-	17%	19%
	DIA1Lc	62%	54%	54%	54%	-	21%
	DIA1	43%	73%	53%	49%	56%	-

Note: dash indicates 100% amino acid identity, as determined by pair-wise CLUSTALW analyses [47]. Amino acid identity values are above the dashed diagonal 'line'. Amino acid similarity values are below the dashed 'line'.
