# Supplementary material for: Characterization of the Deleted in Autism 1 Protein Family: Implications for Studying Cognitive Disorders
Source: PLoS One. 2011 Jan 19;6(1):e14547. doi: 10.1371/journal.pone.0014547 (PMC3023760; doi:10.1371/journal.pone.0014547)
Supplement: Table S10 — Taxa, accession numbers and chromosome location of partial DIA1L paralogues. (0.03 MB PDF) [file pone.0014547.s010.pdf]

Table S10. Taxa, accession numbers and chromosome location of partial *DIA1L* paralogues.

Species[a]	Protein accession number	mRNA (or EST) accession number	Genomic DNA accession number	Intron(s)[b]	Comments
**METAZOA**					
**Chordata**					
Cephalochordata					
Branchiostoma floridae (1)	XP_002593885	XM_002593839	NW_003101415	-	BRAFLDRAFT_214875<br>Length of predicted protein: 126 amino acids<br>Completeness: incomplete on the carboxy-terminal end. Has an annotated initiation methionine, but this may not be correct.<br>Sequence alignments suggest a close relationship to *B. floridae* DIA1Lb (data not shown).<br>EST data supports expression (93% identical to EST with GenBank accession number: FE571008). However, this is the same EST supporting the gene below (*DIA1L-partial 2)*.
Branchiostoma floridae (2)	XP_002593886	XM_002593840	NW_003101415	-	BRAFLDRAFT_161925<br>Length of predicted protein: 91 amino acids.<br>Completeness: Incomplete on both ends.<br>Sequence alignments suggest a close relationship to DIA1Lb and 'partial 1.'<br>EST data supports expression (93% identical to EST with GenBank accession number: FE571008). However, this is the same EST supporting the gene above.
Branchiostoma floridae (3)	XP_002586953	XM_002586907	NW_003101317	-	BRAFLDRAFT_269049<br>Length of predicted protein: 230 amino acids.<br>Completeness: annotated as 'complete,' but appears to be missing N-terminal (5' coding) sequence.<br>Sequence alignments suggest a close relationship to DIA1Lb and 'partial-4' below.<br>Expression is supported by a single EST sequence (Genbank expression number: FE569795).
Branchiostoma floridae (4)	XP_002613084	XM_002613038	NW_003101564	Yes (but not in DIA1-like region -see adjacent comments)	BRAFLDRAFT_125704<br>Length of predicted protein: 703 amino acids, annotated as 'complete.'<br>Only the first exon has similarity to *DIA1L*. In this gene annotation the first exon of DIA1L (encoding 180 amino acids only) has been 'tacked onto' the 5' end of a gene encoding a *B. floridae Notch* homologue. This most likely represents an incorrect gene annotation.<br>A portion of this sequence is identical to that of *B. floridae DIA1L*-partial 3 (see above), and overlaps the relevant EST sequence (Genbank expression number: FE569795). This suggests these two sequences are incorrectly annotated alleles of the same gene. This is further supported by examination of the surrounding genes, which reveals reverse synteny with the 'partial-3' genomic region (data not shown).<br>Sequence alignments suggest a close relationship of this region to DIA1Lb (data not shown).
Branchiostoma floridae (5)	XP_002588671	XM_002588625	NW_003101353	-	BRAFLDRAFT_255455<br>Length of predicted protein: 115 amino acids.<br>Completeness: Incomplete on both ends.<br>Currently annotated as being expressed, but this is not supported by current EST data.

[a]Homologues of *DIA1* are only present in the Metazoa and *DIA11L* orthologues in they phylum Echinodermata and subphylum Cephalochordata.
[b]A dash is used when an absence of introns was found in sequences, due to insufficient data to draw a definitive conclusion of lack in the full-length gene.
