# Supplementary material for: Characterization of the Deleted in Autism 1 Protein Family: Implications for Studying Cognitive Disorders
Source: PLoS One. 2011 Jan 19;6(1):e14547. doi: 10.1371/journal.pone.0014547 (PMC3023760; doi:10.1371/journal.pone.0014547)
Supplement: Figure S1 — Amino acid sequence comparison of DIA1a and DIA1b from zebrafish. The sequence alignment was generated using CLUSTALW [47]. Identical amino acids are highlighted in red font and indicated below the alignment with an asterisk (*). Strongly similar amino acids are highlighted in green font and indicated below the alignment with a colon (:). Weakly similar amino acids are highlighted in blue font and indicated below the alignment with a full stop (.). Dissimilar amino acids are in black font. Amino acid numbering is provided above the alignment. Gaps are indicated by dashes. The alignment shows 88% identical amino acids, and a further 10% similar amino acids, providing an overall similarity of 98%. Standard single-letter amino acid abbreviations are used. Organism abbreviation uses the first letter of the genus name, followed by the first four letters of the species (i.e., Danio rerio DIA1a is abbreviated to DreriDIA1a). Accession numbers can be found in Table S1. (0.01 MB PDF) [file pone.0014547.s011.pdf]

Fig. S1

```
                    10        20        30        40        50        60
                     |         |         |         |         |         |
DreriDIA1b   MLRFLPLKLGRLYRCLKLLFLLGLFVILLMNTHNLFASFQKNELTDRRFINLNKCPACFG
DreriDIA1a   MLRILSLKFGRVYRCGKFLFIVALFVILLMNTHNLFASFQRNELTDRRFIGLNKCPACFG
             ***:*.**:**:*** *:**::.****************:*********.*********

                    70        80        90       100       110       120
                     |         |         |         |         |         |
DreriDIA1b   TSWCRKFMNGQISFETWGRLRFLDVFNVKNVFFAQYGEPREGTRRIVLKRLGSNQELADI
DreriDIA1a   TSWCRKFMNGQVTFEMWGRLRFLDFFNVKNVFFAQYGEPREGTRRVVLKRLGSNQELAEI
             ***********::** ********.********************:************:*

                   130       140       150       160       170       180
                     |         |         |         |         |         |
DreriDIA1b   DQKICKRATGRPRCDLIQAMYKTEFARLNGDVRLLTPDVVEGWSDLVHCPSQRLLDRIVR
DreriDIA1a   DQKICKRATGRPRCDLIQSMYKTEFARLNGDVRLLTPEVVEGWSDLVHCPSQRLLDRVVR
             ******************:******************:*******************:**

                   190       200       210       220       230       240
                     |         |         |         |         |         |
DreriDIA1b   RYAETKDSGSFLLKNLKDTERMQLLMTLAFNPEPLVLQSFPSDEGWPFAKYLGACGRMVA
DreriDIA1a   RYAETKDSGSFLLKNLKDTERMQLLMTLAFNPEPLVLQSFPSDEGWPFAKYLGACGRMVA
             ************************************************************

                   250       260       270       280       290       300
                     |         |         |         |         |         |
DreriDIA1b   VNYVGEELWSFFNAPWEKRVDLAKQLMDIAEQLTNNDFDFALYLLDVSFDNFAVGPRDGK
DreriDIA1a   VNYVGEELWSFYNAPWEKRVDLAKQLMDIAEQLTNNDFDFALYLLDVSFDNFAVGPRDGK
             ***********:************************************************

                   310       320       330       340       350       360
                     |         |         |         |         |         |
DreriDIA1b   VIVVDAENVVVADKRLIKQNKPESYDVWYESRFEECDKEACLSFSKDMLCSRVTVDHNYY
DreriDIA1a   VIVIDAENVIVVDKRLVKQNKPESYDVWYESRYEECDKEACLSFSKDILCSRVTVDHNYY
             ***:*****:*.****:***************:**************:************

                   370       380       390       400       410       420
                     |         |         |         |         |         |
DreriDIA1b   AICQNLLSRYATWRGSSGGLLHDPPPHIAKDGQLEALLEECANPKKRYGRFQAAKELRDY
DreriDIA1a   AVCQDLLSRFSSWRGSTGGLLHDPPPDVVKDGRLIALLDECTRPQKRYGRFQAAKELREF
             *:**:****:::****:*********.:.***:* ***:**:.*:*************::

                   430
                     |
DreriDIA1b   LTQLSGSAR---
DreriDIA1a   LTQLTQTSNADR
             ****: ::.
```
