# Supplementary material for: Characterization of the Deleted in Autism 1 Protein Family: Implications for Studying Cognitive Disorders
Source: PLoS One. 2011 Jan 19;6(1):e14547. doi: 10.1371/journal.pone.0014547 (PMC3023760; doi:10.1371/journal.pone.0014547)
Supplement: Figure S2 — Amino acid sequence comparison of DIA1 proteins. The sequence alignment of all full-length DIA1 proteins was generated using CLUSTALW [47]. A consensus amino acid sequence is presented below the alignment, where uppercase letters indicate absolutely conserved amino acids. Regions of greater than 50% conservation are shaded (identical amino acids are in black boxes and similar amino acids in grey boxes). Amino acid numbering is provided on the left-hand side of the alignment. Gaps required for optimal alignment are indicated by dashes. The alignment reveals that 10 amino acids are absolutely conserved (2% identity), with a further 6% amino acids being similar, providing an overall similarity of 8%. Standard single-letter amino acid abbreviations are used. Organism abbreviation uses the first letter of the genus name, followed by the first four letters of the species (e.g., Strongylocentrotus purpuratus DIA1 is abbreviated to SpurpDIA1). Accession numbers and full species names can be found in Table S1. (0.08 MB PDF) [file pone.0014547.s012.pdf]

Fig. S2

```
GaculDIA1     1 MLRFLPL--KLGRLYRCLKLLLVVGLFVILLMNTHS-----LFASFQKNELTDRRFINLN
OlatiDIA1     1 MLRFLPL--KLGRLYRCLKLLFLVGLFVILLMNTHN-----LFASFQKNELTDRRFINLN
TnigrDIA1     1 MLRFLPL--KLGRLYRCLKLLLVVGLFVILLMNTHN-----LFASFQKNELTDRRFINLN
TrubrDIA1     1 MLRFLPL--KLGRLYRCLKLLLVVGLFVILLMNTHN-----LFASFQKNELTDRRFINLN
DreriDIA1b    1 MLRFLPL--KLGRLYRCLKLLFLLGLFVILLMNTHN-----LFASFQKNELTDRRFINLN
DreriDIA1a    1 MLRILSL--KFGRVYRCGKFLFIVALFVILLMNTHN-----LFASFQRNELTDRRFIGLN
BtaurDIA1     1 MWRLVPP--KLGRLSRSLKLAALGSLLVLMVLHSPS-----LLASWQRNELADRRFLQLN
PpygmDIA1     1 MWRLVPP--KLGRLSRSLKLAALGSLLVLMVLHSPS-----LLASWQRNELADRRFLQLN
CfamiDIA1     1 MWRLVPP--KLGRLSRSLKLAALGSLLVLMVLHSPS-----LLASWQRNELADRRFLQLN
PtrogDIA1     1 MWRLVPP--KLGRLSRSLKLAALGSLLVLMVLHSPS-----LLASWQRNELADRRFLQLN
TtrunDIA1     1 MWRLVPP--KLGRLSRSLKLAALGSLLVLMVLHSPS-----LLASWQRNELADRRFLQLN
HsapiDIA1     1 MWRLVPP--KLGRLSRSLKLAALGSLLVLMVLHSPS-----LLASWQRNELTDRRFLQLN
MmulaDIA1     1 MWRLVPP--KLGRLSRSLKLAALGSLLVLMVLHSPS-----LLASWQRNELTDRRFLQLN
PvampDIA1     1 MWRLVPP--KLGRLSRSLKLAALGSLLVLMVLHSPS-----LLASWQRNELADRRFLQLN
MmuscDIA1     1 MWRLVPL--KLGRLSRALKLAALGSLLVLMLLHSPS-----LLASWQRNELADRRFLQLN
RnorvDIA1     1 MWRLVPL--KLGRLSRALKLAALGSLLVLMLLHSPS-----LLASWQRNELADRRFLQLN
MdomeDIA1     1 MWRLVPP--KLGRLSRSLKLAALGSLLVLMVLHSPS-----LLASWQRNELADRRFLQLN
GgallDIA1     1 MLRLVSL--KLGRLYRYVKLAVLGSLAAALVLNTHS-----LLASLQRNELAERRFLQLN
XtropDIA1     1 MLRLASL--KFGRLFRYAKVLFAASLLVVMLLNTHS-----LLSSFQRNELTDRRFLSLN
BflorDIA1     1 -MRLVRLR-QFRRFFSGWRVRRMCGFFFFVVFFYCF-----VFQPFSYNQLTDTTFLGED
SpurpDIA1     1 MRRYFNQFTNRFCISTTQQLIWLVVLSSMCLFVYYQ-----LYLHFTANHLENEYFTEAT
NvectDIA1     1 -MHFGGILRKRRYICRLVLLLIVIISFSDLLLWLSYQDGRAIKWDFSSQHKLTFQEIGLN
NvitrDIA1     1 -----------MLVTKASVSSFLIALLILILGIYIN-------R----FNLKVAEITERY
AaegyDIA1     1 ---------MSQ--HRILTKLHLVALFLWIVMKYQPE----ERASSLKSLVQQCQYDTTI
CpipiDIA1     1 ---------MSSGFQRILVNLHILALLFCVIFEFQPK----EPESLTELLVNQCEYETQH
AgambDIA1     1 ---------MQS----S-RLPYFVTLFIAVVLQYLPN----EKSTSKRPLENVCDYDRNI
DyakuDIA1     1 ------------------MHLSPGQLKVVLILALLQE----LQVKPQREVFQELFEKDLR
DerecDIA1     1 ------------------MHLSPGQLKVVLILALLQE----LQVKPQREVFQELYEKDLR
DmelaDIA1     1 ------------------MHLSHGQLKVVLILALLQE----LQVKPQREVFQELFEKDLR
DsechDIA1     1 ------------------MHLSYGQLKVVLILALLQE----LQVKTQREVFQELFEKDLR
DananDIA1     1 ------------------MHLLSGQLRIVLVLALLQD----LQVKPQKDIFQEHFESDLR
DpersDIA1     1 ------------------MHLSPGHLRIVLILALLQD----LQVKPQKDIFRAHLERDLQ
DpseuDIA1     1 ------------------MHLSPGHLRIVLILALLQD----LQVKPQKDIFRAHLERDLQ
DwillDIA1     1 ------------------MHLLYGQLKVVLVLALLQE----LDVRTQKDIFRKHFNMDLL
CinteDIA1     1 MLLKMKRYLTVWENGKYIVFIFILITLEVLLEMYNHRSQGSTRVMLEAKLLSDKWSATSP
consensus     1 m r l    klgrl r lkll vg l villl t       l asfqrnel drrfi ln

GaculDIA1    54 KCP--ACFGT--SWCRKFMNGQVSFET-------WGRL-RFLDVFNVKNVYFAQYGEPRE
OlatiDIA1    54 KCP--ACFGT--SWCRKFMNGQVSFET-------WGRL-RFLDVFNVKNVYFAQYGEPRE
TnigrDIA1    54 KCP--ACFGT--SWCRKFMNGQVSFET-------WGRL-RFLDVFNVKNVYFAQYGEPRE
TrubrDIA1    54 KCP--ACFGT--SWCRKFMNGQVSFET-------WGRL-RFLDVFNVKNVYFAQYGEPRE
DreriDIA1b   54 KCP--ACFGT--SWCRKFMNGQISFET-------WGRL-RFLDVFNVKNVFFAQYGEPRE
DreriDIA1a   54 KCP--ACFGT--SWCRKFMNGQVTFEM-------WGRL-RFLDFFNVKNVFFAQYGEPRE
BtaurDIA1    54 KCP--ACFGT--SWCRRFLNGQVVFEA-------WGRL-RLLDFLNVKNVYFAQYGEPRE
PpygmDIA1    54 KCP--ACFGT--SWCRRFLNGQVVFEA-------WGRL-RLLDFLNVKNVYFAQYGEPRE
CfamiDIA1    54 KCP--ACFGT--SWCRRFLNGQVVFEA-------WGRL-RLLDFLNVKNVYFAQYGEPRE
PtrogDIA1    54 KCP--ACFGT--SWCRRFLNGQVVFEA-------WGRL-RLLDFLNVKNVYFAQYGEPRE
TtrunDIA1    54 KCP--ACFGT--SWCRRFLNGQVVFEA-------WGRL-RLLDFLNVKNVYFAQYGEPRE
HsapiDIA1    54 KCP--ACFGT--SWCRRFLNGQVVFEA-------WGRL-RLLDFLNVKNVYFAQYGEPRE
MmulaDIA1    54 KCP--ACFGT--SWCRRFLNGQVVFEA-------WGRL-RLLDFLNVKNVYFAQYGEPRE
PvampDIA1    54 KCP--ACFGT--SWCRRFLNGQVVFEA-------WGRL-RLLDFLNVKNVYFAQYGEPRE
MmuscDIA1    54 KCP--ACFGT--SWCRRFLNGQVGFET-------WGRL-RLLDFLNVKNVYFAQYGEPRE
RnorvDIA1    54 KCP--ACFGT--SWCRRFLNGQVGFET-------WGRL-RLLDFLNVKNVYFAQYGEPRE
MdomeDIA1    54 KCP--ACFGT--SWCRRFLNGQVAFEA-------WGRL-RLLDFLNVKNVYFGQYGEPRE
GgallDIA1    54 KCP--ACWGT--SWCRKFLNGQLRLES-------WGRL-RLFDFFNVKNVYFARYGEPRE
XtropDIA1    54 KCP--ACFGT--SWCRKFMNGQLSFEG-------WGRL-RLLDFFNVKNVHFAQYGEPRE
BflorDIA1    54 KCP--ACFGT--DLCEEFENGKILFKY-------SSRL-RILDIFNIKNVYFAIY----E
SpurpDIA1    56 KCP--ACFGT--SLCKRFSRGDYRFHS-------YSSI-RLFDYVNVKNVYFATYLD---
NvectDIA1    60 KCP--ACFGINLSICHKLLGGGVTVKS-------N----TPWSEERIKGVSYGLWG----
NvitrDIA1    39 KCP--ACFGD--SMCQVIDSNEISFEY-------TDFYSIFNNLFSVKNVYYGKYK----
AaegyDIA1    46 LCP--GCFPRP-VNCSFFENLKSIDS------------NELFEELLTHFNQHGVRYGTLS
CpipiDIA1    48 LCP--ECFVSPTVNCGHLANFVTIDS------------SNPWNQLTCRYNPHGVSYG-LL
AgambDIA1    43 LCP--ECVSSG-LECNKFDDLFTLQD------------DNVFNIINNIFNSHTTRIGTIV
DyakuDIA1    39 LCP--NCFFGQRELCGEIFQKIAEPSD-------W---SKLLKAISLLVDRRVIYFLRFK
DerecDIA1    39 LCP--DCFVGQSELCVGIFQKIAEPSD-------W---SKLLKAISLIVDRRAIYYLRLK
DmelaDIA1    39 LCP--DCFVGQSEPCGEIFQKIAEPSD-------W---SRLVKAISLLVDRRAIYFLRLK
DsechDIA1    39 LCP--DCFVGQKEQCGEIFQKIAEPSD-------W---SRLVKAISLLVDRRAIHYLRLK
DananDIA1    39 LCP--ACFSAQREQCEEVFRAIREPSD-------L---SKLWKAVTLLFDRREIFWLEIG
DpersDIA1    39 LCP--ACFAGQRWQCEEIFEAIAEPSD-------W---SKLLKGIALLIDRREIYFLQLN
DpseuDIA1    39 LCP--ACFAGQRWQCEEIFEAIAEPSD-------W---SKLLKGIALLIDRREIYFLQLN
DwillDIA1    39 ECP--QCFLGQREQCEKFFDTIEEPAK-------L---DRLWKDLTLLVDPREIYWLELN
CinteDIA1    61 TCGNFSCFGT--SLCKNRNRVGLSEDVPTLRSGWLSIYGELVDLHRVKLNVTYKLPSTLP
consensus    61 kCp  aCfgt  swCrkfmngqv fe       wgrl rlld lnvknvyfaqygepre
```

```
GaculDIA1   102 GTRR-VVLKRLGSNQELAEIDQKICKRATGRPR-CDLIQAMYKTEFA-RINGDVRLLTPE
OlatiDIA1   102 GTRR-VVLKRLGSNQELAEIDQKICKRATGRPR-CDLVQAMYKTEFA-RINGDVRLLTPD
TnigrDIA1   102 GTRR-VVLKRLGSNQELADIDQKICKRATGRPR-CDLIQAMYKTEFA-RINGDVRLLTPD
TrubrDIA1   102 GTRR-VVLKRLGSNQELADIDQKICKRATGRPR-CDLIQAMYKTEFA-RINGDVRLLTPE
DreriDIA1b  102 GTRR-IVLKRLGSNQELADIDQKICKRATGRPR-CDLIQAMYKTEFA-RLNGDVRLLTPD
DreriDIA1a  102 GTRR-VVLKRLGSNQELAEIDQKICKRATGRPR-CDLIQSMYKTEFA-RLNGDVRLLTPE
BtaurDIA1   102 GGRRRVVLKRLGSQRELAQLDQSICKRATGRPR-CDLLQAMPRTEFA-RLNGDVRLLTPE
PpygmDIA1   102 GGRRRVVLKRLGSQRELAQLDQSICKRATGRPR-CDLLQAMPRTEFA-RLNGDVRLLTPE
CfamiDIA1   102 GGRRRVVLKRLGSQRELAQLDQSICKRATGRPR-CDLLQAMPRTEFA-RLNGDVRLLTPE
PtrogDIA1   102 GGRRRVVLKRLGSQRELAQLDQSICKRATGRPR-CDLLQAMPRTEFA-RLNGDVRLLTPE
TtrunDIA1   102 GGRRRVVLKRLGSQRELAQLDQSICKRATGRPR-CDLLQAMPRTEFA-RLNGDVRLLTPE
HsapiDIA1   102 GGRRRVVLKRLGSQRELAQLDQSICKRATGRPR-CDLLQAMPRTEFA-RLNGDVRLLTPE
MmulaDIA1   102 GGRRRVVLKRLGSQRELAQLDQSICKRATGRPR-CDLLQAMPRTEFA-RLNGDVRLLTPE
PvampDIA1   102 GGRRRVVLKRLGSQRELAQLDQSICKRATGRPR-CDLLQAMPRTEFA-RLNGDVRLLTPE
MmuscDIA1   102 GGRRRVVLKRLGSQRELAQLDQSICKRATGRPR-CDLLQAMPRTEFA-RLNGDVRLLTPE
RnorvDIA1   102 GGRRRVVLKRLGSQRELAQLDQSICKRATGRPR-CDLLQAMPRTEFA-RLNGDVRLLTPE
MdomeDIA1   102 GGRRRVVLKRLGSQRELAQLDQSICKRATGRPR-CDLLQAMPKTEFA-RLNGDVRLLTPD
GgallDIA1   102 GSRR-VVLKRLGSAQELADIDAKICRRATGRGR-CDLLQALHATEFA-SLNGDVRLLTPD
XtropDIA1   102 GSRR-VVLKRLGSNHELSELDQRICKKATGRPR-CDLVQAMYKTDFA-RLNGDVRLLTPD
BflorDIA1    98 GMEV--ALKRLGHNSEFDQLDRWICETGKEGPQ-CHIPSVTYYSNFS-RVIYEG--LSPK
SpurpDIA1   101 --QKPVVMKKLGHNSEHNQFDKDLCTNAESGLRGCDVSQQIYKSKLS-DIMYAER-LEEK
NvectDIA1   103 AVKQRVVLKTLGTSSELTAFDNEICRGVGKDSN-CKISQEAWDSDVL-VPKPPLQ-----
NvitrDIA1    84 --DKKVIMKKLAQESELKTFDEMICSDDELHELCYQSESRKQDSKTN-FYKLVESELANV
AaegyDIA1    91 GSEQRVVIKNLNKEHGVEELISSICNDLKIYR-NCRLRNKEPYLR-----VLRQRVL---
CpipiDIA1    93 GSGQKVVIKTLNKNRAVEALRDAVCDELGLTQSNCKFKSDENTLK-----VLRRKVL---
AgambDIA1    88 QENKLAVMKHLNRDNTIEKLLQEYCDAYSLTKQNCRWAQHDGSVDRA-KEFITTSIL---
DyakuDIA1    87 DQDQ-----VVAKRKIIDAQNQQ-IKDVKKAF-----------YE---------------
DerecDIA1    87 DQAQ-----VVAKRKIIDARNHK-AENVEKAF-----------YE---------------
DmelaDIA1    87 DRDQEV-Q-LVAKRKIIDAQSNQ-IKNVKKDF-----------YE---------------
DsechDIA1    87 DQDQAV-Q-LVAKRKIINAHSYQ-MKNVKRAF-----------YE---------------
DananDIA1    87 PSQEKSSQ-LLAKRKSIVASHQS-LSSLQEEF-----------LN---------------
DpersDIA1    87 ERHQAA-Q-LVAKRDAIGGHSYRNMGILREAF-----------LE---------------
DpseuDIA1    87 ERHQAA-Q-LVAKRDAIGGHSYRNMGILGEAF-----------LE---------------
DwillDIA1    87 ENREN-VK-CLAKRKMTRKISKD-------------------------------------
CinteDIA1   119 CNGETCSVQIETSVLSGNNVNKWICDKFKKPTN-CDTGKVFKSWRAMNKITSAKGRLDPN
consensus   121 g rr vvlkrlgsnrela idq ickratgrpr cdliqam ktefa ringdvrlltpe
```

```
GaculDIA1   159 VVEGWSDLVHCPSQRLLDRVVRRYAETKDSGSFLLKNLKDTERMQLLMTLAFNPEPLVLQ
OlatiDIA1   159 VVEGWSDLVHCPSQRLLDRVVRRYAETKDSGSFLLKNLKDTERMQLLMTLAFNPEPLVLQ
TnigrDIA1   159 VVEGWSDLVHCPSQRLLDRVVRRYAETKDSGSFLLKNLKDTERMQLLMTLAFNPEPLVLQ
TrubrDIA1   159 VVEGWSDLVHCPSQRLLDRVVRRYAETKDSGSFLLKNLKDTERMQLLMTLAFNPEPLVLQ
DreriDIA1b  159 VVEGWSDLVHCPSQRLLDRIVRRYAETKDSGSFLLKNLKDTERMQLLMTLAFNPEPLVLQ
DreriDIA1a  159 VVEGWSDLVHCPSQRLLDRVVRRYAETKDSGSFLLKNLKDTERMQLLMTLAFNPEPLVLQ
BtaurDIA1   160 AVEGWSDLVHCPSQRLLDRLVRRYAETKDSGSFLLRNLKDSERMQLLLTLAFNPEPLVLQ
PpygmDIA1   160 AVEGWSDLVHCPSQRLLDRLVRRYAETKDSGSFLLRNLKDSERMQLLLTLAFNPEPLVLQ
CfamiDIA1   160 AVEGWSDLVHCPSQRLLDRLVRRYAETKDSGSFLLRNLKDSERMQLLLTLAFNPEPLVLQ
PtrogDIA1   160 AVEGWSDLVHCPSQRLLDRLVRRYAETKDSGSFLLRNLKDSERMQLLLTLAFNPEPLVLQ
TtrunDIA1   160 AVEGWSDLVHCPSQRLLDRLVRRYAETKDSGSFLLRNLKDSERMQLLLTLAFNPEPLVLQ
HsapiDIA1   160 AVEGWSDLVHCPSQRLLDRLVRRYAETKDSGSFLLRNLKDSERMQLLLTLAFNPEPLVLQ
MmulaDIA1   160 AVEGWSDLVHCPSQRLLDRLVRRYAETKDSGSFLLRNLKDSERMQLLLTLAFNPEPLVLQ
PvampDIA1   160 AVEGWSDLVHCPSQRLLDRLVRRYAETKDSGSFLLRNLKDSERMQLLLTLAFNPEPLVLQ
MmuscDIA1   160 AVEGWSDLVHCPSQRLLDRLVRRYAETKDSGSFLLRNLKDSERMQLLLTLAFNPEPLVLQ
RnorvDIA1   160 AVEGWSDLVHCPSQRLLDRLVRRYAETKDSGSFLLRNLKDSERMQLLLTLAFNPEPLVLQ
MdomeDIA1   160 AVEGWSDLVHCPSQRLLDRLVRRYAETKDSGSFLLRNLKDSERMQLLLTLAFNPEPLVLQ
GgallDIA1   159 AVEGWSDLVHCPSQRLLDRLVRRYAETKDSGSFLLRNLKDSERMQLLITLAFNPEPLVLQ
XtropDIA1   159 VVEGWSDLVHCPSQRLLDRLVRRYAETKDSGSFLLRNLKDTERMQLLLTLAFNPEPLVLQ
BflorDIA1   152 SLGDMSDMVRCPSQRLIDRVLEKFAEHLGKET---EELSYQEKLHFLSTLKFNPEPLMLQ
SpurpDIA1   157 DVMGLSDIVRCPSRRLLDRIWDTFGERQFDKS-----LARDHKLMLATTIAFNPEPLILQ
NvectDIA1   156 --DAVRKYFDCPSTRFLEKIESVYGQETPGQLS------LVETIHLITGLHLNPELLLLQ
NvitrDIA1   141 GGDINNKMRLCPTTTKLEKLFVNVKFQSHAEN-------EDYFKYLWSTIKINPEPLILQ
AaegyDIA1   142 NDNQLEGAILCPLMDE--EALKRFLFEIDEND-----------LNKILLMRINAQPLLLK
CpipiDIA1   145 -EQELEGCIICPSKDE--KALNRLLNEVEGTE-----------LLQLILLKVNVQPLLLE
AgambDIA1   144 DSARVEGCIFCPATNNR-QSLQRFLSLFDSTD---------NELWNLLAVRTNVEPLLIK
DyakuDIA1   115 LGERPGGFHLCRTPSRKP-RFVSYLEQRGHA-----------SASVWFYMMHYVSPLLMQ
DerecDIA1   115 QEEQPGGFHLCRTPSRKPPRFVSYLEQRGHA-----------SASVWFYMMHYVSPLLMQ
DmelaDIA1   118 LEERPGGFHLCRTLAREP-RFVSYLEQRGHA-----------AASVWFYMMYYVSPLLMQ
DsechDIA1   118 LEERPGGFHLCRTLAREP-RFVSYLEQRGHVY---------GSTSVWFYMMHYVSPLLMQ
DananDIA1   119 LEERPGGFHLCRTAQKSS-PFINYLNQRGYD-----------SSSIYFYMLHNISPLLMQ
DpersDIA1   119 MEERPGGFQLCRSGFRTP-RFVSYLEQRGHE-----------AATIWYYMMHSITPLLMQ
DpseuDIA1   119 MEERPGGFQLCRSGFRTP-RFVSYLEQRGHE-----------AATIWYYMMHSITPLLMQ
DwillDIA1   108 LHFNPGGFQMCRNSTR----FLAYLEERGYL-----------PATIGYYIHHSISPLLMQ
CinteDIA1   178 LLKDISPPLQCPSTRIADRIVRRVANKQGGVLLR--DLSYTEKKRLIFTCKMSPGSVVQS
consensus   181  vegwsdlvhCpsqrlldrvvrryaetkdsgsfllknlkdtermqllmtlafnpeplvlq
```

```
GaculDIA1   219 SFP----SDEGWPFAKYLGACGRMVAVN-YVGEELWSFYNAPWEKRVDLARQLMDIAEQL
OlatiDIA1   219 SFP----SDEGWPFAKYLGACGRMVAVN-YVGEELWNFYNAPWEKRVDLARQLMDIAEQL
TnigrDIA1   219 SFP----SDEGWPFAKYLGACGRVVAVN-YVGEELWSFYNAPWEKRVDLARQLMDIAEQL
TrubrDIA1   219 SFP----SDEGWPFAKYLGACGRVVAVN-YVGEELWSFYNAPWEKRVDLARQLMDIAEQL
DreriDIA1b  219 SFP----SDEGWPFAKYLGACGRMVAVN-YVGEELWSFFNAPWEKRVDLAKQLMDIAEQL
DreriDIA1a  219 SFP----SDEGWPFAKYLGACGRMVAVN-YVGEELWSFYNAPWEKRVDLAKQLMDIAEQL
BtaurDIA1   220 SFP----SDEGWPFAKYLGACGRMVAVN-YVGEELWSYFNAPWEKRVDLAWQLMEIAEQL
PpygmDIA1   220 SFP----SDEGWPFAKYLGACGRMVAVN-YVGEELWSYFNAPWEKRVDLAWQLMEIAEQL
CfamiDIA1   220 SFP----SDEGWPFAKYLGACGRMVAVN-YVGEELWSYFNAPWEKRVDLAWQLMEIAEQL
PtrogDIA1   220 SFP----SDEGWPFAKYLGACGRMVAVN-YVGEELWSYFNAPWEKRVDLAWQLMEIAEQL
TtrunDIA1   220 SFP----SDEGWPFAKYLGACGRMVAVN-YVGEELWSYFNAPWEKRVDLAWQLMEIAEQL
HsapiDIA1   220 SFP----SDEGWPFAKYLGACGRMVAVN-YVGEELWSYFNAPWEKRVDLAWQLMEIAEQL
MmulaDIA1   220 SFP----SDEGWPFAKYLGACGRMVAVN-YVGEELWSYFNAPWEKRVDLAWQLMEIAEQL
PvampDIA1   220 SFP----SDEGWPFAKYLGACGRMVAVN-YVGEELWSFFNAPWEKRVDLAWQLMEIAEQL
MmuscDIA1   220 SFP----SDEGWPFAKYLGACGRMVAVN-YVGEELWSYFNAPWEKRVDLAWQLMEIAEQL
RnorvDIA1   220 SFP----SDEGWPFAKYLGACGRMVAVN-YVGEELWSYFNAPWEKRVDLAWQLMEIAEQL
MdomeDIA1   220 SFP----SDEGWPFAKYLGACGRMVAVN-YVGEELWSYFNAPWEKRVDLAWQLMEIAEQL
GgallDIA1   219 SFP----SDEGWPFAKYLGACGRMVAVN-YVGEELWSYFNAPWEKRVDLAWQLMEIAEQL
XtropDIA1   219 SFP----SDEGWPFAKYLGACGRMVAVN-YVGEELWSYFNAPWEKRVDLAWQLMEIAEQL
BflorDIA1   209 TFP----ITEGWPFPFYFGACGRLTVVQ-KCDKTLASYYSAPWLKRVELSLQMMKIAEYL
SpurpDIA1   212 AFP----NRKGWPFPAYMGACGRFTVQE-YSGHSLSYFYKFKFGVRAALAVQALKIAEQL
NvectDIA1   208 IFR----QEEGWPFPRFYGVCGRIMVVE-DSGPPISSFLEESWDVRAQIAVNLITLAHQL
NvitrDIA1   194 ILP----AKEGWPTPKYFGACGRLIVEE-YIGLPLSSFIDEPWIRRAKIASSLLQAADTL
AaegyDIA1   189 LL-----QERNFPVPKLIFQGGFTLVES-YEGEALAKYYDRPLNVRLLIANELIKASLNF
CpipiDIA1   191 LF-----DGRGFPVPKVIFQGGFQLFES-FDGDALVNFYDSSLNIRLRIAKELIQASFLF
AgambDIA1   194 LLTVNYHDEPPLYVPKLLHTFGFSIIET-YEGKTLEHYYDFPFVTRMRIAAELIRAGFKF
DyakuDIA1   163 EL-----YLQGFPVPTTYASCGLTHFQS-YAGRTLTNYFEGEEDLRVELALQLMQLSLKL
DerecDIA1   164 EL-----HLQGFPVPTTYASCGLTHFQA-YAGRTLTNYFEAEEGLRMELALQLIQLSLKL
DmelaDIA1   166 EL-----HLQGFPVPTTYASCGLTHFQS-NAGRTLAHYVDAEEGLRVELALQLIQLSLKL
DsechDIA1   168 EL-----HLQGFPVPTPYASCGLTHFQS-YAGRTLAHYVNAEEGLRLELALQLIQLSLKL
DananDIA1   167 EL-----QNLDFPVPNTYAICGLTHFQA-YAGRTLNHYTEAAEDLRREIARQLIHLSLKL
DpersDIA1   167 EL-----FLRGFPVPRSYAACGLTHFQA-YAGRTLVHYATAGENLRLEMARQLLQLSLKL
DpseuDIA1   167 EL-----FLRGFPVPRSYAACGLTHFQA-YAGRTLVHYATAGENLRLEMARQLLQLSLKL
DwillDIA1   153 EL-----WIMDFPVPRSFAVCGSTLFQA-YAGSTLSNYLKAPIDLRLEMAKQLLQLALKL
CinteDIA1   236 MFP----PYEGWLFQRTVGSCGALMVHGRSIVTSLYELYDAPWKVRLDVAIQLLDLAEHF
consensus   241 sfp    sdegwpfakylgacGrmvavn yvgeelwsyynapwekRvdla qlmdiaeql

GaculDIA1   274 TNNDFDFALYLLDVSFDNFAVGPRDGK----VIVVDAENVLVADKRLIKQNKPESFDVWY
OlatiDIA1   274 TNNDFDFALYLLDVSFDNFAVGPRDGK----VIVVDAENVLVADKRLIKQNKPESYDVWY
TnigrDIA1   274 TNNDFEFALYLLDVSFDNFAVGPRDGK----VIIVDAENVLVADKRLIKQNKPENFDVWY
TrubrDIA1   274 TNNDFEFALYLLDVSFDNFAVGPRDGK----VIIVDAENVLVADKRLIKQNKPENYDVWY
DreriDIA1b  274 TNNDFDFALYLLDVSFDNFAVGPRDGK----VIVVDAENVVVADKRLIKQNKPESYDVWY
DreriDIA1a  274 TNNDFDFALYLLDVSFDNFAVGPRDGK----VIVIDAENVIVVDKRLVKQNKPESYDVWY
BtaurDIA1   275 TNNDFEFALYLLDVSFDNFAVGPRDGK----VIIVDAENVLVADKRLIRQNKPENWDVWY
PpygmDIA1   275 TNNDFEFALYLLDVSFDNFAVGPRDGK----VIIVDAENVLVADKRLIRQNKPENWDVWY
CfamiDIA1   275 TNNDFEFALYLLDVSFDNFAVGPRDGK----VIIVDAENVLVADKRLIRQNKPENWDVWY
PtrogDIA1   275 TNNDFEFALYLLDVSFDNFAVGPRDGK----VIIVDAENVLVADKRLIRQNKPENWDVWY
TtrunDIA1   275 TNNDFEFALYLLDVSFDNFAVGPRDGK----VIIVDAENVLVADKRLIRQNKPENWDVWY
HsapiDIA1   275 TNNDFEFALYLLDVSFDNFAVGPRDGK----VIIVDAENVLVADKRLIRQNKPENWDVWY
MmulaDIA1   275 TNNDFEFALYLLDVSFDNFAVGPRDGK----VIIVDAENVLVADKRLIRQNKPENWDVWY
PvampDIA1   275 TNNDFEFALYLLDVSFDNFAVGPRDGK----VIIVDAENVLVADKRLIRQNKPENWDVWY
MmuscDIA1   275 TNNDFEFALYLLDVSFDNFAVGPRDGK----VIIVDAENVLVADKRLIRQNKPENWDVWY
RnorvDIA1   275 TNNDFEFALYLLDVSFDNFAVGPRDGK----VIIVDAENVLVADKRLIRQNKPENWDVWY
MdomeDIA1   275 TNNDFEFALYLLDVSFDNFAVGPRDGK----VIIVDAENVLVADKKLIRQNKPENWDVWY
GgallDIA1   274 TNNDFEFALYLLDVSFDNFAVGPRDGK----VIIVDAENVLVADKRLIRQNKPENWDVWY
XtropDIA1   274 TNNDFDFALYLLDVSFDNFAVGPRDGK----VIIVDAENVLVADKKLIKQNKPENWDVWY
BflorDIA1   264 TNNEADFGLYLTDISYENFGVTS-DGN----LFVIDVENVIVVDKQKIKADKPRNWEARY
SpurpDIA1   267 SENRDEFALYLTDVSEDNLAVND-NGE----VLVIDAENIIVVDRKKIKEDANPGWDVKH
NvectDIA1   263 SSALDDWALYIADPAPGNFGVSD-SGK----VTLLDLEHLVVVDLSEVQMEEAN-----D
NvitrDIA1   249 MSKNSEFAFYLTDISMDNIAVNN-EDK----AIFVDLENIIIVEKNPPEKALVG-IESWN
AaegyDIA1   243 TAGVDNFRFYLTDISPDNIVVSMSSEE-EIKVTFVDLNNVIILDSHSKRLKP-SKQKHVH
CpipiDIA1   245 TEGVNGFRFYLTDINPDNIAVQAQPSG-SFQVSFIDLDNVIILDSQSKRLDRRSKARNIH
AgambDIA1   253 TEGIHGFRVFLTDINPDNVVVNLKNDNKQVYVSIVDLDNVILLDSWAEVFLT-KNTHHVH
DyakuDIA1   217 TFGFSDFRIMLTDFTGDNFAYDEHTKK----VYLIDLDSVVLVD----ASSATGQAEKYE
DerecDIA1   218 TFGFSDFRIFLTDFTGDNFAYDEDSKR----VYLIDLDSVVLVD----ASSAAGQAEKYV
DmelaDIA1   220 TFGFADFRIILTDFTADNLAYDEDTKK----VYLIDLDSVVLVD----ASFAAGHAEKYE
DsechDIA1   222 TFGFADFRIILTDFTADNLAYDEETKK----VYLIDLDSVVLVD----ASSMAGQAEKYE
DananDIA1   221 TFGFADFRIFLTDFTSDNLTFDEDTRR----VLLIDLDSVVMVD----AASTSGQAEKYE
DpersDIA1   221 TFGFADFRIFLTDFTADNLAFDEASQS----VILIDLDSVVLVD----AAVPLGDAQKYE
DpseuDIA1   221 TFGFADFRIFLTDFTADNLAFDEASQS----VILIDLDSVVLVD----AAVPLGDAQKYE
DwillDIA1   207 SAGFKGFRIYLTDFTADNFAYNEANGT----VLLIDMDTMVLVESNDEAESESQLSKKYK
CinteDIA1   292 TYNDLNYMFALHRLTPRDIGISD-TGK----LRIKNADNVILIDNN--SKNDKGLYEVRY
consensus   301 tnndfdfalylldvsfdnfavgprdgk    vivvdaenvlvadkrlikqnkpenfdvwy
```

```
GaculDIA1   330 ESRFEECD--REACLSFSKESLCSRVTVDHNYYAVCQNLLSRYATWRGTT---GGLLHDP
OlatiDIA1   330 ESRFEECD--REACLSFSKDSLCSRVTVDHNYYAVCQNLLSRYATWRGTT---GGLLHDP
TnigrDIA1   330 ESRFEECD--REACLSFSKDSLCSRVTVDHNYYAVCQNLLSRYATWRGTT---GGLLHDP
TrubrDIA1   330 ESRFEECD--REACLSFSKDSLCSRVTVDHNYYAVCQNLLSRYATWRGTT---GGLLHDP
DreriDIA1b  330 ESRFEECD--KEACLSFSKDMLCSRVTVDHNYYAICQNLLSRYATWRGSS---GGLLHDP
DreriDIA1a  330 ESRYEECD--KEACLSFSKDILCSRVTVDHNYYAVCQDLLSRFSSWRGST---GGLLHDP
BtaurDIA1   331 ESKFDDCD--KEACLSFSKEILCARATVDHNYYAVCQNLLSRHATWRGTS---GGLLHDP
PpygmDIA1   331 ESKFDDCD--KEACLSFSKEILCARATVDHNYYAVCQNLLSRHATWRGTS---GGLLHDP
CfamiDIA1   331 ESKFDDCD--KEACLSFSKEILCARATVDHNYYAVCQNLLSRHATWRGTS---GGLLHDP
PtrogDIA1   331 ESKFDDCD--KEACLSFSKEILCARATVDHNYYAVCQNLLSRHATWRGTS---GGLLHDP
TtrunDIA1   331 ESKFDDCD--KEACLSFSKEILCARATVDHNYYAVCQNLLSRHATWRGTS---GGLLHDP
HsapiDIA1   331 ESKFDDCD--KEACLSFSKEILCARATVDHNYYAVCQNLLSRHATWRGTS---GGLLHDP
MmulaDIA1   331 ESKFDDCD--KEACLSFSKEILCARATVDHNYYAVCQNLLSRHATWRGTS---GGLLHDP
PvampDIA1   331 ESKFDDCD--KEACLSFSKEILCARVTVDHNYYAVCQNLLFRHATWRGTS---GGLLHDP
MmuscDIA1   331 ESKFDDCD--KEACLSFSKEILCARVTVDHNYYAVCQNLLSRHATWRGTS---GGLLHDP
RnorvDIA1   331 ESKFDDCD--KEACLSFSKEILCARVTVDHNYYAVCQNLLSRHATWRGTS---GGLLHDP
MdomeDIA1   331 ESKFDDCD--KEACLSFSKEILCSRATVDHNYYAICQNLLSRHATWRGTS---GGLLHDP
GgallDIA1   330 ESKFDDCD--KEACLSFSKEILCARVTVDHNYYAVCQNLLSRHATWRGTS---GGLLHDP
XtropDIA1   330 ESKFDDCD--KEACLSFSKEILCSRATVDHNYYAICQNLLSRHATWRGTS---GGLLHDP
BflorDIA1   319 QSHFDECPG-MSNCLSFDVSALCRYLHTDHNYYAVCRNMLSEYASEMGKP---GGLLHDP
SpurpDIA1   322 QSEHEGCGS-RRECIMFSHTDLCNHYYSDHNYYAVCQGLFGKDSPHG--D---GGLLHSI
NvectDIA1   313 KPKPSDCS--QPDCLSFSPSDMCSGGSRDHNYYAICQGVLAGSSRRQ-------GLLHNP
NvitrDIA1   303 ETYTNAVDLDCQDCFVFSPNDICSHKVSDHNFYAICQHILTQALGAVFHN---QGFLHDP
AaegyDIA1   301 SRIDCDGC------FAYIQEDICVHQISDINLFAVCQLLLENLNGDSKRG-----FLHSL
CpipiDIA1   304 SRIPCDGC------FAYVQEDLCSYQHSDINQFAICQLLYENLNGDREGG-----FLHIQ
AgambDIA1   312 SKIECNGC------FAYVQEDVCRYQNSDLNLFATCQLLLENLNGHYAKG-----LLHYD
DyakuDIA1   269 P-LPGEG-------FTFDVSAFCSGHQLDANIYQACLLLRDYLLKNLNN----------
DerecDIA1   270 P-LPGEG-------FTFDVSAFCSGHQLDANIYQACLLLRDFLLKNLDN----------
DmelaDIA1   272 P-LTGEG-------FTFDVSAFCSGHQLDANIYQACLLLRDYLLKNLDN----------
DsechDIA1   274 P-LAGEG-------FTFDVSAFCSGHQLDANIYQACLLLRDSLLKNLDN----------
DananDIA1   273 P-LPGEG-------FTFDVSAFCSGRQLDANVYQACLLLRDFLLKDLDN----------
DpersDIA1   273 P-LPGDG-------FTFDVKSFCGGQQLDANVYQACLLLKDFLLRDLAN----------
DpseuDIA1   273 P-LPGDG-------FTFDVKSFCGGQQLDANVYQACLLLKDFLLRDLAN----------
DwillDIA1   263 P-FPGEG-------FTYDVNAFCDNQDLDANIYQVCLLLRDQLLPDVKN----------
CinteDIA1   345 K--QEK--------VDITEENLCDHSENDVNYYVICRHFLGGTWKERKGKKYPGGLLHDP
consensus   361 esrfedcd  reaclsfske lC r tvDhNyyavCqnllsr atwrgtt   gglllhdp

GaculDIA1   385 PAHIAK--DGQLLTLLDECT------RPKKRYGRFQAAKELREYLTQLAAASSSATAR--
OlatiDIA1   385 PAHIAK--DGQLEALLDECT------RPKKRYGRFQAAKELREYLTKLAAASSSATSR--
TnigrDIA1   385 PAHIAK--DGQLEALLDECT------KPKKRYGRFQAAKELREYLTQLA-----------
TrubrDIA1   385 PAHIAK--DGQLEALLDECT------KPKKRFGRFQAAKELREYLTQLAASSSSLVSAR-
DreriDIA1b  385 PPHIAK--DGQLEALLEECA------NPKKRYGRFQAAKELRDYLTQLSGSAR-------
DreriDIA1a  385 PPDVVK--DGRLIALLDECT------RPQKRYGRFQAAKELREFLTQLTQTSNADR----
BtaurDIA1   386 PSEIAK--DGRLEALLDECA------NPKKRYGRFQAAKELREYLAQLSNNVR-------
PpygmDIA1   386 PSEIAK--DGRLEALLDECA------NPKKRYGRFQAAKELREYLAQLSNNVR-------
CfamiDIA1   386 PSEIAK--DGRLEALLDECA------NPKKRYGRFQAAKELREYLAQLSNNVR-------
PtrogDIA1   386 PSEIAK--DGRLEALLDECA------NPKKRYGRFQAAKELREYLAQLSNNVR-------
TtrunDIA1   386 PSEIAK--DGRLEALLDECA------NPKKRYGRFQAAKELREYLAQLSNNVR-------
HsapiDIA1   386 PSEIAK--DGRLEALLDECA------NPKKRYGRFQAAKELREYLAQLSNNVR-------
MmulaDIA1   386 PSEIAK--DGRLEALLDECA------NPKKRYGRFQAAKELREYLAQLSNNVR-------
PvampDIA1   386 PSEIAK--DGRLEALLDECA------NPKKRYGRFHAAKELREYLAQLSNNVR-------
MmuscDIA1   386 PSEIAK--DGRLEALLDECT------NPKKRYGRFQAAKELRGYLAQLSHNVR-------
RnorvDIA1   386 PSEIAK--DGRLEALLDECT------NPKKRYGRFQAAKELRGYLAQLSHNVR-------
MdomeDIA1   386 PGEIAK--DGRLEALLDECA------HPKKRFGRFQAAKELREYLAQLSNNGR-------
GgallDIA1   385 PADIAK--DGRLEALLDECA------NPKKRYGRFQAAKELREYLAQLSNNVR-------
XtropDIA1   385 PAEIAK--DGRLEALLDECA------NPKKRYGRFKSAKELREYLAQLSNNAR-------
BflorDIA1   375 PETVVR--DGTLQRLLAECA------KPRTLYGRFDAAKELIELLGSFLKDR--------
SpurpDIA1   376 PSDLSSYMRAKVKGQIAECR------SPSKRDGRYLVVKELKETLMKLIT----------
NvectDIA1   364 PVALKA----SLNQLLQDCT------AQDMRNTREETAQKLSELLQGYL-----------
NvitrDIA1   360 PDYILQK-HPTLIDLLEQCA------KPDIGYSRIDIAHKLIVLLDSVIKNA--------
AaegyDIA1   350 GTDS---KLEAFRKLLHQCV----YCQPPYCEDRQEVLQRIMEIIHDVLHEVV-------
CpipiDIA1   353 PNDDSQPRLSEIRQLLHHCV----YCVPPDCRDRQGLLQQVQEIIDGILVES--------
AgambDIA1   361 RDAEAFPVLGEASKMLQNLLSECVYCQPPDCQNRSHILKDMLHIIDQTIIQS--------
DyakuDIA1   310 ---------ERLQLLLEQCV----ACQDDFCDMRFQHAYDLIKMLDSKN-----------
DerecDIA1   311 ---------ERLQLLLEQCV----ACQDDFCDMRFQHAYDLIKVLDSKI-----------
DmelaDIA1   313 ---------EKLQLLLEQCV----ACQDDFCDMRFQHAYDLIKVLESNN-----------
DsechDIA1   315 ---------EKLQLLLEQCV----ACQDDFCDMRFQHAYDLIKELESKN-----------
DananDIA1   314 ---------ERLQLLLEKCV----ECQDDLCDMRFQYAYDLIKLLDS-------------
DpersDIA1   314 ---------GRLEMLLEQCV----RCDDATCDMRFQQAYDLIKLLGELLGS---------
DpseuDIA1   314 ---------GRLEMLLEQCV----RCDDATCDMRFQQAYDLIKLLGELLGS---------
DwillDIA1   304 ---------HRLKEMLNDCV----QCEDDKCDIRFKYANNLIDLLVEINNKIEIL-----
CinteDIA1   395 KEFGVFS--SQMFVLLDICSRGKTAVAPERRTLSHSIRRLAANQLRNVLERRRICSPDFK
consensus   421 p  iak  dgrlealldec       npkkrygrfqaakelreyl ql
```

```
GaculDIA1       -------------------------
OlatiDIA1       -------------------------
TnigrDIA1       -------------------------
TrubrDIA1       -------------------------
DreriDIA1b      -------------------------
DreriDIA1a      -------------------------
BtaurDIA1       -------------------------
PpygmDIA1       -------------------------
CfamiDIA1       -------------------------
PtrogDIA1       -------------------------
TtrunDIA1       -------------------------
HsapiDIA1       -------------------------
MmulaDIA1       -------------------------
PvampDIA1       -------------------------
MmuscDIA1       -------------------------
RnorvDIA1       -------------------------
MdomeDIA1       -------------------------
GgallDIA1       -------------------------
XtropDIA1       -------------------------
BflorDIA1       -------------------------
SpurpDIA1       -------------------------
NvectDIA1       -------------------------
NvitrDIA1       -------------------------
AaegyDIA1       -------------------------
CpipiDIA1       -------------------------
AgambDIA1       -------------------------
DyakuDIA1       -------------------------
DerecDIA1       -------------------------
DmelaDIA1       -------------------------
DsechDIA1       -------------------------
DananDIA1       -------------------------
DpersDIA1       -------------------------
DpseuDIA1       -------------------------
DwillDIA1       -------------------------
CinteDIA1   453 YRYPECELAEDAGINLPSIELQWLH
consensus   481
```
