# Supplementary material for: Characterization of the Deleted in Autism 1 Protein Family: Implications for Studying Cognitive Disorders
Source: PLoS One. 2011 Jan 19;6(1):e14547. doi: 10.1371/journal.pone.0014547 (PMC3023760; doi:10.1371/journal.pone.0014547)
Supplement: Figure S3 — Amino acid sequence comparison of DIA1R proteins. The sequence alignment was generated using CLUSTALW [47]. Identical amino acids are highlighted in red font and indicated below the alignment with a red asterisk (*). Strongly similar amino acids are highlighted in green font and indicated below the alignment with a green colon (:). Weakly similar amino acids are highlighted in blue font and indicated below the alignment with a blue full stop (.). Dissimilar amino acids are in black font. Amino acid numbering is provided above the alignment. Gaps are indicated by dashes. The alignment shows 124 identical amino acids (27% identity), with a further 27% similar amino acids, providing an overall similarity of 54%. Standard single-letter amino acid abbreviations are used. A consensus line is provided below the alignment where the number “2” is inserted when no consensus amino acid is found. Organism abbreviations use the first letter of the genus name, followed by the first four letters of the species (e.g., Monodelphis domestica DIA1R is abbreviated to MdomeDIA1R). Accession numbers and full species names can be found in Table S5. (0.03 MB PDF) [file pone.0014547.s013.pdf]

Fig. S3

```
                        10        20        30        40        50        60
                         |         |         |         |         |         |
BtaurDIA1R      MEP-RLGPKAAALHLGWPF-LLLWVSGLSYSVSSPASPSPSP--VSRVRTSYNLGKTFLG
EcabaDIA1R      MEP-WLGPEAAALRPGWPA-LLLWVSALRCSVSSPASPSPSL--VPRVRTSYNFGRTFLG
DordiDIA1R      MDP-QLGPEAAALHPGWQV-LLLLVVSVLSCFSSPASPPPSL--VSRVRTSYNLGRTFLG
MmulaDIA1R      MEP-QLGPEAAALRPGWLA-LLLWVSALSCSFSLPASSPSSL--VSQVRTSYNFGRTFLG
HsapiDIA1R      MEP-QLGPEAAALRPGWLA-LLLWVSALSCSFSLPASSLSSL--VPQVRTSYNFGRTFLG
RnorvDIA1R      MELGRPGAAATAFRQRWPAWMLLWVSTLSCSFSLPASLPPSL--VPRVRSSYTLGKTFLG
MmuscDIA1R      MESQWRGAAATAFHQHWLARLLLWVSTLSCSFSLPASLPPSL--VPRVRSSYTMGKTFLG
MdomeDIA1R      MES-VIHGPLASPCLGWLT--LLQLVTLSCISSTESLTAPSS--VPRVKSSYNFGRTFLG
OanatDIA1R      MGL-RLGCRLFSAALSWMT--LLQFLTRGHGPVADASPAPSL--VPRVKPSYSFGRTFLG
GgallDIA1R      MGR--WICCLCSRVADWLM--LLLVLARSSNPSAAATASPS---APHVRPSYSFGRTFLG
SsalaDIA1R      MPL--KRCDQRAVGSVRLT--VCVLSWLLCVCWAPSVVEPAGTPPAPQQKALILQRAFLG
DreriDIA1R      ----------MAG--------IWSGVWILCFILVFGTADPS---PAPQDKSHDFRKIFLG
                           :         :             .   .:    .    :  : : ***
Consensus       MEP22LGPEAAALRPGWLA2LLLWVS2LSCSFSLPASPPPSLTPVPRVRTSYNFGRTFLG

                        70        80        90       100       110       120
                         |         |         |         |         |         |
BtaurDIA1R      LDKCNACIGTSICKKFFKEEIRFDNGLALHLGPPPDDLPSYSANYSDDFKTWRPVEISRL
EcabaDIA1R      LDKCNACIGTSICKKFFKEEIRFDNRLASHLGLPSGYLPSYSANYSDDSKTWRPVEVSRL
DordiDIA1R      LDKCNACIGTSICKKFFKEEIRFDNSLASDLELASDYLPSYSANYSDDSKTWRPVEIFRL
MmulaDIA1R      LDKCNACIGTSICKKFFKEEIRSDNWLASHLGLPPDFLLSYPANYSDDSKIWRPVEIFRL
HsapiDIA1R      LDKCNACIGTSICKKFFKEEIRSDNWLASHLGLPPDSLLSYPANYSDDSKIWRPVEIFRL
RnorvDIA1R      LDKCNACIGTSICKKFFKEEIRLDNSLVSHLGLPSQDLLSYAANYSDDSKTWRPVEISRL
MmuscDIA1R      LDKCNACIGTSICKKFFKEEIRFDNSLASHLGLPPQDLHSYAANYSDDSKTWRPVEISQL
MdomeDIA1R      LDKCNACIGTSICKKFFKEEIRFDNWLASHLKLPPDYFSTYPANYSDDTKSWRPVEISRL
OanatDIA1R      LDKCNACIGTSICKKFFKEEIRFDNWPASHLKPPPDHFPGYSANYTDDSKSWRLVEISRL
GgallDIA1R      LDKCNACIGTSICKKFFKEEIRFDTWLSSHLKLPPSYLLSYLGNYTDDAQSWRMVDITRL
SsalaDIA1R      LDKCNACVGTSICKKLLKDQIRFDWWMSPDTTLPLAEKQSFPGNLTDDSLSWRPVVLSFL
DreriDIA1R      LDKCNACIGTSICKKFFKDEIRFERRLTAQSNLSSADVRSYEGNYTDSTAGWRPVVVSRL
                *******:*******::*::** :     .   .      : .* :*.   ** * :  *
Consensus       LDKCNACIGTSICKKFFKEEIRFDNWLASHLGLPPD2L2SY2ANYSDDSKTWRPVEISRL

                       130       140       150       160       170       180
                         |         |         |         |         |         |
BtaurDIA1R      VSKQQNKISDGRICASAAAPKTCSIERVLRKTGRFQKWLQAKRLTPDLVRGLSSPLLRCP
EcabaDIA1R      VSKQQNEISDRRICAFAAAPKTCSIERVLRKTRRFQKWLQAKRLTPDLVRGLPSPLLRCP
DordiDIA1R      VSKEQNEISDRRICAFASAPKTCSIERILRKTGRFQKWLQAKRLTPDLVQGLPSPFLRCP
MmulaDIA1R      VSNYQNEISDRRICASASAPRTCSIERVLRKTERFQKWLQAKRLTPDLVQGLASPLLRCP
HsapiDIA1R      VSKYQNEISDRRICASASAPKTCSIERVLRKTERFQKWLQAKRLTPDLVQGLASPLLRCP
RnorvDIA1R      VSKYQSEISDKRICASASGPKTCSIERILRKTGRFQKWLQAKRLTPDLVQGLPSPFLHCP
MmuscDIA1R      VSRYQIEISDRRICASVSAPKTCSIERILQKTGRFQKWLQAKRLTPDLVQGLPSPFLRCP
MdomeDIA1R      ISKYQHELSDRRICASASGSKTCSIERVLRKTERVKKWLKAKRLTPDLVQGLPSPMLRCP
OanatDIA1R      IGKDQNELSDQKICTSVSLVKTCSIERVLRKTERFQKWLKARRLTPALVQGLPSLLLRCP
GgallDIA1R      TTKYQHDRADQHICTSLLKTKTCSLERALRRTHRFQKWLRAKRLTPDLVQGLSSPMLRCP
SsalaDIA1R      SSPCLHSSSDRSICRSVGRQGPCSIEAVLRVTPRFQSLNQSHLLLPHIVKGLAPPLLRCP
DreriDIA1R      MSPHLHQLSDNSICTSAGKGKSCSIEGVLRATSRFQTWVHSNLLLPSMVKGLVTPMLRCP
                      . :*  **       .**:*  *: * *.:.  ::. * * :*:** . :*:**
Consensus       VSKYQNEISDRRICASASAPKTCSIERVLRKT2RFQKWLQAKRLTPDLVQGLPSPLLRCP

                       190       200       210       220       230       240
                         |         |         |         |         |         |
BtaurDIA1R      SQRLLDRVVRRYAEVADAGSIFMDHFTDRDKLRLLYTLAVNTHPVLLQIFPGAEGWPLPQ
EcabaDIA1R      SQRLLDRVVRRYAEVPDAGSIFMDHFTDRDKLRLLYTLAVNAHPILLQIFPGAEGWPLPR
DordiDIA1R      SQRLLDRVVRRYAEVADAGSIFMDHFTDRDKLRLLYTLAVNAHPIFLQIFPGAEGWPLPK
MmulaDIA1R      SQRLLDRVVRRYAEVADAGSIFMDHFTDRDKLRLLYTLAVNSHPILLQIFPGAEGWPLPQ
HsapiDIA1R      SQRLLDRVVRRYAEVADAGSIFMDHFTDRDKLRLLYTLAVNSHPILLQIFPGAEGWPLPK
RnorvDIA1R      SQRLLDRVVRRYAEVVDAGSIFMDHFTDRDKLRLLYTLAVNAHPIILQIFPGAEGWPMPK
MmuscDIA1R      SQRLLDRVVRRYAEVVDAGSIFMDHFTAGDKLRLLYTLAVNAHPIILQIFPGAEGWPMPR
MdomeDIA1R      SQRLLDRIVRRYAEVADAGSVFMDHFTDRDKLRLLYTLSINAHPIMLQIFPGAEGWPLPR
OanatDIA1R      TQRLLDRIVRRYAEVPDAGSVYMDHFTDRDKLRLLYTLSVNAHPVLLQVFPGAEGWPFPK
GgallDIA1R      SQRLLDRIVRRYAEVPDAGSIYMDHLTDQDKLRLLYTLSVNSHPILLQIFPDVEGWPFPR
SsalaDIA1R      SQRLLDRIVRRYAEVVDVGSVQMKHFSERDKLRLLYTLAVNQQPLILQMFPGTEGWPFPR
DreriDIA1R      SQRLLDRIVRRYFEVTDVGSVQMKHFNEKDKLRLLYTLAVNQQPLILQMFPGTEGWPFLR
                :******:**** ** *.**: *.*:.  *********::* :*::**:**..****: :
Consensus       SQRLLDRVVRRYAEVADAGSIFMDHFTDRDKLRLLYTLAVNAHPILLQIFPGAEGWPLPR
```

```
                      250       260       270       280       290       300
                        |         |         |         |         |         |
BtaurDIA1R    YLGSCGRFLVSTSTSPLQEFYGAP--PDQAADLAYQLLGVLESLRSNDLNYFFYFTHVDA
EcabaDIA1R    YLGSCGRFLISTSTSPLQEFYSAP--PDQAADLAYQLLGVLESLRSNDLNYFFYFTHVDA
DordiDIA1R    YLGSCGRFFVSTSTRPLQEFYGAP--PDEAADLAYQLLGVLESLRSNDLNYFFYFTHVDA
MmulaDIA1R    YLGSCGRFLVSTSTRPLQEFYGAP--PDQAADLAYQLLGVLESLRSNDLNYFFYFTHIDA
HsapiDIA1R    YLGSCGRFLVSTSTRPLQEFYDAP--PDQAADLAYQLLGVLESLRSNDLNYFFYFTHIDA
RnorvDIA1R    YLGSCGRFLVSTSTRPLQEFYDAP--PEQAADIAYQLLGVLESLRSNDLNYFFYFTHVDA
MmuscDIA1R    YLGSCGRFLVSTSTRPLQEFYDAS--PEQAADLAYQLLRVLESLRSNDLNYFFYFTHVDA
MdomeDIA1R    YLGSCGRFLVSTSTQPLEEFYSSP--ADQAADLAYQLLGILDSLRNNDLNYFFYFTRIDA
OanatDIA1R    YLGSCGRLLVSTSTTPLLELSSGP--PDRAADLGHQLLGVIEFLRHNDWNYFFYFTRIHA
GgallDIA1R    YLGSCGRLVVSASTRPLRDFFRAA--PEVAADLALQLLAVLHSMGTNDLNYFFYFTRVDV
SsalaDIA1R    YQGSCGRLMVWASSRPLWGLYGSSREFVQRVDVAYQLLHITQGLGHNSLGFLLYYTRLGE
DreriDIA1R    YHGSCGRLMVWAASRALRTLFSSP--LERRADLAYQLLHITQSLSANSLRFRLFYTRIPE
              * *****:.: ::: .*  :  ..      .*:. *** : . :  *.  : :::*::
Consensus     YLGSCGRFLVSTSTRPLQEFY2APREPDQAADLAYQLLGVLESLRSNDLNYFFYFTHVDA

                      310       320       330       340       350       360
                        |         |         |         |         |         |
BtaurDIA1R    DMFGIFNNGHLFIRDASALGVIDRQEGSQAAS--GAGDNKDIFSCL-VSGCQTK-LPSCD
EcabaDIA1R    GMFGIFNNGHLFIRDASTLGVIDRQEGSQAAA--RAGENKDIFSCL-LSDCQAE-LPSCD
DordiDIA1R    AMFGIFSNGHLFIRDASALGVIDKQEGSQAAP--KAGENQDIFSCL-VSGCQAQLLPSCD
MmulaDIA1R    GMFGVFNNGHLFIRDASAVGVIDKQEGSQEAT--RAGENKDIFSCL-VSGCQAQ-LPSCE
HsapiDIA1R    GMFGVFNNGHLFIRDASAVGVIDKQEGSQEAN--RAGENKDIFSCL-VSGCQAQ-LPSCE
RnorvDIA1R    GMFGIFDNGHLFIRDASALGIIDKQEASQAAD--RTGENEDIFSCL-VSGCQVQ-MSSCD
MmuscDIA1R    GMFGIFDNGHLFIRDASALGIIDKQEGSQAAA--RTGENEDIFSCL-VSDCQIQ-LSSCD
MdomeDIA1R    GTFGIFDNGHLFIRDASTVGVIDKQRGSLTLD--RQEESKDIFSCL-VLDCQSP-FPSCS
OanatDIA1R    GTFGVFDNGHLFIRDASSLGVIDRQEGKRPAR--SSPEPRDIFSCL-AADCQSD-LPSCN
GgallDIA1R    GTFGVFSNGHLFIRDASTLGIIDKEEGSQPID--GQQEYKDIFSCL-TVDCQSA-FVSCN
SsalaDIA1R    DMFGLLDDQRVFITDASSIGVIDLEQGFPPDPPSQTGSDGDIFSCLGQGTSPCHRSPPCS
DreriDIA1R    DMFGILEDNKVFIVDTSTIGIIDLQEGHPPDK-DLLPEELDVFSCL-SG--SCVRPPPCE
                **::.: ::** *:*::*:** :..          .  *:****           .*.
Consensus     GMFGIFDNGHLFIRDASALGVIDKQEGSQAADP2RAGENKDIFSCLGVS2CQAQRLPSCD

                      370       380       390       400       410       420
                        |         |         |         |         |         |
BtaurDIA1R    TIPEKQNLVLVCSQVLP-LLLQAK-------------FPSPVQEEIDAELTRCADGTRPD
EcabaDIA1R    TVPEKQSLVLVCRQVLP-RLLQGK-------------FPSPVQEEIDATLTWCGEDTRPD
DordiDIA1R    NIPDKQSVVLVCQQLLP-QLLQGK-------------FPSPVQVEIDSALAQCGQGIRPD
MmulaDIA1R    SVSEKQSLVLVCQKLLP-RLLQGR-------------FPSPVQDDIDSALAQCGDSTRPD
HsapiDIA1R    SISEKQSLVLVCQKLLP-RLLQGR-------------FPSPVQDDIDSILVQCGDSIRPD
RnorvDIA1R    TVPEKQNLVLVCQQLLP-QLLRGK-------------FPSPVQQEIDSALSLCSKDASTN
MmuscDIA1R    TVPEKQSLVLVCQQLLP-QLLQGK-------------FPSPVQKEIDSALSLCSKDNSTD
MdomeDIA1R    SVKEKQSWVMACQQLLP-QLLKEK-------------FPQPIQEQIDSALVLCGNSSLSD
OanatDIA1R    TVQEHQSLVLVCRELLP-KLLEGK-------------FPKPVQEEIDALLDRCGDSSLDG
GgallDIA1R    SIREKQSLVMVCQELLP-KLLRGK-------------FLPPVQEKIDSFLQHCAEGLADD
SsalaDIA1R    SVRPTQSLTLLCTALLPRLLLTERGAQTTRLPMEGEAEAGRLARDVPLLLGVCADPSQPD
DreriDIA1R    TVREAQSFILLCKYILN-NLLTSN-----------DKQSG-LPRAAVDELLVCADPSQLD
              .:   *.  : *  :*   **  .                 :       *  *..    .
Consensus     TVPEKQSLVLVCQQLLPRQLLQGKGAQTTRLPMEG22FPSPVQEEIDSAL42CGDSSRPD

                      430       440       450
                        |         |         |
BtaurDIA1R    PEVLGAASRLKDILRPLRTCDPRFAYRYPDCKYDDKF
EcabaDIA1R    AEVLGAASRLKDILRPLRTCDPRFAYRYPDCKYNDKF
DordiDIA1R    SEVFGAASRLKDILRPLRTCDPRFAYRYPDCKYNDRF
MmulaDIA1R    PEVLGAASWLKHILRPLRTCDSRFAYRYPDCKYNDKF
HsapiDIA1R    PEVLGAASQLKDILRPLRTCDSRFAYRYPDCKYNDKF
RnorvDIA1R    LEVFGATSLLKNILRSLRTCDPRFAYRYPDCKYNDRF
MmuscDIA1R    LEVLGATSWLKDILRSLRTCDPRFAYRYPDCKYNDRF
MdomeDIA1R    QEVIEAASRLKAILKPLRTCDPRYAYRYPDCKYSDRY
OanatDIA1R    QTVIGGARRLMDILKSLRTCDARFAYRYPDCKYSEKY
GgallDIA1R    QDVNEAMAKLAQLLKPLRSCDSRFAYRYPDCKYSDKY
SsalaDIA1R    WRIMAAVGSLMDLLKPMRPCNPHYTYRYPECRYNQDY
DreriDIA1R    QTIIKSVQSLKNILKTLRPCSPQYAYRYPECLYSDKF
                :  .   *  :*:.:*.*..:::****:* *.: :
Consensus     QEVLGAASRLKDILRPLRTCDPRFAYRYPDCKYNDKF
```
