# Supplementary material for: Characterization of the Deleted in Autism 1 Protein Family: Implications for Studying Cognitive Disorders
Source: PLoS One. 2011 Jan 19;6(1):e14547. doi: 10.1371/journal.pone.0014547 (PMC3023760; doi:10.1371/journal.pone.0014547)
Supplement: Figure S4 — Amino acid sequence alignment of DIA1 and DIA1R proteins. The sequence alignment of all full-length DIA1 and DIA1R proteins was generated using CLUSTALW [47]. A consensus amino acid sequence is presented below the alignment, where uppercase letters indicate absolutely conserved amino acids. Regions of greater than 50% conservation are shaded (identical amino acids are in black boxes and similar amino acids in grey boxes) and 56% of aligned positions showed conservation in >50% of sequences. Only 8 amino acids are absolutely conserved (∼2% identity), with a further 23 amino acids being similar (∼5%) in all DIA1 and DIA1R proteins. Amino acid numbering is provided on the left-hand side of the alignment. Gaps required for optimal alignment are indicated by dashes. Standard single-letter amino acid abbreviations are used. Organism abbreviations use the first letter of the genus name, followed by the first four letters of the species (e.g., Gallus gallus DIA1R is abbreviated to GgallDIA1R). Accession numbers and full species names can be found in Tables S1 and S5. (0.13 MB PDF) [file pone.0014547.s014.pdf]

Fig. S4

```
AaegyDIA1     1 --------------MSQ--HRILTKLHLVALFLWIVMKYQPEERASSLKSLVQQCQYDTT
CpipiDIA1     1 --------------MSSGFQRILVNLHILALLFCVIFEFQPKEPESLTELLVNQCEYETQ
AgambDIA1     1 --------------MQS----S-RLPYFVTLFIAVVLQYLPNEKSTSKRPLENVCDYDRN
DyakuDIA1     1 -----------------------MHLSPGQLKVVLILALLQELQVKPQREVFQELFEKDL
DerecDIA1     1 -----------------------MHLSPGQLKVVLILALLQELQVKPQREVFQELYEKDL
DmelaDIA1     1 -----------------------MHLSHGQLKVVLILALLQELQVKPQREVFQELFEKDL
DsechDIA1     1 -----------------------MHLSYGQLKVVLILALLQELQVKTQREVFQELFEKDL
DananDIA1     1 -----------------------MHLLSGQLRIVLVLALLQDLQVKPQKDIFQEHFESDL
DpersDIA1     1 -----------------------MHLSPGHLRIVLILALLQDLQVKPQKDIFRAHLERDL
DpseuDIA1     1 -----------------------MHLSPGHLRIVLILALLQDLQVKPQKDIFRAHLERDL
DwillDIA1     1 -----------------------MHLLYGQLKVVLVLALLQELDVRTQKDIFRKHFNMDL
GaculDIA1     1 ------MLRFLPLKLGR--LYRCLKLLLVVGLFVILLMNTHSLFASFQKNELTDRRFINL
OlatiDIA1     1 ------MLRFLPLKLGR--LYRCLKLLFLVGLFVILLMNTHNLFASFQKNELTDRRFINL
TnigrDIA1     1 ------MLRFLPLKLGR--LYRCLKLLLVVGLFVILLMNTHNLFASFQKNELTDRRFINL
TrubrDIA1     1 ------MLRFLPLKLGR--LYRCLKLLLVVGLFVILLMNTHNLFASFQKNELTDRRFINL
DreriDIA1b    1 ------MLRFLPLKLGR--LYRCLKLLFLLGLFVILLMNTHNLFASFQKNELTDRRFINL
DreriDIA1a    1 ------MLRILSLKFGR--VYRCGKFLFIVALFVILLMNTHNLFASFQRNELTDRRFIGL
BtaurDIA1     1 ------MWRLVPPKLGR--LSRSLKLAALGSLLVLMVLHSPSLLASWQRNELADRRFLQL
PpygmDIA1     1 ------MWRLVPPKLGR--LSRSLKLAALGSLLVLMVLHSPSLLASWQRNELADRRFLQL
CfamiDIA1     1 ------MWRLVPPKLGR--LSRSLKLAALGSLLVLMVLHSPSLLASWQRNELADRRFLQL
TtrunDIA1     1 ------MWRLVPPKLGR--LSRSLKLAALGSLLVLMVLHSPSLLASWQRNELADRRFLQL
PtrogDIA1     1 ------MWRLVPPKLGR--LSRSLKLAALGSLLVLMVLHSPSLLASWQRNELADRRFLQL
HsapiDIA1     1 ------MWRLVPPKLGR--LSRSLKLAALGSLLVLMVLHSPSLLASWQRNELTDRRFLQL
MmulaDIA1     1 ------MWRLVPPKLGR--LSRSLKLAALGSLLVLMVLHSPSLLASWQRNELTDRRFLQL
PvampDIA1     1 ------MWRLVPPKLGR--LSRSLKLAALGSLLVLMVLHSPSLLASWQRNELADRRFLQL
MmuscDIA1     1 ------MWRLVPLKLGR--LSRALKLAALGSLLVLMLLHSPSLLASWQRNELADRRFLQL
RnorvDIA1     1 ------MWRLVPLKLGR--LSRALKLAALGSLLVLMLLHSPSLLASWQRNELADRRFLQL
MdomeDIA1     1 ------MWRLVPPKLGR--LSRSLKLAALGSLLVLMVLHSPSLLASWQRNELADRRFLQL
GgallDIA1     1 ------MLRLVSLKLGR--LYRYVKLAVLGSLAAALVLNTHSLLASLQRNELAERRFLQL
XtropDIA1     1 ------MLRLASLKFGR--LFRYAKVLFAASLLVVMLLNTHSLLSSFQRNELTDRRFLSL
SpurpDIA1     1 ------MRRYFNQFTNRFCISTTQQLIWLVVLSSMCLFVYYQLYLHFTANHLENEYFTEA
RnorvDIA1R    1 MELGRPGAAATAFRQRWP-AWMLLWVSTLSCSFSLPASLPPSLVPRVRSSYTLGKTFLGL
MmuscDIA1R    1 MESQWRGAAATAFHQHWL-ARLLLWVSTLSCSFSLPASLPPSLVPRVRSSYTMGKTFLGL
MmulaDIA1R    1 MEP-QLGPEAAALRPGWL-A-LLLWVSALSCSFSLPASSPSSLVSQVRTSYNFGRTFLGL
HsapiDIA1R    1 MEP-QLGPEAAALRPGWL-A-LLLWVSALSCSFSLPASSLSSLVPQVRTSYNFGRTFLGL
BtaurDIA1R    1 MEP-RLGPKAAALHLGWP-F-LLLWVSGLSYSVSSPASPSPSPVSRVRTSYNLGKTFLGL
EcabaDIA1R    1 MEP-WLGPEAAALRPGWP-A-LLLWVSALRCSVSSPASPSPSLVPRVRTSYNFGRTFLGL
DordiDIA1R    1 MDP-QLGPEAAALHPGWQ-V-LLLLVVSVLSCFSSPASPPPSLVSRVRTSYNLGRTFLGL
MdomeDIA1R    1 MES-VIHGPLASPCLGWL-T--LLQLVTLSCISSTESLTAPSSVPRVKSSYNFGRTFLGL
OanatDIA1R    1 MGL-RLGCRLFSAALSWM-T--LLQFLTRGHGPVADASPAPSLVPRVKPSYSFGRTFLGL
GgallDIA1R    1 MGR--WICCLCSRVADWL-M--LLLVLARSSNPSAAATASPS-APHVRPSYSFGRTFLGL
DreriDIA1R    1 ---------MAG--IWS--G-VWILCFILVFGTADP-----SPAPQ-DKSHDFRKIFLGL
SsalaDIA1R    1 MPLKRCDQRAVG-SVRLT-VCVLSWLLCVCWAPSVVEPAGTPPAPQ-QKALILQRAFLGL
NvitrDIA1     1 -----------------------MLVTKASVSSFLIALLILILGIYINRFNLKVAEITER
BflorDIA1     1 -------MRLVRLRQFRR-FFSGWRVRRMCGFFFFVVFFYCFVFQPFSYNQLTDTTFLGE
NvectDIA1     1 --MHFGGILRKRRYICRLVLLLIVIISFSDLLLWLSYQDGRAIKWDFSSQHKLTFQEIGL
CinteDIA1     1 -MLLKMKRYLTVWENGKYIVFIFILITLEVLLEMYNHRSQGSTRVMLEAKLLSDKWSATS
consensus     1         r    kmg   l   lkl  v  llvlvm    sl    qk  v er fl l
```

```
AaegyDIA1     45 ILC--PGCFPRP-VNCSFFENLKSIDS-----NELFEELLTHFNQHGVRYGT---LSGSE
CpipiDIA1     47 HLC--PECFVSPTVNCGHLANFVTIDS-----SNPWNQLTCRYNPHGVSYG----LLGSG
AgambDIA1     42 ILC--PECVSSG-LECNKFDDLFTLQD-----DNVFNIINNIFNSHTTRIGT---IVQEN
DyakuDIA1     38 RLC--PNCFFGQRELCGEIFQKIAEPSD---WSKLLKAISLLVDRRVIYFLR---FKDQD
DerecDIA1     38 RLC--PDCFVGQSELCVGIFQKIAEPSD---WSKLLKAISLIVDRRAIYYLR---LKDQA
DmelaDIA1     38 RLC--PDCFVGQSEPCGEIFQKIAEPSD---WSRLVKAISLLVDRRAIYFLR---LKDRD
DsechDIA1     38 RLC--PDCFVGQKEQCGEIFQKIAEPSD---WSRLVKAISLLVDRRAIHYLR---LKDQD
DananDIA1     38 RLC--PACFSAQREQCEEVFRAIREPSD---LSKLWKAVTLLFDRREIFWLE---IGPSQ
DpersDIA1     38 QLC--PACFAGQRWQCEEIFEAIAEPSD---WSKLLKGIALLIDRREIYFLQ---LNERH
DpseuDIA1     38 QLC--PACFAGQRWQCEEIFEAIAEPSD---WSKLLKGIALLIDRREIYFLQ---LNERH
DwillDIA1     38 LEC--PQCFLGQREQCEKFFDTIEEPAK---LDRLWKDLTLLVDPREIYWLE---LNENR
GaculDIA1     53 NKC--PACFGTS--WCRKFMNGQVSFET----WGRLRFLD-VFNVKNVYFAQ---YGEPR
OlatiDIA1     53 NKC--PACFGTS--WCRKFMNGQVSFET----WGRLRFLD-VFNVKNVYFAQ---YGEPR
TnigrDIA1     53 NKC--PACFGTS--WCRKFMNGQVSFET----WGRLRFLD-VFNVKNVYFAQ---YGEPR
TrubrDIA1     53 NKC--PACFGTS--WCRKFMNGQVSFET----WGRLRFLD-VFNVKNVYFAQ---YGEPR
DreriDIA1b    53 NKC--PACFGTS--WCRKFMNGQISFET----WGRLRFLD-VFNVKNVFFAQ---YGEPR
DreriDIA1a    53 NKC--PACFGTS--WCRKFMNGQVTFEM----WGRLRFLD-FFNVKNVFFAQ---YGEPR
BtaurDIA1     53 NKC--PACFGTS--WCRRFLNGQVVFEA----WGRLRLLD-FLNVKNVYFAQ---YGEPR
PpygmDIA1     53 NKC--PACFGTS--WCRRFLNGQVVFEA----WGRLRLLD-FLNVKNVYFAQ---YGEPR
CfamiDIA1     53 NKC--PACFGTS--WCRRFLNGQVVFEA----WGRLRLLD-FLNVKNVYFAQ---YGEPR
TtrunDIA1     53 NKC--PACFGTS--WCRRFLNGQVVFEA----WGRLRLLD-FLNVKNVYFAQ---YGEPR
PtrogDIA1     53 NKC--PACFGTS--WCRRFLNGQVVFEA----WGRLRLLD-FLNVKNVYFAQ---YGEPR
HsapiDIA1     53 NKC--PACFGTS--WCRRFLNGQVVFEA----WGRLRLLD-FLNVKNVYFAQ---YGEPR
MmulaDIA1     53 NKC--PACFGTS--WCRRFLNGQVVFEA----WGRLRLLD-FLNVKNVYFAQ---YGEPR
PvampDIA1     53 NKC--PACFGTS--WCRRFLNGQVVFEA----WGRLRLLD-FLNVKNVYFAQ---YGEPR
MmuscDIA1     53 NKC--PACFGTS--WCRRFLNGQVGFET----WGRLRLLD-FLNVKNVYFAQ---YGEPR
RnorvDIA1     53 NKC--PACFGTS--WCRRFLNGQVGFET----WGRLRLLD-FLNVKNVYFAQ---YGEPR
MdomeDIA1     53 NKC--PACFGTS--WCRRFLNGQVAFEA----WGRLRLLD-FLNVKNVYFGQ---YGEPR
GgallDIA1     53 NKC--PACWGTS--WCRKFLNGQLRLES----WGRLRLFD-FFNVKNVYFAR---YGEPR
XtropDIA1     53 NKC--PACFGTS--WCRKFMNGQLSFEG----WGRLRLLD-FFNVKNVHFAQ---YGEPR
SpurpDIA1     55 TKC--PACFGTS--LCKRFSRGDYRFHS----YSSIRLFD-YVNVKNVYFAT---Y----
RnorvDIA1R    60 DKC--NACIGTS--ICKKFFKEEIRLDN----SLVSHLGL-PSQDLLSYAAN---YSD-D
MmuscDIA1R    60 DKC--NACIGTS--ICKKFFKEEIRFDN----SLASHLGL-PPQDLHSYAAN---YSD-D
MmulaDIA1R    58 DKC--NACIGTS--ICKKFFKEEIRSDN----WLASHLGL-PPDFLLSYPAN---YSD-D
HsapiDIA1R    58 DKC--NACIGTS--ICKKFFKEEIRSDN----WLASHLGL-PPDSLLSYPAN---YSD-D
BtaurDIA1R    58 DKC--NACIGTS--ICKKFFKEEIRFDN----GLALHLGP-PPDDLPSYSAN---YSD-D
EcabaDIA1R    58 DKC--NACIGTS--ICKKFFKEEIRFDN----RLASHLGL-PSGYLPSYSAN---YSD-D
DordiDIA1R    58 DKC--NACIGTS--ICKKFFKEEIRFDN----SLASDLEL-ASDYLPSYSAN---YSD-D
MdomeDIA1R    57 DKC--NACIGTS--ICKKFFKEEIRFDN----WLASHLKL-PPDYFSTYPAN---YSD-D
OanatDIA1R    57 DKC--NACIGTS--ICKKFFKEEIRFDN----WPASHLKP-PPDHFPGYSAN---YTD-D
GgallDIA1R    55 DKC--NACIGTS--ICKKFFKEEIRFDT----WLSSHLKL-PPSYLLSYLGN---YTD-D
DreriDIA1R    41 DKC--NACIGTS--ICKKFFKDEIRFER----RLTAQSNL-SSADVRSYEGN---YTD-S
SsalaDIA1R    58 DKC--NACVGTS--ICKKLLKDQIRFDW----WMSPDTTL-PLAEKQSFPGN---LTD-D
NvitrDIA1     38 YKC--PACFGDS--MCQVIDSNEISFEY----TDFYSIFNNLFSVKNVYYGK---YKD--
BflorDIA1     53 DKC--PACFGTD--LCEEFENGKILFKY----SSRLRILD-IFNIKNVYFAI---YEG--
NvectDIA1     59 NKC--PACFGINLSICHKLLGGGVTVKS----NTPWSEER----IKGVSYGL---WGAVK
CinteDIA1     60 PTCGNFSCFGTS--LCKNRNRVGLSEDVPTLRSGWLSIYGELVDLHRVKLNVTYKLPSTL
consensus     61  kC  paCfgts   Crhf na v fd     w  lkll    nvk vyfaq   y d
```

```
AaegyDIA1     94 QR----VVIKNLNKEHGVEELISSICNDLKIYR----NCRLRNKEPYLR-----VLRQRV
CpipiDIA1     96 QK----VVIKTLNKNRAVEALRDAVCDELGLTQS---NCKFKSDENTLK-----VLRRKV
AgambDIA1     91 KL----AVMKHLNRDNTIEKLLQEYCDAYSLTKQ---NCRWAQHDGSVDR-AKEFITTSI
DyakuDIA1     90 Q---------VVAKRKIIDAQNQQ-IKDVKKAF---------------YE----------
DerecDIA1     90 Q---------VVAKRKIIDARNHK-AENVEKAF---------------YE----------
DmelaDIA1     90 QE----V-Q-LVAKRKIIDAQSNQ-IKNVKKDF---------------YE----------
DsechDIA1     90 QA----V-Q-LVAKRKIINAHSYQ-MKNVKRAF---------------YE----------
DananDIA1     90 EK----SSQ-LLAKRKSIVASHQS-LSSLQEEF---------------LN----------
DpersDIA1     90 QA----A-Q-LVAKRDAIGGHSYRNMGILREAF---------------LE----------
DpseuDIA1     90 QA----A-Q-LVAKRDAIGGHSYRNMGILGEAF---------------LE----------
DwillDIA1     90 EN-----VK-CLAKRKMTRKISKD------------------------------------
GaculDIA1    101 EGTRR-VVLKRLGSNQELAEIDQKICKRATGRPR-CDLIQAMYKTEFARINGDVRLLTPE
OlatiDIA1    101 EGTRR-VVLKRLGSNQELAEIDQKICKRATGRPR-CDLVQAMYKTEFARINGDVRLLTPD
TnigrDIA1    101 EGTRR-VVLKRLGSNQELADIDQKICKRATGRPR-CDLIQAMYKTEFARINGDVRLLTPD
TrubrDIA1    101 EGTRR-VVLKRLGSNQELADIDQKICKRATGRPR-CDLIQAMYKTEFARINGDVRLLTPE
DreriDIA1b   101 EGTRR-IVLKRLGSNQELADIDQKICKRATGRPR-CDLIQAMYKTEFARLNGDVRLLTPD
DreriDIA1a   101 EGTRR-VVLKRLGSNQELAEIDQKICKRATGRPR-CDLIQSMYKTEFARLNGDVRLLTPE
BtaurDIA1    101 EGGRRRVVLKRLGSQRELAQLDQSICKRATGRPR-CDLLQAMPRTEFARLNGDVRLLTPE
PpygmDIA1    101 EGGRRRVVLKRLGSQRELAQLDQSICKRATGRPR-CDLLQAMPRTEFARLNGDVRLLTPE
CfamiDIA1    101 EGGRRRVVLKRLGSQRELAQLDQSICKRATGRPR-CDLLQAMPRTEFARLNGDVRLLTPE
TtrunDIA1    101 EGGRRRVVLKRLGSQRELAQLDQSICKRATGRPR-CDLLQAMPRTEFARLNGDVRLLTPE
PtrogDIA1    101 EGGRRRVVLKRLGSQRELAQLDQSICKRATGRPR-CDLLQAMPRTEFARLNGDVRLLTPE
HsapiDIA1    101 EGGRRRVVLKRLGSQRELAQLDQSICKRATGRPR-CDLLQAMPRTEFARLNGDVRLLTPE
MmulaDIA1    101 EGGRRRVVLKRLGSQRELAQLDQSICKRATGRPR-CDLLQAMPRTEFARLNGDVRLLTPE
PvampDIA1    101 EGGRRRVVLKRLGSQRELAQLDQSICKRATGRPR-CDLLQAMPRTEFARLNGDVRLLTPE
MmuscDIA1    101 EGGRRRVVLKRLGSQRELAQLDQSICKRATGRPR-CDLLQAMPRTEFARLNGDVRLLTPE
RnorvDIA1    101 EGGRRRVVLKRLGSQRELAQLDQSICKRATGRPR-CDLLQAMPRTEFARLNGDVRLLTPE
MdomeDIA1    101 EGGRRRVVLKRLGSQRELAQLDQSICKRATGRPR-CDLLQAMPKTEFARLNGDVRLLTPD
GgallDIA1    101 EGSRR-VVLKRLGSAQELADIDAKICRRATGRGR-CDLLQALHATEFASLNGDVRLLTPD
XtropDIA1    101 EGSRR-VVLKRLGSNHELSELDQRICKKATGRPR-CDLVQAMYKTDFARLNGDVRLLTPD
SpurpDIA1     99 -LDQKPVVMKKLGHNSEHNQFDKDLCTNAESGLRGCDVSQQIYKSKLSDIMYAERLEEKD
RnorvDIA1R   107 SKTWRPVEISRLVSKYQSEISDKRICASASGPKT-CSIERILRKTGRFQKWLQAKRLTPD
MmuscDIA1R   107 SKTWRPVEISQLVSRYQIEISDRRICASVSAPKT-CSIERILQKTGRFQKWLQAKRLTPD
MmulaDIA1R   105 SKIWRPVEIFRLVSNYQNEISDRRICASASAPRT-CSIERVLRKTERFQKWLQAKRLTPD
HsapiDIA1R   105 SKIWRPVEIFRLVSKYQNEISDRRICASASAPKT-CSIERVLRKTERFQKWLQAKRLTPD
BtaurDIA1R   105 FKTWRPVEISRLVSKQQNKISDGRICASAAAPKT-CSIERVLRKTGRFQKWLQAKRLTPD
EcabaDIA1R   105 SKTWRPVEVSRLVSKQQNEISDRRICAFAAAPKT-CSIERVLRKTRRFQKWLQAKRLTPD
DordiDIA1R   105 SKTWRPVEIFRLVSKEQNEISDRRICAFASAPKT-CSIERILRKTGRFQKWLQAKRLTPD
MdomeDIA1R   104 TKSWRPVEISRLISKYQHELSDRRICASASGSKT-CSIERVLRKTERVKKWLKAKRLTPD
OanatDIA1R   104 SKSWRLVEISRLIGKDQNELSDQKICTSVSLVKT-CSIERVLRKTERFQKWLKARRLTPA
GgallDIA1R   102 AQSWRMVDITRLTTKYQHDRADQHICTSLLKTKT-CSLERALRRTHRFQKWLRAKRLTPD
DreriDIA1R    88 TAGWRPVVVSRLMSPHLHQLSDNSICTSAGKGKS-CSIEGVLRATSRFQTWVHSNLLLPS
SsalaDIA1R   105 SLSWRPVVLSFLSSPCLHSSSDRSICRSVGRQGP-CSIEAVLRVTPRFQSLNQSHLLLPH
NvitrDIA1     85 ----KKVIMKKLAQESELKTFDEMICSDDELHEL-CYQSESRKQDSKTNFYKLVESELAN
BflorDIA1     99 ----MEVALKRLGHNSEFDQLDRWICETGKEGPQ-CHIPSVTYYSNFSRVIYEG--LSPK
NvectDIA1    106 QR----VVLKTLGTSSELTAFDNEICRGVGKDSN-CKISQEAWDSDVLVP--------KP
CinteDIA1    118 PCNGETCSVQIETSVLSGNNVNKWICDKFKKPTN-CDTGKVFKSWRAMNKITSAKGRLDP
consensus    121     r vvikrlas   v  ldq ick asg    c l   m kte        rlltpe
```

```
AaegyDIA1    141 LNDNQLEGAILCPLMDE--EALKRFLFEID------E-----NDLNKILLMRINAQPLLL
CpipiDIA1    144 L-EQELEGCIICPSKDE--KALNRLLNEVE------G-----TELLQLILLKVNVQPLLL
AgambDIA1    143 LDSARVEGCIFCPATNNR-QSLQRFLSLFDS----TD-----NELWNLLAVRTNVEPLLI
DyakuDIA1    115 -LGERPGGFHLCRTPSRKP-RFVSYLEQRGH----A-------SASVWFYMMHYVSPLLM
DerecDIA1    115 -QEEQPGGFHLCRTPSRKPPRFVSYLEQRGH----A-------SASVWFYMMHYVSPLLM
DmelaDIA1    118 -LEERPGGFHLCRTLAREP-RFVSYLEQRGH----A-------AASVWFYMMYYVSPLLM
DsechDIA1    118 -LEERPGGFHLCRTLAREP-RFVSYLEQRGH----VY-----GSTSVWFYMMHYVSPLLM
DananDIA1    119 -LEERPGGFHLCRTAQKSS-PFINYLNQRGY----D-------SSSIYFYMLHNISPLLM
DpersDIA1    119 -MEERPGGFQLCRSGFRTP-RFVSYLEQRGH----E-------AATIWYYMMHSITPLLM
DpseuDIA1    119 -MEERPGGFQLCRSGFRTP-RFVSYLEQRGH----E-------AATIWYYMMHSITPLLM
DwillDIA1    108 -LHFNPGGFQMCRNSTR----FLAYLEERGY----L-------PATIGYYIHHSISPLLM
GaculDIA1    159 VVEGWSDLVH-CPSQRLLDRVVRRYAETKDSGSFLLKNLKDTERMQLLMTLAFNPEPLVL
OlatiDIA1    159 VVEGWSDLVH-CPSQRLLDRVVRRYAETKDSGSFLLKNLKDTERMQLLMTLAFNPEPLVL
TnigrDIA1    159 VVEGWSDLVH-CPSQRLLDRVVRRYAETKDSGSFLLKNLKDTERMQLLMTLAFNPEPLVL
TrubrDIA1    159 VVEGWSDLVH-CPSQRLLDRVVRRYAETKDSGSFLLKNLKDTERMQLLMTLAFNPEPLVL
DreriDIA1b   159 VVEGWSDLVH-CPSQRLLDRIVRRYAETKDSGSFLLKNLKDTERMQLLMTLAFNPEPLVL
DreriDIA1a   159 VVEGWSDLVH-CPSQRLLDRVVRRYAETKDSGSFLLKNLKDTERMQLLMTLAFNPEPLVL
BtaurDIA1    160 AVEGWSDLVH-CPSQRLLDRLVRRYAETKDSGSFLLRNLKDSERMQLLLTLAFNPEPLVL
PpygmDIA1    160 AVEGWSDLVH-CPSQRLLDRLVRRYAETKDSGSFLLRNLKDSERMQLLLTLAFNPEPLVL
CfamiDIA1    160 AVEGWSDLVH-CPSQRLLDRLVRRYAETKDSGSFLLRNLKDSERMQLLLTLAFNPEPLVL
TtrunDIA1    160 AVEGWSDLVH-CPSQRLLDRLVRRYAETKDSGSFLLRNLKDSERMQLLLTLAFNPEPLVL
PtrogDIA1    160 AVEGWSDLVH-CPSQRLLDRLVRRYAETKDSGSFLLRNLKDSERMQLLLTLAFNPEPLVL
HsapiDIA1    160 AVEGWSDLVH-CPSQRLLDRLVRRYAETKDSGSFLLRNLKDSERMQLLLTLAFNPEPLVL
MmulaDIA1    160 AVEGWSDLVH-CPSQRLLDRLVRRYAETKDSGSFLLRNLKDSERMQLLLTLAFNPEPLVL
PvampDIA1    160 AVEGWSDLVH-CPSQRLLDRLVRRYAETKDSGSFLLRNLKDSERMQLLLTLAFNPEPLVL
MmuscDIA1    160 AVEGWSDLVH-CPSQRLLDRLVRRYAETKDSGSFLLRNLKDSERMQLLLTLAFNPEPLVL
RnorvDIA1    160 AVEGWSDLVH-CPSQRLLDRLVRRYAETKDSGSFLLRNLKDSERMQLLLTLAFNPEPLVL
MdomeDIA1    160 AVEGWSDLVH-CPSQRLLDRLVRRYAETKDSGSFLLRNLKDSERMQLLLTLAFNPEPLVL
GgallDIA1    159 AVEGWSDLVH-CPSQRLLDRLVRRYAETKDSGSFLLRNLKDSERMQLLITLAFNPEPLVL
XtropDIA1    159 VVEGWSDLVH-CPSQRLLDRLVRRYAETKDSGSFLLRNLKDTERMQLLLTLAFNPEPLVL
SpurpDIA1    158 -VMGLSDIVR-CPSRRLLDRIWDTFGERQ----FD-KSLARDHKLMLATTIAFNPEPLIL
RnorvDIA1R   166 LVQGLPSPFLHCPSQRLLDRVVRRYAEVVDAGSIFMDHFTDRDKLRLLYTLAVNAHPIIL
MmuscDIA1R   166 LVQGLPSPFLRCPSQRLLDRVVRRYAEVVDAGSIFMDHFTAGDKLRLLYTLAVNAHPIIL
MmulaDIA1R   164 LVQGLASPLLRCPSQRLLDRVVRRYAEVADAGSIFMDHFTDRDKLRLLYTLAVNSHPILL
HsapiDIA1R   164 LVQGLASPLLRCPSQRLLDRVVRRYAEVADAGSIFMDHFTDRDKLRLLYTLAVNSHPILL
BtaurDIA1R   164 LVRGLSSPLLRCPSQRLLDRVVRRYAEVADAGSIFMDHFTDRDKLRLLYTLAVNTHPVLL
EcabaDIA1R   164 LVRGLPSPLLRCPSQRLLDRVVRRYAEVPDAGSIFMDHFTDRDKLRLLYTLAVNAHPILL
DordiDIA1R   164 LVQGLPSPFLRCPSQRLLDRVVRRYAEVADAGSIFMDHFTDRDKLRLLYTLAVNAHPIFL
MdomeDIA1R   163 LVQGLPSPMLRCPSQRLLDRIVRRYAEVADAGSVFMDHFTDRDKLRLLYTLSINAHPIML
OanatDIA1R   163 LVQGLPSLLLRCPTQRLLDRIVRRYAEVPDAGSVYMDHFTDRDKLRLLYTLSVNAHPVLL
GgallDIA1R   161 LVQGLSSPMLRCPSQRLLDRIVRRYAEVPDAGSIYMDHLTDQDKLRLLYTLSVNSHPILL
DreriDIA1R   147 MVKGLVTPMLRCPSQRLLDRIVRRYFEVTDVGSVQMKHFNEKDKLRLLYTLAVNQQPLIL
SsalaDIA1R   164 IVKGLAPPLLRCPSQRLLDRIVRRYAEVVDVGSVQMKHFSERDKLRLLYTLAVNQQPLIL
NvitrDIA1    140 VGGDINNKMRLCPTTTKLEKLFVNVKFQSHAE-------NEDYFKYLWSTIKINPEPLIL
BflorDIA1    152 SLGDMSDMVR-CPSQRLIDRVLEKFAEHLG---KETEELSYQEKLHFLSTLKFNPEPLML
NvectDIA1    153 PLQDAVRKYFDCPSTRFLEKIESVYGQETPG------QLSLVETIHLITGLHLNPELLLL
CinteDIA1    177 NLLKDISPPLQCPSTRIADRIVRRVANKQGG--VLLRDLSYTEKKRLIFTCKMSPGSVVQ
consensus    181  veg  e vh Cpsqrlldrvvrryae rd gs  v  l d erl ll tla npeplll
```

```
AaegyDIA1   188 KLL-----QERNFPVPKLIFQGGFTLVESYEG-EALAKYYDRPLN--VRLLIANELIKAS
CpipiDIA1   190 ELF-----DGRGFPVPKVIFQGGFQLFESFDG-DALVNFYDSSLN--IRLRIAKELIQAS
AgambDIA1   193 KLLTVNYHDEPPLYVPKLLHTFGFSIIETYEG-KTLEHYYDFPFV--TRMRIAAELIRAG
DyakuDIA1   162 QEL-----YLQGFPVPTTYASCGLTHFQSYAG-RTLTNYFEGEED--LRVELALQLMQLS
DerecDIA1   163 QEL-----HLQGFPVPTTYASCGLTHFQAYAG-RTLTNYFEAEEG--LRMELALQLIQLS
DmelaDIA1   165 QEL-----HLQGFPVPTTYASCGLTHFQSNAG-RTLAHYVDAEEG--LRVELALQLIQLS
DsechDIA1   167 QEL-----HLQGFPVPTPYASCGLTHFQSYAG-RTLAHYVNAEEG--LRLELALQLIQLS
DananDIA1   166 QEL-----QNLDFPVPNTYAICGLTHFQAYAG-RTLNHYTEAAED--LRREIARQLIHLS
DpersDIA1   166 QEL-----FLRGFPVPRSYAACGLTHFQAYAG-RTLVHYATAGEN--LRLEMARQLLQLS
DpseuDIA1   166 QEL-----FLRGFPVPRSYAACGLTHFQAYAG-RTLVHYATAGEN--LRLEMARQLLQLS
DwillDIA1   152 QEL-----WIMDFPVPRSFAVCGSTLFQAYAG-STLSNYLKAPID--LRLEMAKQLLQLA
GaculDIA1   218 QSFP----SDEGWPFAKYLGACGRMVAVNYVG-EELWSFYNAPWE--KRVDLARQLMDIA
OlatiDIA1   218 QSFP----SDEGWPFAKYLGACGRMVAVNYVG-EELWNFYNAPWE--KRVDLARQLMDIA
TnigrDIA1   218 QSFP----SDEGWPFAKYLGACGRVVAVNYVG-EELWSFYNAPWE--KRVDLARQLMDIA
TrubrDIA1   218 QSFP----SDEGWPFAKYLGACGRVVAVNYVG-EELWSFYNAPWE--KRVDLARQLMDIA
DreriDIA1b  218 QSFP----SDEGWPFAKYLGACGRMVAVNYVG-EELWSFFNAPWE--KRVDLAKQLMDIA
DreriDIA1a  218 QSFP----SDEGWPFAKYLGACGRMVAVNYVG-EELWSFYNAPWE--KRVDLAKQLMDIA
BtaurDIA1   219 QSFP----SDEGWPFAKYLGACGRMVAVNYVG-EELWSYFNAPWE--KRVDLAWQLMEIA
PpygmDIA1   219 QSFP----SDEGWPFAKYLGACGRMVAVNYVG-EELWSYFNAPWE--KRVDLAWQLMEIA
CfamiDIA1   219 QSFP----SDEGWPFAKYLGACGRMVAVNYVG-EELWSYFNAPWE--KRVDLAWQLMEIA
TtrunDIA1   219 QSFP----SDEGWPFAKYLGACGRMVAVNYVG-EELWSYFNAPWE--KRVDLAWQLMEIA
PtrogDIA1   219 QSFP----SDEGWPFAKYLGACGRMVAVNYVG-EELWSYFNAPWE--KRVDLAWQLMEIA
HsapiDIA1   219 QSFP----SDEGWPFAKYLGACGRMVAVNYVG-EELWSYFNAPWE--KRVDLAWQLMEIA
MmulaDIA1   219 QSFP----SDEGWPFAKYLGACGRMVAVNYVG-EELWSYFNAPWE--KRVDLAWQLMEIA
PvampDIA1   219 QSFP----SDEGWPFAKYLGACGRMVAVNYVG-EELWSFFNAPWE--KRVDLAWQLMEIA
MmuscDIA1   219 QSFP----SDEGWPFAKYLGACGRMVAVNYVG-EELWSYFNAPWE--KRVDLAWQLMEIA
RnorvDIA1   219 QSFP----SDEGWPFAKYLGACGRMVAVNYVG-EELWSYFNAPWE--KRVDLAWQLMEIA
MdomeDIA1   219 QSFP----SDEGWPFAKYLGACGRMVAVNYVG-EELWSYFNAPWE--KRVDLAWQLMEIA
GgallDIA1   218 QSFP----SDEGWPFAKYLGACGRMVAVNYVG-EELWSYFNAPWE--KRVDLAWQLMEIA
XtropDIA1   218 QSFP----SDEGWPFAKYLGACGRMVAVNYVG-EELWSYFNAPWE--KRVDLAWQLMEIA
SpurpDIA1   211 QAFP----NRKGWPFPAYMGACGRFTVQEYSG-HSLSYFYKFKFG--VRAALAVQALKIA
RnorvDIA1R  226 QIFP----GAEGWPMPKYLGSCGRFLVSTST--RPLQEFYDAPPE--QAADIAYQLLGVL
MmuscDIA1R  226 QIFP----GAEGWPMPRYLGSCGRFLVSTST--RPLQEFYDASPE--QAADLAYQLLRVL
MmulaDIA1R  224 QIFP----GAEGWPLPQYLGSCGRFLVSTST--RPLQEFYGAPPD--QAADLAYQLLGVL
HsapiDIA1R  224 QIFP----GAEGWPLPKYLGSCGRFLVSTST--RPLQEFYDAPPD--QAADLAYQLLGVL
BtaurDIA1R  224 QIFP----GAEGWPLPQYLGSCGRFLVSTST--SPLQEFYGAPPD--QAADLAYQLLGVL
EcabaDIA1R  224 QIFP----GAEGWPLPRYLGSCGRFLISTST--SPLQEFYSAPPD--QAADLAYQLLGVL
DordiDIA1R  224 QIFP----GAEGWPLPKYLGSCGRFFVSTST--RPLQEFYGAPPD--EAADLAYQLLGVL
MdomeDIA1R  223 QIFP----GAEGWPLPRYLGSCGRFLVSTST--QPLEEFYSSPAD--QAADLAYQLLGIL
OanatDIA1R  223 QVFP----GAEGWPFPKYLGSCGRLLVSTST--TPLLELSSGPPD--RAADLGHQLLGVI
GgallDIA1R  221 QIFP----DVEGWPFPRYLGSCGRLVVSAST--RPLRDFFRAAPE--VAADLALQLLAVL
DreriDIA1R  207 QMFP----GTEGWPFLRYHGSCGRLMVWAAS--RALRTLFSSPLE--RRADLAYQLLHIT
SsalaDIA1R  224 QMFP----GTEGWPFPRYQGSCGRLMVWASS--RPLWGLYGSSREFVQRVDVAYQLLHIT
NvitrDIA1   193 QILP----AKEGWPTPKYFGACGRLIVEEYIG-LPLSSFIDEPWIR--RAKIASSLLQAA
BflorDIA1   208 QTFP----ITEGWPFPFYFGACGRLTVVQKCD-KTLASYYSAPWL--KRVELSLQMMKIA
NvectDIA1   207 QIFR----QEEGWPFPRFYGVCGRIMVVEDSG-PPISSFLEESWD--VRAQIAVNLITLA
CinteDIA1   235 SMFP----PYEGWLFQRTVGSCGALMVHGRSIVTSLYELYDAPWK--VRLDVAIQLLDLA
consensus   241 q fp       egwpfpkylgacGrml   y g   l  yy apfe   rldla qli ia
```

```
AaegyDIA1    240 LNFTAGVDNFRFYLTDISPDNIVVSMSSEE-EIKVTFVDLNNVIILDSHSKRLKP---SK
CpipiDIA1    242 FLFTEGVNGFRFYLTDINPDNIAVQAQPSG-SFQVSFIDLDNVIILDSQSKRLDRR--SK
AgambDIA1    250 FKFTEGIHGFRVFLTDINPDNVVVNLKNDNKQVYVSIVDLDNVILLDSWAEVFLT---KN
DyakuDIA1    214 LKLTFGFSDFRIMLTDFTGDNFAYDEHTKK----VYLIDLDSVVLVD----ASSAT--GQ
DerecDIA1    215 LKLTFGFSDFRIFLTDFTGDNFAYDEDSKR----VYLIDLDSVVLVD----ASSAA--GQ
DmelaDIA1    217 LKLTFGFADFRIILTDFTADNLAYDEDTKK----VYLIDLDSVVLVD----ASFAA--GH
DsechDIA1    219 LKLTFGFADFRIILTDFTADNLAYDEETKK----VYLIDLDSVVLVD----ASSMA--GQ
DananDIA1    218 LKLTFGFADFRIFLTDFTSDNLTFDEDTRR----VLLIDLDSVVMVD----AASTS--GQ
DpersDIA1    218 LKLTFGFADFRIFLTDFTADNLAFDEASQS----VILIDLDSVVLVD----AAVPL--GD
DpseuDIA1    218 LKLTFGFADFRIFLTDFTADNLAFDEASQS----VILIDLDSVVLVD----AAVPL--GD
DwillDIA1    204 LKLSAGFKGFRIYLTDFTADNFAYNEANGT----VLLIDMDTMVLVESNDEAESES--QL
GaculDIA1    271 EQLTNNDFDFALYLLDVSFDNFAVGPRDGK----VIVVDAENVLVADKRLIKQNK-PESF
OlatiDIA1    271 EQLTNNDFDFALYLLDVSFDNFAVGPRDGK----VIVVDAENVLVADKRLIKQNK-PESY
TnigrDIA1    271 EQLTNNDFEFALYLLDVSFDNFAVGPRDGK----VIIVDAENVLVADKRLIKQNK-PENF
TrubrDIA1    271 EQLTNNDFEFALYLLDVSFDNFAVGPRDGK----VIIVDAENVLVADKRLIKQNK-PENY
DreriDIA1b   271 EQLTNNDFDFALYLLDVSFDNFAVGPRDGK----VIVVDAENVVVADKRLIKQNK-PESY
DreriDIA1a   271 EQLTNNDFDFALYLLDVSFDNFAVGPRDGK----VIVIDAENVIVVDKRLVKQNK-PESY
BtaurDIA1    272 EQLTNNDFEFALYLLDVSFDNFAVGPRDGK----VIIVDAENVLVADKRLIRQNK-PENW
PpygmDIA1    272 EQLTNNDFEFALYLLDVSFDNFAVGPRDGK----VIIVDAENVLVADKRLIRQNK-PENW
CfamiDIA1    272 EQLTNNDFEFALYLLDVSFDNFAVGPRDGK----VIIVDAENVLVADKRLIRQNK-PENW
TtrunDIA1    272 EQLTNNDFEFALYLLDVSFDNFAVGPRDGK----VIIVDAENVLVADKRLIRQNK-PENW
PtrogDIA1    272 EQLTNNDFEFALYLLDVSFDNFAVGPRDGK----VIIVDAENVLVADKRLIRQNK-PENW
HsapiDIA1    272 EQLTNNDFEFALYLLDVSFDNFAVGPRDGK----VIIVDAENVLVADKRLIRQNK-PENW
MmulaDIA1    272 EQLTNNDFEFALYLLDVSFDNFAVGPRDGK----VIIVDAENVLVADKRLIRQNK-PENW
PvampDIA1    272 EQLTNNDFEFALYLLDVSFDNFAVGPRDGK----VIIVDAENVLVADKRLIRQNK-PENW
MmuscDIA1    272 EQLTNNDFEFALYLLDVSFDNFAVGPRDGK----VIIVDAENVLVADKRLIRQNK-PENW
RnorvDIA1    272 EQLTNNDFEFALYLLDVSFDNFAVGPRDGK----VIIVDAENVLVADKRLIRQNK-PENW
MdomeDIA1    272 EQLTNNDFEFALYLLDVSFDNFAVGPRDGK----VIIVDAENVLVADKKLIRQNK-PENW
GgallDIA1    271 EQLTNNDFEFALYLLDVSFDNFAVGPRDGK----VIIVDAENVLVADKRLIRQNK-PENW
XtropDIA1    271 EQLTNNDFDFALYLLDVSFDNFAVGPRDGK----VIIVDAENVLVADKKLIKQNK-PENW
SpurpDIA1    264 EQLSENRDEFALYLTDVSEDNLAVND-NGE----VLVIDAENIIVVDRKKIKEDA-NPGW
RnorvDIA1R   278 ESLRSNDLNYFFYFTHVDAGMFGIFD-NGH----LFIRDASALGIIDKQEASQA---ADR
MmuscDIA1R   278 ESLRSNDLNYFFYFTHVDAGMFGIFD-NGH----LFIRDASALGIIDKQEGSQA---AAR
MmulaDIA1R   276 ESLRSNDLNYFFYFTHIDAGMFGVFN-NGH----LFIRDASAVGVIDKQEGSQE---ATR
HsapiDIA1R   276 ESLRSNDLNYFFYFTHIDAGMFGVFN-NGH----LFIRDASAVGVIDKQEGSQE---ANR
BtaurDIA1R   276 ESLRSNDLNYFFYFTHVDADMFGIFN-NGH----LFIRDASALGVIDRQEGSQA---ASG
EcabaDIA1R   276 ESLRSNDLNYFFYFTHVDAGMFGIFN-NGH----LFIRDASTLGVIDRQEGSQA---AAR
DordiDIA1R   276 ESLRSNDLNYFFYFTHVDAAMFGIFS-NGH----LFIRDASALGVIDKQEGSQA---APK
MdomeDIA1R   275 DSLRNNDLNYFFYFTRIDAGTFGIFD-NGH----LFIRDASTVGVIDKQRGSLT---LDR
OanatDIA1R   275 EFLRHNDWNYFFYFTRIHAGTFGVFD-NGH----LFIRDASSLGVIDRQEGKRP---ARS
GgallDIA1R   273 HSMGTNDLNYFFYFTRVDVGTFGVFS-NGH----LFIRDASTLGIIDKEEGSQP---IDG
DreriDIA1R   259 QSLSANSLRFRLFYTRIPEDMFGILE-DNK----VFIVDTSTIGIIDLQEGHPPD--KDL
SsalaDIA1R   278 QGLGHNSLGFLLYYTRLGEDMFGLLD-DQR----VFITDASSIGVIDLEQGFPPD-PPSQ
NvitrDIA1    246 DTLMSKNSEFAFYLTDISMDNIAVNN-EDK----AIFVDLENIIIVEKNPPEKA--LVGI
BflorDIA1    261 EYLTNNEADFGLYLTDISYENFGVTS-DGN----LFVIDVENVIVVDKQKIKADK-PRNW
NvectDIA1    260 HQLSSALDDWALYIADPAPGNFGVSD-SGK----VTLLDLEHLVVVDLSEVQME----EA
CinteDIA1    289 EHFTYNDLNYMFALHRLTPRDIGISD-TGK----LRIKNADNVIIDNNSKNDK----GL
consensus    301 e lt nd df lyltdvs dnfav    gk    vlivdadnvivldk   rq
```

```
AaegyDIA1   296 QKHVHSRIDCDGC-----FAYIQEDICVHQISDINLFAVCQLLLENLNGDSKRGFLHSLG
CpipiDIA1   299 ARNIHSRIPCDGC-----FAYVQEDLCSYQHSDINQFAICQLLYENLNGDREGGFLHIQP
AgambDIA1   307 THHVHSKIECNGC-----FAYVQEDVCRYQNSDLNLFATCQLLLENLNGHYAKGLLHYDR
DyakuDIA1   264 AEKYEP-LPGEG------FTFDVSAFCSGHQLDANIYQACLLLRDYLLKNLNN-------
DerecDIA1   265 AEKYVP-LPGEG------FTFDVSAFCSGHQLDANIYQACLLLRDFLLKNLDN-------
DmelaDIA1   267 AEKYEP-LTGEG------FTFDVSAFCSGHQLDANIYQACLLLRDYLLKNLDN-------
DsechDIA1   269 AEKYEP-LAGEG------FTFDVSAFCSGHQLDANIYQACLLLRDSLLKNLDN-------
DananDIA1   268 AEKYEP-LPGEG------FTFDVSAFCSGRQLDANVYQACLLLRDFLLKDLDN-------
DpersDIA1   268 AQKYEP-LPGDG------FTFDVKSFCGGQQLDANVYQACLLLKDFLLRDLAN-------
DpseuDIA1   268 AQKYEP-LPGDG------FTFDVKSFCGGQQLDANVYQACLLLKDFLLRDLAN-------
DwillDIA1   258 SKKYKP-FPGEG------FTYDVNAFCDNQDLDANIYQVCLLLRDQLLPDVKN-------
GaculDIA1   326 DVWYESRFEECD--REACLSFSKESLCSRVTVDHNYYAVCQNLLSRYATWRGTT------
OlatiDIA1   326 DVWYESRFEECD--REACLSFSKDSLCSRVTVDHNYYAVCQNLLSRYATWRGTT------
TnigrDIA1   326 DVWYESRFEECD--REACLSFSKDSLCSRVTVDHNYYAVCQNLLSRYATWRGTT------
TrubrDIA1   326 DVWYESRFEECD--REACLSFSKDSLCSRVTVDHNYYAVCQNLLSRYATWRGTT------
DreriDIA1b  326 DVWYESRFEECD--KEACLSFSKDMLCSRVTVDHNYYAICQNLLSRYATWRGSS------
DreriDIA1a  326 DVWYESRYEECD--KEACLSFSKDILCSRVTVDHNYYAVCQDLLSRFSSWRGST------
BtaurDIA1   327 DVWYESKFDDCD--KEACLSFSKEILCARATVDHNYYAVCQNLLSRHATWRGTS------
PpygmDIA1   327 DVWYESKFDDCD--KEACLSFSKEILCARATVDHNYYAVCQNLLSRHATWRGTS------
CfamiDIA1   327 DVWYESKFDDCD--KEACLSFSKEILCARATVDHNYYAVCQNLLSRHATWRGTS------
TtrunDIA1   327 DVWYESKFDDCD--KEACLSFSKEILCARATVDHNYYAVCQNLLSRHATWRGTS------
PtrogDIA1   327 DVWYESKFDDCD--KEACLSFSKEILCARATVDHNYYAVCQNLLSRHATWRGTS------
HsapiDIA1   327 DVWYESKFDDCD--KEACLSFSKEILCARATVDHNYYAVCQNLLSRHATWRGTS------
MmulaDIA1   327 DVWYESKFDDCD--KEACLSFSKEILCARATVDHNYYAVCQNLLSRHATWRGTS------
PvampDIA1   327 DVWYESKFDDCD--KEACLSFSKEILCARVTVDHNYYAVCQNLLFRHATWRGTS------
MmuscDIA1   327 DVWYESKFDDCD--KEACLSFSKEILCARVTVDHNYYAVCQNLLSRHATWRGTS------
RnorvDIA1   327 DVWYESKFDDCD--KEACLSFSKEILCARVTVDHNYYAVCQNLLSRHATWRGTS------
MdomeDIA1   327 DVWYESKFDDCD--KEACLSFSKEILCSRATVDHNYYAICQNLLSRHATWRGTS------
GgallDIA1   326 DVWYESKFDDCD--KEACLSFSKEILCARVTVDHNYYAVCQNLLSRHATWRGTS------
XtropDIA1   326 DVWYESKFDDCD--KEACLSFSKEILCSRATVDHNYYAICQNLLSRHATWRGTS------
SpurpDIA1   318 DVKHQSEHEGCGS-RRECIMFSHTDLCNHYYSDHNYYAVCQGLFGKDSPHG--D------
RnorvDIA1R  330 TGENEDIFSCL---VSGCQVQ--MSSCDTVPEKQNLVLVCQQLLPQLLRG----------
MmuscDIA1R  330 TGENEDIFSCL---VSDCQIQ--LSSCDTVPEKQSLVLVCQQLLPQLLQG----------
MmulaDIA1R  328 AGENKDIFSCL---VSGCQAQ--LPSCESVSEKQSLVLVCQKLLPRLLQG----------
HsapiDIA1R  328 AGENKDIFSCL---VSGCQAQ--LPSCESISEKQSLVLVCQKLLPRLLQG----------
BtaurDIA1R  328 AGDNKDIFSCL---VSGCQTK--LPSCDTIPEKQNLVLVCSQVLPLLLQA----------
EcabaDIA1R  328 AGENKDIFSCL---LSDCQAE--LPSCDTVPEKQSLVLVCRQVLPRLLQG----------
DordiDIA1R  328 AGENQDIFSCL---VSGCQAQL-LPSCDNIPDKQSVVLVCQQLLPQLLQG----------
MdomeDIA1R  327 QEESKDIFSCL---VLDCQSP--FPSCSSVKEKQSWVMACQQLLPQLLKE----------
OanatDIA1R  327 SPEPRDIFSCL---AADCQSD--LPSCNTVQEHQSLVLVCRELLPKLLEG----------
GgallDIA1R  325 QQEYKDIFSCL---TVDCQSA--FVSCNSIREKQSLVMVCQELLPKLLRG----------
DreriDIA1R  312 LPEELDVFSCL---SGSCVRP---PPCETVREAQSFILLCKYILNNLLTSN---------
SsalaDIA1R  332 TGSDGDIFSCLGQGTSPCHRS---PPCSSVRPTQSLTLLCTALLPRLLLTERGA------
NvitrDIA1   299 ESWNETYTNAVDLDCQDCFVFSPNDICSHKVSDHNFYAICQHILTQALGAVFHN------
BflorDIA1   315 EARYQSHFDECPG-MSNCLSFDVSALCRYLHTDHNYYAVCRNMLSEYASEMGKP------
NvectDIA1   311 N--DKPKPSDCS--QPDCLSFSPSDMCSGGSRDHNYYAICQGVLAGSSRRQ---------
CinteDIA1   340 Y---EVRYKQEK------VDITEENLCDHSENDVNYYVICRHFLGGTWKERKGK------
consensus   361    yesrf        aclsf    iC    ld nyyavCqnll r
```

```
AaegyDIA1   351 TDS---KLEAFRKLLH----------QCVYCQPPYCED-----------RQEVLQRIMEI
CpipiDIA1   354 NDDSQPRLSEIRQLLH----------HCVYCVPPDCRD-----------RQGLLQQVQEI
AgambDIA1   362 DAEAFPVLGEASKMLQN------LLSECVYCQPPDCQN-----------RSHILKDMLHI
DyakuDIA1   310 --------ERLQLLLE----------QCVACQDDFCDM-----------RFQHAYDLIKM
DerecDIA1   311 --------ERLQLLLE----------QCVACQDDFCDM-----------RFQHAYDLIKV
DmelaDIA1   313 --------EKLQLLLE----------QCVACQDDFCDM-----------RFQHAYDLIKV
DsechDIA1   315 --------EKLQLLLE----------QCVACQDDFCDM-----------RFQHAYDLIKE
DananDIA1   314 --------ERLQLLLE----------KCVECQDDLCDM-----------RFQYAYDLIKL
DpersDIA1   314 --------GRLEMLLE----------QCVRCDDATCDM-----------RFQQAYDLIKL
DpseuDIA1   314 --------GRLEMLLE----------QCVRCDDATCDM-----------RFQQAYDLIKL
DwillDIA1   304 --------HRLKEMLN----------DCVQCEDDKCDI-----------RFKYANNLIDL
GaculDIA1   378 ----GGLLHDPPAHIAK---DGQLLTLLDECTRPKKRY-G---------RFQAAKELREY
OlatiDIA1   378 ----GGLLHDPPAHIAK---DGQLEALLDECTRPKKRY-G---------RFQAAKELREY
TnigrDIA1   378 ----GGLLHDPPAHIAK---DGQLEALLDECTKPKKRY-G---------RFQAAKELREY
TrubrDIA1   378 ----GGLLHDPPAHIAK---DGQLEALLDECTKPKKRF-G---------RFQAAKELREY
DreriDIA1b  378 ----GGLLHDPPPHIAK---DGQLEALLEECANPKKRY-G---------RFQAAKELRDY
DreriDIA1a  378 ----GGLLHDPPPDVVK---DGRLIALLDECTRPQKRY-G---------RFQAAKELREF
BtaurDIA1   379 ----GGLLHDPPSEIAK---DGRLEALLDECANPKKRY-G---------RFQAAKELREY
PpygmDIA1   379 ----GGLLHDPPSEIAK---DGRLEALLDECANPKKRY-G---------RFQAAKELREY
CfamiDIA1   379 ----GGLLHDPPSEIAK---DGRLEALLDECANPKKRY-G---------RFQAAKELREY
TtrunDIA1   379 ----GGLLHDPPSEIAK---DGRLEALLDECANPKKRY-G---------RFQAAKELREY
PtrogDIA1   379 ----GGLLHDPPSEIAK---DGRLEALLDECANPKKRY-G---------RFQAAKELREY
HsapiDIA1   379 ----GGLLHDPPSEIAK---DGRLEALLDECANPKKRY-G---------RFQAAKELREY
MmulaDIA1   379 ----GGLLHDPPSEIAK---DGRLEALLDECANPKKRY-G---------RFQAAKELREY
PvampDIA1   379 ----GGLLHDPPSEIAK---DGRLEALLDECANPKKRY-G---------RFHAAKELREY
MmuscDIA1   379 ----GGLLHDPPSEIAK---DGRLEALLDECTNPKKRY-G---------RFQAAKELRGY
RnorvDIA1   379 ----GGLLHDPPSEIAK---DGRLEALLDECTNPKKRY-G---------RFQAAKELRGY
MdomeDIA1   379 ----GGLLHDPPGEIAK---DGRLEALLDECAHPKKRF-G---------RFQAAKELREY
GgallDIA1   378 ----GGLLHDPPADIAK---DGRLEALLDECANPKKRY-G---------RFQAAKELREY
XtropDIA1   378 ----GGLLHDPPAEIAK---DGRLEALLDECANPKKRY-G---------RFKSAKELREY
SpurpDIA1   369 ----GGLLHSIPSDLSSY-MRAKVKGQIAECRSPSKRD-G---------RYLVVKELKET
RnorvDIA1R  375 -----KFPSPVQQEIDS---------ALSLCSKDASTNLE---------VFGATSLLKNI
MmuscDIA1R  375 -----KFPSPVQKEIDS---------ALSLCSKDNSTDLE---------VLGATSWLKDI
MmulaDIA1R  373 -----RFPSPVQDDIDS---------ALAQCGDSTRPDPE---------VLGAASWLKHI
HsapiDIA1R  373 -----RFPSPVQDDIDS---------ILVQCGDSIRPDPE---------VLGAASQLKDI
BtaurDIA1R  373 -----KFPSPVQEEIDA---------ELTRCADGTRPDPE---------VLGAASRLKDI
EcabaDIA1R  373 -----KFPSPVQEEIDA---------TLTWCGEDTRPDAE---------VLGAASRLKDI
DordiDIA1R  374 -----KFPSPVQVEIDS---------ALAQCGQGIRPDSE---------VFGAASRLKDI
MdomeDIA1R  372 -----KFPQPIQEQIDS---------ALVLCGNSSLSDQE---------VIEAASRLKAI
OanatDIA1R  372 -----KFPKPVQEEIDA---------LLDRCGDSSLDGQT---------VIGGARRLMDI
GgallDIA1R  370 -----KFLPPVQEKIDS---------FLQHCAEGLADDQD---------VNEAMAKLAQL
DreriDIA1R  357 -----DKQSGLPRAAVD---------ELLVCADPSQLDQT---------IIKSVQSLKNI
SsalaDIA1R  383 ----QTTRLPMEGEAEAGRLARDVPLLLGVCADPSQPDWR---------IMAAVGSLMDL
NvitrDIA1   353 ----QGFLHDPPDYILQK--HPTLIDLLEQCAKPDIGY-S---------RIDIAHKLIVL
BflorDIA1   368 ----GGLLHDPPETVVR---DGTLQRLLAECAKPRTLYG----------RFDAAKELIEL
NvectDIA1   358 -----GLLHNPPVALKAS-----LNQLLQDCTAQDMRN-T---------REETAQKLSEL
CinteDIA1   385 K-YPGGLLHDPKEFGVFS---SQMFVLLDICSRGKTAVAPERRTLSHSIRRLAANQLRNV
consensus   421      gvlhe p di        l  ll  Ca p k             rfqaakdlrei
```

```
AaegyDIA1    387 IHDVLHEVV----------------------------
CpipiDIA1    393 IDGILVES-----------------------------
AgambDIA1    405 IDQTIIQS-----------------------------
DyakuDIA1    341 LDSKN--------------------------------
DerecDIA1    342 LDSKI--------------------------------
DmelaDIA1    344 LESNN--------------------------------
DsechDIA1    346 LESKN--------------------------------
DananDIA1    345 LDS----------------------------------
DpersDIA1    345 LGELLGS------------------------------
DpseuDIA1    345 LGELLGS------------------------------
DwillDIA1    335 LVEINNKIEIL--------------------------
GaculDIA1    421 LTQLAAASSSATAR-----------------------
OlatiDIA1    421 LTKLAAASSSATSR-----------------------
TnigrDIA1    421 LTQLA--------------------------------
TrubrDIA1    421 LTQLAASSSSLVSAR----------------------
DreriDIA1b   421 LTQLSGSAR----------------------------
DreriDIA1a   421 LTQLTQTSNADR-------------------------
BtaurDIA1    422 LAQLSNNVR----------------------------
PpygmDIA1    422 LAQLSNNVR----------------------------
CfamiDIA1    422 LAQLSNNVR----------------------------
TtrunDIA1    422 LAQLSNNVR----------------------------
PtrogDIA1    422 LAQLSNNVR----------------------------
HsapiDIA1    422 LAQLSNNVR----------------------------
MmulaDIA1    422 LAQLSNNVR----------------------------
PvampDIA1    422 LAQLSNNVR----------------------------
MmuscDIA1    422 LAQLSHNVR----------------------------
RnorvDIA1    422 LAQLSHNVR----------------------------
MdomeDIA1    422 LAQLSNNGR----------------------------
GgallDIA1    421 LAQLSNNVR----------------------------
XtropDIA1    421 LAQLSNNAR----------------------------
SpurpDIA1    414 LMKLIT-------------------------------
RnorvDIA1R   412 LRSLRTCDPRFAYRYPDCKYNDRF-------------
MmuscDIA1R   412 LRSLRTCDPRFAYRYPDCKYNDRF-------------
MmulaDIA1R   410 LRPLRTCDSRFAYRYPDCKYNDKF-------------
HsapiDIA1R   410 LRPLRTCDSRFAYRYPDCKYNDKF-------------
BtaurDIA1R   410 LRPLRTCDPRFAYRYPDCKYDDKF-------------
EcabaDIA1R   410 LRPLRTCDPRFAYRYPDCKYNDKF-------------
DordiDIA1R   411 LRPLRTCDPRFAYRYPDCKYNDRF-------------
MdomeDIA1R   409 LKPLRTCDPRYAYRYPDCKYSDRY-------------
OanatDIA1R   409 LKSLRTCDARFAYRYPDCKYSEKY-------------
GgallDIA1R   407 LKPLRSCDSRFAYRYPDCKYSDKY-------------
DreriDIA1R   394 LKTLRPCSPQYAYRYPECLYSDKF-------------
SsalaDIA1R   430 LKPMRPCNPHYTYRYPECRYNQDY-------------
NvitrDIA1    397 LDSVIKNA-----------------------------
BflorDIA1    411 LGSFLKDR-----------------------------
NvectDIA1    398 LQGYL--------------------------------
CinteDIA1    441 LERRRICSPDFKYRYPECELAEDAGINLPSIELQWLH
consensus    481 l  l
```
