# Supplementary material for: Characterization of the Deleted in Autism 1 Protein Family: Implications for Studying Cognitive Disorders
Source: PLoS One. 2011 Jan 19;6(1):e14547. doi: 10.1371/journal.pone.0014547 (PMC3023760; doi:10.1371/journal.pone.0014547)
Supplement: Figure S5 — Signal peptide localization in DIA1 and DIA1R proteins. The sequence alignment and consensus sequence of the amino terminal region of all full-length DIA1 and DIA1R proteins is from Figure S4. Abbreviations are as in Figure S4. DIA1R proteins were grouped together in the top portion of the figure, with aligned DIA1 proteins below, where arthropod sequences are placed above, nonvertebrate/nonarthropod sequences below, and vertebrate DIA1 sequences in the middle (see annotation on right-hand side of alignment). Initiation methiones were not manually aligned. Bold red amino acids represent the last amino acid of the amino-terminal signal peptide predicted using the NN algorithm [51]. Bold blue amino acids represent the last amino acid of the signal peptide predicted using the HMM prediction method [51]. Bold purple amino acids represent the last amino acid of the signal peptide predicted by both NN and HMM prediction methods. Bold underlined amino acids represent the last amino acid of the signal peptide predicted by the Sigcleave algorithm [52]. Lack of an underlined residue indicates no signal peptide cleavage site was predicted within the aligned region by Sigcleave, and lack of a red residue (or purple) indicates no signal peptide cleavage site predicted by the NN algorithm. The most commonly predicted site for cleavage of DIA1R signal peptides or vertebrate and arthropod DIA1 signal peptides are indicated with arrows above the alignment. (0.03 MB PDF) [file pone.0014547.s015.pdf]

Fig. S5

```
                                              DIA1R         DIA1
                                                       Vertebrate  Arthropod
                                                 ↓          ↓  ↓
RnorvDIA1R     1 MELGRPGAAATAFRQRWP-AWMLLWVSTLSCSFSLPASLPPSLVPRVRSSYTLGKTFLGL
MmuscDIA1R     1 MESQWRGAAATAFHQHWL-ARLLLWVSTLSCSFSLPASLPPSLVPRVRSSYTMGKTFLGL
MmulaDIA1R     1 MEP-QLGPEAAALRPGWL-A-LLLWVSALSCSFSLPASSPSSLVSQVRTSYNFGRTFLGL
HsapiDIA1R     1 MEP-QLGPEAAALRPGWL-A-LLLWVSALSCSFSLPASSLSSLVPQVRTSYNFGRTFLGL
BtaurDIA1R     1 MEP-RLGPKAAALHLGWP-F-LLLWVSGLSYSVSSPASPSPSPVSRVRTSYNLGKTFLGL
EcabaDIA1R     1 MEP-WLGPEAAALRPGWP-A-LLLWVSALRCSVSSPASPSPSLVPRVRTSYNFGRTFLGL
DordiDIA1R     1 MDP-QLGPEAAALHPGWQ-V-LLLLVVSVLSCFSSPASPPPSLVSRVRTSYNLGRTFLGL
MdomeDIA1R     1 MES-VIHGPLASPCLGWL-T--LLQLVTLSCISSTESLTAPSSVPRVKSSYNFGRTFLGL
OanatDIA1R     1 MGL-RLGCRLFSAALSWM-T--LLQFLTRGHGPVADASPAPSLVPRVKPSYSFGRTFLGL
GgallDIA1R     1 MGR--WICCLCSRVADWL-M--LLLVLARSSNPSAAATASPS-APHVRPSYSFGRTFLGL
DreriDIA1R     1 ---------MAG--IWS--G-VWILCFILVFGTADP-----SPAPQ-DKSHDFRKIFLGL
SsalaDIA1R     1 MPLKRCDQRAVG-SVRLT-VCVLSWLLCVCWAPSVVEPAGTPPAPQ-QKALILQRAFLGL
AaegyDIA1      1 --------------MSQ--HRILTKLHLVALFLWIVMKYQPEERASSLKSLVQQCQYDTT
CpipiDIA1      1 --------------MSSGFQRILVNLHILALLFCVIFEFQPKEPESLTELLVNQCEYETQ
AgambDIA1      1 --------------MQS----S-RLPYFVTLFIAVVLQYLPNEKSTSKRPLENVCDYDRN
DyakuDIA1      1 -----------------------MHLSPGQLKVVLILALLQELQVKPQREVFQELFEKDL
DerecDIA1      1 -----------------------MHLSPGQLKVVLILALLQELQVKPQREVFQELYEKDL
DmelaDIA1      1 -----------------------MHLSHGQLKVVLILALLQELQVKPQREVFQELFEKDL
DsechDIA1      1 -----------------------MHLSYGQLKVVLILALLQELQVKTQREVFQELFEKDL
DananDIA1      1 -----------------------MHLLSGQLRIVLVLALLQDLQVKPQKDIFQEHFESDL
DpersDIA1      1 -----------------------MHLSPGHLRIVLILALLQDLQVKPQKDIFRAHLERDL
DpseuDIA1      1 -----------------------MHLSPGHLRIVLILALLQDLQVKPQKDIFRAHLERDL
DwillDIA1      1 -----------------------MHLLYGQLKVVLVLALLQELDVRTQKDIFRKHFNMDL
NvitrDIA1      1 -----------------------MLVTKASVSSFLIALLILILGIYINRFNLKVAEITER
GaculDIA1      1 ------MLRFLPLKLGR--LYRCLKLLLVVGLFVILLMNTHSLFASFQKNELTDRRFINL
OlatiDIA1      1 ------MLRFLPLKLGR--LYRCLKLLFLVGLFVILLMNTHNLFASFQKNELTDRRFINL
TnigrDIA1      1 ------MLRFLPLKLGR--LYRCLKLLLVVGLFVILLMNTHNLFASFQKNELTDRRFINL
TrubrDIA1      1 ------MLRFLPLKLGR--LYRCLKLLLVVGLFVILLMNTHNLFASFQKNELTDRRFINL
DreriDIA1b     1 ------MLRFLPLKLGR--LYRCLKLLFLLGLFVILLMNTHNLFASFQKNELTDRRFINL
DreriDIA1a     1 ------MLRILSLKFGR--VYRCGKFLFIVALFVILLMNTHNLFASFQRNELTDRRFIGL
BtaurDIA1      1 ------MWRLVPPKLGR--LSRSLKLAALGSLLVLMVLHSPSLLASWQRNELADRRFLQL
PpygmDIA1      1 ------MWRLVPPKLGR--LSRSLKLAALGSLLVLMVLHSPSLLASWQRNELADRRFLQL
CfamiDIA1      1 ------MWRLVPPKLGR--LSRSLKLAALGSLLVLMVLHSPSLLASWQRNELADRRFLQL
TtrunDIA1      1 ------MWRLVPPKLGR--LSRSLKLAALGSLLVLMVLHSPSLLASWQRNELADRRFLQL
PtrogDIA1      1 ------MWRLVPPKLGR--LSRSLKLAALGSLLVLMVLHSPSLLASWQRNELADRRFLQL
HsapiDIA1      1 ------MWRLVPPKLGR--LSRSLKLAALGSLLVLMVLHSPSLLASWQRNELTDRRFLQL
MmulaDIA1      1 ------MWRLVPPKLGR--LSRSLKLAALGSLLVLMVLHSPSLLASWQRNELTDRRFLQL
PvampDIA1      1 ------MWRLVPPKLGR--LSRSLKLAALGSLLVLMVLHSPSLLASWQRNELADRRFLQL
MmuscDIA1      1 ------MWRLVPLKLGR--LSRALKLAALGSLLVLMLLHSPSLLASWQRNELADRRFLQL
RnorvDIA1      1 ------MWRLVPLKLGR--LSRALKLAALGSLLVLMLLHSPSLLASWQRNELADRRFLQL
MdomeDIA1      1 ------MWRLVPPKLGR--LSRSLKLAALGSLLVLMVLHSPSLLASWQRNELADRRFLQL
GgallDIA1      1 ------MLRLVSLKLGR--LYRYVKLAVLGSLAAALVLNTHSLLASLQRNELAERRFLQL
XtropDIA1      1 ------MLRLASLKFGR--LFRYAKVLFAASLLVVMLLNTHSLLSSFQRNELTDRRFLSL
SpurpDIA1      1 ------MRRYFNQFTNRFCISTTQQLIWLVVLSSMCLFVYYQLYLHFTANHLENEYFTEA
BflorDIA1      1 -------MRLVRLRQFRR-FFSGWRVRRMCGFFFFVVFFYCFVFQPFSYNQLTDTTFLGE
NvectDIA1      1 --MHFGGILRKRRYICRLVLLLIVIISFSDLLLWLSYQDGRAIKWDFSSQHKLTFQEIGL
CinteDIA1      1 -MLLKMKRYLTVWENGKYIVFIFILITLEVLLEMYNHRSQGSTRVMLEAKLLSDKWSATS
consensus      1         r    kmg   l   lkl  v  llvlvm    sl    qk  v er fl l
```

DIA1R

Arthropod DIA1

Vertebrate DIA1

Non-vertebrate/ Non-arthropod DIA1
