# Supplementary material for: Characterization of the Deleted in Autism 1 Protein Family: Implications for Studying Cognitive Disorders
Source: PLoS One. 2011 Jan 19;6(1):e14547. doi: 10.1371/journal.pone.0014547 (PMC3023760; doi:10.1371/journal.pone.0014547)
Supplement: Figure S6 — Amino acid sequence comparison of DIA1L proteins. A single DIA1L protein from S. purpuratus (SpurpDIA1L) and three DIA1L paralogues from B. floridae (BflorDIA1La, BflorDIA1Lb, BflorDIA1Lc) were aligned using CLUSTALW [47]. Identical amino acids shared between all four proteins are highlighted in red font and indicated below the alignment with a red asterisk (*). Strongly similar amino acids are highlighted in green font and indicated below the alignment with a green colon (:). Weakly similar amino acids are highlighted in blue font and indicated below the alignment with a blue full stop (.). Dissimilar amino acids are in black font. Amino acid numbering is provided above the alignment. Gaps are indicated by dashes. The alignment shows 7% identical amino acids, with a further 20% similar amino acids, providing an overall similarity of 27%. Amino acid similarity and identity shared at the amino-terminal ends of the three longer DIA1L proteins are indicated using the coloured fonts described above, but the corresponding annotations (*, : and .) are in grey font below the alignment. Standard single-letter amino acid abbreviations are used. Accession numbers can be found in Table S8. (0.02 MB PDF) [file pone.0014547.s016.pdf]

Fig. S6

```
                      10        20        30        40        50        60        70        80        90       100
                       |         |         |         |         |         |         |         |         |         |
BflorDIA1Lb   MASARRWCRRLHYSGWTRVLFLLTLMIAVGFIARNYWQVEDDLEARKRISNGGDKKYAQSIQELLAQGIDEKRMREALRQKAEEVHRIVKERGEALAKEA
BflorDIA1Lc   ----------------------------------------------------------------------------------------------------
BflorDIA1La   --------------------MPILVESEAGYGGR---------HGRAMVARRMVRKCCLPLLVLVSATLAVLYIYLVQVQGSPWVSLGQSQAGNKQKKDE
SpurpDIA1L    ---MGPRIRKKILILGSSILFTIFTVQLTGLNSL---------RD--DDNNALPSTNGKFRTFVPSKGDRKRNLLHAGKQGKEELGFDSKD----TRRTL
                                    :     .              .      .    .       : :       :  .  *    :    .:      :

                     110       120       130       140       150       160       170       180       190       200
                       |         |         |         |         |         |         |         |         |         |
BflorDIA1Lb   QAKAKAKANVVRINIHKPELNPENQHSWKAIEKMAAHDEIEDHKKQPEQYMTFKRLMDAKKCPACYGESLCEQAEVG-LITMDVADKTLEHKG-VYFGHF
BflorDIA1Lc   ------------------------------MVIADGDNDIDISWAEPE------------KCPACFGD-KCELLRRGHFVKVDSESDRYMKKGIVSTGRI
BflorDIA1La   YIVWRGEEQKVEVQQKQ----EEKKHPAEEPKEKPRQDKSAVTERKPKG--VPRSLLRNAPCPACLGDNLCEEFEDG---MIDLGSEVTSWKVKAYTGTW
SpurpDIA1L    QDIRTADEIKGHPRIQAG---DETALTQDGVQGMKNATVVMTTRLQFEN--LFPSLLEEERCPLCYGTTNCDQIYAG-NISFHVGSVNLSEPH-AIRGTW
                   ..    . . :      *.  . .                : :       *:    ** * *   *:    *    ..  .        .  *

                     210       220       230       240       250       260       270       280       290       300
                       |         |         |         |         |         |         |         |         |         |
BflorDIA1Lb   RNTEVVAKRLVGKDGWTRFDEFICQNASLPKDCDVSHMISDTVLVTDNVLQVSFLQDAWRIAHTRRSI--AMEACMTDRLIELIKTAYDENVNGKLSK--
BflorDIA1Lc   GGVKVIAKSMNEAGAWQRYERFICRSSSRPHVCNASSFILETMLVTDVALKVPWLREAWKICHLEKS---ALSLCVSDRFLEDVRQLYVEDGGTDMTKEG
BflorDIA1La   DKVEVMVTQCASEERLERFEEFVCRNLSETATSCDPGKVLLQEDLKERLQPAHLKKSLREVFHPNTTSSLQATTCMSKPFLKLLQKTFDDNGNKRLNR--
SpurpDIA1L    GDRRIVGKRLVSREVFERLEKIICNQSQVDPKRSCEVNAAATNSWMSKSSALNRVHKLHQEVYESQLIAISATTCASPDFFEELKQLYRVSRWGTGIAS-
                 .:: .         * :.::*.. .                  .        :.  .  :           * :  ::: ::  :  .

                     310       320       330       340       350       360       370       380       390       400
                       |         |         |         |         |         |         |         |         |         |
BflorDIA1Lb   -TERAYMITALLLNPEAALLKHFTSRAEEYWPFPKYLGACGRVILVESGGKLLGSAIESP--WKERANIALQLLEMIDKFRNGDPKWVVIFVDFSFNNFA
BflorDIA1Lc   DTGRAFLSTSLLLNEEAVLLRYFTTKSTTPWPFPKFYGACGRVIVVEHAGRTLDTFMESS--WEVRADIALQLLQLVDALREKDPDWVLFFLDVSFQNFA
BflorDIA1La   ------------LE---------QAIPRKKLPIPAFLGACGNLVATETAGKPLSMYLEVKGPWQVRANLSLQLLQMLDDFQNKDPDWLLMFVEVNIENFS
SpurpDIA1L    -TEMAILATTMALNPEPALLKFFRNIPSLRPYFTEYLGECGRVILTEPSGKPLSSYLKAS--WKDRVDISLKILQMIEDFHDSSDKWLVLLLDFGYENFV
                          *:            .     :. : * **.:: .* .*: *.  ::    *: *.:::*::*:::: ::: . .*:::::::.. :**

                     410       420       430       440       450       460       470       480       490       500
                       |         |         |         |         |         |         |         |         |         |
BflorDIA1Lb   VNNYGRLTLIDFDDVMLIDREEFVG--------ENKTEPCDLKCFKTFISQIEAMNSYDSCSAMPQYSQMMYALACVRLLSHLPEHLSEP--NPMDPKPD
BflorDIA1Lc   VDSRGWVRLIDLDDVMVIDRRTVVN--------QEQTEMCNEQCYTDFQKKLYS-DEYH-CDDIFKYAPMMYASICARLLSNLQKHPERRKWGEIREYQE
BflorDIA1La   VSSDGRLILTDLGNMTIINKHDLDKN-----STKKRSSVCNEACFKRFTRNLSHRPETS-CRQAGRYSQLMYARACQRILGCWQTERSGE--GRVVLSSS
SpurpDIA1L    MTSEGQLKVVNLGGMVIVDKDQTSTTPDMNPHLNNRTELCNEDCLNTFVKQLQTEPDTH-CREVPRHVELMYMMACHSLLSDLMTTKYER--FFQPLDTP
              : . * : : ::..: ::::             ::::. *:  * . *  ::    .   *    ::  :**   *  :*.

                     510       520       530       540       550       560       570
                       |         |         |         |         |         |         |
BflorDIA1Lb   KHRPDSERP---RLLWGPPEQEAKVLEELLRGCVEENVAGGRLEAVGELKTFLRRNAKGEN----------
BflorDIA1Lc   GQSIELDEPPVKGFLHNPPDEIREALEGALTQCVHETLPRGRLGAVLRLQEILGTT---------------
BflorDIA1La   KDASCAYGR---GLLFGPPREAKQELEDLLTECVEETKAGGRITALKQIRVLLAA----------------
SpurpDIA1L    R----KHHPG--GMLHDAPYEVDSVLSELLYECVFEGQPGRRMHSVRVLKRLLTIIQRGFSYKDAADRTSL
                           :* ..* :  . *.  *  ** *  .  *: ::  :: :*
```
