# Supplementary material for: Characterization of the Deleted in Autism 1 Protein Family: Implications for Studying Cognitive Disorders
Source: PLoS One. 2011 Jan 19;6(1):e14547. doi: 10.1371/journal.pone.0014547 (PMC3023760; doi:10.1371/journal.pone.0014547)
Supplement: Figure S7 — Amino acid sequence comparison of DIA1-family proteins. The sequence alignment of all full-length DIA1, DIA1R and DIA1L proteins was generated using CLUSTALW [47]. Identical amino acids are highlighted in red font and indicated below the alignment with a red asterisk (*). Strongly similar amino acids are highlighted in green font and indicated below the alignment with a green colon (:). Weakly similar amino acids are highlighted in blue font and indicated below the alignment with a blue full stop (.). Dissimilar amino acids are in black font. Amino acid numbering is provided above the alignment. Gaps are indicated by dashes. The alignment shows only 4 identical amino acids and 18 similar amino acids conserved across the entire protein family (3% similarity). Standard single-letter amino acid abbreviations are used. Organism abbreviations use the first letter of the genus name, followed by the first four letters of the species (e.g., Pteropus vampyrus DIA1 is abbreviated to PvampDIA1). Accession numbers and full species names can be found in Tables S1, S5 and S8. (0.05 MB PDF) [file pone.0014547.s017.pdf]

Fig. S7

```
                        10        20        30        40        50        60        70        80
                         |         |         |         |         |         |         |         |
SpurpDIA1     --------------------------------------------------------------------------------
NvectDIA1     --------------------------------------------------------------------------------
GaculDIA1     --------------------------------------------------------------------------------
OlatiDIA1     --------------------------------------------------------------------------------
TnigrDIA1     --------------------------------------------------------------------------------
TrubrDIA1     --------------------------------------------------------------------------------
DreriDIA1b    --------------------------------------------------------------------------------
DreriDIA1a    --------------------------------------------------------------------------------
BtaurDIA1     --------------------------------------------------------------------------------
PpygmDIA1     --------------------------------------------------------------------------------
CfamiDIA1     --------------------------------------------------------------------------------
PtrogDIA1     --------------------------------------------------------------------------------
TtrunDIA1     --------------------------------------------------------------------------------
HsapiDIA1     --------------------------------------------------------------------------------
MmulaDIA1     --------------------------------------------------------------------------------
PvampDIA1     --------------------------------------------------------------------------------
MdomeDIA1     --------------------------------------------------------------------------------
MmuscDIA1     --------------------------------------------------------------------------------
RnorvDIA1     --------------------------------------------------------------------------------
GgallDIA1     --------------------------------------------------------------------------------
XtropDIA1     --------------------------------------------------------------------------------
BflorDIA1     --------------------------------------------------------------------------------
SsalaDIA1R    --------------------------------------------------------------------------------
DreriDIA1R    --------------------------------------------------------------------------------
RnorvDIA1R    --------------------------------------------------------------------------------
MmuscDIA1R    --------------------------------------------------------------------------------
MmulaDIA1R    --------------------------------------------------------------------------------
HsapiDIA1R    --------------------------------------------------------------------------------
BtaurDIA1R    --------------------------------------------------------------------------------
EcabaDIA1R    --------------------------------------------------------------------------------
DordiDIA1R    --------------------------------------------------------------------------------
MdomeDIA1R    --------------------------------------------------------------------------------
OanatDIA1R    --------------------------------------------------------------------------------
GgallDIA1R    --------------------------------------------------------------------------------
BflorDIA1Lb   MASARRWCRRLHYSGWTRVLFLLTLMIAVGFIARNYWQVEDDLEARKRISNGGDKKYAQSIQELLAQGIDEKRMREALRQ
BflorDIA1Lc   --------------------------------------------------------------------------------
AaegyDIA1     --------------------------------------------------------------------------------
CpipiDIA1     --------------------------------------------------------------------------------
DyakuDIA1     --------------------------------------------------------------------------------
DerecDIA1     --------------------------------------------------------------------------------
DmelaDIA1     --------------------------------------------------------------------------------
DsechDIA1     --------------------------------------------------------------------------------
DpersDIA1     --------------------------------------------------------------------------------
DpseuDIA1     --------------------------------------------------------------------------------
DananDIA1     --------------------------------------------------------------------------------
DwillDIA1     --------------------------------------------------------------------------------
NvitrDIA1     --------------------------------------------------------------------------------
SpurpDIA1L    ---------------------MGPRIRKKILILGSSILFTIFTVQLTGLNSLRDDDNNALPSTNGKFRTFVPSKGDRKRN
BflorDIA1La   -----------------------------MPILVESEAGYGGRHGRAMVARRMVRKCCLPLLVLVSATLAVLYIYLVQVQ
CinteDIA1     --------------------------------------------------------------------------------
AgambDIA1     --------------------------------------------------------------------------------
```

```
                       90        100       110       120       130       140       150       160
                        |         |         |         |         |         |         |         |
SpurpDIA1     ---------MRRYFNQFTNRFCISTTQQLIWLVVLSSMCLFVYYQ----LYLHFTANHLENEYFTEAT------------
NvectDIA1     ---------MHFGGILRKRRYICRLVLLLIVIISFSDLLLWLSYQDGRAIKWDFSSQHKLTFQEIGLN------------
GaculDIA1     ----------MLRFLPL-KLGRLYRCLKLLLVVGLFVILLMNTHS----LFASFQKNELTDRRFINLN------------
OlatiDIA1     ----------MLRFLPL-KLGRLYRCLKLLFLVGLFVILLMNTHN----LFASFQKNELTDRRFINLN------------
TnigrDIA1     ----------MLRFLPL-KLGRLYRCLKLLLVVGLFVILLMNTHN----LFASFQKNELTDRRFINLN------------
TrubrDIA1     ----------MLRFLPL-KLGRLYRCLKLLLVVGLFVILLMNTHN----LFASFQKNELTDRRFINLN------------
DreriDIA1b    ----------MLRFLPL-KLGRLYRCLKLLFLLGLFVILLMNTHN----LFASFQKNELTDRRFINLN------------
DreriDIA1a    ----------MLRILSL-KFGRVYRCGKFLFIVALFVILLMNTHN----LFASFQRNELTDRRFIGLN------------
BtaurDIA1     ----------MWRLVPP-KLGRLSRSLKLAALGSLLVLMVLHSPS----LLASWQRNELADRRFLQLN------------
PpygmDIA1     ----------MWRLVPP-KLGRLSRSLKLAALGSLLVLMVLHSPS----LLASWQRNELADRRFLQLN------------
CfamiDIA1     ----------MWRLVPP-KLGRLSRSLKLAALGSLLVLMVLHSPS----LLASWQRNELADRRFLQLN------------
PtrogDIA1     ----------MWRLVPP-KLGRLSRSLKLAALGSLLVLMVLHSPS----LLASWQRNELADRRFLQLN------------
TtrunDIA1     ----------MWRLVPP-KLGRLSRSLKLAALGSLLVLMVLHSPS----LLASWQRNELADRRFLQLN------------
HsapiDIA1     ----------MWRLVPP-KLGRLSRSLKLAALGSLLVLMVLHSPS----LLASWQRNELTDRRFLQLN------------
MmulaDIA1     ----------MWRLVPP-KLGRLSRSLKLAALGSLLVLMVLHSPS----LLASWQRNELTDRRFLQLN------------
PvampDIA1     ----------MWRLVPP-KLGRLSRSLKLAALGSLLVLMVLHSPS----LLASWQRNELADRRFLQLN------------
MdomeDIA1     ----------MWRLVPP-KLGRLSRSLKLAALGSLLVLMVLHSPS----LLASWQRNELADRRFLQLN------------
MmuscDIA1     ----------MWRLVPL-KLGRLSRALKLAALGSLLVLMLLHSPS----LLASWQRNELADRRFLQLN------------
RnorvDIA1     ----------MWRLVPL-KLGRLSRALKLAALGSLLVLMLLHSPS----LLASWQRNELADRRFLQLN------------
GgallDIA1     ----------MLRLVSL-KLGRLYRYVKLAVLGSLAAALVLNTHS----LLASLQRNELAERRFLQLN------------
XtropDIA1     ----------MLRLASL-KFGRLFRYAKVLFAASLLVVMLLNTHS----LLSSFQRNELTDRRFLSLN------------
BflorDIA1     -----------MRLVRLRQFRRFFSGWRVRRMCGFFFFVVFFYCF----VFQPFSYNQLTDTTFLGED------------
SsalaDIA1R    ----MPL--KRCDQRAVGSVRLT--VCVLSWLLCVCWAPSVVEPAGT--PPAPQQKALILQRAFLGLD------------
DreriDIA1R    ----------------MAG------IWSGVWILCFILVFGTADPS-----PAPQDKSHDFRKIFLGLD------------
RnorvDIA1R    ----MELGRPGAAATAFRQRWPAWMLLWVSTLSCSFSLPASLPPSL----VPRVRSSYTLGKTFLGLD------------
MmuscDIA1R    ----MESQWRGAAATAFHQHWLARLLLWVSTLSCSFSLPASLPPSL----VPRVRSSYTMGKTFLGLD------------
MmulaDIA1R    ----MEP-QLGPEAAALRPGWLA-LLLWVSALSCSFSLPASSPSSL----VSQVRTSYNFGRTFLGLD------------
HsapiDIA1R    ----MEP-QLGPEAAALRPGWLA-LLLWVSALSCSFSLPASSLSSL----VPQVRTSYNFGRTFLGLD------------
BtaurDIA1R    ----MEP-RLGPKAAALHLGWPF-LLLWVSGLSYSVSSPASPSPSP----VSRVRTSYNLGKTFLGLD------------
EcabaDIA1R    ----MEP-WLGPEAAALRPGWPA-LLLWVSALRCSVSSPASPSPSL----VPRVRTSYNFGRTFLGLD------------
DordiDIA1R    ----MDP-QLGPEAAALHPGWQV-LLLLVVSVLSCFSSPASPPPSL----VSRVRTSYNLGRTFLGLD------------
MdomeDIA1R    ----MES-VIHGPLASPCLGWLT--LLQLVTLSCISSTESLTAPSS----VPRVKSSYNFGRTFLGLD------------
OanatDIA1R    ----MGL-RLGCRLFSAALSWMT--LLQFLTRGHGPVADASPAPSL----VPRVKPSYSFGRTFLGLD------------
GgallDIA1R    ----MGR--WICCLCSRVADWLM--LLLVLARSSNPSAAATASPS-----APHVRPSYSFGRTFLGLD------------
BflorDIA1Lb   KAEEVHRIVKERGEALAKEAQAKAKAKANVVRINIHKPELNPENQHSWKAIEKMAAHDEIEDHKKQPEQYMTFKRLMDAK
BflorDIA1Lc   --------------------------------------------------MVIADGDNDIDISWAEPE------------
AaegyDIA1     ----------------MSQHRILTKLHLVALFLWIVMKYQPEERA-------SSLKSLVQQCQYDTTI------------
CpipiDIA1     --------------MSSGFQRILVNLHILALLFCVIFEFQPKEPE-------SLTELLVNQCEYETQH------------
DyakuDIA1     -------------------MHLSPGQLKVVLILALLQELQVKPQ-----------REVFQELFEKDLR------------
DerecDIA1     -------------------MHLSPGQLKVVLILALLQELQVKPQ-----------REVFQELYEKDLR------------
DmelaDIA1     -------------------MHLSHGQLKVVLILALLQELQVKPQ-----------REVFQELFEKDLR------------
DsechDIA1     -------------------MHLSYGQLKVVLILALLQELQVKTQ-----------REVFQELFEKDLR------------
DpersDIA1     -------------------MHLSPGHLRIVLILALLQDLQVKPQ-----------KDIFRAHLERDLQ------------
DpseuDIA1     -------------------MHLSPGHLRIVLILALLQDLQVKPQ-----------KDIFRAHLERDLQ------------
DananDIA1     -------------------MHLLSGQLRIVLVLALLQDLQVKPQ-----------KDIFQEHFESDLR------------
DwillDIA1     -------------------MHLLYGQLKVVLVLALLQELDVRTQ-----------KDIFRKHFNMDLL------------
NvitrDIA1     --------------------MLVTKASVSSFLIALLILILGIYI-----------NRFNLKVAEITER-----------Y
SpurpDIA1L    LLHAGKQGKEELGFDSKDTRRTLQDIRTADEIKGHPRIQAGDETALTQDGVQGMKNATVVMTTRLQFEN--LFPSLLEEE
BflorDIA1La   GSPWVSLGQSQAGNKQKKDEYIVWRGEEQKVEVQQKQEEKKHPAEEP-KEKPRQDKSAVTERKPKGVPRSLLRN-----A
CinteDIA1     ---------MLLKMKRYLTVWENGKYIVFIFILITLEVLLEMYNHRSQGSTRVMLEAKLLSDKWSATSP---------TC
AgambDIA1     -------------------MQSSRLPYFVTLFIAVVLQYLPNEKS--------TSKRPLENVCDYDRN-----------I
```

```
                     170       180       190       200       210       220       230       240
                       |         |         |         |         |         |         |         |
SpurpDIA1     KCPACFGT--SLCKRFSRGDYRFHSYSSIR-------LFDYVN-VKNVYFATYLD----QKP-VVMKKLGHNSEHNQFDK
NvectDIA1     KCPACFGINLSICHKLLGGGVTVKSN---T-------PWSEER-IKGVSYGLWGA----VKQRVVLKTLGTSSELTAFDN
GaculDIA1     KCPACFGT--SWCRKFMNGQVSFETWGRLR-------FLDVFN-VKNVYFAQYGEPREGTRR-VVLKRLGSNQELAEIDQ
OlatiDIA1     KCPACFGT--SWCRKFMNGQVSFETWGRLR-------FLDVFN-VKNVYFAQYGEPREGTRR-VVLKRLGSNQELAEIDQ
TnigrDIA1     KCPACFGT--SWCRKFMNGQVSFETWGRLR-------FLDVFN-VKNVYFAQYGEPREGTRR-VVLKRLGSNQELADIDQ
TrubrDIA1     KCPACFGT--SWCRKFMNGQVSFETWGRLR-------FLDVFN-VKNVYFAQYGEPREGTRR-VVLKRLGSNQELADIDQ
DreriDIA1b    KCPACFGT--SWCRKFMNGQISFETWGRLR-------FLDVFN-VKNVFFAQYGEPREGTRR-IVLKRLGSNQELADIDQ
DreriDIA1a    KCPACFGT--SWCRKFMNGQVTFEMWGRLR-------FLDFFN-VKNVFFAQYGEPREGTRR-VVLKRLGSNQELAEIDQ
BtaurDIA1     KCPACFGT--SWCRRFLNGQVVFEAWGRLR-------LLDFLN-VKNVYFAQYGEPREGGRRRVVLKRLGSQRELAQLDQ
PpygmDIA1     KCPACFGT--SWCRRFLNGQVVFEAWGRLR-------LLDFLN-VKNVYFAQYGEPREGGRRRVVLKRLGSQRELAQLDQ
CfamiDIA1     KCPACFGT--SWCRRFLNGQVVFEAWGRLR-------LLDFLN-VKNVYFAQYGEPREGGRRRVVLKRLGSQRELAQLDQ
PtrogDIA1     KCPACFGT--SWCRRFLNGQVVFEAWGRLR-------LLDFLN-VKNVYFAQYGEPREGGRRRVVLKRLGSQRELAQLDQ
TtrunDIA1     KCPACFGT--SWCRRFLNGQVVFEAWGRLR-------LLDFLN-VKNVYFAQYGEPREGGRRRVVLKRLGSQRELAQLDQ
HsapiDIA1     KCPACFGT--SWCRRFLNGQVVFEAWGRLR-------LLDFLN-VKNVYFAQYGEPREGGRRRVVLKRLGSQRELAQLDQ
MmulaDIA1     KCPACFGT--SWCRRFLNGQVVFEAWGRLR-------LLDFLN-VKNVYFAQYGEPREGGRRRVVLKRLGSQRELAQLDQ
PvampDIA1     KCPACFGT--SWCRRFLNGQVVFEAWGRLR-------LLDFLN-VKNVYFAQYGEPREGGRRRVVLKRLGSQRELAQLDQ
MdomeDIA1     KCPACFGT--SWCRRFLNGQVAFEAWGRLR-------LLDFLN-VKNVYFGQYGEPREGGRRRVVLKRLGSQRELAQLDQ
MmuscDIA1     KCPACFGT--SWCRRFLNGQVGFETWGRLR-------LLDFLN-VKNVYFAQYGEPREGGRRRVVLKRLGSQRELAQLDQ
RnorvDIA1     KCPACFGT--SWCRRFLNGQVGFETWGRLR-------LLDFLN-VKNVYFAQYGEPREGGRRRVVLKRLGSQRELAQLDQ
GgallDIA1     KCPACWGT--SWCRKFLNGQLRLESWGRLR-------LFDFFN-VKNVYFARYGEPREGSRR-VVLKRLGSAQELADIDA
XtropDIA1     KCPACFGT--SWCRKFMNGQLSFEGWGRLR-------LLDFFN-VKNVHFAQYGEPREGSRR-VVLKRLGSNHELSELDQ
BflorDIA1     KCPACFGT--DLCEEFENGKILFKYSSRLR-------ILDIFN-IKNVYFAIY----EGME--VALKRLGHNSEFDQLDR
SsalaDIA1R    KCNACVGT--SICKKLLKDQIRFDWWMSPD-------TTLPLA-EKQSFPGNLTDDSLSWRP-VVLSFLSSPCLHSSSDR
DreriDIA1R    KCNACIGT--SICKKFFKDEIRFERRLTAQ-------SNLSSA-DVRSYEGNYTDSTAGWRP-VVVSRLMSPHLHQLSDN
RnorvDIA1R    KCNACIGT--SICKKFFKEEIRLDNSLVSH-------LGLPSQ-DLLSYAANYSDDSKTWRP-VEISRLVSKYQSEISDK
MmuscDIA1R    KCNACIGT--SICKKFFKEEIRFDNSLASH-------LGLPPQ-DLHSYAANYSDDSKTWRP-VEISQLVSRYQIEISDR
MmulaDIA1R    KCNACIGT--SICKKFFKEEIRSDNWLASH-------LGLPPD-FLLSYPANYSDDSKIWRP-VEIFRLVSNYQNEISDR
HsapiDIA1R    KCNACIGT--SICKKFFKEEIRSDNWLASH-------LGLPPD-SLLSYPANYSDDSKIWRP-VEIFRLVSKYQNEISDR
BtaurDIA1R    KCNACIGT--SICKKFFKEEIRFDNGLALH-------LGPPPD-DLPSYSANYSDDFKTWRP-VEISRLVSKQQNKISDG
EcabaDIA1R    KCNACIGT--SICKKFFKEEIRFDNRLASH-------LGLPSG-YLPSYSANYSDDSKTWRP-VEVSRLVSKQQNEISDR
DordiDIA1R    KCNACIGT--SICKKFFKEEIRFDNSLASD-------LELASD-YLPSYSANYSDDSKTWRP-VEIFRLVSKEQNEISDR
MdomeDIA1R    KCNACIGT--SICKKFFKEEIRFDNWLASH-------LKLPPD-YFSTYPANYSDDTKSWRP-VEISRLISKYQHELSDR
OanatDIA1R    KCNACIGT--SICKKFFKEEIRFDNWPASH-------LKPPPD-HFPGYSANYTDDSKSWRL-VEISRLIGKDQNELSDQ
GgallDIA1R    KCNACIGT--SICKKFFKEEIRFDTWLSSH-------LKLPPS-YLLSYLGNYTDDAQSWRM-VDITRLTTKYQHDRADQ
BflorDIA1Lb   KCPACYGES--LCEQAEVG-LITMDVADKT-------LEHKG--VYFGHFRNT-----E----VVAKRLVGKDGWTRFDE
BflorDIA1Lc   KCPACFGD---KCELLRRGHFVKVDSESDR-------YMKKGI-VSTGRIGGV-----K----VIAKSMNEAGAWQRYER
AaegyDIA1     LCPGCFPRP-VNCSFFENLKSIDS----NE-------LFEELL-THFNQHGVRYGTLSGSEQRVVIKNLNKEHGVEELIS
CpipiDIA1     LCPECFVSPTVNCGHLANFVTIDS----SN-------PWNQLT-CRYNPHGVSYG-LLGSGQKVVIKTLNKNRAVEALRD
DyakuDIA1     LCPNCFFGQRELCGEIFQKIAEPSDW--SK-------LLKAIS-LLVDRRVIYFLRFKDQDQ-----VVAKRKIIDAQNQ
DerecDIA1     LCPDCFVGQSELCVGIFQKIAEPSDW--SK-------LLKAIS-LIVDRRAIYYLRLKDQAQ-----VVAKRKIIDARNH
DmelaDIA1     LCPDCFVGQSEPCGEIFQKIAEPSDW--SR-------LVKAIS-LLVDRRAIYFLRLKDRDQEV-Q-LVAKRKIIDAQSN
DsechDIA1     LCPDCFVGQKEQCGEIFQKIAEPSDW--SR-------LVKAIS-LLVDRRAIHYLRLKDQDQAV-Q-LVAKRKIINAHSY
DpersDIA1     LCPACFAGQRWQCEEIFEAIAEPSDW--SK-------LLKGIA-LLIDRREIYFLQLNERHQAA-Q-LVAKRDAIGGHSY
DpseuDIA1     LCPACFAGQRWQCEEIFEAIAEPSDW--SK-------LLKGIA-LLIDRREIYFLQLNERHQAA-Q-LVAKRDAIGGHSY
DananDIA1     LCPACFSAQREQCEEVFRAIREPSDL--SK-------LWKAVT-LLFDRREIFWLEIGPSQEKSSQ-LLAKRKSIVASHQ
DwillDIA1     ECPQCFLGQREQCEKFFDTIEEPAKL--DR-------LWKDLT-LLVDPREIYWLELNENREN-VK-CLAKRKMTRKISK
NvitrDIA1     KCPACFGD--SMCQVIDSNEISFEYTDFYS-------IFNNLFSVKNVYYGKYKD------KKVIMKKLAQESELKTFDE
SpurpDIA1L    RCPLCYGT--TNCDQIYAGNISFHVG---S-------VNLSEP---HAIRGTWGD------RRIVGKRLVSREVFERLEK
BflorDIA1La   PCPACLGD--NLCEEFEDGMIDLG--------------SEVTSWKVKAYTGTWD------KVEVMVTQCASEERLERFEE
CinteDIA1     GNFSCFGT--SLCKNRNRVGLSEDVPTLRSGWLSIYGELVDLHRVKLNVTYKLPSTLPCNGETCSVQIETSVLSGNNVNK
AgambDIA1     LCPECVSSG-LECNKFDDLFTLQDDN-----------VFNIIN-NIFNSHTTRIGTIVQENKLAVMKHLNRDNTIEKLLQ
                  *       *
```

```
                     250       260       270       280       290       300       310       320
                       |         |         |         |         |         |         |         |
SpurpDIA1      DLCTNAES-GLRGCDVSQQIYKSKLS------DIMYAERLEEKDVMG---L-SDIVRCPSRRLLDRIWDTFGERQFDKS-
NvectDIA1      EICRGVGK-DSN-CKISQEAWDS---------DVLVPKPPLQDAVR-------KYFDCPSTRFLEKIESVYG-QETPGQ-
GaculDIA1      KICKRATG-RPR-CDLIQAMYKTEFA------RINGDVRLLTPEVVEG--W-SDLVHCPSQRLLDRVVRRYAETKDSGSF
OlatiDIA1      KICKRATG-RPR-CDLVQAMYKTEFA------RINGDVRLLTPDVVEG--W-SDLVHCPSQRLLDRVVRRYAETKDSGSF
TnigrDIA1      KICKRATG-RPR-CDLIQAMYKTEFA------RINGDVRLLTPDVVEG--W-SDLVHCPSQRLLDRVVRRYAETKDSGSF
TrubrDIA1      KICKRATG-RPR-CDLIQAMYKTEFA------RINGDVRLLTPEVVEG--W-SDLVHCPSQRLLDRVVRRYAETKDSGSF
DreriDIA1b     KICKRATG-RPR-CDLIQAMYKTEFA------RLNGDVRLLTPDVVEG--W-SDLVHCPSQRLLDRIVRRYAETKDSGSF
DreriDIA1a     KICKRATG-RPR-CDLIQSMYKTEFA------RLNGDVRLLTPEVVEG--W-SDLVHCPSQRLLDRVVRRYAETKDSGSF
BtaurDIA1      SICKRATG-RPR-CDLLQAMPRTEFA------RLNGDVRLLTPEAVEG--W-SDLVHCPSQRLLDRLVRRYAETKDSGSF
PpygmDIA1      SICKRATG-RPR-CDLLQAMPRTEFA------RLNGDVRLLTPEAVEG--W-SDLVHCPSQRLLDRLVRRYAETKDSGSF
CfamiDIA1      SICKRATG-RPR-CDLLQAMPRTEFA------RLNGDVRLLTPEAVEG--W-SDLVHCPSQRLLDRLVRRYAETKDSGSF
PtrogDIA1      SICKRATG-RPR-CDLLQAMPRTEFA------RLNGDVRLLTPEAVEG--W-SDLVHCPSQRLLDRLVRRYAETKDSGSF
TtrunDIA1      SICKRATG-RPR-CDLLQAMPRTEFA------RLNGDVRLLTPEAVEG--W-SDLVHCPSQRLLDRLVRRYAETKDSGSF
HsapiDIA1      SICKRATG-RPR-CDLLQAMPRTEFA------RLNGDVRLLTPEAVEG--W-SDLVHCPSQRLLDRLVRRYAETKDSGSF
MmulaDIA1      SICKRATG-RPR-CDLLQAMPRTEFA------RLNGDVRLLTPEAVEG--W-SDLVHCPSQRLLDRLVRRYAETKDSGSF
PvampDIA1      SICKRATG-RPR-CDLLQAMPRTEFA------RLNGDVRLLTPEAVEG--W-SDLVHCPSQRLLDRLVRRYAETKDSGSF
MdomeDIA1      SICKRATG-RPR-CDLLQAMPKTEFA------RLNGDVRLLTPDAVEG--W-SDLVHCPSQRLLDRLVRRYAETKDSGSF
MmuscDIA1      SICKRATG-RPR-CDLLQAMPRTEFA------RLNGDVRLLTPEAVEG--W-SDLVHCPSQRLLDRLVRRYAETKDSGSF
RnorvDIA1      SICKRATG-RPR-CDLLQAMPRTEFA------RLNGDVRLLTPEAVEG--W-SDLVHCPSQRLLDRLVRRYAETKDSGSF
GgallDIA1      KICRRATG-RGR-CDLLQALHATEFA------SLNGDVRLLTPDAVEG--W-SDLVHCPSQRLLDRLVRRYAETKDSGSF
XtropDIA1      RICKKATG-RPR-CDLVQAMYKTDFA------RLNGDVRLLTPDVVEG--W-SDLVHCPSQRLLDRLVRRYAETKDSGSF
BflorDIA1      WICETGKE-GPQ-CHIPSVTYYSNFS------RVIYEG--LSPKSLGD--M-SDMVRCPSQRLIDRVLEKFAEHLGKET-
SsalaDIA1R     SICRSVGR-QGP-CSIEAVLRVTPRF------QSLNQSHLLLPHIVKG--LAPPLLRCPSQRLLDRIVRRYAEVVDVGSV
DreriDIA1R     SICTSAGK-GKS-CSIEGVLRATSRF------QTWVHSNLLLPSMVKG--LVTPMLRCPSQRLLDRIVRRYFEVTDVGSV
RnorvDIA1R     RICASASG-PKT-CSIERILRKTGRF------QKWLQAKRLTPDLVQG--LPSPFLHCPSQRLLDRVVRRYAEVVDAGSI
MmuscDIA1R     RICASVSA-PKT-CSIERILQKTGRF------QKWLQAKRLTPDLVQG--LPSPFLRCPSQRLLDRVVRRYAEVVDAGSI
MmulaDIA1R     RICASASA-PRT-CSIERVLRKTERF------QKWLQAKRLTPDLVQG--LASPLLRCPSQRLLDRVVRRYAEVADAGSI
HsapiDIA1R     RICASASA-PKT-CSIERVLRKTERF------QKWLQAKRLTPDLVQG--LASPLLRCPSQRLLDRVVRRYAEVADAGSI
BtaurDIA1R     RICASAAA-PKT-CSIERVLRKTGRF------QKWLQAKRLTPDLVRG--LSSPLLRCPSQRLLDRVVRRYAEVADAGSI
EcabaDIA1R     RICAFAAA-PKT-CSIERVLRKTRRF------QKWLQAKRLTPDLVRG--LPSPLLRCPSQRLLDRVVRRYAEVPDAGSI
DordiDIA1R     RICAFASA-PKT-CSIERILRKTGRF------QKWLQAKRLTPDLVQG--LPSPFLRCPSQRLLDRVVRRYAEVADAGSI
MdomeDIA1R     RICASASG-SKT-CSIERVLRKTERV------KKWLKAKRLTPDLVQG--LPSPMLRCPSQRLLDRIVRRYAEVADAGSV
OanatDIA1R     KICTSVSL-VKT-CSIERVLRKTERF------QKWLKARRLTPALVQG--LPSLLLRCPTQRLLDRIVRRYAEVPDAGSV
GgallDIA1R     HICTSLLK-TKT-CSLERALRRTHRF------QKWLRAKRLTPDLVQG--LSSPMLRCPSQRLLDRIVRRYAEVPDAGSI
BflorDIA1Lb    FICQNASL-PKD-CDVSHMISDTVLVTDNVLQVSFLQDAWRIAHTRRS--I--AMEACMTDRLIELIKTAYDENVNGKLS
BflorDIA1Lc    FICRSSSR-PHV-CNASSFILETMLVTDVALKVPWLREAWKICHLEKS-----ALSLCVSDRFLEDVRQLYVEDGGTDMT
AaegyDIA1      SICNDLKI-YR-NCRLRNKEPYLRVL--------------RQRVLNDN--QLEGAILCPLMDEE--ALKRFLFEIDEN--
CpipiDIA1      AVCDELGL-TQSNCKFKSDENTLKVL--------------RRKVL-EQ--ELEGCIICPSKDEK--ALNRLLNEVEGT--
DyakuDIA1      Q-IKDVKK-AF--------------------------------YELGE--RPGGFHLCRTPSRKP-RFVSYLEQRGHA--
DerecDIA1      K-AENVEK-AF--------------------------------YEQEE--QPGGFHLCRTPSRKPPRFVSYLEQRGHA--
DmelaDIA1      Q-IKNVKK-DF--------------------------------YELEE--RPGGFHLCRTLAREP-RFVSYLEQRGHA--
DsechDIA1      Q-MKNVKR-AF--------------------------------YELEE--RPGGFHLCRTLAREP-RFVSYLEQRGHVY-
DpersDIA1      RNMGILRE-AF--------------------------------LEMEE--RPGGFQLCRSGFRTP-RFVSYLEQRGHE--
DpseuDIA1      RNMGILGE-AF--------------------------------LEMEE--RPGGFQLCRSGFRTP-RFVSYLEQRGHE--
DananDIA1      S-LSSLQE-EF--------------------------------LNLEE--RPGGFHLCRTAQKSS-PFINYLNQRGYD--
DwillDIA1      D------------------------------------------LHF--NPGGFQMCRNSTR----FLAYLEERGYL--
NvitrDIA1      MICSDDEL-HELCYQSESRKQDSKTN-------FYKLVESELANVGGD--INNKMRLCPTTTKLEKLFVNVKFQSHAEN-
SpurpDIA1L     IICNQSQVDPKRSCEVNAAATNSWMSKS----SALNRVHKLHQEVYESQLIAISATTCASPDFFEELKQLYRVSRWGTG-
BflorDIA1La    FVCRNLSETATS-CDPGKVLLQEDLK-----------------ERLQP----AHLKKSLREVFHPNTTSSLQATTCMS--
CinteDIA1      WICDKFKKPTN--CDTGKVFKSWRAMN-----KITSAKGRLDPNLLKD--I-SPPLQCPSTRIADRIVRRVANKQGGVLL
AgambDIA1      EYCDAYSLTKQN-CRWAQHDGSVDR-----------AKEFITTSILDS-ARVEGCIFCPATNNRQ-SLQRFLSLFDSTDN
                                                                          .
```

```
                      330       340       350       360       370       380       390       400
                       |         |         |         |         |         |         |         |
SpurpDIA1     ----LARDHKLMLATTIAFNPEPLILQAFP----NRKGWPFPAYMGACGRFTVQEYSG-HSLSYFYKFK--FGVRAALAV
NvectDIA1     ----LSLVETIHLITGLHLNPELLLLQIFR----QEEGWPFPRFYGVCGRIMVVEDSG-PPISSFLEES--WDVRAQIAV
GaculDIA1     LLKNLKDTERMQLLMTLAFNPEPLVLQSFP----SDEGWPFAKYLGACGRMVAVNYVG-EELWSFYNAP--WEKRVDLAR
OlatiDIA1     LLKNLKDTERMQLLMTLAFNPEPLVLQSFP----SDEGWPFAKYLGACGRMVAVNYVG-EELWNFYNAP--WEKRVDLAR
TnigrDIA1     LLKNLKDTERMQLLMTLAFNPEPLVLQSFP----SDEGWPFAKYLGACGRVVAVNYVG-EELWSFYNAP--WEKRVDLAR
TrubrDIA1     LLKNLKDTERMQLLMTLAFNPEPLVLQSFP----SDEGWPFAKYLGACGRVVAVNYVG-EELWSFYNAP--WEKRVDLAR
DreriDIA1b    LLKNLKDTERMQLLMTLAFNPEPLVLQSFP----SDEGWPFAKYLGACGRMVAVNYVG-EELWSFFNAP--WEKRVDLAK
DreriDIA1a    LLKNLKDTERMQLLMTLAFNPEPLVLQSFP----SDEGWPFAKYLGACGRMVAVNYVG-EELWSFYNAP--WEKRVDLAK
BtaurDIA1     LLRNLKDSERMQLLLTLAFNPEPLVLQSFP----SDEGWPFAKYLGACGRMVAVNYVG-EELWSYFNAP--WEKRVDLAW
PpygmDIA1     LLRNLKDSERMQLLLTLAFNPEPLVLQSFP----SDEGWPFAKYLGACGRMVAVNYVG-EELWSYFNAP--WEKRVDLAW
CfamiDIA1     LLRNLKDSERMQLLLTLAFNPEPLVLQSFP----SDEGWPFAKYLGACGRMVAVNYVG-EELWSYFNAP--WEKRVDLAW
PtrogDIA1     LLRNLKDSERMQLLLTLAFNPEPLVLQSFP----SDEGWPFAKYLGACGRMVAVNYVG-EELWSYFNAP--WEKRVDLAW
TtrunDIA1     LLRNLKDSERMQLLLTLAFNPEPLVLQSFP----SDEGWPFAKYLGACGRMVAVNYVG-EELWSYFNAP--WEKRVDLAW
HsapiDIA1     LLRNLKDSERMQLLLTLAFNPEPLVLQSFP----SDEGWPFAKYLGACGRMVAVNYVG-EELWSYFNAP--WEKRVDLAW
MmulaDIA1     LLRNLKDSERMQLLLTLAFNPEPLVLQSFP----SDEGWPFAKYLGACGRMVAVNYVG-EELWSYFNAP--WEKRVDLAW
PvampDIA1     LLRNLKDSERMQLLLTLAFNPEPLVLQSFP----SDEGWPFAKYLGACGRMVAVNYVG-EELWSFFNAP--WEKRVDLAW
MdomeDIA1     LLRNLKDSERMQLLLTLAFNPEPLVLQSFP----SDEGWPFAKYLGACGRMVAVNYVG-EELWSYFNAP--WEKRVDLAW
MmuscDIA1     LLRNLKDSERMQLLLTLAFNPEPLVLQSFP----SDEGWPFAKYLGACGRMVAVNYVG-EELWSYFNAP--WEKRVDLAW
RnorvDIA1     LLRNLKDSERMQLLLTLAFNPEPLVLQSFP----SDEGWPFAKYLGACGRMVAVNYVG-EELWSYFNAP--WEKRVDLAW
GgallDIA1     LLRNLKDSERMQLLITLAFNPEPLVLQSFP----SDEGWPFAKYLGACGRMVAVNYVG-EELWSYFNAP--WEKRVDLAW
XtropDIA1     LLRNLKDTERMQLLLTLAFNPEPLVLQSFP----SDEGWPFAKYLGACGRMVAVNYVG-EELWSYFNAP--WEKRVDLAW
BflorDIA1     --EELSYQEKLHFLSTLKFNPEPLMLQTFP----ITEGWPFPFYFGACGRLTVVQKCD-KTLASYYSAP--WLKRVELSL
SsalaDIA1R    QMKHFSERDKLRLLYTLAVNQQPLILQMFP----GTEGWPFPRYQGSCGRLMVWASS--RPLWGLYGSSREFVQRVDVAY
DreriDIA1R    QMKHFNEKDKLRLLYTLAVNQQPLILQMFP----GTEGWPFLRYHGSCGRLMVWAAS--RALRTLFSSP--LERRADLAY
RnorvDIA1R    FMDHFTDRDKLRLLYTLAVNAHPIILQIFP----GAEGWPMPKYLGSCGRFLVSTST--RPLQEFYDAP--PEQAADIAY
MmuscDIA1R    FMDHFTAGDKLRLLYTLAVNAHPIILQIFP----GAEGWPMPRYLGSCGRFLVSTST--RPLQEFYDAS--PEQAADLAY
MmulaDIA1R    FMDHFTDRDKLRLLYTLAVNSHPILLQIFP----GAEGWPLPQYLGSCGRFLVSTST--RPLQEFYGAP--PDQAADLAY
HsapiDIA1R    FMDHFTDRDKLRLLYTLAVNSHPILLQIFP----GAEGWPLPKYLGSCGRFLVSTST--RPLQEFYDAP--PDQAADLAY
BtaurDIA1R    FMDHFTDRDKLRLLYTLAVNTHPVLLQIFP----GAEGWPLPQYLGSCGRFLVSTST--SPLQEFYGAP--PDQAADLAY
EcabaDIA1R    FMDHFTDRDKLRLLYTLAVNAHPILLQIFP----GAEGWPLPRYLGSCGRFLISTST--SPLQEFYSAP--PDQAADLAY
DordiDIA1R    FMDHFTDRDKLRLLYTLAVNAHPIFLQIFP----GAEGWPLPKYLGSCGRFFVSTST--RPLQEFYGAP--PDEAADLAY
MdomeDIA1R    FMDHFTDRDKLRLLYTLSINAHPIMLQIFP----GAEGWPLPRYLGSCGRFLVSTST--QPLEEFYSSP--ADQAADLAY
OanatDIA1R    YMDHFTDRDKLRLLYTLSVNAHPVLLQVFP----GAEGWPFPKYLGSCGRLLVSTST--TPLLELSSGP--PDRAADLGH
GgallDIA1R    YMDHLTDQDKLRLLYTLSVNSHPILLQIFP----DVEGWPFPRYLGSCGRLVVSAST--RPLRDFFRAA--PEVAADLAL
BflorDIA1Lb   K------TERAYMITALLLNPEAALLKHFTS--RAEEYWPFPKYLGACGRVILVESGG-KLLGSAIESP--WKERANIAL
BflorDIA1Lc   KE---GDTGRAFLSTSLLLNEEAVLLRYFTT--KSTTPWPFPKFYGACGRVIVVEHAG-RTLDTFMESS--WEVRADIAL
AaegyDIA1     ---------DLNKILLMRINAQPLLLKLLQ-----ERNFPVPKLIFQGGFTLVESYEG-EALAKYYDRP--LNVRLLIAN
CpipiDIA1     ---------ELLQLILLKVNVQPLLLELFD-----GRGFPVPKVIFQGGFQLFESFDG-DALVNFYDSS--LNIRLRIAK
DyakuDIA1     ---------SASVWFYMMHYVSPLLMQELY-----LQGFPVPTTYASCGLTHFQSYAG-RTLTNYFEGE--EDLRVELAL
DerecDIA1     ---------SASVWFYMMHYVSPLLMQELH-----LQGFPVPTTYASCGLTHFQAYAG-RTLTNYFEAE--EGLRMELAL
DmelaDIA1     ---------AASVWFYMMYYVSPLLMQELH-----LQGFPVPTTYASCGLTHFQSNAG-RTLAHYVDAE--EGLRVELAL
DsechDIA1     --------GSTSVWFYMMHYVSPLLMQELH-----LQGFPVPTPYASCGLTHFQSYAG-RTLAHYVNAE--EGLRLELAL
DpersDIA1     ---------AATIWYYMMHSITPLLMQELF-----LRGFPVPRSYAACGLTHFQAYAG-RTLVHYATAG--ENLRLEMAR
DpseuDIA1     ---------AATIWYYMMHSITPLLMQELF-----LRGFPVPRSYAACGLTHFQAYAG-RTLVHYATAG--ENLRLEMAR
DananDIA1     ---------SSSIYFYMLHNISPLLMQELQ-----NLDFPVPNTYAICGLTHFQAYAG-RTLNHYTEAA--EDLRREIAR
DwillDIA1     ---------PATIGYYIHHSISPLLMQELW-----IMDFPVPRSFAVCGSTLFQAYAG-STLSNYLKAP--IDLRLEMAK
NvitrDIA1     ------EDYFKYLWSTIKINPEPLILQILP----AKEGWPTPKYFGACGRLIVEEYIG-LPLSSFIDEP--WIRRAKIAS
SpurpDIA1L    ----IASTEMAILATTMALNPEPALLKFFRN--IPSLRPYFTEYLGECGRVILTEPSG-KPLSSYLKAS--WKDRVDISL
BflorDIA1La   ------KPFLKLLQKTFDDNGNKRLNRLEQA--IPRKKLPIPAFLGACGNLVATETAG-KPLSMYLEVKGPWQVRANLSL
CinteDIA1     R--DLSYTEKKRLIFTCKMSPGSVVQSMFP----PYEGWLFQRTVGSCGALMVHGRSIVTSLYELYDAP--WKVRLDVAI
AgambDIA1     ---------ELWNLLAVRTNVEPLLIKLLTVNYHDEPPLYVPKLLHTFGFSIIETYEG-KTLEHYYDFP--FVTRMRIAA
                                      .                       *            :               :.
```

```
                       410       420       430       440       450       460       470       480
                         |         |         |         |         |         |         |         |
SpurpDIA1       QALKIAEQLSENRDEFALYLTDVSEDNLAVND-NG----EVLVIDAENIIVVDRKKIKEDANPGWDVKH-----QSEHEG
NvectDIA1       NLITLAHQLSSALDDWALYIADPAPGNFGVSD-SG----KVTLLDLEHLVVVDLSEVQMEEANDKPKPS----------D
GaculDIA1       QLMDIAEQLTNNDFDFALYLLDVSFDNFAVGPRDG----KVIVVDAENVLVADKRLIKQNKPESFDVWY-----ESRFEE
OlatiDIA1       QLMDIAEQLTNNDFDFALYLLDVSFDNFAVGPRDG----KVIVVDAENVLVADKRLIKQNKPESYDVWY-----ESRFEE
TnigrDIA1       QLMDIAEQLTNNDFEFALYLLDVSFDNFAVGPRDG----KVIIVDAENVLVADKRLIKQNKPENFDVWY-----ESRFEE
TrubrDIA1       QLMDIAEQLTNNDFEFALYLLDVSFDNFAVGPRDG----KVIIVDAENVLVADKRLIKQNKPENYDVWY-----ESRFEE
DreriDIA1b      QLMDIAEQLTNNDFDFALYLLDVSFDNFAVGPRDG----KVIVVDAENVVVADKRLIKQNKPESYDVWY-----ESRFEE
DreriDIA1a      QLMDIAEQLTNNDFDFALYLLDVSFDNFAVGPRDG----KVIVIDAENVIVVDKRLVKQNKPESYDVWY-----ESRYEE
BtaurDIA1       QLMEIAEQLTNNDFEFALYLLDVSFDNFAVGPRDG----KVIIVDAENVLVADKRLIRQNKPENWDVWY-----ESKFDD
PpygmDIA1       QLMEIAEQLTNNDFEFALYLLDVSFDNFAVGPRDG----KVIIVDAENVLVADKRLIRQNKPENWDVWY-----ESKFDD
CfamiDIA1       QLMEIAEQLTNNDFEFALYLLDVSFDNFAVGPRDG----KVIIVDAENVLVADKRLIRQNKPENWDVWY-----ESKFDD
PtrogDIA1       QLMEIAEQLTNNDFEFALYLLDVSFDNFAVGPRDG----KVIIVDAENVLVADKRLIRQNKPENWDVWY-----ESKFDD
TtrunDIA1       QLMEIAEQLTNNDFEFALYLLDVSFDNFAVGPRDG----KVIIVDAENVLVADKRLIRQNKPENWDVWY-----ESKFDD
HsapiDIA1       QLMEIAEQLTNNDFEFALYLLDVSFDNFAVGPRDG----KVIIVDAENVLVADKRLIRQNKPENWDVWY-----ESKFDD
MmulaDIA1       QLMEIAEQLTNNDFEFALYLLDVSFDNFAVGPRDG----KVIIVDAENVLVADKRLIRQNKPENWDVWY-----ESKFDD
PvampDIA1       QLMEIAEQLTNNDFEFALYLLDVSFDNFAVGPRDG----KVIIVDAENVLVADKRLIRQNKPENWDVWY-----ESKFDD
MdomeDIA1       QLMEIAEQLTNNDFEFALYLLDVSFDNFAVGPRDG----KVIIVDAENVLVADKKLIRQNKPENWDVWY-----ESKFDD
MmuscDIA1       QLMEIAEQLTNNDFEFALYLLDVSFDNFAVGPRDG----KVIIVDAENVLVADKRLIRQNKPENWDVWY-----ESKFDD
RnorvDIA1       QLMEIAEQLTNNDFEFALYLLDVSFDNFAVGPRDG----KVIIVDAENVLVADKRLIRQNKPENWDVWY-----ESKFDD
GgallDIA1       QLMEIAEQLTNNDFEFALYLLDVSFDNFAVGPRDG----KVIIVDAENVLVADKRLIRQNKPENWDVWY-----ESKFDD
XtropDIA1       QLMEIAEQLTNNDFDFALYLLDVSFDNFAVGPRDG----KVIIVDAENVLVADKKLIKQNKPENWDVWY-----ESKFDD
BflorDIA1       QMMKIAEYLTNNEADFGLYLTDISYENFGVTS-DG----NLFVIDVENVIVVDKQKIKADKPRNWEARY-----QSHFDE
SsalaDIA1R      QLLHITQGLGHNSLGFLLYYTRLGEDMFGLLD-DQ----RVFITDASSIGVIDLEQGFPPDPPS-QTGS-----DGDIFS
DreriDIA1R      QLLHITQSLSANSLRFRLFYTRIPEDMFGILE-DN----KVFIVDTSTIGIIDLQEGHPPDK-D-LLPE-----ELDVFS
RnorvDIA1R      QLLGVLESLRSNDLNYFFYFTHVDAGMFGIFD-NG----HLFIRDASALGIIDKQEASQAAD---RTGE-----NEDIFS
MmuscDIA1R      QLLRVLESLRSNDLNYFFYFTHVDAGMFGIFD-NG----HLFIRDASALGIIDKQEGSQAAA---RTGE-----NEDIFS
MmulaDIA1R      QLLGVLESLRSNDLNYFFYFTHIDAGMFGVFN-NG----HLFIRDASAVGVIDKQEGSQEAT---RAGE-----NKDIFS
HsapiDIA1R      QLLGVLESLRSNDLNYFFYFTHIDAGMFGVFN-NG----HLFIRDASAVGVIDKQEGSQEAN---RAGE-----NKDIFS
BtaurDIA1R      QLLGVLESLRSNDLNYFFYFTHVDADMFGIFN-NG----HLFIRDASALGVIDRQEGSQAAS---GAGD-----NKDIFS
EcabaDIA1R      QLLGVLESLRSNDLNYFFYFTHVDAGMFGIFN-NG----HLFIRDASTLGVIDRQEGSQAAA---RAGE-----NKDIFS
DordiDIA1R      QLLGVLESLRSNDLNYFFYFTHVDAAMFGIFS-NG----HLFIRDASALGVIDKQEGSQAAP---KAGE-----NQDIFS
MdomeDIA1R      QLLGILDSLRNNDLNYFFYFTRIDAGTFGIFD-NG----HLFIRDASTVGVIDKQRGSLTLD---RQEE-----SKDIFS
OanatDIA1R      QLLGVIEFLRHNDWNYFFYFTRIHAGTFGVFD-NG----HLFIRDASSLGVIDRQEGKRPAR---SSPE-----PRDIFS
GgallDIA1R      QLLAVLHSMGTNDLNYFFYFTRVDVGTFGVFS-NG----HLFIRDASTLGIIDKEEGSQPID---GQQE-----YKDIFS
BflorDIA1Lb     QLLEMIDKFRNGDPKWVVIFVDFSFNNFAVNN-YG----RLTLIDFDDVMLIDREEFVGENK--TEPCD-----LKCFKT
BflorDIA1Lc     QLLQLVDALREKDPDWVLFFLDVSFQNFAVDS-RG----WVRLIDLDDVMVIDRRTVVNQEQ--TEMCN-----EQCYTD
AaegyDIA1       ELIKASLNFTAGVDNFRFYLTDISPDNIVVSMSSEEE-IKVTFVDLNNVIILDSHSKRLKP------SK-----QKHVHS
CpipiDIA1       ELIQASFLFTEGVNGFRFYLTDINPDNIAVQAQPSGS-FQVSFIDLDNVIILDSQSKRLDRR-----SK-----ARNIHS
DyakuDIA1       QLMQLSLKLTFGFSDFRIMLTDFTGDNFAYDEHTK----KVYLIDLDSVVLVD----ASSAT-----GQ-----AEKYEP
DerecDIA1       QLIQLSLKLTFGFSDFRIFLTDFTGDNFAYDEDSK----RVYLIDLDSVVLVD----ASSAA-----GQ-----AEKYVP
DmelaDIA1       QLIQLSLKLTFGFADFRIILTDFTADNLAYDEDTK----KVYLIDLDSVVLVD----ASFAA-----GH-----AEKYEP
DsechDIA1       QLIQLSLKLTFGFADFRIILTDFTADNLAYDEETK----KVYLIDLDSVVLVD----ASSMA-----GQ-----AEKYEP
DpersDIA1       QLLQLSLKLTFGFADFRIFLTDFTADNLAFDEASQ----SVILIDLDSVVLVD----AAVPL-----GD-----AQKYEP
DpseuDIA1       QLLQLSLKLTFGFADFRIFLTDFTADNLAFDEASQ----SVILIDLDSVVLVD----AAVPL-----GD-----AQKYEP
DananDIA1       QLIHLSLKLTFGFADFRIFLTDFTSDNLTFDEDTR----RVLLIDLDSVVMVD----AASTS-----GQ-----AEKYEP
DwillDIA1       QLLQLALKLSAGFKGFRIYLTDFTADNFAYNEANG----TVLLIDMDTMVLVESNDEAESES-----QL-----SKKYKP
NvitrDIA1       SLLQAADTLMSKNSEFAFYLTDISMDNIAVNNEDK-----AIFVDLENIIIVEKNPPEKALVGIESWNE-----TYTNAV
SpurpDIA1L      KILQMIEDFHDSSDKWLVLLLDFGYENFVMTS-EG----QLKVVNLGGMVIVDKDQTSTTPDMNPHLNNRTELCNEDCLN
BflorDIA1La     QLLQMLDDFQNKDPDWLLMFVEVNIENFSVSS-DG----RLILTDLGNMTIINKHDLDKNSTKKRSSVC-----NEACFK
CinteDIA1       QLLDLAEHFTYNDLNYMFALHRLTPRDIGISD-TG----KLRIKNADNVILID----NNSKN-----------DKGLYE
AgambDIA1       ELIRAGFKFTEGIHGFRVFLTDINPDNVVVNLKNDNKQVYVSIVDLDNVILLDSWAEVFLTKN-----------THHVHS
                . :     :      : .         .              . :   : : :
```

```
                    490       500       510       520       530       540       550       560
                      |         |         |         |         |         |         |         |
SpurpDIA1     CGSRRECIMFSHTDLCNHYYSDHNYYAVCQGLFGKDSPHGD-----------GGLLHSIPSDL--------SSYMRAKVK
NvectDIA1     CS-QPDCLSFSPSDMCSGGSRDHNYYAICQGVLAGSSRRQ-------------GLLHNPP------------VALKASLN
GaculDIA1     CD-REACLSFSKESLCSRVTVDHNYYAVCQNLLSRYATWRGTT---------GGLLHDPPAH----------IAKDGQLL
OlatiDIA1     CD-REACLSFSKDSLCSRVTVDHNYYAVCQNLLSRYATWRGTT---------GGLLHDPPAH----------IAKDGQLE
TnigrDIA1     CD-REACLSFSKDSLCSRVTVDHNYYAVCQNLLSRYATWRGTT---------GGLLHDPPAH----------IAKDGQLE
TrubrDIA1     CD-REACLSFSKDSLCSRVTVDHNYYAVCQNLLSRYATWRGTT---------GGLLHDPPAH----------IAKDGQLE
DreriDIA1b    CD-KEACLSFSKDMLCSRVTVDHNYYAICQNLLSRYATWRGSS---------GGLLHDPPPH----------IAKDGQLE
DreriDIA1a    CD-KEACLSFSKDILCSRVTVDHNYYAVCQDLLSRFSSWRGST---------GGLLHDPPPD----------VVKDGRLI
BtaurDIA1     CD-KEACLSFSKEILCARATVDHNYYAVCQNLLSRHATWRGTS---------GGLLHDPPSE----------IAKDGRLE
PpygmDIA1     CD-KEACLSFSKEILCARATVDHNYYAVCQNLLSRHATWRGTS---------GGLLHDPPSE----------IAKDGRLE
CfamiDIA1     CD-KEACLSFSKEILCARATVDHNYYAVCQNLLSRHATWRGTS---------GGLLHDPPSE----------IAKDGRLE
PtrogDIA1     CD-KEACLSFSKEILCARATVDHNYYAVCQNLLSRHATWRGTS---------GGLLHDPPSE----------IAKDGRLE
TtrunDIA1     CD-KEACLSFSKEILCARATVDHNYYAVCQNLLSRHATWRGTS---------GGLLHDPPSE----------IAKDGRLE
HsapiDIA1     CD-KEACLSFSKEILCARATVDHNYYAVCQNLLSRHATWRGTS---------GGLLHDPPSE----------IAKDGRLE
MmulaDIA1     CD-KEACLSFSKEILCARATVDHNYYAVCQNLLSRHATWRGTS---------GGLLHDPPSE----------IAKDGRLE
PvampDIA1     CD-KEACLSFSKEILCARVTVDHNYYAVCQNLLFRHATWRGTS---------GGLLHDPPSE----------IAKDGRLE
MdomeDIA1     CD-KEACLSFSKEILCSRATVDHNYYAICQNLLSRHATWRGTS---------GGLLHDPPGE----------IAKDGRLE
MmuscDIA1     CD-KEACLSFSKEILCARVTVDHNYYAVCQNLLSRHATWRGTS---------GGLLHDPPSE----------IAKDGRLE
RnorvDIA1     CD-KEACLSFSKEILCARVTVDHNYYAVCQNLLSRHATWRGTS---------GGLLHDPPSE----------IAKDGRLE
GgallDIA1     CD-KEACLSFSKEILCARVTVDHNYYAVCQNLLSRHATWRGTS---------GGLLHDPPAD----------IAKDGRLE
XtropDIA1     CD-KEACLSFSKEILCSRATVDHNYYAICQNLLSRHATWRGTS---------GGLLHDPPAE----------IAKDGRLE
BflorDIA1     CPGMSNCLSFDVSALCRYLHTDHNYYAVCRNMLSEYASEMGKP---------GGLLHDPPET----------VVRDGTLQ
SsalaDIA1R    CLGQGTSPCHR-SPPCSSVRPTQSLTLLCTALLPRLLLTERGAQTTRLPMEGEAEAGRLARDV--------PLLLGVCAD
DreriDIA1R    CL--SGSCVR--PPPCETVREAQSFILLCKYILN-NLLTSN-----------DKQSG-LPRAA--------VDELLVCAD
RnorvDIA1R    CL-VSGCQVQ--MSSCDTVPEKQNLVLVCQQLLP-QLLRG-------------KFPSPVQQEI--------DSALSLCSK
MmuscDIA1R    CL-VSDCQIQ--LSSCDTVPEKQSLVLVCQQLLP-QLLQG-------------KFPSPVQKEI--------DSALSLCSK
MmulaDIA1R    CL-VSGCQAQ--LPSCESVSEKQSLVLVCQKLLP-RLLQG-------------RFPSPVQDDI--------DSALAQCGD
HsapiDIA1R    CL-VSGCQAQ--LPSCESISEKQSLVLVCQKLLP-RLLQG-------------RFPSPVQDDI--------DSILVQCGD
BtaurDIA1R    CL-VSGCQTK--LPSCDTIPEKQNLVLVCSQVLP-LLLQA-------------KFPSPVQEEI--------DAELTRCAD
EcabaDIA1R    CL-LSDCQAE--LPSCDTVPEKQSLVLVCRQVLP-RLLQG-------------KFPSPVQEEI--------DATLTWCGE
DordiDIA1R    CL-VSGCQAQL-LPSCDNIPDKQSVVLVCQQLLP-QLLQG-------------KFPSPVQVEI--------DSALAQCGQ
MdomeDIA1R    CL-VLDCQSP--FPSCSSVKEKQSWVMACQQLLP-QLLKE-------------KFPQPIQEQI--------DSALVLCGN
OanatDIA1R    CL-AADCQSD--LPSCNTVQEHQSLVLVCRELLP-KLLEG-------------KFPKPVQEEI--------DALLDRCGD
GgallDIA1R    CL-TVDCQSA--FVSCNSIREKQSLVMVCQELLP-KLLRG-------------KFLPPVQEKI--------DSFLQHCAE
BflorDIA1Lb   FISQIEAMNSYDSCSAMPQYSQMMYALACVRLLSHLPEHLSEP--NPMDPKPDKHRPDSERP---RLLWGPPEQEAKVLE
BflorDIA1Lc   FQKKLYS-DEYH-CDDIFKYAPMMYASICARLLSNLQKHPERRKWGEIREYQEGQSIELDEPPVKGFLHNPPDEIREALE
AaegyDIA1     RIDCDGCFAYIQEDICVHQISDINLFAVCQLLLENLNGDSKR-----------GFLHSLGTDS-----------KLEAFR
CpipiDIA1     RIPCDGCFAYVQEDLCSYQHSDINQFAICQLLYENLNGDREG-----------GFLHIQPNDD--------SQPRLSEIR
DyakuDIA1     -LPGEG-FTFDVSAFCSGHQLDANIYQACLLLRDYLLKNLNN----------------------------------ERLQ
DerecDIA1     -LPGEG-FTFDVSAFCSGHQLDANIYQACLLLRDFLLKNLDN----------------------------------ERLQ
DmelaDIA1     -LTGEG-FTFDVSAFCSGHQLDANIYQACLLLRDYLLKNLDN----------------------------------EKLQ
DsechDIA1     -LAGEG-FTFDVSAFCSGHQLDANIYQACLLLRDSLLKNLDN----------------------------------EKLQ
DpersDIA1     -LPGDG-FTFDVKSFCGGQQLDANVYQACLLLKDFLLRDLAN----------------------------------GRLE
DpseuDIA1     -LPGDG-FTFDVKSFCGGQQLDANVYQACLLLKDFLLRDLAN----------------------------------GRLE
DananDIA1     -LPGEG-FTFDVSAFCSGRQLDANVYQACLLLRDFLLKDLDN----------------------------------ERLQ
DwillDIA1     -FPGEG-FTYDVNAFCDNQDLDANIYQVCLLLRDQLLPDVKN----------------------------------HRLK
NvitrDIA1     DLDCQDCFVFSPNDICSHKVSDHNFYAICQHILTQALGAVFHN---------QGFLHDPPD---------YILQKHPTLI
SpurpDIA1L    TFVKQLQTEPDTHCREVPRHVELMYMMACHSLLSDLMTTKYER--------FFQPLDTPRKHHPGGMLHDAPYEVDSVLS
BflorDIA1La   RFTRNLSHRPETSCRQAGRYSQLMYARACQRILGCWQTERSGE-----GRVVLSSSKDASCAYGRGLLFGPPREAKQELE
CinteDIA1     VRYKQEKVDITEENLCDHSENDVNYYVICRHFLGGTWKERKGK------KYPGGLLHDPKEFG----------VFSSQMF
AgambDIA1     KIECNGCFAYVQEDVCRYQNSDLNLFATCQLLLENLNGHYAK-----------GLLHYDRDAEAFP----VLGEASKMLQ
                                          *  .
```

```
                     570       580       590       600       610       620       630
                       |         |         |         |         |         |         |
SpurpDIA1     GQIAECRSPSK--RDGRYLVVKELKETLM---------------KLIT------------------------
NvectDIA1     QLLQDCTAQDM--RNTREETAQKLSELLQ---------------GYL-------------------------
GaculDIA1     TLLDECTRPKK--RYGRFQAAKELREYLT---------------QLAAASSSATAR----------------
OlatiDIA1     ALLDECTRPKK--RYGRFQAAKELREYLT---------------KLAAASSSATSR----------------
TnigrDIA1     ALLDECTKPKK--RYGRFQAAKELREYLT---------------QLA-------------------------
TrubrDIA1     ALLDECTKPKK--RFGRFQAAKELREYLT---------------QLAASSSSLVSAR---------------
DreriDIA1b    ALLEECANPKK--RYGRFQAAKELRDYLT---------------QLSGSAR---------------------
DreriDIA1a    ALLDECTRPQK--RYGRFQAAKELREFLT---------------QLTQTSNADR------------------
BtaurDIA1     ALLDECANPKK--RYGRFQAAKELREYLA---------------QLSNNVR---------------------
PpygmDIA1     ALLDECANPKK--RYGRFQAAKELREYLA---------------QLSNNVR---------------------
CfamiDIA1     ALLDECANPKK--RYGRFQAAKELREYLA---------------QLSNNVR---------------------
PtrogDIA1     ALLDECANPKK--RYGRFQAAKELREYLA---------------QLSNNVR---------------------
TtrunDIA1     ALLDECANPKK--RYGRFQAAKELREYLA---------------QLSNNVR---------------------
HsapiDIA1     ALLDECANPKK--RYGRFQAAKELREYLA---------------QLSNNVR---------------------
MmulaDIA1     ALLDECANPKK--RYGRFQAAKELREYLA---------------QLSNNVR---------------------
PvampDIA1     ALLDECANPKK--RYGRFHAAKELREYLA---------------QLSNNVR---------------------
MdomeDIA1     ALLDECAHPKK--RFGRFQAAKELREYLA---------------QLSNNGR---------------------
MmuscDIA1     ALLDECTNPKK--RYGRFQAAKELRGYLA---------------QLSHNVR---------------------
RnorvDIA1     ALLDECTNPKK--RYGRFQAAKELRGYLA---------------QLSHNVR---------------------
GgallDIA1     ALLDECANPKK--RYGRFQAAKELREYLA---------------QLSNNVR---------------------
XtropDIA1     ALLDECANPKK--RYGRFKSAKELREYLA---------------QLSNNAR---------------------
BflorDIA1     RLLAECAKPRT--LYGRFDAAKELIELLG---------------SFLKDR----------------------
SsalaDIA1R    PSQPDWRIMAA--VGSLMDLLKPMRPCNP---------------HYTYRYPECRYNQDY-------------
DreriDIA1R    PSQLDQTIIKS--VQSLKNILKTLRPCSP---------------QYAYRYPECLYSDKF-------------
RnorvDIA1R    DASTNLEVFGA--TSLLKNILRSLRTCDP---------------RFAYRYPDCKYNDRF-------------
MmuscDIA1R    DNSTDLEVLGA--TSWLKDILRSLRTCDP---------------RFAYRYPDCKYNDRF-------------
MmulaDIA1R    STRPDPEVLGA--ASWLKHILRPLRTCDS---------------RFAYRYPDCKYNDKF-------------
HsapiDIA1R    SIRPDPEVLGA--ASQLKDILRPLRTCDS---------------RFAYRYPDCKYNDKF-------------
BtaurDIA1R    GTRPDPEVLGA--ASRLKDILRPLRTCDP---------------RFAYRYPDCKYDDKF-------------
EcabaDIA1R    DTRPDAEVLGA--ASRLKDILRPLRTCDP---------------RFAYRYPDCKYNDKF-------------
DordiDIA1R    GIRPDSEVFGA--ASRLKDILRPLRTCDP---------------RFAYRYPDCKYNDRF-------------
MdomeDIA1R    SSLSDQEVIEA--ASRLKAILKPLRTCDP---------------RYAYRYPDCKYSDRY-------------
OanatDIA1R    SSLDGQTVIGG--ARRLMDILKSLRTCDA---------------RFAYRYPDCKYSEKY-------------
GgallDIA1R    GLADDQDVNEA--MAKLAQLLKPLRSCDS---------------RFAYRYPDCKYSDKY-------------
BflorDIA1Lb   ELLRGCVEENV--AGGRLEAVGELKTFLR---------------RNAKGEN---------------------
BflorDIA1Lc   GALTQCVHETL--PRGRLGAVLRLQEILG---------------TT--------------------------
AaegyDIA1     KLLHQCVYCQPPYCEDRQEVLQRIMEIIH---------------DVLHEVV---------------------
CpipiDIA1     QLLHHCVYCVPPDCRDRQGLLQQVQEIID---------------GILVES----------------------
DyakuDIA1     LLLEQCVACQDDFCDMRFQHAYDLIKMLD---------------SKN-------------------------
DerecDIA1     LLLEQCVACQDDFCDMRFQHAYDLIKVLD---------------SKI-------------------------
DmelaDIA1     LLLEQCVACQDDFCDMRFQHAYDLIKVLE---------------SNN-------------------------
DsechDIA1     LLLEQCVACQDDFCDMRFQHAYDLIKELE---------------SKN-------------------------
DpersDIA1     MLLEQCVRCDDATCDMRFQQAYDLIKLLG---------------ELLGS-----------------------
DpseuDIA1     MLLEQCVRCDDATCDMRFQQAYDLIKLLG---------------ELLGS-----------------------
DananDIA1     LLLEKCVECQDDLCDMRFQYAYDLIKLLD---------------S---------------------------
DwillDIA1     EMLNDCVQCEDDKCDIRFKYANNLIDLLV---------------EINNKIEIL-------------------
NvitrDIA1     DLLEQCAKPDIG--YSRIDIAHKLIVLLD---------------SVIKNA----------------------
SpurpDIA1L    ELLYECVFEGQ--PGRRMHSVRVLKRLLT---------------IIQRGFSYKDAADRTSL-----------
BflorDIA1La   DLLTECVEETK--AGGRITALKQIRVLLA---------------A---------------------------
CinteDIA1     VLLDICSRGKTAVAPERRTLSHSIRRLAANQLRNVLERRRICSPDFKYRYPECELAEDAGINLPSIELQWLH
AgambDIA1     NLLSECVYCQPPDCQNRSHILKDMLHIID---------------QTIIQS----------------------
                                     :
```
