# Supplementary material for: Characterization of the Deleted in Autism 1 Protein Family: Implications for Studying Cognitive Disorders
Source: PLoS One. 2011 Jan 19;6(1):e14547. doi: 10.1371/journal.pone.0014547 (PMC3023760; doi:10.1371/journal.pone.0014547)
Supplement: Figure S8 — Amino acids conserved in at least 80% of DIA1-family members. The sequence alignment of all full-length DIA1, DIA1R and DIA1L proteins was generated using CLUSTALW [47]. A consensus amino acid sequence is presented below the alignment, where uppercase letters indicate absolutely conserved amino acids. Regions of greater than 80% conservation are shaded (identical amino acids are in black boxes and similar amino acids in grey boxes), and 10% of aligned positions showed conservation in >80% of sequences. Amino acid numbering is provided on the left-hand side of the alignment. Gaps required for optimal alignment are indicated by dashes. Standard single-letter amino acid abbreviations are used. Organism abbreviations use the first letter of the genus name, followed by the first four letters of the species (e.g., Salmo salar DIA1R is abbreviated to SsalaDIA1R). Accession numbers and full species names can be found in Tables S1, S5 and S8. (0.10 MB PDF) [file pone.0014547.s018.pdf]

Fig. S8

```
SpurpDIA1      1 ------------------------------------------------------------
NvectDIA1      1 ------------------------------------------------------------
GaculDIA1      1 ------------------------------------------------------------
OlatiDIA1      1 ------------------------------------------------------------
TnigrDIA1      1 ------------------------------------------------------------
TrubrDIA1      1 ------------------------------------------------------------
DreriDIA1b     1 ------------------------------------------------------------
DreriDIA1a     1 ------------------------------------------------------------
BtaurDIA1      1 ------------------------------------------------------------
PpygmDIA1      1 ------------------------------------------------------------
CfamiDIA1      1 ------------------------------------------------------------
PtrogDIA1      1 ------------------------------------------------------------
TtrunDIA1      1 ------------------------------------------------------------
HsapiDIA1      1 ------------------------------------------------------------
MmulaDIA1      1 ------------------------------------------------------------
PvampDIA1      1 ------------------------------------------------------------
MdomeDIA1      1 ------------------------------------------------------------
MmuscDIA1      1 ------------------------------------------------------------
RnorvDIA1      1 ------------------------------------------------------------
GgallDIA1      1 ------------------------------------------------------------
XtropDIA1      1 ------------------------------------------------------------
BflorDIA1      1 ------------------------------------------------------------
SsalaDIA1R     1 ------------------------------------------------------------
DreriDIA1R     1 ------------------------------------------------------------
RnorvDIA1R     1 ------------------------------------------------------------
MmuscDIA1R     1 ------------------------------------------------------------
MmulaDIA1R     1 ------------------------------------------------------------
HsapiDIA1R     1 ------------------------------------------------------------
BtaurDIA1R     1 ------------------------------------------------------------
EcabaDIA1R     1 ------------------------------------------------------------
DordiDIA1R     1 ------------------------------------------------------------
MdomeDIA1R     1 ------------------------------------------------------------
OanatDIA1R     1 ------------------------------------------------------------
GgallDIA1R     1 ------------------------------------------------------------
BflorDIA1Lb    1 MASARRWCRRLHYSGWTRVLFLLTLMIAVGFIARNYWQVEDDLEARKRISNGGDKKYAQS
BflorDIA1Lc    1 ------------------------------------------------------------
AaegyDIA1      1 ------------------------------------------------------------
CpipiDIA1      1 ------------------------------------------------------------
DyakuDIA1      1 ------------------------------------------------------------
DerecDIA1      1 ------------------------------------------------------------
DmelaDIA1      1 ------------------------------------------------------------
DsechDIA1      1 ------------------------------------------------------------
DpersDIA1      1 ------------------------------------------------------------
DpseuDIA1      1 ------------------------------------------------------------
DananDIA1      1 ------------------------------------------------------------
DwillDIA1      1 ------------------------------------------------------------
NvitrDIA1      1 ------------------------------------------------------------
SpurpDIA1L     1 ---------------------MGPRIRKKILILGSSILFTIFTVQLTGLNSLRDDDNNAL
BflorDIA1La    1 -----------------------------MPILVESEAGYGGRHGRAMVARRMVRKCCLP
CinteDIA1      1 ------------------------------------------------------------
AgambDIA1      1 ------------------------------------------------------------
consensus      1
```

```
SpurpDIA1      1 -----------------------------MRRYFNQFTNRFCISTTQQLIWLVVLSSMCL
NvectDIA1      1 -----------------------------MHFGGILRKRRYICRLVLLLIVIISFSDLLL
GaculDIA1      1 ------------------------------MLRFLPL-KLGRLYRCLKLLLVVGLFVILL
OlatiDIA1      1 ------------------------------MLRFLPL-KLGRLYRCLKLLFLVGLFVILL
TnigrDIA1      1 ------------------------------MLRFLPL-KLGRLYRCLKLLLVVGLFVILL
TrubrDIA1      1 ------------------------------MLRFLPL-KLGRLYRCLKLLLVVGLFVILL
DreriDIA1b     1 ------------------------------MLRFLPL-KLGRLYRCLKLLFLLGLFVILL
DreriDIA1a     1 ------------------------------MLRILSL-KFGRVYRCGKFLFIVALFVILL
BtaurDIA1      1 ------------------------------MWRLVPP-KLGRLSRSLKLAALGSLLVLMV
PpygmDIA1      1 ------------------------------MWRLVPP-KLGRLSRSLKLAALGSLLVLMV
CfamiDIA1      1 ------------------------------MWRLVPP-KLGRLSRSLKLAALGSLLVLMV
PtrogDIA1      1 ------------------------------MWRLVPP-KLGRLSRSLKLAALGSLLVLMV
TtrunDIA1      1 ------------------------------MWRLVPP-KLGRLSRSLKLAALGSLLVLMV
HsapiDIA1      1 ------------------------------MWRLVPP-KLGRLSRSLKLAALGSLLVLMV
MmulaDIA1      1 ------------------------------MWRLVPP-KLGRLSRSLKLAALGSLLVLMV
PvampDIA1      1 ------------------------------MWRLVPP-KLGRLSRSLKLAALGSLLVLMV
MdomeDIA1      1 ------------------------------MWRLVPP-KLGRLSRSLKLAALGSLLVLMV
MmuscDIA1      1 ------------------------------MWRLVPL-KLGRLSRALKLAALGSLLVLML
RnorvDIA1      1 ------------------------------MWRLVPL-KLGRLSRALKLAALGSLLVLML
GgallDIA1      1 ------------------------------MLRLVSL-KLGRLYRYVKLAVLGSLAAALV
XtropDIA1      1 ------------------------------MLRLASL-KFGRLFRYAKVLFAASLLVVML
BflorDIA1      1 -------------------------------MRLVRLRQFRRFFSGWRVRRMCGFFFFVV
SsalaDIA1R     1 ------------------------MPL--KRCDQRAVGSVRLT--VCVLSWLLCVCWAPS
DreriDIA1R     1 ------------------------------------MAG------IWSGVWILCFILVFG
RnorvDIA1R     1 ------------------------MELGRPGAAATAFRQRWPAWMLLWVSTLSCSFSLPA
MmuscDIA1R     1 ------------------------MESQWRGAAATAFHQHWLARLLLWVSTLSCSFSLPA
MmulaDIA1R     1 ------------------------MEP-QLGPEAAALRPGWLA-LLLWVSALSCSFSLPA
HsapiDIA1R     1 ------------------------MEP-QLGPEAAALRPGWLA-LLLWVSALSCSFSLPA
BtaurDIA1R     1 ------------------------MEP-RLGPKAAALHLGWPF-LLLWVSGLSYSVSSPA
EcabaDIA1R     1 ------------------------MEP-WLGPEAAALRPGWPA-LLLWVSALRCSVSSPA
DordiDIA1R     1 ------------------------MDP-QLGPEAAALHPGWQV-LLLLVVSVLSCFSSPA
MdomeDIA1R     1 ------------------------MES-VIHGPLASPCLGWLT--LLQLVTLSCISSTES
OanatDIA1R     1 ------------------------MGL-RLGCRLFSAALSWMT--LLQFLTRGHGPVADA
GgallDIA1R     1 ------------------------MGR--WICCLCSRVADWLM--LLLVLARSSNPSAAA
BflorDIA1Lb   61 IQELLAQGIDEKRMREALRQKAEEVHRIVKERGEALAKEAQAKAKAKANVVRINIHKPEL
BflorDIA1Lc    1 ------------------------------------------------------------
AaegyDIA1      1 ------------------------------------MSQHRILTKLHLVALFLWIVMKYQ
CpipiDIA1      1 ----------------------------------MSSGFQRILVNLHILALLFCVIFEFQ
DyakuDIA1      1 ---------------------------------------MHLSPGQLKVVLILALLQELQ
DerecDIA1      1 ---------------------------------------MHLSPGQLKVVLILALLQELQ
DmelaDIA1      1 ---------------------------------------MHLSHGQLKVVLILALLQELQ
DsechDIA1      1 ---------------------------------------MHLSYGQLKVVLILALLQELQ
DpersDIA1      1 ---------------------------------------MHLSPGHLRIVLILALLQDLQ
DpseuDIA1      1 ---------------------------------------MHLSPGHLRIVLILALLQDLQ
DananDIA1      1 ---------------------------------------MHLLSGQLRIVLVLALLQDLQ
DwillDIA1      1 ---------------------------------------MHLLYGQLKVVLVLALLQELD
NvitrDIA1      1 ----------------------------------------MLVTKASVSSFLIALLILIL
SpurpDIA1L    40 PSTNGKFRTFVPSKGDRKRNLLHAGKQGKEELGFDSKDTRRTLQDIRTADEIKGHPRIQA
BflorDIA1La   32 LLVLVSATLAVLYIYLVQVQGSPWVSLGQSQAGNKQKKDEYIVWRGEEQKVEVQQKQEEK
CinteDIA1      1 -----------------------------MLLKMKRYLTVWENGKYIVFIFILITLEVLL
AgambDIA1      1 ---------------------------------------MQSSRLPYFVTLFIAVVLQYL
consensus     61                                                   l  l
```

Signal peptide

```
SpurpDIA1      32 FVYYQ----LYLHFTANHLENEYFTEAT------------KCPACFGT--SLCKRFSRGD
NvectDIA1      32 WLSYQDGRAIKWDFSSQHKLTFQEIGLN------------KCPACFGINLSICHKLLGGG
GaculDIA1      30 MNTHS----LFASFQKNELTDRRFINLN------------KCPACFGT--SWCRKFMNGQ
OlatiDIA1      30 MNTHN----LFASFQKNELTDRRFINLN------------KCPACFGT--SWCRKFMNGQ
TnigrDIA1      30 MNTHN----LFASFQKNELTDRRFINLN------------KCPACFGT--SWCRKFMNGQ
TrubrDIA1      30 MNTHN----LFASFQKNELTDRRFINLN------------KCPACFGT--SWCRKFMNGQ
DreriDIA1b     30 MNTHN----LFASFQKNELTDRRFINLN------------KCPACFGT--SWCRKFMNGQ
DreriDIA1a     30 MNTHN----LFASFQRNELTDRRFIGLN------------KCPACFGT--SWCRKFMNGQ
BtaurDIA1      30 LHSPS----LLASWQRNELADRRFLQLN------------KCPACFGT--SWCRRFLNGQ
PpygmDIA1      30 LHSPS----LLASWQRNELADRRFLQLN------------KCPACFGT--SWCRRFLNGQ
CfamiDIA1      30 LHSPS----LLASWQRNELADRRFLQLN------------KCPACFGT--SWCRRFLNGQ
PtrogDIA1      30 LHSPS----LLASWQRNELADRRFLQLN------------KCPACFGT--SWCRRFLNGQ
TtrunDIA1      30 LHSPS----LLASWQRNELADRRFLQLN------------KCPACFGT--SWCRRFLNGQ
HsapiDIA1      30 LHSPS----LLASWQRNELTDRRFLQLN------------KCPACFGT--SWCRRFLNGQ
MmulaDIA1      30 LHSPS----LLASWQRNELTDRRFLQLN------------KCPACFGT--SWCRRFLNGQ
PvampDIA1      30 LHSPS----LLASWQRNELADRRFLQLN------------KCPACFGT--SWCRRFLNGQ
MdomeDIA1      30 LHSPS----LLASWQRNELADRRFLQLN------------KCPACFGT--SWCRRFLNGQ
MmuscDIA1      30 LHSPS----LLASWQRNELADRRFLQLN------------KCPACFGT--SWCRRFLNGQ
RnorvDIA1      30 LHSPS----LLASWQRNELADRRFLQLN------------KCPACFGT--SWCRRFLNGQ
GgallDIA1      30 LNTHS----LLASLQRNELAERRFLQLN------------KCPACWGT--SWCRKFLNGQ
XtropDIA1      30 LNTHS----LLSSFQRNELTDRRFLSLN------------KCPACFGT--SWCRKFMNGQ
BflorDIA1      30 FFYCF----VFQPFSYNQLTDTTFLGED------------KCPACFGT--DLCEEFENGK
SsalaDIA1R     33 VVEPAGT--PPAPQQKALILQRAFLGLD------------KCNACVGT--SICKKLLKDQ
DreriDIA1R     19 TADPS-----PAPQDKSHDFRKIFLGLD------------KCNACIGT--SICKKFFKDE
RnorvDIA1R     37 SLPPSL----VPRVRSSYTLGKTFLGLD------------KCNACIGT--SICKKFFKEE
MmuscDIA1R     37 SLPPSL----VPRVRSSYTMGKTFLGLD------------KCNACIGT--SICKKFFKEE
MmulaDIA1R     35 SSPSSL----VSQVRTSYNFGRTFLGLD------------KCNACIGT--SICKKFFKEE
HsapiDIA1R     35 SSLSSL----VPQVRTSYNFGRTFLGLD------------KCNACIGT--SICKKFFKEE
BtaurDIA1R     35 SPSPSP----VSRVRTSYNLGKTFLGLD------------KCNACIGT--SICKKFFKEE
EcabaDIA1R     35 SPSPSL----VPRVRTSYNFGRTFLGLD------------KCNACIGT--SICKKFFKEE
DordiDIA1R     35 SPPPSL----VSRVRTSYNLGRTFLGLD------------KCNACIGT--SICKKFFKEE
MdomeDIA1R     34 LTAPSS----VPRVKSSYNFGRTFLGLD------------KCNACIGT--SICKKFFKEE
OanatDIA1R     34 SPAPSL----VPRVKPSYSFGRTFLGLD------------KCNACIGT--SICKKFFKEE
GgallDIA1R     33 TASPS-----APHVRPSYSFGRTFLGLD------------KCNACIGT--SICKKFFKEE
BflorDIA1Lb   121 NPENQHSWKAIEKMAAHDEIEDHKKQPEQYMTFKRLMDAKKCPACYGES--LCEQAEVG-
BflorDIA1Lc     1 ----------MVIADGDNDIDISWAEPE------------KCPACFGD---KCELLRRGH
AaegyDIA1      25 PEERA-------SSLKSLVQQCQYDTTI------------LCPGCFPRP-VNCSFFENLK
CpipiDIA1      27 PKEPE-------SLTELLVNQCEYETQH------------LCPECFVSPTVNCGHLANFV
DyakuDIA1      22 VKPQ-----------REVFQELFEKDLR------------LCPNCFFGQRELCGEIFQKI
DerecDIA1      22 VKPQ-----------REVFQELYEKDLR------------LCPDCFVGQSELCVGIFQKI
DmelaDIA1      22 VKPQ-----------REVFQELFEKDLR------------LCPDCFVGQSEPCGEIFQKI
DsechDIA1      22 VKTQ-----------REVFQELFEKDLR------------LCPDCFVGQKEQCGEIFQKI
DpersDIA1      22 VKPQ-----------KDIFRAHLERDLQ------------LCPACFAGQRWQCEEIFEAI
DpseuDIA1      22 VKPQ-----------KDIFRAHLERDLQ------------LCPACFAGQRWQCEEIFEAI
DananDIA1      22 VKPQ-----------KDIFQEHFESDLR------------LCPACFSAQREQCEEVFRAI
DwillDIA1      22 VRTQ-----------KDIFRKHFNMDLL------------ECPQCFLGQREQCEKFFDTI
NvitrDIA1      21 GIYI-----------NRFNLKVAEITER-----------YKCPACFGD--SMCQVIDSNE
SpurpDIA1L    100 GDETALTQDGVQGMKNATVVMTTRLQFEN--LFPSLLEEERCPLCYGT--TNCDQIYAGN
BflorDIA1La    92 KHPAEEP-KEKPRQDKSAVTERKPKGVPRSLLRN-----APCPACLGD--NLCEEFEDGM
CinteDIA1      32 EMYNHRSQGSTRVMLEAKLLSDKWSATSP---------TCGNFSCFGT--SLCKNRNRVG
AgambDIA1      22 PNEKS--------TSKRPLENVCDYDRN-----------ILCPECVSSG-LECNKFDDLF
consensus     121                       fl l            c aC g     C

                                     Motif 1                  Motif 2
```

```
SpurpDIA1      74 YRFHSYSSIR-------LFDYVN-VKNVYFATYLD----QKP-VVMKKLGHNSEHNQFDK
NvectDIA1      80 VTVKSN---T-------PWSEER-IKGVSYGLWGA----VKQRVVLKTLGTSSELTAFDN
GaculDIA1      72 VSFETWGRLR-------FLDVFN-VKNVYFAQYGEPREGTRR-VVLKRLGSNQELAEIDQ
OlatiDIA1      72 VSFETWGRLR-------FLDVFN-VKNVYFAQYGEPREGTRR-VVLKRLGSNQELAEIDQ
TnigrDIA1      72 VSFETWGRLR-------FLDVFN-VKNVYFAQYGEPREGTRR-VVLKRLGSNQELADIDQ
TrubrDIA1      72 VSFETWGRLR-------FLDVFN-VKNVYFAQYGEPREGTRR-VVLKRLGSNQELADIDQ
DreriDIA1b     72 ISFETWGRLR-------FLDVFN-VKNVFFAQYGEPREGTRR-IVLKRLGSNQELADIDQ
DreriDIA1a     72 VTFEMWGRLR-------FLDFFN-VKNVFFAQYGEPREGTRR-VVLKRLGSNQELAEIDQ
BtaurDIA1      72 VVFEAWGRLR-------LLDFLN-VKNVYFAQYGEPREGGRRRVVLKRLGSQRELAQLDQ
PpygmDIA1      72 VVFEAWGRLR-------LLDFLN-VKNVYFAQYGEPREGGRRRVVLKRLGSQRELAQLDQ
CfamiDIA1      72 VVFEAWGRLR-------LLDFLN-VKNVYFAQYGEPREGGRRRVVLKRLGSQRELAQLDQ
PtrogDIA1      72 VVFEAWGRLR-------LLDFLN-VKNVYFAQYGEPREGGRRRVVLKRLGSQRELAQLDQ
TtrunDIA1      72 VVFEAWGRLR-------LLDFLN-VKNVYFAQYGEPREGGRRRVVLKRLGSQRELAQLDQ
HsapiDIA1      72 VVFEAWGRLR-------LLDFLN-VKNVYFAQYGEPREGGRRRVVLKRLGSQRELAQLDQ
MmulaDIA1      72 VVFEAWGRLR-------LLDFLN-VKNVYFAQYGEPREGGRRRVVLKRLGSQRELAQLDQ
PvampDIA1      72 VVFEAWGRLR-------LLDFLN-VKNVYFAQYGEPREGGRRRVVLKRLGSQRELAQLDQ
MdomeDIA1      72 VAFEAWGRLR-------LLDFLN-VKNVYFGQYGEPREGGRRRVVLKRLGSQRELAQLDQ
MmuscDIA1      72 VGFETWGRLR-------LLDFLN-VKNVYFAQYGEPREGGRRRVVLKRLGSQRELAQLDQ
RnorvDIA1      72 VGFETWGRLR-------LLDFLN-VKNVYFAQYGEPREGGRRRVVLKRLGSQRELAQLDQ
GgallDIA1      72 LRLESWGRLR-------LFDFFN-VKNVYFARYGEPREGSRR-VVLKRLGSAQELADIDA
XtropDIA1      72 LSFEGWGRLR-------LLDFFN-VKNVHFAQYGEPREGSRR-VVLKRLGSNHELSELDQ
BflorDIA1      72 ILFKYSSRLR-------ILDIFN-IKNVYFAIY----EGME--VALKRLGHNSEFDQLDR
SsalaDIA1R     77 IRFDWWMSPD-------TTLPLA-EKQSFPGNLTDDSLSWRP-VVLSFLSSPCLHSSSDR
DreriDIA1R     60 IRFERRLTAQ-------SNLSSA-DVRSYEGNYTDSTAGWRP-VVVSRLMSPHLHQLSDN
RnorvDIA1R     79 IRLDNSLVSH-------LGLPSQ-DLLSYAANYSDDSKTWRP-VEISRLVSKYQSEISDK
MmuscDIA1R     79 IRFDNSLASH-------LGLPPQ-DLHSYAANYSDDSKTWRP-VEISQLVSRYQIEISDR
MmulaDIA1R     77 IRSDNWLASH-------LGLPPD-FLLSYPANYSDDSKIWRP-VEIFRLVSNYQNEISDR
HsapiDIA1R     77 IRSDNWLASH-------LGLPPD-SLLSYPANYSDDSKIWRP-VEIFRLVSKYQNEISDR
BtaurDIA1R     77 IRFDNGLALH-------LGPPPD-DLPSYSANYSDDFKTWRP-VEISRLVSKQQNKISDG
EcabaDIA1R     77 IRFDNRLASH-------LGLPSG-YLPSYSANYSDDSKTWRP-VEVSRLVSKQQNEISDR
DordiDIA1R     77 IRFDNSLASD-------LELASD-YLPSYSANYSDDSKTWRP-VEIFRLVSKEQNEISDR
MdomeDIA1R     76 IRFDNWLASH-------LKLPPD-YFSTYPANYSDDTKSWRP-VEISRLISKYQHELSDR
OanatDIA1R     76 IRFDNWPASH-------LKPPPD-HFPGYSANYTDDSKSWRL-VEISRLIGKDQNELSDQ
GgallDIA1R     74 IRFDTWLSSH-------LKLPPS-YLLSYLGNYTDDAQSWRM-VDITRLTTKYQHDRADQ
BflorDIA1Lb   178 LITMDVADKT-------LEHKG--VYFGHFRNT-----E----VVAKRLVGKDGWTRFDE
BflorDIA1Lc    36 FVKVDSESDR-------YMKKGI-VSTGRIGGV-----K----VIAKSMNEAGAWQRYER
AaegyDIA1      65 SIDS----NE-------LFEELL-THFNQHGVRYGTLSGSEQRVVIKNLNKEHGVEELIS
CpipiDIA1      68 TIDS----SN-------PWNQLT-CRYNPHGVSYG-LLGSGQKVVIKTLNKNRAVEALRD
DyakuDIA1      59 AEPSDW--SK-------LLKAIS-LLVDRRVIYFLRFKDQDQ-----VVAKRKIIDAQNQ
DerecDIA1      59 AEPSDW--SK-------LLKAIS-LIVDRRAIYYLRLKDQAQ-----VVAKRKIIDARNH
DmelaDIA1      59 AEPSDW--SR-------LVKAIS-LLVDRRAIYFLRLKDRDQEV-Q-LVAKRKIIDAQSN
DsechDIA1      59 AEPSDW--SR-------LVKAIS-LLVDRRAIHYLRLKDQDQAV-Q-LVAKRKIINAHSY
DpersDIA1      59 AEPSDW--SK-------LLKGIA-LLIDRREIYFLQLNERHQAA-Q-LVAKRDAIGGHSY
DpseuDIA1      59 AEPSDW--SK-------LLKGIA-LLIDRREIYFLQLNERHQAA-Q-LVAKRDAIGGHSY
DananDIA1      59 REPSDL--SK-------LWKAVT-LLFDRREIFWLEIGPSQEKSSQ-LLAKRKSIVASHQ
DwillDIA1      59 EEPAKL--DR-------LWKDLT-LLVDPREIYWLELNENREN-VK-CLAKRKMTRKISK
NvitrDIA1      57 ISFEYTDFYS-------IFNNLFSVKNVYYGKYKD------KKVIMKKLAQESELKTFDE
SpurpDIA1L    156 ISFHVG---S-------VNLSEP---HAIRGTWGD------RRIVGKRLVSREVFERLEK
BflorDIA1La   144 IDLG--------------SEVTSWKVKAYTGTWD------KVEVMVTQCASEERLERFEE
CinteDIA1      81 LSEDVPTLRSGWLSIYGELVDLHRVKLNVTYKLPSTLPCNGETCSVQIETSVLSGNNVNK
AgambDIA1      62 TLQDDN-----------VFNIIN-NIFNSHTTRIGTIVQENKLAVMKHLNRDNTIEKLLQ
consensus     181                               a y              l
```

Motif 3

```
SpurpDIA1     121 DLCTNAES-GLRGCDVSQQIYKSKLS------DIMYAERLEEKDVMG---L-SDIVRCPS
NvectDIA1     125 EICRGVGK-DSN-CKISQEAWDS---------DVLVPKPPLQDAVR-------KYFDCPS
GaculDIA1     123 KICKRATG-RPR-CDLIQAMYKTEFA------RINGDVRLLTPEVVEG--W-SDLVHCPS
OlatiDIA1     123 KICKRATG-RPR-CDLVQAMYKTEFA------RINGDVRLLTPDVVEG--W-SDLVHCPS
TnigrDIA1     123 KICKRATG-RPR-CDLIQAMYKTEFA------RINGDVRLLTPDVVEG--W-SDLVHCPS
TrubrDIA1     123 KICKRATG-RPR-CDLIQAMYKTEFA------RINGDVRLLTPEVVEG--W-SDLVHCPS
DreriDIA1b    123 KICKRATG-RPR-CDLIQAMYKTEFA------RLNGDVRLLTPDVVEG--W-SDLVHCPS
DreriDIA1a    123 KICKRATG-RPR-CDLIQSMYKTEFA------RLNGDVRLLTPEVVEG--W-SDLVHCPS
BtaurDIA1     124 SICKRATG-RPR-CDLLQAMPRTEFA------RLNGDVRLLTPEAVEG--W-SDLVHCPS
PpygmDIA1     124 SICKRATG-RPR-CDLLQAMPRTEFA------RLNGDVRLLTPEAVEG--W-SDLVHCPS
CfamiDIA1     124 SICKRATG-RPR-CDLLQAMPRTEFA------RLNGDVRLLTPEAVEG--W-SDLVHCPS
PtrogDIA1     124 SICKRATG-RPR-CDLLQAMPRTEFA------RLNGDVRLLTPEAVEG--W-SDLVHCPS
TtrunDIA1     124 SICKRATG-RPR-CDLLQAMPRTEFA------RLNGDVRLLTPEAVEG--W-SDLVHCPS
HsapiDIA1     124 SICKRATG-RPR-CDLLQAMPRTEFA------RLNGDVRLLTPEAVEG--W-SDLVHCPS
MmulaDIA1     124 SICKRATG-RPR-CDLLQAMPRTEFA------RLNGDVRLLTPEAVEG--W-SDLVHCPS
PvampDIA1     124 SICKRATG-RPR-CDLLQAMPRTEFA------RLNGDVRLLTPEAVEG--W-SDLVHCPS
MdomeDIA1     124 SICKRATG-RPR-CDLLQAMPKTEFA------RLNGDVRLLTPDAVEG--W-SDLVHCPS
MmuscDIA1     124 SICKRATG-RPR-CDLLQAMPRTEFA------RLNGDVRLLTPEAVEG--W-SDLVHCPS
RnorvDIA1     124 SICKRATG-RPR-CDLLQAMPRTEFA------RLNGDVRLLTPEAVEG--W-SDLVHCPS
GgallDIA1     123 KICRRATG-RGR-CDLLQALHATEFA------SLNGDVRLLTPDAVEG--W-SDLVHCPS
XtropDIA1     123 RICKKATG-RPR-CDLVQAMYKTDFA------RLNGDVRLLTPDVVEG--W-SDLVHCPS
BflorDIA1     118 WICETGKE-GPQ-CHIPSVTYYSNFS------RVIYEG--LSPKSLGD--M-SDMVRCPS
SsalaDIA1R    128 SICRSVGR-QGP-CSIEAVLRVTPRF------QSLNQSHLLLPHIVKG--LAPPLLRCPS
DreriDIA1R    111 SICTSAGK-GKS-CSIEGVLRATSRF------QTWVHSNLLLPSMVKG--LVTPMLRCPS
RnorvDIA1R    130 RICASASG-PKT-CSIERILRKTGRF------QKWLQAKRLTPDLVQG--LPSPFLHCPS
MmuscDIA1R    130 RICASVSA-PKT-CSIERILQKTGRF------QKWLQAKRLTPDLVQG--LPSPFLRCPS
MmulaDIA1R    128 RICASASA-PRT-CSIERVLRKTERF------QKWLQAKRLTPDLVQG--LASPLLRCPS
HsapiDIA1R    128 RICASASA-PKT-CSIERVLRKTERF------QKWLQAKRLTPDLVQG--LASPLLRCPS
BtaurDIA1R    128 RICASAAA-PKT-CSIERVLRKTGRF------QKWLQAKRLTPDLVRG--LSSPLLRCPS
EcabaDIA1R    128 RICAFAAA-PKT-CSIERVLRKTRRF------QKWLQAKRLTPDLVRG--LPSPLLRCPS
DordiDIA1R    128 RICAFASA-PKT-CSIERILRKTGRF------QKWLQAKRLTPDLVQG--LPSPFLRCPS
MdomeDIA1R    127 RICASASG-SKT-CSIERVLRKTERV------KKWLKAKRLTPDLVQG--LPSPMLRCPS
OanatDIA1R    127 KICTSVSL-VKT-CSIERVLRKTERF------QKWLKARRLTPALVQG--LPSLLLRCPT
GgallDIA1R    125 HICTSLLK-TKT-CSLERALRRTHRF------QKWLRAKRLTPDLVQG--LSSPMLRCPS
BflorDIA1Lb   220 FICQNASL-PKD-CDVSHMISDTVLVTDNVLQVSFLQDAWRIAHTRRS--I--AMEACMT
BflorDIA1Lc    79 FICRSSSR-PHV-CNASSFILETMLVTDVALKVPWLREAWKICHLEKS-----ALSLCVS
AaegyDIA1     113 SICNDLKI-YR-NCRLRNKEPYLRVL--------------RQRVLNDN--QLEGAILCPL
CpipiDIA1     115 AVCDELGL-TQSNCKFKSDENTLKVL--------------RRKVL-EQ--ELEGCIICPS
DyakuDIA1     104 Q-IKDVKK-AF--------------------------------YELGE--RPGGFHLCRT
DerecDIA1     104 K-AENVEK-AF--------------------------------YEQEE--QPGGFHLCRT
DmelaDIA1     107 Q-IKNVKK-DF--------------------------------YELEE--RPGGFHLCRT
DsechDIA1     107 Q-MKNVKR-AF--------------------------------YELEE--RPGGFHLCRT
DpersDIA1     107 RNMGILRE-AF--------------------------------LEMEE--RPGGFQLCRS
DpseuDIA1     107 RNMGILGE-AF--------------------------------LEMEE--RPGGFQLCRS
DananDIA1     108 S-LSSLQE-EF--------------------------------LNLEE--RPGGFHLCRT
DwillDIA1     107 D--------------------------------------------LHF--NPGGFQMCRN
NvitrDIA1     104 MICSDDEL-HELCYQSESRKQDSKTN-------FYKLVESELANVGGD--INNKMRLCPT
SpurpDIA1L    197 IICNQSQVDPKRSCEVNAAATNSWMSKS----SALNRVHKLHQEVYESQLIAISATTCAS
BflorDIA1La   184 FVCRNLSETATS-CDPGKVLLQEDLK------------------ERLQP----AHLKKSLR
CinteDIA1     141 WICDKFKKPTN--CDTGKVFKSWRAMN-----KITSAKGRLDPNLLKD--I-SPPLQCPS
AgambDIA1     110 EYCDAYSLTKQN-CRWAQHDGSVDR-----------AKEFITTSILDS-ARVEGCIFCPA
consensus     241  ic          c                               v           c s
```

Motif 4   Motif 5 …

```
SpurpDIA1     170 RRLLDRIWDTFGERQFDKS-----LARDHKLMLATTIAFNPEPLILQAFP----NRKGWP
NvectDIA1     167 TRFLEKIESVYG-QETPGQ-----LSLVETIHLITGLHLNPELLLLQIFR----QEEGWP
GaculDIA1     172 QRLLDRVVRRYAETKDSGSFLLKNLKDTERMQLLMTLAFNPEPLVLQSFP----SDEGWP
OlatiDIA1     172 QRLLDRVVRRYAETKDSGSFLLKNLKDTERMQLLMTLAFNPEPLVLQSFP----SDEGWP
TnigrDIA1     172 QRLLDRVVRRYAETKDSGSFLLKNLKDTERMQLLMTLAFNPEPLVLQSFP----SDEGWP
TrubrDIA1     172 QRLLDRVVRRYAETKDSGSFLLKNLKDTERMQLLMTLAFNPEPLVLQSFP----SDEGWP
DreriDIA1b    172 QRLLDRIVRRYAETKDSGSFLLKNLKDTERMQLLMTLAFNPEPLVLQSFP----SDEGWP
DreriDIA1a    172 QRLLDRVVRRYAETKDSGSFLLKNLKDTERMQLLMTLAFNPEPLVLQSFP----SDEGWP
BtaurDIA1     173 QRLLDRLVRRYAETKDSGSFLLRNLKDSERMQLLLTLAFNPEPLVLQSFP----SDEGWP
PpygmDIA1     173 QRLLDRLVRRYAETKDSGSFLLRNLKDSERMQLLLTLAFNPEPLVLQSFP----SDEGWP
CfamiDIA1     173 QRLLDRLVRRYAETKDSGSFLLRNLKDSERMQLLLTLAFNPEPLVLQSFP----SDEGWP
PtrogDIA1     173 QRLLDRLVRRYAETKDSGSFLLRNLKDSERMQLLLTLAFNPEPLVLQSFP----SDEGWP
TtrunDIA1     173 QRLLDRLVRRYAETKDSGSFLLRNLKDSERMQLLLTLAFNPEPLVLQSFP----SDEGWP
HsapiDIA1     173 QRLLDRLVRRYAETKDSGSFLLRNLKDSERMQLLLTLAFNPEPLVLQSFP----SDEGWP
MmulaDIA1     173 QRLLDRLVRRYAETKDSGSFLLRNLKDSERMQLLLTLAFNPEPLVLQSFP----SDEGWP
PvampDIA1     173 QRLLDRLVRRYAETKDSGSFLLRNLKDSERMQLLLTLAFNPEPLVLQSFP----SDEGWP
MdomeDIA1     173 QRLLDRLVRRYAETKDSGSFLLRNLKDSERMQLLLTLAFNPEPLVLQSFP----SDEGWP
MmuscDIA1     173 QRLLDRLVRRYAETKDSGSFLLRNLKDSERMQLLLTLAFNPEPLVLQSFP----SDEGWP
RnorvDIA1     173 QRLLDRLVRRYAETKDSGSFLLRNLKDSERMQLLLTLAFNPEPLVLQSFP----SDEGWP
GgallDIA1     172 QRLLDRLVRRYAETKDSGSFLLRNLKDSERMQLLITLAFNPEPLVLQSFP----SDEGWP
XtropDIA1     172 QRLLDRLVRRYAETKDSGSFLLRNLKDTERMQLLLTLAFNPEPLVLQSFP----SDEGWP
BflorDIA1     165 QRLIDRVLEKFAEHLGKET---EELSYQEKLHFLSTLKFNPEPLMLQTFP----ITEGWP
SsalaDIA1R    178 QRLLDRIVRRYAEVVDVGSVQMKHFSERDKLRLLYTLAVNQQPLILQMFP----GTEGWP
DreriDIA1R    161 QRLLDRIVRRYFEVTDVGSVQMKHFNEKDKLRLLYTLAVNQQPLILQMFP----GTEGWP
RnorvDIA1R    180 QRLLDRVVRRYAEVVDAGSIFMDHFTDRDKLRLLYTLAVNAHPIILQIFP----GAEGWP
MmuscDIA1R    180 QRLLDRVVRRYAEVVDAGSIFMDHFTAGDKLRLLYTLAVNAHPIILQIFP----GAEGWP
MmulaDIA1R    178 QRLLDRVVRRYAEVADAGSIFMDHFTDRDKLRLLYTLAVNSHPILLQIFP----GAEGWP
HsapiDIA1R    178 QRLLDRVVRRYAEVADAGSIFMDHFTDRDKLRLLYTLAVNSHPILLQIFP----GAEGWP
BtaurDIA1R    178 QRLLDRVVRRYAEVADAGSIFMDHFTDRDKLRLLYTLAVNTHPVLLQIFP----GAEGWP
EcabaDIA1R    178 QRLLDRVVRRYAEVPDAGSIFMDHFTDRDKLRLLYTLAVNAHPILLQIFP----GAEGWP
DordiDIA1R    178 QRLLDRVVRRYAEVADAGSIFMDHFTDRDKLRLLYTLAVNAHPIFLQIFP----GAEGWP
MdomeDIA1R    177 QRLLDRIVRRYAEVADAGSVFMDHFTDRDKLRLLYTLSINAHPIMLQIFP----GAEGWP
OanatDIA1R    177 QRLLDRIVRRYAEVPDAGSVYMDHFTDRDKLRLLYTLSVNAHPVLLQVFP----GAEGWP
GgallDIA1R    175 QRLLDRIVRRYAEVPDAGSIYMDHLTDQDKLRLLYTLSVNSHPILLQIFP----DVEGWP
BflorDIA1Lb   274 DRLIELIKTAYDENVNGKLSK------TERAYMITALLLNPEAALLKHFTS--RAEEYWP
BflorDIA1Lc   132 DRFLEDVRQLYVEDGGTDMTKE---GDTGRAFLSTSLLLNEEAVLLRYFTT--KSTTPWP
AaegyDIA1     155 MDEE--ALKRFLFEIDEN-----------DLNKILLMRINAQPLLLKLLQ-----ERNFP
CpipiDIA1     157 KDEK--ALNRLLNEVEGT-----------ELLQLILLKVNVQPLLLELFD-----GRGFP
DyakuDIA1     128 PSRKP-RFVSYLEQRGHA-----------SASVWFYMMHYVSPLLMQELY-----LQGFP
DerecDIA1     128 PSRKPPRFVSYLEQRGHA-----------SASVWFYMMHYVSPLLMQELH-----LQGFP
DmelaDIA1     131 LAREP-RFVSYLEQRGHA-----------AASVWFYMMYYVSPLLMQELH-----LQGFP
DsechDIA1     131 LAREP-RFVSYLEQRGHVY---------GSTSVWFYMMHYVSPLLMQELH-----LQGFP
DpersDIA1     132 GFRTP-RFVSYLEQRGHE-----------AATIWYYMMHSITPLLMQELF-----LRGFP
DpseuDIA1     132 GFRTP-RFVSYLEQRGHE-----------AATIWYYMMHSITPLLMQELF-----LRGFP
DananDIA1     132 AQKSS-PFINYLNQRGYD-----------SSSIYFYMLHNISPLLMQELQ-----NLDFP
DwillDIA1     121 STR----FLAYLEERGYL-----------PATIGYYIHHSISPLLMQELW-----IMDFP
NvitrDIA1     154 TTKLEKLFVNVKFQSHAEN-------EDYFKYLWSTIKINPEPLILQILP----AKEGWP
SpurpDIA1L    253 PDFFEELKQLYRVSRWGTG-----IASTEMAILATTMALNPEPALLKFFRN--IPSLRPY
BflorDIA1La   222 EVFHPNTTSSLQATTCMS--------KPFLKLLQKTFDDNGNKRLNRLEQA--IPRKKLP
CinteDIA1     191 TRIADRIVRRVANKQGGVLLR--DLSYTEKKRLIFTCKMSPGSVVQSMFP----PYEGWL
AgambDIA1     157 TNNRQ-SLQRFLSLFDSTDN---------ELWNLLAVRTNVEPLLIKLLTVNYHDEPPLY
consensus     301           y e                   l   l  n  plvlq          gwp
```

… Motif 5 Motif 6 Motif 7 …

```
SpurpDIA1    221 FPAYMGACGRFTVQEYSG-HSLSYFYKFK--FGVRAALAVQALKIAEQLSENRDEFALYL
NvectDIA1    217 FPRFYGVCGRIMVVEDSG-PPISSFLEES--WDVRAQIAVNLITLAHQLSSALDDWALYI
GaculDIA1    228 FAKYLGACGRMVAVNYVG-EELWSFYNAP--WEKRVDLARQLMDIAEQLTNNDFDFALYL
OlatiDIA1    228 FAKYLGACGRMVAVNYVG-EELWNFYNAP--WEKRVDLARQLMDIAEQLTNNDFDFALYL
TnigrDIA1    228 FAKYLGACGRVVAVNYVG-EELWSFYNAP--WEKRVDLARQLMDIAEQLTNNDFEFALYL
TrubrDIA1    228 FAKYLGACGRVVAVNYVG-EELWSFYNAP--WEKRVDLARQLMDIAEQLTNNDFEFALYL
DreriDIA1b   228 FAKYLGACGRMVAVNYVG-EELWSFFNAP--WEKRVDLAKQLMDIAEQLTNNDFDFALYL
DreriDIA1a   228 FAKYLGACGRMVAVNYVG-EELWSFYNAP--WEKRVDLAKQLMDIAEQLTNNDFDFALYL
BtaurDIA1    229 FAKYLGACGRMVAVNYVG-EELWSYFNAP--WEKRVDLAWQLMEIAEQLTNNDFEFALYL
PpygmDIA1    229 FAKYLGACGRMVAVNYVG-EELWSYFNAP--WEKRVDLAWQLMEIAEQLTNNDFEFALYL
CfamiDIA1    229 FAKYLGACGRMVAVNYVG-EELWSYFNAP--WEKRVDLAWQLMEIAEQLTNNDFEFALYL
PtrogDIA1    229 FAKYLGACGRMVAVNYVG-EELWSYFNAP--WEKRVDLAWQLMEIAEQLTNNDFEFALYL
TtrunDIA1    229 FAKYLGACGRMVAVNYVG-EELWSYFNAP--WEKRVDLAWQLMEIAEQLTNNDFEFALYL
HsapiDIA1    229 FAKYLGACGRMVAVNYVG-EELWSYFNAP--WEKRVDLAWQLMEIAEQLTNNDFEFALYL
MmulaDIA1    229 FAKYLGACGRMVAVNYVG-EELWSYFNAP--WEKRVDLAWQLMEIAEQLTNNDFEFALYL
PvampDIA1    229 FAKYLGACGRMVAVNYVG-EELWSFFNAP--WEKRVDLAWQLMEIAEQLTNNDFEFALYL
MdomeDIA1    229 FAKYLGACGRMVAVNYVG-EELWSYFNAP--WEKRVDLAWQLMEIAEQLTNNDFEFALYL
MmuscDIA1    229 FAKYLGACGRMVAVNYVG-EELWSYFNAP--WEKRVDLAWQLMEIAEQLTNNDFEFALYL
RnorvDIA1    229 FAKYLGACGRMVAVNYVG-EELWSYFNAP--WEKRVDLAWQLMEIAEQLTNNDFEFALYL
GgallDIA1    228 FAKYLGACGRMVAVNYVG-EELWSYFNAP--WEKRVDLAWQLMEIAEQLTNNDFEFALYL
XtropDIA1    228 FAKYLGACGRMVAVNYVG-EELWSYFNAP--WEKRVDLAWQLMEIAEQLTNNDFDFALYL
BflorDIA1    218 FPFYFGACGRLTVVQKCD-KTLASYYSAP--WLKRVELSLQMMKIAEYLTNNEADFGLYL
SsalaDIA1R   234 FPRYQGSCGRLMVWASS--RPLWGLYGSSREFVQRVDVAYQLLHITQGLGHNSLGFLLYY
DreriDIA1R   217 FLRYHGSCGRLMVWAAS--RALRTLFSSP--LERRADLAYQLLHITQSLSANSLRFRLFY
RnorvDIA1R   236 MPKYLGSCGRFLVSTST--RPLQEFYDAP--PEQAADIAYQLLGVLESLRSNDLNYFFYF
MmuscDIA1R   236 MPRYLGSCGRFLVSTST--RPLQEFYDAS--PEQAADLAYQLLRVLESLRSNDLNYFFYF
MmulaDIA1R   234 LPQYLGSCGRFLVSTST--RPLQEFYGAP--PDQAADLAYQLLGVLESLRSNDLNYFFYF
HsapiDIA1R   234 LPKYLGSCGRFLVSTST--RPLQEFYDAP--PDQAADLAYQLLGVLESLRSNDLNYFFYF
BtaurDIA1R   234 LPQYLGSCGRFLVSTST--SPLQEFYGAP--PDQAADLAYQLLGVLESLRSNDLNYFFYF
EcabaDIA1R   234 LPRYLGSCGRFLISTST--SPLQEFYSAP--PDQAADLAYQLLGVLESLRSNDLNYFFYF
DordiDIA1R   234 LPKYLGSCGRFFVSTST--RPLQEFYGAP--PDEAADLAYQLLGVLESLRSNDLNYFFYF
MdomeDIA1R   233 LPRYLGSCGRFLVSTST--QPLEEFYSSP--ADQAADLAYQLLGILDSLRNNDLNYFFYF
OanatDIA1R   233 FPKYLGSCGRLLVSTST--TPLLELSSGP--PDRAADLGHQLLGVIEFLRHNDWNYFFYF
GgallDIA1R   231 FPRYLGSCGRLVVSAST--RPLRDFFRAA--PEVAADLALQLLAVLHSMGTNDLNYFFYF
BflorDIA1Lb  326 FPKYLGACGRVILVESGG-KLLGSAIESP--WKERANIALQLLEMIDKFRNGDPKWVVIF
BflorDIA1Lc  187 FPKFYGACGRVIVVEHAG-RTLDTFMESS--WEVRADIALQLLQLVDALREKDPDWVLFF
AaegyDIA1    197 VPKLIFQGGFTLVESYEG-EALAKYYDRP--LNVRLLIANELIKASLNFTAGVDNFRFYL
CpipiDIA1    199 VPKVIFQGGFQLFESFDG-DALVNFYDSS--LNIRLRIAKELIQASFLFTEGVNGFRFYL
DyakuDIA1    171 VPTTYASCGLTHFQSYAG-RTLTNYFEGE--EDLRVELALQLMQLSLKLTFGFSDFRIML
DerecDIA1    172 VPTTYASCGLTHFQAYAG-RTLTNYFEAE--EGLRMELALQLIQLSLKLTFGFSDFRIFL
DmelaDIA1    174 VPTTYASCGLTHFQSNAG-RTLAHYVDAE--EGLRVELALQLIQLSLKLTFGFADFRIIL
DsechDIA1    176 VPTPYASCGLTHFQSYAG-RTLAHYVNAE--EGLRLELALQLIQLSLKLTFGFADFRIIL
DpersDIA1    175 VPRSYAACGLTHFQAYAG-RTLVHYATAG--ENLRLEMARQLLQLSLKLTFGFADFRIFL
DpseuDIA1    175 VPRSYAACGLTHFQAYAG-RTLVHYATAG--ENLRLEMARQLLQLSLKLTFGFADFRIFL
DananDIA1    175 VPNTYAICGLTHFQAYAG-RTLNHYTEAA--EDLRREIARQLIHLSLKLTFGFADFRIFL
DwillDIA1    161 VPRSFAVCGSTLFQAYAG-STLSNYLKAP--IDLRLEMAKQLLQLALKLSAGFKGFRIYL
NvitrDIA1    203 TPKYFGACGRLIVEEYIG-LPLSSFIDEP--WIRRAKIASSLLQAADTLMSKNSEFAFYL
SpurpDIA1L   306 FTEYLGECGRVILTEPSG-KPLSSYLKAS--WKDRVDISLKILQMIEDFHDSSDKWLVLL
BflorDIA1La  272 IPAFLGACGNLVATETAG-KPLSMYLEVKGPWQVRANLSLQLLQMLDDFQNKDPDWLLMF
CinteDIA1    245 FQRTVGSCGALMVHGRSIVTSLYELYDAP--WKVRLDVAIQLLDLAEHFTYNDLNYMFAL
AgambDIA1    207 VPKLLHTFGFSIIETYEG-KTLEHYYDFP--FVTRMRIAAELIRAGFKFTEGIHGFRVFL
consensus    361      g cG  m         l  y         r dla qlm  i   l      f ly
```

… Motif 7 Motif 8 Motif 9 Motif 10 …

```
SpurpDIA1      278 TDVSEDNLAVND-NG----EVLVIDAENIIVVDRKKIKEDANPGWDVKH-----QSEHEG
NvectDIA1      274 ADPAPGNFGVSD-SG----KVTLLDLEHLVVVDLSEVQMEEANDKPKPS----------D
GaculDIA1      285 LDVSFDNFAVGPRDG----KVIVVDAENVLVADKRLIKQNKPESFDVWY-----ESRFEE
OlatiDIA1      285 LDVSFDNFAVGPRDG----KVIVVDAENVLVADKRLIKQNKPESYDVWY-----ESRFEE
TnigrDIA1      285 LDVSFDNFAVGPRDG----KVIIVDAENVLVADKRLIKQNKPENFDVWY-----ESRFEE
TrubrDIA1      285 LDVSFDNFAVGPRDG----KVIIVDAENVLVADKRLIKQNKPENYDVWY-----ESRFEE
DreriDIA1b     285 LDVSFDNFAVGPRDG----KVIVVDAENVVVADKRLIKQNKPESYDVWY-----ESRFEE
DreriDIA1a     285 LDVSFDNFAVGPRDG----KVIVIDAENVIVVDKRLVKQNKPESYDVWY-----ESRYEE
BtaurDIA1      286 LDVSFDNFAVGPRDG----KVIIVDAENVLVADKRLIRQNKPENWDVWY-----ESKFDD
PpygmDIA1      286 LDVSFDNFAVGPRDG----KVIIVDAENVLVADKRLIRQNKPENWDVWY-----ESKFDD
CfamiDIA1      286 LDVSFDNFAVGPRDG----KVIIVDAENVLVADKRLIRQNKPENWDVWY-----ESKFDD
PtrogDIA1      286 LDVSFDNFAVGPRDG----KVIIVDAENVLVADKRLIRQNKPENWDVWY-----ESKFDD
TtrunDIA1      286 LDVSFDNFAVGPRDG----KVIIVDAENVLVADKRLIRQNKPENWDVWY-----ESKFDD
HsapiDIA1      286 LDVSFDNFAVGPRDG----KVIIVDAENVLVADKRLIRQNKPENWDVWY-----ESKFDD
MmulaDIA1      286 LDVSFDNFAVGPRDG----KVIIVDAENVLVADKRLIRQNKPENWDVWY-----ESKFDD
PvampDIA1      286 LDVSFDNFAVGPRDG----KVIIVDAENVLVADKRLIRQNKPENWDVWY-----ESKFDD
MdomeDIA1      286 LDVSFDNFAVGPRDG----KVIIVDAENVLVADKKLIRQNKPENWDVWY-----ESKFDD
MmuscDIA1      286 LDVSFDNFAVGPRDG----KVIIVDAENVLVADKRLIRQNKPENWDVWY-----ESKFDD
RnorvDIA1      286 LDVSFDNFAVGPRDG----KVIIVDAENVLVADKRLIRQNKPENWDVWY-----ESKFDD
GgallDIA1      285 LDVSFDNFAVGPRDG----KVIIVDAENVLVADKRLIRQNKPENWDVWY-----ESKFDD
XtropDIA1      285 LDVSFDNFAVGPRDG----KVIIVDAENVLVADKKLIKQNKPENWDVWY-----ESKFDD
BflorDIA1      275 TDISYENFGVTS-DG----NLFVIDVENVIVVDKQKIKADKPRNWEARY-----QSHFDE
SsalaDIA1R     292 TRLGEDMFGLLD-DQ----RVFITDASSIGVIDLEQGFPPDPPS-QTGS-----DGDIFS
DreriDIA1R     273 TRIPEDMFGILE-DN----KVFIVDTSTIGIIDLQEGHPPDK-D-LLPE-----ELDVFS
RnorvDIA1R     292 THVDAGMFGIFD-NG----HLFIRDASALGIIDKQEASQAAD---RTGE-----NEDIFS
MmuscDIA1R     292 THVDAGMFGIFD-NG----HLFIRDASALGIIDKQEGSQAAA---RTGE-----NEDIFS
MmulaDIA1R     290 THIDAGMFGVFN-NG----HLFIRDASAVGVIDKQEGSQEAT---RAGE-----NKDIFS
HsapiDIA1R     290 THIDAGMFGVFN-NG----HLFIRDASAVGVIDKQEGSQEAN---RAGE-----NKDIFS
BtaurDIA1R     290 THVDADMFGIFN-NG----HLFIRDASALGVIDRQEGSQAAS---GAGD-----NKDIFS
EcabaDIA1R     290 THVDAGMFGIFN-NG----HLFIRDASTLGVIDRQEGSQAAA---RAGE-----NKDIFS
DordiDIA1R     290 THVDAAMFGIFS-NG----HLFIRDASALGVIDKQEGSQAAP---KAGE-----NQDIFS
MdomeDIA1R     289 TRIDAGTFGIFD-NG----HLFIRDASTVGVIDKQRGSLTLD---RQEE-----SKDIFS
OanatDIA1R     289 TRIHAGTFGVFD-NG----HLFIRDASSLGVIDRQEGKRPAR---SSPE-----PRDIFS
GgallDIA1R     287 TRVDVGTFGVFS-NG----HLFIRDASTLGIIDKEEGSQPID---GQQE-----YKDIFS
BflorDIA1Lb    383 VDFSFNNFAVNN-YG----RLTLIDFDDVMLIDREEFVGENK--TEPCD-----LKCFKT
BflorDIA1Lc    244 LDVSFQNFAVDS-RG----WVRLIDLDDVMVIDRRTVVNQEQ--TEMCN-----EQCYTD
AaegyDIA1      254 TDISPDNIVVSMSSEEE-IKVTFVDLNNVIILDSHSKRLKP------SK-----QKHVHS
CpipiDIA1      256 TDINPDNIAVQAQPSGS-FQVSFIDLDNVIILDSQSKRLDRR-----SK-----ARNIHS
DyakuDIA1      228 TDFTGDNFAYDEHTK----KVYLIDLDSVVLVD----ASSAT-----GQ-----AEKYEP
DerecDIA1      229 TDFTGDNFAYDEDSK----RVYLIDLDSVVLVD----ASSAA-----GQ-----AEKYVP
DmelaDIA1      231 TDFTADNLAYDEDTK----KVYLIDLDSVVLVD----ASFAA-----GH-----AEKYEP
DsechDIA1      233 TDFTADNLAYDEETK----KVYLIDLDSVVLVD----ASSMA-----GQ-----AEKYEP
DpersDIA1      232 TDFTADNLAFDEASQ----SVILIDLDSVVLVD----AAVPL-----GD-----AQKYEP
DpseuDIA1      232 TDFTADNLAFDEASQ----SVILIDLDSVVLVD----AAVPL-----GD-----AQKYEP
DananDIA1      232 TDFTSDNLTFDEDTR----RVLLIDLDSVVMVD----AASTS-----GQ-----AEKYEP
DwillDIA1      218 TDFTADNFAYNEANG----TVLLIDMDTMVLVESNDEAESES-----QL-----SKKYKP
NvitrDIA1      260 TDISMDNIAVNNEDK-----AIFVDLENIIIVEKNPPEKALVGIESWNE-----TYTNAV
SpurpDIA1L     363 LDFGYENFVMTS-EG----QLKVVNLGGMVIVDKDQTSTTPDMNPHLNNRTELCNEDCLN
BflorDIA1La    331 VEVNIENFSVSS-DG----RLILTDLGNMTIINKHDLDKNSTKKRSSVC-----NEACFK
CinteDIA1      303 HRLTPRDIGISD-TG----KLRIKNADNVILID----NNSKN------------DKGLYE
AgambDIA1      264 TDINPDNVVVNLKNDNKQVYVSIVDLDNVILLDSWAEVFLTKN-----------THHVHS
consensus      421  d     fav         kv i d e i v d
```

… Motif 10 Motif 11

```
SpurpDIA1     328 CGSRRECIMFSHTDLCNHYYSDHNYYAVCQGLFGKDSPHGD-----------GGLLHSIP
NvectDIA1     318 CS-QPDCLSFSPSDMCSGGSRDHNYYAICQGVLAGSSRRQ-------------GLLHNPP
GaculDIA1     336 CD-REACLSFSKESLCSRVTVDHNYYAVCQNLLSRYATWRGTT--------GGLLHDPP
OlatiDIA1     336 CD-REACLSFSKDSLCSRVTVDHNYYAVCQNLLSRYATWRGTT--------GGLLHDPP
TnigrDIA1     336 CD-REACLSFSKDSLCSRVTVDHNYYAVCQNLLSRYATWRGTT--------GGLLHDPP
TrubrDIA1     336 CD-REACLSFSKDSLCSRVTVDHNYYAVCQNLLSRYATWRGTT--------GGLLHDPP
DreriDIA1b    336 CD-KEACLSFSKDMLCSRVTVDHNYYAICQNLLSRYATWRGSS--------GGLLHDPP
DreriDIA1a    336 CD-KEACLSFSKDILCSRVTVDHNYYAVCQDLLSRFSSWRGST--------GGLLHDPP
BtaurDIA1     337 CD-KEACLSFSKEILCARATVDHNYYAVCQNLLSRHATWRGTS--------GGLLHDPP
PpygmDIA1     337 CD-KEACLSFSKEILCARATVDHNYYAVCQNLLSRHATWRGTS--------GGLLHDPP
CfamiDIA1     337 CD-KEACLSFSKEILCARATVDHNYYAVCQNLLSRHATWRGTS--------GGLLHDPP
PtrogDIA1     337 CD-KEACLSFSKEILCARATVDHNYYAVCQNLLSRHATWRGTS--------GGLLHDPP
TtrunDIA1     337 CD-KEACLSFSKEILCARATVDHNYYAVCQNLLSRHATWRGTS--------GGLLHDPP
HsapiDIA1     337 CD-KEACLSFSKEILCARATVDHNYYAVCQNLLSRHATWRGTS--------GGLLHDPP
MmulaDIA1     337 CD-KEACLSFSKEILCARATVDHNYYAVCQNLLSRHATWRGTS--------GGLLHDPP
PvampDIA1     337 CD-KEACLSFSKEILCARVTVDHNYYAVCQNLLFRHATWRGTS--------GGLLHDPP
MdomeDIA1     337 CD-KEACLSFSKEILCSRATVDHNYYAICQNLLSRHATWRGTS--------GGLLHDPP
MmuscDIA1     337 CD-KEACLSFSKEILCARVTVDHNYYAVCQNLLSRHATWRGTS--------GGLLHDPP
RnorvDIA1     337 CD-KEACLSFSKEILCARVTVDHNYYAVCQNLLSRHATWRGTS--------GGLLHDPP
GgallDIA1     336 CD-KEACLSFSKEILCARVTVDHNYYAVCQNLLSRHATWRGTS--------GGLLHDPP
XtropDIA1     336 CD-KEACLSFSKEILCSRATVDHNYYAICQNLLSRHATWRGTS--------GGLLHDPP
BflorDIA1     325 CPGMSNCLSFDVSALCRYLHTDHNYYAVCRNMLSEYASEMGKP--------GGLLHDPP
SsalaDIA1R    341 CLGQGTSPCHR-SPPCSSVRPTQSLTLLCTALLPRLLLTERGAQTTRLPMEGEAEAGRLA
DreriDIA1R    321 CL--SGSCVR--PPPCETVREAQSFILLCKYILN-NLLTSN-----------DKQSG-LP
RnorvDIA1R    339 CL-VSGCQVQ--MSSCDTVPEKQNLVLVCQQLLP-QLLRG-------------KFPSPVQ
MmuscDIA1R    339 CL-VSDCQIQ--LSSCDTVPEKQSLVLVCQQLLP-QLLQG-------------KFPSPVQ
MmulaDIA1R    337 CL-VSGCQAQ--LPSCESVSEKQSLVLVCQKLLP-RLLQG-------------RFPSPVQ
HsapiDIA1R    337 CL-VSGCQAQ--LPSCESISEKQSLVLVCQKLLP-RLLQG-------------RFPSPVQ
BtaurDIA1R    337 CL-VSGCQTK--LPSCDTIPEKQNLVLVCSQVLP-LLLQA-------------KFPSPVQ
EcabaDIA1R    337 CL-LSDCQAE--LPSCDTVPEKQSLVLVCRQVLP-RLLQG-------------KFPSPVQ
DordiDIA1R    337 CL-VSGCQAQL-LPSCDNIPDKQSVVLVCQQLLP-QLLQG-------------KFPSPVQ
MdomeDIA1R    336 CL-VLDCQSP--FPSCSSVKEKQSWVMACQQLLP-QLLKE-------------KFPQPIQ
OanatDIA1R    336 CL-AADCQSD--LPSCNTVQEHQSLVLVCRELLP-KLLEG-------------KFPKPVQ
GgallDIA1R    334 CL-TVDCQSA--FVSCNSIREKQSLVMVCQELLP-KLLRG-------------KFLPPVQ
BflorDIA1Lb   431 FISQIEAMNSYDSCSAMPQYSQMMYALACVRLLSHLPEHLSEP--NPMDPKPDKHRPDSE
BflorDIA1Lc   292 FQKKLYS-DEYH-CDDIFKYAPMMYASICARLLSNLQKHPERRKWGEIREYQEGQSIELD
AaegyDIA1     302 RIDCDGCFAYIQEDICVHQISDINLFAVCQLLLENLNGDSKR-----------GFLHSLG
CpipiDIA1     305 RIPCDGCFAYVQEDLCSYQHSDINQFAICQLLYENLNGDREG-----------GFLHIQP
DyakuDIA1     270 -LPGEG-FTFDVSAFCSGHQLDANIYQACLLLRDYLLKNLNN------------------
DerecDIA1     271 -LPGEG-FTFDVSAFCSGHQLDANIYQACLLLRDFLLKNLDN------------------
DmelaDIA1     273 -LTGEG-FTFDVSAFCSGHQLDANIYQACLLLRDYLLKNLDN------------------
DsechDIA1     275 -LAGEG-FTFDVSAFCSGHQLDANIYQACLLLRDSLLKNLDN------------------
DpersDIA1     274 -LPGDG-FTFDVKSFCGGQQLDANVYQACLLLKDFLLRDLAN------------------
DpseuDIA1     274 -LPGDG-FTFDVKSFCGGQQLDANVYQACLLLKDFLLRDLAN------------------
DananDIA1     274 -LPGEG-FTFDVSAFCSGRQLDANVYQACLLLRDFLLKDLDN------------------
DwillDIA1     264 -FPGEG-FTYDVNAFCDNQDLDANIYQVCLLLRDQLLPDVKN------------------
NvitrDIA1     310 DLDCQDCFVFSPNDICSHKVSDHNFYAICQHILTQALGAVFHN---------QGFLHDPP
SpurpDIA1L    418 TFVKQLQTEPDTHCREVPRHVELMYMMACHSLLSDLMTTKYER--------FFQPLDTPR
BflorDIA1La   381 RFTRNLSHRPETSCRQAGRYSQLMYARACQRILGCWQTERSGE-----GRVVLSSSKDAS
CinteDIA1     342 VRYKQEKVDITEENLCDHSENDVNYYVICRHFLGGTWKERKGK------KYPGGLLHDPK
AgambDIA1     313 KIECNGCFAYVQEDVCRYQNSDLNLFATCQLLLENLNGHYAK-----------GLLHYDR
consensus     481 c    ac        c     d n y  C  ll                    g lh
```

Motif 12 Motif 13 Motif 14 …

```
SpurpDIA1     377 SDL--------SSYMRAKVKGQIAECRSPSK--RDGRYLVVKELKETLM-----------
NvectDIA1     365 ------------VALKASLNQLLQDCTAQDM--RNTREETAQKLSELLQ-----------
GaculDIA1     386 AH----------IAKDGQLLTLLDECTRPKK--RYGRFQAAKELREYLT-----------
OlatiDIA1     386 AH----------IAKDGQLEALLDECTRPKK--RYGRFQAAKELREYLT-----------
TnigrDIA1     386 AH----------IAKDGQLEALLDECTKPKK--RYGRFQAAKELREYLT-----------
TrubrDIA1     386 AH----------IAKDGQLEALLDECTKPKK--RFGRFQAAKELREYLT-----------
DreriDIA1b    386 PH----------IAKDGQLEALLEECANPKK--RYGRFQAAKELRDYLT-----------
DreriDIA1a    386 PD----------VVKDGRLIALLDECTRPQK--RYGRFQAAKELREFLT-----------
BtaurDIA1     387 SE----------IAKDGRLEALLDECANPKK--RYGRFQAAKELREYLA-----------
PpygmDIA1     387 SE----------IAKDGRLEALLDECANPKK--RYGRFQAAKELREYLA-----------
CfamiDIA1     387 SE----------IAKDGRLEALLDECANPKK--RYGRFQAAKELREYLA-----------
PtrogDIA1     387 SE----------IAKDGRLEALLDECANPKK--RYGRFQAAKELREYLA-----------
TtrunDIA1     387 SE----------IAKDGRLEALLDECANPKK--RYGRFQAAKELREYLA-----------
HsapiDIA1     387 SE----------IAKDGRLEALLDECANPKK--RYGRFQAAKELREYLA-----------
MmulaDIA1     387 SE----------IAKDGRLEALLDECANPKK--RYGRFQAAKELREYLA-----------
PvampDIA1     387 SE----------IAKDGRLEALLDECANPKK--RYGRFHAAKELREYLA-----------
MdomeDIA1     387 GE----------IAKDGRLEALLDECAHPKK--RFGRFQAAKELREYLA-----------
MmuscDIA1     387 SE----------IAKDGRLEALLDECTNPKK--RYGRFQAAKELRGYLA-----------
RnorvDIA1     387 SE----------IAKDGRLEALLDECTNPKK--RYGRFQAAKELRGYLA-----------
GgallDIA1     386 AD----------IAKDGRLEALLDECANPKK--RYGRFQAAKELREYLA-----------
XtropDIA1     386 AE----------IAKDGRLEALLDECANPKK--RYGRFKSAKELREYLA-----------
BflorDIA1     376 ET----------VVRDGTLQRLLAECAKPRT--LYGRFDAAKELIELLG-----------
SsalaDIA1R    400 RDV--------PLLLGVCADPSQPDWRIMAA--VGSLMDLLKPMRPCNP-----------
DreriDIA1R    364 RAA--------VDELLVCADPSQLDQTIIKS--VQSLKNILKTLRPCSP-----------
RnorvDIA1R    382 QEI--------DSALSLCSKDASTNLEVFGA--TSLLKNILRSLRTCDP-----------
MmuscDIA1R    382 KEI--------DSALSLCSKDNSTDLEVLGA--TSWLKDILRSLRTCDP-----------
MmulaDIA1R    380 DDI--------DSALAQCGDSTRPDPEVLGA--ASWLKHILRPLRTCDS-----------
HsapiDIA1R    380 DDI--------DSILVQCGDSIRPDPEVLGA--ASQLKDILRPLRTCDS-----------
BtaurDIA1R    380 EEI--------DAELTRCADGTRPDPEVLGA--ASRLKDILRPLRTCDP-----------
EcabaDIA1R    380 EEI--------DATLTWCGEDTRPDAEVLGA--ASRLKDILRPLRTCDP-----------
DordiDIA1R    381 VEI--------DSALAQCGQGIRPDSEVFGA--ASRLKDILRPLRTCDP-----------
MdomeDIA1R    379 EQI--------DSALVLCGNSSLSDQEVIEA--ASRLKAILKPLRTCDP-----------
OanatDIA1R    379 EEI--------DALLDRCGDSSLDGQTVIGG--ARRLMDILKSLRTCDA-----------
GgallDIA1R    377 EKI--------DSFLQHCAEGLADDQDVNEA--MAKLAQLLKPLRSCDS-----------
BflorDIA1Lb   489 RP---RLLWGPPEQEAKVLEELLRGCVEENV--AGGRLEAVGELKTFLR-----------
BflorDIA1Lc   350 EPPVKGFLHNPPDEIREALEGALTQCVHETL--PRGRLGAVLRLQEILG-----------
AaegyDIA1     351 TDS-----------KLEAFRKLLHQCVYCQPPYCEDRQEVLQRIMEIIH-----------
CpipiDIA1     354 NDD--------SQPRLSEIRQLLHHCVYCVPPDCRDRQGLLQQVQEIID-----------
DyakuDIA1     310 ----------------ERLQLLLEQCVACQDDFCDMRFQHAYDLIKMLD-----------
DerecDIA1     311 ----------------ERLQLLLEQCVACQDDFCDMRFQHAYDLIKVLD-----------
DmelaDIA1     313 ----------------EKLQLLLEQCVACQDDFCDMRFQHAYDLIKVLE-----------
DsechDIA1     315 ----------------EKLQLLLEQCVACQDDFCDMRFQHAYDLIKELE-----------
DpersDIA1     314 ----------------GRLEMLLEQCVRCDDATCDMRFQQAYDLIKLLG-----------
DpseuDIA1     314 ----------------GRLEMLLEQCVRCDDATCDMRFQQAYDLIKLLG-----------
DananDIA1     314 ----------------ERLQLLLEKCVECQDDLCDMRFQYAYDLIKLLD-----------
DwillDIA1     304 ----------------HRLKEMLNDCVQCEDDKCDIRFKYANNLIDLLV-----------
NvitrDIA1     361 D---------YILQKHPTLIDLLEQCAKPDIG--YSRIDIAHKLIVLLD-----------
SpurpDIA1L    470 KHHPGGMLHDAPYEVDSVLSELLYECVFEGQ--PGRRMHSVRVLKRLLT-----------
BflorDIA1La   436 CAYGRGLLFGPPREAKQELEDLLTECVEETK--AGGRITALKQIRVLLA-----------
CinteDIA1     396 EFG----------VFSSQMFVLLDICSRGKTAVAPERRTLSHSIRRLAANQLRNVLERRR
AgambDIA1     362 DAEAFP----VLGEASKMLQNLLSECVYCQPPDCQNRSHILKDMLHIID-----------
consensus     541  e                    l e                  l
```

… Motif 14 Motif 15

```
SpurpDIA1    416 ----KLIT------------------------
NvectDIA1    400 ----GYL-------------------------
GaculDIA1    423 ----QLAAASSSATAR----------------
OlatiDIA1    423 ----KLAAASSSATSR----------------
TnigrDIA1    423 ----QLA-------------------------
TrubrDIA1    423 ----QLAASSSSLVSAR---------------
DreriDIA1b   423 ----QLSGSAR---------------------
DreriDIA1a   423 ----QLTQTSNADR------------------
BtaurDIA1    424 ----QLSNNVR---------------------
PpygmDIA1    424 ----QLSNNVR---------------------
CfamiDIA1    424 ----QLSNNVR---------------------
PtrogDIA1    424 ----QLSNNVR---------------------
TtrunDIA1    424 ----QLSNNVR---------------------
HsapiDIA1    424 ----QLSNNVR---------------------
MmulaDIA1    424 ----QLSNNVR---------------------
PvampDIA1    424 ----QLSNNVR---------------------
MdomeDIA1    424 ----QLSNNGR---------------------
MmuscDIA1    424 ----QLSHNVR---------------------
RnorvDIA1    424 ----QLSHNVR---------------------
GgallDIA1    423 ----QLSNNVR---------------------
XtropDIA1    423 ----QLSNNAR---------------------
BflorDIA1    413 ----SFLKDR----------------------
SsalaDIA1R   439 ----HYTYRYPECRYNQDY-------------
DreriDIA1R   403 ----QYAYRYPECLYSDKF-------------
RnorvDIA1R   421 ----RFAYRYPDCKYNDRF-------------
MmuscDIA1R   421 ----RFAYRYPDCKYNDRF-------------
MmulaDIA1R   419 ----RFAYRYPDCKYNDKF-------------
HsapiDIA1R   419 ----RFAYRYPDCKYNDKF-------------
BtaurDIA1R   419 ----RFAYRYPDCKYDDKF-------------
EcabaDIA1R   419 ----RFAYRYPDCKYNDKF-------------
DordiDIA1R   420 ----RFAYRYPDCKYNDRF-------------
MdomeDIA1R   418 ----RYAYRYPDCKYSDRY-------------
OanatDIA1R   418 ----RFAYRYPDCKYSEKY-------------
GgallDIA1R   416 ----RFAYRYPDCKYSDKY-------------
BflorDIA1Lb  533 ----RNAKGEN---------------------
BflorDIA1Lc  397 ----TT--------------------------
AaegyDIA1    389 ----DVLHEVV---------------------
CpipiDIA1    395 ----GILVES----------------------
DyakuDIA1    343 ----SKN-------------------------
DerecDIA1    344 ----SKI-------------------------
DmelaDIA1    346 ----SNN-------------------------
DsechDIA1    348 ----SKN-------------------------
DpersDIA1    347 ----ELLGS-----------------------
DpseuDIA1    347 ----ELLGS-----------------------
DananDIA1    347 ----S---------------------------
DwillDIA1    337 ----EINNKIEIL-------------------
NvitrDIA1    399 ----SVIKNA----------------------
SpurpDIA1L   517 ----IIQRGFSYKDAADRTSL-----------
BflorDIA1La  483 ----A---------------------------
CinteDIA1    446 ICSPDFKYRYPECELAEDAGINLPSIELQWLH
AgambDIA1    407 ----QTIIQS----------------------
consensus    601
```
