# Supplementary material for: Characterization of the Deleted in Autism 1 Protein Family: Implications for Studying Cognitive Disorders
Source: PLoS One. 2011 Jan 19;6(1):e14547. doi: 10.1371/journal.pone.0014547 (PMC3023760; doi:10.1371/journal.pone.0014547)
Supplement: Figure S10 — DIA1-family amino acid sequences in FASTA format. Each DIA1-family amino acid sequence starts with a “>” (greater-than) symbol followed by the species abbreviation and protein type (e.g., Oryzias latipes DIA1 is abbreviated to OlatiDIA1 and Pongo pygmaeus DIA1R to PpygmDIA1R). Following the initial title line is the actual amino acid sequence, in standard single-letter code. Accession numbers, full species names, and differences to current database sequence data (due to corrections) can be found in Tables S1, S5 and S8. (0.04 MB PDF) [file pone.0014547.s020.pdf]

Fig. S10

>GaculDIA1
MLRFLPLKLGRLYRCLKLLLVVGLFVILLMNTHSLFASFQKNELTDRRFINLNKCPACFG
TSWCRKFMNGQVSFETWGRLRFLDVFNVKNVYFAQYGEPREGTRRVVLKRLGSNQELAEI
DQKICKRATGRPRCDLIQAMYKTEFARINGDVRLLTPEVVEGWSDLVHCPSQRLLDRVVR
RYAETKDSGSFLLKNLKDTERMQLLMTLAFNPEPLVLQ
SFPSDEGWPFAKYLGACGRMV
AVNYVGEELWSFYNAPWEKRVDLARQLMDIAEQLTNNDFDFALYLLDVSFDNFAVGPRDG
KVIVVDAENVLVADKRLIKQNKPESFDVWYESRFEECDREACLSFSKESLCSRVTVDHNY
YAVCQNLLSRYATWRGTTGGLLHDPPAHIAKDGQLLTLLDECTRPKKRYGRFQAAKELRE
YLTQLAAASSSATAR
>TtrunDIA1
MWRLVPPKLGRLSRSLKLAALGSLLVLMVLHSPSLLASWQRNELADRRFLQLNKCPACFG
TSWCRRFLNGQVVFEAWGRLRLLDFLNVKNVYFAQYGEPREGGRRRVVLKRLGSQRELAQ
LDQSICKRATGRPRCDLLQAMPRTEFARLNGDVRLLTPEAVEGWSDLVHCPSQRLLDRLV
RRYAETKDSGSFLLRNLKDSERMQLLLTLAFNPEPLVLQSFPSDEGWPFAKYLGACGRMV
AVNYVGEELWSYFNAPWEKRVDLAWQLMEIAEQLTNNDFEFALYLLDVSFDNFAVGPRDG
KVIIVDAENVLVADKRLIRQNKPENWDVWYESKFDDCDKEACLSFSKEILCARATVDHNY
YAVCQNLLSRHATWRGTSGGLLHDPPSEIAKDGRLEALLDECANPKKRYGRFQAAKELRE
YLAQLSNNVR
>PvampDIA1
MWRLVPPKLGRLSRSLKLAALGSLLVLMVLHSPSLLASWQRNELADRRFLQLNKCPACFG
TSWCRRFLNGQVVFEAWGRLRLLDFLNVKNVYFAQYGEPREGGRRRVVLKRLGSQRELAQ
LDQSICKRATGRPRCDLLQAMPRTEFARLNGDVRLLTPEAVEGWSDLVHCPSQRLLDRLV
RRYAETKDSGSFLLRNLKDSERMQLLLTLAFNPEPLVLQSFPSDEGWPFAKYLGACGRMV
AVNYVGEELWSFFNAPWEKRVDLAWQLMEIAEQLTNNDFEFALYLLDVSFDNFAVGPRDG
KVIIVDAENVLVADKRLIRQNKPENWDVWYESKFDDCDKEACLSFSKEILCARVTVDHNY
YAVCQNLLFRHATWRGTSGGLLHDPPSEIAKDGRLEALLDECANPKKRYGRFHAAKELRE
YLAQLSNNVR
>OlatiDIA1
MLRFLPLKLGRLYRCLKLLFLVGLFVILLMNTHNLFASFQKNELTDRRFINLNKCPACFG
TSWCRKFMNGQVSFETWGRLRFLDVFNVKNVYFAQYGEPREGTRRVVLKRLGSNQELAEI
DQKICKRATGRPRCDLVQAMYKTEFARINGDVRLLTPDVVEGWSDLVHCPSQRLLDRVVR
RYAETKDSGSFLLKNLKDTERMQLLMTLAFNPEPLVLQSFPSDEGWPFAKYLGACGRMVA
VNYVGEELWNFYNAPWEKRVDLARQLMDIAEQLTNNDFDFALYLLDVSFDNFAVGPRDGK
VIVVDAENVLVADKRLIKQNKPESYDVWYESRFEECDREACLSFSKDSLCSRVTVDHNYY
AVCQNLLSRYATWRGTTGGLLHDPPAHIAKDGQLEALLDECTRPKKRYGRFQAAKELREY
LTKLAAASSSATSR
>HsapiDIA1
MWRLVPPKLGRLSRSLKLAALGSLLVLMVLHSPSLLASWQRNELTDRRFLQLNKCPACFG
TSWCRRFLNGQVVFEAWGRLRLLDFLNVKNVYFAQYGEPREGGRRRVVLKRLGSQRELAQ
LDQSICKRATGRPRCDLLQAMPRTEFARLNGDVRLLTPEAVEGWSDLVHCPSQRLLDRLV
RRYAETKDSGSFLLRNLKDSERMQLLLTLAFNPEPLVLQSFPSDEGWPFAKYLGACGRMV
AVNYVGEELWSYFNAPWEKRVDLAWQLMEIAEQLTNNDFEFALYLLDVSFDNFAVGPRDG
KVIIVDAENVLVADKRLIRQNKPENWDVWYESKFDDCDKEACLSFSKEILCARATVDHNY
YAVCQNLLSRHATWRGTSGGLLHDPPSEIAKDGRLEALLDECANPKKRYGRFQAAKELRE
YLAQLSNNVR
>MmuscDIA1
MWRLVPLKLGRLSRALKLAALGSLLVLMLLHSPSLLASWQRNELADRRFLQLNKCPACFG
TSWCRRFLNGQVGFETWGRLRLLDFLNVKNVYFAQYGEPREGGRRRVVLKRLGSQRELAQ
LDQSICKRATGRPRCDLLQAMPRTEFARLNGDVRLLTPEAVEGWSDLVHCPSQRLLDRLV
RRYAETKDSGSFLLRNLKDSERMQLLLTLAFNPEPLVLQSFPSDEGWPFAKYLGACGRMV
AVNYVGEELWSYFNAPWEKRVDLAWQLMEIAEQLTNNDFEFALYLLDVSFDNFAVGPRDG
KVIIVDAENVLVADKRLIRQNKPENWDVWYESKFDDCDKEACLSFSKEILCARVTVDHNY
YAVCQNLLSRHATWRGTSGGLLHDPPSEIAKDGRLEALLDECTNPKKRYGRFQAAKELRG
YLAQLSHNVR
>MmulaDIA1
MWRLVPPKLGRLSRSLKLAALGSLLVLMVLHSPSLLASWQRNELTDRRFLQLNKCPACFG
TSWCRRFLNGQVVFEAWGRLRLLDFLNVKNVYFAQYGEPREGGRRRVVLKRLGSQRELAQ
LDQSICKRATGRPRCDLLQAMPRTEFARLNGDVRLLTPEAVEGWSDLVHCPSQRLLDRLV
RRYAETKDSGSFLLRNLKDSERMQLLLTLAFNPEPLVLQSFPSDEGWPFAKYLGACGRMV
AVNYVGEELWSYFNAPWEKRVDLAWQLMEIAEQLTNNDFEFALYLLDVSFDNFAVGPRDG
KVIIVDAENVLVADKRLIRQNKPENWDVWYESKFDDCDKEACLSFSKEILCARATVDHNY
YAVCQNLLSRHATWRGTSGGLLHDPPSEIAKDGRLEALLDECANPKKRYGRFQAAKELRE
YLAQLSNNVR
>PtrogDIA1
MWRLVPPKLGRLSRSLKLAALGSLLVLMVLHSPSLLASWQRNELADRRFLQLNKCPACFG
TSWCRRFLNGQVVFEAWGRLRLLDFLNVKNVYFAQYGEPREGGRRRVVLKRLGSQRELAQ
LDQSICKRATGRPRCDLLQAMPRTEFARLNGDVRLLTPEAVEGWSDLVHCPSQRLLDRLV
RRYAETKDSGSFLLRNLKDSERMQLLLTLAFNPEPLVLQSFPSDEGWPFAKYLGACGRMV
AVNYVGEELWSYFNAPWEKRVDLAWQLMEIAEQLTNNDFEFALYLLDVSFDNFAVGPRDG
KVIIVDAENVLVADKRLIRQNKPENWDVWYESKFDDCDKEACLSFSKEILCARATVDHNY
YAVCQNLLSRHATWRGTSGGLLHDPPSEIAKDGRLEALLDECANPKKRYGRFQAAKELRE
YLAQLSNNVR
>CfamiDIA1

MWRLVPPKLGRLSRSLKLAALGSLLVLMVLHSPSLLASWQRNELADRRFLQLNKCPACFG
TSWCRRFLNGQVVFEAWGRLRLLDFLNVKNVYFAQYGEPREGGRRRVVLKRLGSQRELAQ
LDQSICKRATGRPRCDLLQAMPRTEFARLNGDVRLLTPEAVEGWSDLVHCPSQRLLDRLV
RRYAETKDSGSFLLRNLKDSERMQLLLTLAFNPEPLVLQSFPSDEGWPFAKYLGACGRMV
AVNYVGEELWSYFNAPWEKRVDLAWQLMEIAEQLTNNDFEFALYLLDVSFDNFAVGPRDG
KVIIVDAENVLVADKRLIRQNKPENWDVWYESKFDDCDKEACLSFSKEILCARATVDHNY
YAVCQNLLSRHATWRGTSGGLLHDPPSEIAKDGRLEALLDECANPKKRYGRFQAAKELRE
YLAQLSNNVR
>BtaurDIA1
MWRLVPPKLGRLSRSLKLAALGSLLVLMVLHSPSLLASWQRNELADRRFLQLNKCPACFG
TSWCRRFLNGQVVFEAWGRLRLLDFLNVKNVYFAQYGEPREGGRRRVVLKRLGSQRELAQ
LDQSICKRATGRPRCDLLQAMPRTEFARLNGDVRLLTPEAVEGWSDLVHCPSQRLLDRLV
RRYAETKDSGSFLLRNLKDSERMQLLLTLAFNPEPLVLQSFPSDEGWPFAKYLGACGRMV
AVNYVGEELWSYFNAPWEKRVDLAWQLMEIAEQLTNNDFEFALYLLDVSFDNFAVGPRDG
KVIIVDAENVLVADKRLIRQNKPENWDVWYESKFDDCDKEACLSFSKEILCARATVDHNY
YAVCQNLLSRHATWRGTSGGLLHDPPSEIAKDGRLEALLDECANPKKRYGRFQAAKELRE
YLAQLSNNVR
>MdomeDIA1
MWRLVPPKLGRLSRSLKLAALGSLLVLMVLHSPSLLASWQRNELADRRFLQLNKCPACFG
TSWCRRFLNGQVAFEAWGRLRLLDFLNVKNVYFGQYGEPREGGRRRVVLKRLGSQRELAQ
LDQSICKRATGRPRCDLLQAMPKTEFARLNGDVRLLTPDAVEGWSDLVHCPSQRLLDRLV
RRYAETKDSGSFLLRNLKDSERMQLLLTLAFNPEPLVLQSFPSDEGWPFAKYLGACGRMV
AVNYVGEELWSYFNAPWEKRVDLAWQLMEIAEQLTNNDFEFALYLLDVSFDNFAVGPRDG
KVIIVDAENVLVADKKLIRQNKPENWDVWYESKFDDCDKEACLSFSKEILCSRATVDHNY
YAICQNLLSRHATWRGTSGGLLHDPPGEIAKDGRLEALLDECAHPKKRFGRFQAAKELRE
YLAQLSNNGR
>RnorvDIA1
MWRLVPLKLGRLSRALKLAALGSLLVLMLLHSPSLLASWQRNELADRRFLQLNKCPACFG
TSWCRRFLNGQVGFETWGRLRLLDFLNVKNVYFAQYGEPREGGRRRVVLKRLGSQRELAQ
LDQSICKRATGRPRCDLLQAMPRTEFARLNGDVRLLTPEAVEGWSDLVHCPSQRLLDRLV
RRYAETKDSGSFLLRNLKDSERMQLLLTLAFNPEPLVLQSFPSDEGWPFAKYLGACGRMV
AVNYVGEELWSYFNAPWEKRVDLAWQLMEIAEQLTNNDFEFALYLLDVSFDNFAVGPRDG
KVIIVDAENVLVADKRLIRQNKPENWDVWYESKFDDCDKEACLSFSKEILCARVTVDHNY
YAVCQNLLSRHATWRGTSGGLLHDPPSEIAKDGRLEALLDECTNPKKRYGRFQAAKELRG
YLAQLSHNVR
>XtropDIA1
MLRLASLKFGRLFRYAKVLFAASLLVVMLLNTHSLLSSFQRNELTDRRFLSLNKCPACFG
TSWCRKFMNGQLSFEGWGRLRLLDFFNVKNVHFAQYGEPREGSRRVVLKRLGSNHELSEL
DQRICKKATGRPRCDLVQAMYKTDFARLNGDVRLLTPDVVEGWSDLVHCPSQRLLDRLVR
RYAETKDSGSFLLRNLKDTERMQLLLTLAFNPEPLVLQSFPSDEGWPFAKYLGACGRMVA
VNYVGEELWSYFNAPWEKRVDLAWQLMEIAEQLTNNDFDFALYLLDVSFDNFAVGPRDGK
VIIVDAENVLVADKKLIKQNKPENWDVWYESKFDDCDKEACLSFSKEILCSRATVDHNYY
AICQNLLSRHATWRGTSGGLLHDPPAEIAKDGRLEALLDECANPKKRYGRFKSAKELREY
LAQLSNNAR
>GgallDIA1
MLRLVSLKLGRLYRYVKLAVLGSLAAALVLNTHSLLASLQRNELAERRFLQLNKCPACWG
TSWCRKFLNGQLRLESWGRLRLFDFFNVKNVYFARYGEPREGSRRVVLKRLGSAQELADI
DAKICRRATGRGRCDLLQALHATEFASLNGDVRLLTPDAVEGWSDLVHCPSQRLLDRLVR
RYAETKDSGSFLLRNLKDSERMQLLITLAFNPEPLVLQSFPSDEGWPFAKYLGACGRMVA
VNYVGEELWSYFNAPWEKRVDLAWQLMEIAEQLTNNDFEFALYLLDVSFDNFAVGPRDGK
VIIVDAENVLVADKRLIRQNKPENWDVWYESKFDDCDKEACLSFSKEILCARVTVDHNYY
AVCQNLLSRHATWRGTSGGLLHDPPADIAKDGRLEALLDECANPKKRYGRFQAAKELREY
LAQLSNNVR
>TnigrDIA1
MLRFLPLKLGRLYRCLKLLLVVGLFVILLMNTHNLFASFQKNELTDRRFINLNKCPACFG
TSWCRKFMNGQVSFETWGRLRFLDVFNVKNVYFAQYGEPREGTRRVVLKRLGSNQELADI
DQKICKRATGRPRCDLIQAMYKTEFARINGDVRLLTPDVVEGWSDLVHCPSQRLLDRVVR
RYAETKDSGSFLLKNLKDTERMQLLMTLAFNPEPLVLQSFPSDEGWPFAKYLGACGRVVA
VNYVGEELWSFYNAPWEKRVDLARQLMDIAEQLTNNDFEFALYLLDVSFDNFAVGPRDGK
VIIVDAENVLVADKRLIKQNKPENFDVWYESRFEECDREACLSFSKDSLCSRVTVDHNYY
AVCQNLLSRYATWRGTTGGLLHDPPAHIAKDGQLEALLDECTKPKKRYGRFQAAKELREY
LTQLA
>DreriDIA1b
MLRFLPLKLGRLYRCLKLLFLLGLFVILLMNTHNLFASFQKNELTDRRFINLNKCPACFG
TSWCRKFMNGQISFETWGRLRFLDVFNVKNVFFAQYGEPREGTRRIVLKRLGSNQELADI
DQKICKRATGRPRCDLIQAMYKTEFARLNGDVRLLTPDVVEGWSDLVHCPSQRLLDRIVR
RYAETKDSGSFLLKNLKDTERMQLLMTLAFNPEPLVLQSFPSDEGWPFAKYLGACGRMVA
VNYVGEELWSFFNAPWEKRVDLAKQLMDIAEQLTNNDFDFALYLLDVSFDNFAVGPRDGK
VIVVDAENVVVADKRLIKQNKPESYDVWYESRFEECDKEACLSFSKDMLCSRVTVDHNYY
AICQNLLSRYATWRGSSGGLLHDPPPHIAKDGQLEALLEECANPKKRYGRFQAAKELRDY
LTQLSGSAR
>DreriDIA1a
MLRILSLKFGRVYRCGKFLFIVALFVILLMNTHNLFASFQRNELTDRRFIGLNKCPACFG
TSWCRKFMNGQVTFEMWGRLRFLDFFNVKNVFFAQYGEPREGTRRVVLKRLGSNQELAEI
DQKICKRATGRPRCDLIQSMYKTEFARLNGDVRLLTPEVVEGWSDLVHCPSQRLLDRVVR
RYAETKDSGSFLLKNLKDTERMQLLMTLAFNPEPLVLQSFPSDEGWPFAKYLGACGRMVA
VNYVGEELWSFYNAPWEKRVDLAKQLMDIAEQLTNNDFDFALYLLDVSFDNFAVGPRDGK

VIVIDAENVIVVDKRLVKQNKPESYDVWYESRYEECDKEACLSFSKDILCSRVTVDHNYY
AVCQDLLSRFSSWRGSTGGLLHDPPPDVVKDGRLIALLDECTRPQKRYGRFQAAKELREF
LTQLTQTSNADR
>SpurpDIA1
MRRYFNQFTNRFCISTTQQLIWLVVLSSMCLFVYYQLYLHFTANHLEN
EYFTEATKCPACFGTSLCKRFSRGDYRFHSYSSIRLFDYVNVKNVYFATYLDQKPVVMKK
LGHNSEHNQFDKDLCTNAESGLRGCDVSQQIYKSKLSDIMYAERLEEKDVMGLSDIVRCP
SRRLLDRIWDTFGERQFDKSLARDHKLMLATTIAFNPEPLILQAFPNRKGWPFPAYMGAC
GRFTVQEYSGHSLSYFYKFKFGVRAALAVQALKIAEQLSENRDEFALYLTDVSEDNLAVN
DNGEVLVIDAENIIVVDRKKIKEDANPGWDVKHQSEHEGCGSRRECIMFSHTDLCNHYYS
DHNYYAVCQGLFGKDSPHGDGGLLHSIPSDLSSYMRAKVKGQIAECRSPSKRDGRYLVVK
ELKETLMKLIT
>NvitrDIA1
MLVTKASVSSFLIALLILILGIYINR
FNLKVAEITERYKCPACFGDSMCQVIDSNEISFEYTDFYSIFNNLFSVKNVYYGKYK
DKKVIMKKLAQESELKTFDEMICSDDELHELCYQSESRKQDSKTNFYKLVESELANVGGD
INNKMRLCPTTTKLEKLFVNVKFQSHAENEDYFKYLWSTIKINPEPLILQILPAKEGWPT
PKYFGACGRLIVEEYIGLPLSSFIDEPWIRRAKIASSLLQAADTLMSKNSEFAFYLTDIS
MDNIAVNNEDKAIFVDLENIIIVEKNPPEKALVGIESWNETYTNAVDLDCQDCFVFSPND
ICSHKVSDHNFYAICQHILTQALGAVFHNQGFLHDPPDYILQKHPTLIDLLEQCAKPDIG
YSRIDIAHKLIVLLDSVIKNA
>AaegyDIA1
MSQHRILTKLHLVALFLWIVMKYQPEERASSLKSLVQQCQYDTTILCPGCFPRPVNCSFF
ENLKSIDSNELFEELLTHFNQHGVRYGTLSGSEQRVVIKNLNKEHGVEELISSICNDLKI
YRNCRLRNKEPYLRVLRQRVLNDNQLEGAILCPLMDEEALKRFLFEIDENDLNKILLMRI
NAQPLLLKLLQERNFPVPKLIFQGGFTLVESYEGEALAKYYDRPLNVRLLIANELIKASL
NFTAGVDNFRFYLTDISPDNIVVSMSSEEEIKVTFVDLNNVIILDSHSKRLKPSKQKHVH
SRIDCDGCFAYIQEDICVHQISDINLFAVCQLLLENLNGDSKRGFLHSLGTDSKLEAFRK
LLHQCVYCQPPYCEDRQEVLQRIMEIIHDVLHEVV
>AgambDIA1
MQSSRLPYFVTLFIAVVLQYLPNEKSTSKRPLENVCDYDRNILCPECVSSGLECNKFDDL
FTLQDDNVFNIINNIFNSHTTRIGTIVQENKLAVMKHLNRDNTIEKLLQEYCDAYSLTKQ
NCRWAQHDGSVDRAKEFITTSILDSARVEGCIFCPATNNRQSLQRFLSLFDSTDNELWNL
LAVRTNVEPLLIKLLTVNYHDEPPLYVPKLLHTFGFSIIETYEGKTLEHYYDFPFVTRMR
IAAELIRAGFKFTEGIHGFRVFLTDINPDNVVVNLKNDNKQVYVSIVDLDNVILLDSWAE
VFLTKNTHHVHSKIECNGCFAYVQEDVCRYQNSDLNLFATCQLLLENLNGHYAKGLLHYD
RDAEAFPVLGEASKMLQNLLSECVYCQPPDCQNRSHILKDMLHIIDQTIIQS
>CpipiDIA1
MSSGFQRILVNLHILALLFCVIFEFQPKEPESLTELLVNQCEYETQHLCPECFVSPTVNC
GHLANFVTIDSSNPWNQLTCRYNPHGVSYGLLGSGQKVVIKTLNKNRAVEALRDAVCDEL
GLTQSNCKFKSDENTLKVLRRKVLEQELEGCIICPSKDEKALNRLLNEVEGTELLQLILL
KVNVQPLLLELFDGRGFPVPKVIFQGGFQLFESFDGDALVNFYDSSLNIRLRIAKELIQA
SFLFTEGVNGFRFYLTDINPDNIAVQAQPSGSFQVSFIDLDNVIILDSQSKRLDRRSKAR
NIHSRIPCDGCFAYVQEDLCSYQHSDINQFAICQLLYENLNGDREGGFLHIQPNDDSQPR
LSEIRQLLHHCVYCVPPDCRDRQGLLQQVQEIIDGILVES
>DyakuDIA1
MHLSPGQLKVVLILALLQELQVKPQREVFQELFEKDLRLCPNCF
FGQRELCGEIFQKIAEPSDWSKLLKAISLLVDRRVIYFLRFKDQDQVVAKRKIIDAQN
QQIKDVKKAFYELGERPGGFHLCRTPSRKPRFVSYLEQRGHASASVWFYMMHYVSPLL
MQELYLQGFPVPTTYASCGLTHFQSYAGRTLTNYFEGEEDLRVELALQLMQLSLKLTF
GFSDFRIMLTDFTGDNFAYDEHTKKVYLIDLDSVVLVDASSATGQAEKYEPLPGEGFT
FDVSAFCSGHQLDANIYQACLLLRDYLLKNLNNERLQLLLEQCVACQDDFCDMRFQHA
YDLIKMLDSKN
>DmelaDIA1
MHLSHGQLKVVLILALLQELQVKPQREVFQELFEKDLRLCPDCF
VGQSEPCGEIFQKIAEPSDWSRLVKAISLLVDRRAIYFLRLKDRDQEVQLVAKRKIID
AQSNQIKNVKKDFYELEERPGGFHLCRTLAREPRFVSYLEQRGHAAASVWFYMMYYVS
PLLMQELHLQGFPVPTTYASCGLTHFQSNAGRTLAHYVDAEEGLRVELALQLIQLSLK
LTFGFADFRIILTDFTADNLAYDEDTKKVYLIDLDSVVLVDASFAAGHAEKYEPLTGE
GFTFDVSAFCSGHQLDANIYQACLLLRDYLLKNLDNEKLQLLLEQCVACQDDFCDMRF
QHAYDLIKVLESNN
>DpersDIA1
MHLSPGHLRIVLILALLQDLQVKPQKDIFRAHLERDLQLCPACF
AGQRWQCEEIFEAIAEPSDWSKLLKGIALLIDRREIYFLQLNERHQAAQLVAKRDAIG
GHSYRNMGILREAFLEMEERPGGFQLCRSGFRTPRFVSYLEQRGHEAATIWYYMMHSI
TPLLMQELFLRGFPVPRSYAACGLTHFQAYAGRTLVHYATAGENLRLEMARQLLQLSL
KLTFGFADFRIFLTDFTADNLAFDEASQSVILIDLDSVVLVDAAVPLGDAQKYEPLPG
DGFTFDVKSFCGGQQLDANVYQACLLLKDFLLRDLANGRLEMLLEQCVRCDDATCDMR
FQQAYDLIKLLGELLGS
>DpseuDIA1
MHLSPGHLRIVLILALLQDLQVKPQKDIFRAHL
ERDLQLCPACFAGQRWQCEEIFEAIAEPSDWSKLLKGIALLIDRREIYFLQLNERHQA
AQLVAKRDAIGGHSYRNMGILGEAFLEMEERPGGFQLCRSGFRTPRFVSYLEQRGHEA
ATIWYYMMHSITPLLMQELFLRGFPVPRSYAACGLTHFQAYAGRTLVHYATAGENLRL
EMARQLLQLSLKLTFGFADFRIFLTDFTADNLAFDEASQSVILIDLDSVVLVDAAVPL
GDAQKYEPLPGDGFTFDVKSFCGGQQLDANVYQACLLLKDFLLRDLANGRLEMLLEQC
VRCDDATCDMRFQQAYDLIKLLGELLGS

>DerecDIA1
MHLSPGQLKVVLILALLQELQVKPQREVFQELYEKDLRLCPDCF
VGQSELCVGIFQKIAEPSDWSKLLKAISLIVDRRAIYYLRLKDQAQVVAKRKIIDARN
HKAENVEKAFYEQEEQPGGFHLCRTPSRKPPRFVSYLEQRGHASASVWFYMMHYVSPL
LMQELHLQGFPVPTTYASCGLTHFQAYAGRTLTNYFEAEEGLRMELALQLIQLSLKLT
FGFSDFRIFLTDFTGDNFAYDEDSKRVYLIDLDSVVLVDASSAAGQAEKYVPLPGEGF
TFDVSAFCSGHQLDANIYQACLLLRDFLLKNLDNERLQLLLEQCVACQDDFCDMRFQH
AYDLIKVLDSKI
>DsechDIA1
MHLSYGQLKVVLILALLQELQVKTQREVFQELFEKDLRLCPDCF
VGQKEQCGEIFQKIAEPSDWSRLVKAISLLVDRRAIHYLRLKDQDQAVQLVAKRKIIN
AHSYQMKNVKRAFYELEERPGGFHLCRTLAREPRFVSYLEQRGHVYGSTSVWFYMMHY
VSPLLMQELHLQGFPVPTPYASCGLTHFQSYAGRTLAHYVNAEEGLRLELALQLIQLS
LKLTFGFADFRIILTDFTADNLAYDEETKKVYLIDLDSVVLVDASSMAGQAEKYEPLA
GEGFTFDVSAFCSGHQLDANIYQACLLLRDSLLKNLDNEKLQLLLEQCVACQDDFCDM
RFQHAYDLIKELESKN
>DwillDIA1
MHLLYGQLKVVLVLALLQELDVRTQKDIFRKHFNMDLLECPQCF
LGQREQCEKFFDTIEEPAKLDRLWKDLTLLVDPREIYWLELNENRENVKCLAKRKMTR
KISKDLHFNPGGFQMCRNSTRFLAYLEERGYLPATIGYYIHHSISPLLMQELWIMDFP
VPRSFAVCGSTLFQAYAGSTLSNYLKAPIDLRLEMAKQLLQLALKLSAGFKGFRIYLT
DFTADNFAYNEANGTVLLIDMDTMVLVESNDEAESESQLSKKYKPFPGEGFTYDVNAF
CDNQDLDANIYQVCLLLRDQLLPDVKNHRLKEMLNDCVQCEDDKCDIRFKYANNLIDL
LVEINNKIEIL
>DananDIA1
MHLLSGQLRIVLVLALLQDLQVKPQKDIFQEHFESDLRLCPACF
SAQREQCEEVFRAIREPSDLSKLWKAVTLLFDRREIFWLEIGPSQEKSSQLLAKRKSI
VASHQSLSSLQEEFLNLEERPGGFHLCRTAQKSSPFINYLNQRGYDSSSIYFYMLHNI
SPLLMQELQNLDFPVPNTYAICGLTHFQAYAGRTLNHYTEAAEDLRREIARQLIHLSL
KLTFGFADFRIFLTDFTSDNLTFDEDTRRVLLIDLDSVVMVDAASTSGQAEKYEPLPG
EGFTFDVSAFCSGRQLDANVYQACLLLRDFLLKDLDNERLQLLLEKCVECQDDLCDMR
FQYAYDLIKLLDS
>CinteDIA1
MLLKMKRYLTVWENGKYIVFIFILITLEVLLEMYNHRSQGSTRVMLEAKLLSDKWSATSP
TCGNFSCFGTSLCKNRNRVGLSEDVPTLRSGWLSIYGELVDLHRVKLNVTYKLPSTLPCN
GETCSVQIETSVLSGNNVNKWICDKFKKPTNCDTGKVFKSWRAMNKITSAKGRLDPNLLK
DISPPLQCPSTRIADRIVRRVANKQGGVLLRDLSYTEKKRLIFTCKMSPGSVVQSMFPPY
EGWLFQRTVGSCGALMVHGRSIVTSLYELYDAPWKVRLDVAIQLLDLAEHFTYNDLNYMF
ALHRLTPRDIGISDTGKLRIKNADNVILIDNNSKNDKGLYEVRYKQEKVDITEENLCDHS
ENDVNYYVICRHFLGGTWKERKGKKYPGGLLHDPKEFGVFSSQMFVLLDICSRGKTAVAP
ERRTLSHSIRRLAANQLRNVLERRRICSPDFKYRYPECELAEDAGINLPSIELQWLH
>NvectDIA1
MHFGGILRKRRYICRLVLLLIVIISFSDLLLWLSYQDGRAIKWDFSSQHKLTFQEIGLNK
CPACFGINLSICHKLLGGGVTVKSNTPWSEERIKGVSYGLWGAVKQRVVLKTLGTSSELT
AFDNEICRGVGKDSNCKISQEAWDSDVLVPKPPLQDAVRKYFDCPSTRFLEKIESVYGQE
TPGQLSLVETIHLITGLHLNPELLLLQIFRQEEGWPFPRFYGVCGRIMVVEDSGPPISSF
LEESWDVRAQIAVNLITLAHQLSSALDDWALYIADPAPGNFGVSDSGKVTLLDLEHLVVV
DLSEVQMEEANDKPKPSDCSQPDCLSFSPSDMCSGGSRDHNYYAICQGVLAGSSRRQGLL
HNPPVALKASLNQLLQDCTAQDMRNTREETAQKLSELLQGYL
>BflorDIA1
MRLVRLRQFRRFFSGWRVRRMCGFFFFVVFFYCFVFQPFSYNQLTDTTFLGEDKCPACFGTDLCEEFENG
KILFKYSSRLRILDIFNIKNVYFAIYEGMEVALKRLGHNSEFDQLDRWICETGKEGPQCHIPSVTYYSNF
SRVIYEGLSPKSLGDMSDMVRCPSQRLIDRVLEKFAEHLGKETEELSYQEKLHFLSTLKFNPEPLMLQTF
PITEGWPFPFYFGACGRLTVVQKCDKTLASYYSAPWLKRVELSLQMMKIAEYLTNNEADFGLYLTDISYE
NFGVTSDGNLFVIDVENVIVVDKQKIKADKPRNWEARYQSHFDECPGMSNCLSFDVSALCRYLHTDHNYY
AVCRNMLSEYASEMGKPGGLLHDPPETVVRDGTLQRLLAECAKPRTLYGRFDAAKELIELLGSFLKDR
>TrubrDIA1
MLRFLPLKLGRLYRCLKLLLVVGLFVILLMNTHNLFASFQKNELTDRRFINLNKCPACFG
TSWCRKFMNGQVSFETWGRLRFLDVFNVKNVYFAQYGEPREGTRRVVLKRLGSNQELADI
DQKICKRATGRPRCDLIQAMYKTEFARINGDVRLLTPEVVEGWSDLVHCPSQRLLDRVVR
RYAETKDSGSFLLKNLKDTERMQLLMTLAFNPEPLVLQSFPSDEGWPFAKYLGACGRVVA
VNYVGEELWSFYNAPWEKRVDLARQLMDIAEQLTNNDFEFALYLLDVSFDNFAVGPRDGK
VIIVDAENVLVADKRLIKQNKPENYDVWYESRFEECDREACLSFSKDSLCSRVTVDHNYY
AVCQNLLSRYATWRGTTGGLLHDPPAHIAKDGQLEALLDECTKPKKRFGRFQAAKELREY
LTQLAASSSSLVSAR
>PpygmDIA1
MWRLVPPKLGRLSRSLKLAALGSLLVLMVLHSPSLLASWQRNELADRRFLQLNKCPACFG
TSWCRRFLNGQVVFEAWGRLRLLDFLNVKNVYFAQYGEPREGGRRRVVLKRLGSQRELAQ
LDQSICKRATGRPRCDLLQAMPRTEFARLNGDVRLLTPEAVEGWSDLVHCPSQRLLDRLV
RRYAETKDSGSFLLRNLKDSERMQLLLTLAFNPEPLVLQSFPSDEGWPFAKYLGACGRMV
AVNYVGEELWSYFNAPWEKRVDLAWQLMEIAEQLTNNDFEFALYLLDVSFDNFAVGPRDG
KVIIVDAENVLVADKRLIRQNKPENWDVWYESKFDDCDKEACLSFSKEILCARATVDHNY
YAVCQNLLSRHATWRGTSGGLLHDPPSEIAKDGRLEALLDECANPKKRYGRFQAAKELRE
YLAQLSNNVR
>SsalaDIA1R
MPLKRCDQRAVGSVRLTVCVLSWLLCVCWAPSVVEPAGTPPAPQQKALILQRAFLGLDKC
NACVGTSICKKLLKDQIRFDWWMSPDTTLPLAEKQSFPGNLTDDSLSWRPVVLSFLSSPC

```
LHSSSDRSICRSVGRQGPCSIEAVLRVTPRFQSLNQSHLLLPHIVKGLAPPLLRCPSQRL
LDRIVRRYAEVVDVGSVQMKHFSERDKLRLLYTLAVNQQPLILQMFPGTEGWPFPRYQGS
CGRLMVWASSRPLWGLYGSSREFVQRVDVAYQLLHITQGLGHNSLGFLLYYTRLGEDMFG
LLDDQRVFITDASSIGVIDLEQGFPPDPPSQTGSDGDIFSCLGQGTSPCHRSPPCSSVRP
TQSLTLLCTALLPRLLLTERGAQTTRLPMEGEAEAGRLARDVPLLLGVCADPSQPDWRIM
AAVGSLMDLLKPMRPCNPHYTYRYPECRYNQDY
>MdomeDIA1R
MESVIHGPLASPCLGWLTLLQLVTLSCISSTESLTAPSSVPRVKSSYNFGRTFLGLDKCN
ACIGTSICKKFFKEEIRFDNWLASHLKLPPDYFSTYPANYSDDTKSWRPVEISRLISKYQ
HELSDRRICASASGSKTCSIERVLRKTERVKKWLKAKRLTPDLVQGLPSPMLRCPSQRLL
DRIVRRYAEVADAGSVFMDHFTDRDKLRLLYTLSINAHPIMLQIFPGAEGWPLPRYLGSC
GRFLVSTSTQPLEEFYSSPADQAADLAYQLLGILDSLRNNDLNYFFYFTRIDAGTFGIFD
NGHLFIRDASTVGVIDKQRGSLTLDRQEESKDIFSCLVLDCQSPFPSCSSVKEKQSWVMA
CQQLLPQLLKEKFPQPIQEQIDSALVLCGNSSLSDQEVIEAASRLKAILKPLRTCDPRYA
YRYPDCKYSDRY
>RnorvDIA1R
MELGRPGAAATAFRQRWPAWMLLWVSTLSCSFSLPASLPPSLVPRVRSSYTLGKTFLGLD
KCNACIGTSICKKFFKEEIRLDNSLVSHLGLPSQDLLSYAANYSDDSKTWRPVEISRLVS
KYQSEISDKRICASASGPKTCSIERILRKTGRFQKWLQAKRLTPDLVQGLPSPFLHCPSQ
RLLDRVVRRYAEVVDAGSIFMDHFTDRDKLRLLYTLAVNAHPIILQIFPGAEGWPMPKYL
GSCGRFLVSTSTRPLQEFYDAPPEQAADIAYQLLGVLESLRSNDLNYFFYFTHVDAGMFG
IFDNGHLFIRDASALGIIDKQEASQAADRTGENEDIFSCLVSGCQVQMSSCDTVPEKQNL
VLVCQQLLPQLLRGKFPSPVQQEIDSALSLCSKDASTNLEVFGATSLLKNILRSLRTCDP
RFAYRYPDCKYNDRF
>MmulaDIA1R
MEPQLGPEAAALRPGWLALLLWVSALSCSFSLPASSPSSLVSQVRTSYNFGRTFLGLDKC
NACIGTSICKKFFKEEIRSDNWLASHLGLPPDFLLSYPANYSDDSKIWRPVEIFRLVSNY
QNEISDRRICASASAPRTCSIERVLRKTERFQKWLQAKRLTPDLVQGLASPLLRCPSQRL
LDRVVRRYAEVADAGSIFMDHFTDRDKLRLLYTLAVNSHPILLQIFPGAEGWPLPQYLGS
CGRFLVSTSTRPLQEFYGAPPDQAADLAYQLLGVLESLRSNDLNYFFYFTHIDAGMFGVF
NNGHLFIRDASAVGVIDKQEGSQEATRAGENKDIFSCLVSGCQAQLPSCESVSEKQSLVL
VCQKLLPRLLQGRFPSPVQDDIDSALAQCGDSTRPDPEVLGAASWLKHILRPLRTCDSRF
AYRYPDCKYNDKF
>BtaurDIA1R
MEPRLGPKAAALHLGWPFLLLWVSGLSYSVSSPASPSPSPVSRVRTSYNLGKTFLGLDKC
NACIGTSICKKFFKEEIRFDNGLALHLGPPPDDLPSYSANYSDDFKTWRPVEISRLVSKQ
QNKISDGRICASAAAPKTCSIERVLRKTGRFQKWLQAKRLTPDLVRGLSSPLLRCPSQRL
LDRVVRRYAEVADAGSIFMDHFTDRDKLRLLYTLAVNTHPVLLQIFPGAEGWPLPQYLGS
CGRFLVSTSTSPLQEFYGAPPDQAADLAYQLLGVLESLRSNDLNYFFYFTHVDADMFGIF
NNGHLFIRDASALGVIDRQEGSQAASGAGDNKDIFSCLVSGCQTKLPSCDTIPEKQNLVL
VCSQVLPLLLQAKFPSPVQEEIDAELTRCADGTRPDPEVLGAASRLKDILRPLRTCDPRF
AYRYPDCKYDDKF
>GgallDIA1R
MGRWICCLCSRVADWLMLLLVLARSSNPSAAATASPSAPHVRPSYSFGRTFLGLDKCNAC
IGTSICKKFFKEEIRFDTWLSSHLKLPPSYLLSYLGNYTDDA
QSWRMVDITRLTTKYQHDRADQHICTSLLKTKTCSLERALRRTHRFQKWLRAKRLTPDLV
QGLSSPMLRCPSQRLLDRIVRRYAEVPDAGSIYMDHLTDQDKLRLLYTLSVNSHPILLQI
FPDVEGWPFPRYLGSCGRLVVSASTRPLRDFFRAAPEVAADLALQLLAVLHSMGTNDLNY
FFYFTRVDVGTFGVFSNGHLFIRDASTLGIIDKEEGSQPIDGQQEYKDIFSCLTVDCQSA
FVSCNSIREKQSLVMVCQELLPKLLRGKFLPPVQEKIDSFLQHCAEGLADDQDVNEAMAK
LAQLLKPLRSCDSRFAYRYPDCKYSDKY
>DreriDIA1R
MAGIWSGVWILCFILVFGTADPSPAPQDKSHDFRKIFLGLDKCNACIGTSICKKFFKDEI
RFERRLTAQSNLSSADVRSYEGNYTDSTAGWRPVVVSRLMSPHLHQLSDNSICTSAGKGK
SCSIEGVLRATSRFQTWVHSNLLLPSMVKGLVTPMLRCPSQRLLDRIVRRYFEVTDVGSV
QMKHFNEKDKLRLLYTLAVNQQPLILQMFPGTEGWPFLRYHGSCGRLMVWAASRALRTLF
SSPLERRADLAYQLLHITQSLSANSLRFRLFYTRIPEDMFGILEDNKVFIVDTSTIGIID
LQEGHPPDKDLLPEELDVFSCLSGSCVRPPPCETVREAQSFILLCKYILNNLLTSNDKQS
GLPRAAVDELLVCADPSQLDQTIIKSVQSLKNILKTLRPCSPQYAYRYPECLYSDKF
>MmuscDIA1R
MESQWRGAAATAFHQHWLARLLLWVSTLSCSFSLPASLPPSLVPRVRSSYTMGKTFLGLD
KCNACIGTSICKKFFKEEIRFDNSLASHLGLPPQDLHSYAANYSDDSKTWRPVEISQLVS
RYQIEISDRRICASVSAPKTCSIERILQKTGRFQKWLQAKRLTPDLVQGLPSPFLRCPSQ
RLLDRVVRRYAEVVDAGSIFMDHFTAGDKLRLLYTLAVNAHPIILQIFPGAEGWPMPRYL
GSCGRFLVSTSTRPLQEFYDASPEQAADLAYQLLRVLESLRSNDLNYFFYFTHVDAGMFG
IFDNGHLFIRDASALGIIDKQEGSQAAARTGENEDIFSCLVSDCQIQLSSCDTVPEKQSL
VLVCQQLLPQLLQGKFPSPVQKEIDSALSLCSKDNSTDLEVLGATSWLKDILRSLRTCDP
RFAYRYPDCKYNDRF
>HsapiDIA1R
MEPQLGPEAAALRPGWLALLLWVSALSCSFSLPASSLSSLVPQVRTSYNFGRTFLGLDKC
NACIGTSICKKFFKEEIRSDNWLASHLGLPPDSLLSYPANYSDDSKIWRPVEIFRLVSKY
QNEISDRRICASASAPKTCSIERVLRKTERFQKWLQAKRLTPDLVQGLASPLLRCPSQRL
LDRVVRRYAEVADAGSIFMDHFTDRDKLRLLYTLAVNSHPILLQIFPGAEGWPLPKYLGS
CGRFLVSTSTRPLQEFYDAPPDQAADLAYQLLGVLESLRSNDLNYFFYFTHIDAGMFGVF
NNGHLFIRDASAVGVIDKQEGSQEANRAGENKDIFSCLVSGCQAQLPSCESISEKQSLVL
VCQKLLPRLLQGRFPSPVQDDIDSILVQCGDSIRPDPEVLGAASQLKDILRPLRTCDSRF
AYRYPDCKYNDKF
```

>EcabaDIA1R
MEPWLGPEAAALRPGWPALLLWVSALRCSVSSPASPSPSLVPRVRTSYNFGRTFLGLDKC
NACIGTSICKKFFKEEIRFDNRLASHLGLPSGYLPSYSANYSDDSKTWRPVEVSRLVSKQ
QNEISDRRICAFAAAPKTCSIERVLRKTRRFQKWLQAKRLTPDLVRGLPSPLLRCPSQRL
LDRVVRRYAEVPDAGSIFMDHFTDRDKLRLLYTLAVNAHPILLQIFPGAEGWPLPRYLGS
CGRFLISTSTSPLQEFYSAPPDQAADLAYQLLGVLESLRSNDLNYFFYFTHVDAGMFGIF
NNGHLFIRDASTLGVIDRQEGSQAAARAGENKDIFSCLLSDCQAELPSCDTVPEKQSLVL
VCRQVLPRLLQGKFPSPVQEEIDATLTWCGEDTRPDAEVLGAASRLKDILRPLRTCDPRF
AYRYPDCKYNDKF
>DordiDIA1R
MDPQLGPEAAALHPGWQVLLLLVVSVLSCFSSPASPPPSLVSRVRTSYNLGRTFLGLDKC
NACIGTSICKKFFKEEIRFDNSLASDLELASDYLPSYSANYSDDSKTWRPVEIFRLVSKE
QNEISDRRICAFASAPKTCSIERILRKTGRFQKWLQAKRLTPDLVQGLPSPFLRCPSQRL
LDRVVRRYAEVADAGSIFMDHFTDRDKLRLLYTLAVNAHPIFLQIFPGAEGWPLPKYLGS
CGRFFVSTSTRPLQEFYGAPPDEAADLAYQLLGVLESLRSNDLNYFFYFTHVDAAMFGIF
SNGHLFIRDASALGVIDKQEGSQAAPKAGENQDIFSCLVSGCQAQLLPSCDNIPDKQSVV
LVCQQLLPQLLQGKFPSPVQVEIDSALAQCGQGIRPDSEVFGAASRLKDILRPLRTCDPR
FAYRYPDCKYNDRF
>OanatDIA1R
MGLRLGCRLFSAALSWMTLLQFLTRGHGPVADASPAPSLVPRVKPSYSFGRTFLGLDKCN
ACIGTSICKKFFKEEIRFDNWPASHLKPPPDHFPGYSANYTDDSKSWRLVEISRLIGKDQ
NELSDQKICTSVSLVKTCSIERVLRKTERFQKWLKARRLTPALVQGLPSLLLRCPTQRLL
DRIVRRYAEVPDAGSVYMDHFTDRDKLRLLYTLSVNAHPVLLQVFPGAEGWPFPKYLGSC
GRLLVSTSTTPLLELSSGPPDRAADLGHQLLGVIEFLRHNDWNYFFYFTRIHAGTFGVFD
NGHLFIRDASSLGVIDRQEGKRPARSSPEPRDIFSCLAADCQSDLPSCNTVQEHQSLVLV
CRELLPKLLEGKFPKPVQEEIDALLDRCGDSSLDGQTVIGGARRLMDILKSLRTCDARFA
YRYPDCKYSEKY
>SpurpDIA1L
MGPRIRKKILILGSSILFTIFTVQLTGLNSLRDDDNNALPSTNGKFRTFVPSKGDRKRNL
LHAGKQGKEELGFDSKDTRRTLQDIRTADEIKGHPRIQAGDETALTQDGVQGMKNATVVM
TTRLQFENLFPSLLEEERCPLCYGTTNCDQIYAGNISFHVGSVNLSEPHAIRGTWGDRRI
VGKRLVSREVFERLEKIICNQSQVDPKRSCEVNAAATNSWMSKSSALNRVHKLHQEVYES
QLIAISATTCASPDFFEELKQLYRVSRWGTGIASTEMAILATTMALNPEPALLKFFRNIP
SLRPYFTEYLGECGRVILTEPSGKPLSSYLKASWKDRVDISLKILQMIEDFHDSSDKWLV
LLLDFGYENFVMTSEGQLKVVNLGGMVIVDKDQTSTTPDMNPHLNNRTELCNEDCLNTFV
KQLQTEPDTHCREVPRHVELMYMMACHSLLSDLMTTKYERFFQPLDTPRKHHPGGMLHDA
PYEVDSVLSELLYECVFEGQPGRRMHSVRVLKRLLTIIQRGFSYKDAADRTSL
>BflorDIA1Lb
MASARRWCRRLHYSGWTRVLFLLTLMIAVGFIARNYWQVEDDLEARKRISNGGDKKYAQS
IQELLAQGIDEKRMREALRQKAEEVHRIVKERGEALAKEAQAKAKAKANVVRINIHKPEL
NPENQHSWKAIEKMAAHDEIEDHKKQPEQYMTFKRLMDAKKCPACYGESLCEQAEVGLIT
MDVADKTLEHKGVYFGHFRNTEVVAKRLVGKDGWTRFDEFICQNASLPKDCDVSHMISDT
VLVTDNVLQVSFLQDAWRIAHTRRSIAMEACMTDRLIELIKTAYDENVNGKLSKTERAYM
ITALLLNPEAALLKHFTSRAEEYWPFPKYLGACGRVILVESGGKLLGSAIESPWKERANI
ALQLLEMIDKFRNGDPKWVVIFVDFSFNNFAVNNYGRLTLIDFDDVMLIDREEFVGENKT
EPCDLKCFKTFISQIEAMNSYDSCSAMPQYSQMMYALACVRLLSHLPEHLSEPNPMDPKP
DKHRPDSERPRLLWGPPEQEAKVLEELLRGCVEENVAGGRLEAVGELKTFLRRNAKGEN
>BflorDIA1La
MPILVESEAGYGGRHGRAMVARRMVRKCCLPLLVLVSATLAVLYIYLVQVQGSPWVSLGQ
SQAGNKQKKDEYIVWRGEEQKVEVQQKQEEKKHPAEEPKEKPRQDKSAVTERKPKGVPRS
LLRNAPCPACLGDNLCEEFEDGMIDLGSEVTSWKVKAYTGTWDKVEVMVTQCASEERLER
FEEFVCRNLSETATSCDPGKVLLQEDLKERLQPAHLKKSLREVFHPNTTSSLQATTCMSK
PFLKLLQKTFDDNGNKRLNRLEQAIPRKKLPIPAFLGACGNLVATETAGKPLSMYLEVKG
PWQVRANLSLQLLQMLDDFQNKDPDWLLMFVEVNIENFSVSSDGRLILTDLGNMTIINKH
DLDKNSTKKRSSVCNEACFKRFTRNLSHRPETSCRQAGRYSQLMYARACQRILGCWQTER
SGEGRVVLSSSKDASCAYGRGLLFGPPREAKQELEDLLTECVEETKAGGRITALKQIRVL
LAA
>BflorDIA1Lc
MVIADGDNDIDISWAEPEKCPACFGDKCELLRRGHFVKVDSESD
RYMKKGIVSTGRIGGVKVIAKSMNEAGAWQRYERFICRSSSRPHVCNASSFILETMLV
TDVALKVPWLREAWKICHLEKSALSLCVSDRFLEDVRQLYVEDGGTDMTKEGDTGRAF
LSTSLLLNEEAVLLRYFTTKSTTPWPFPKFYGACGRVIVVEHAGRTLDTFMESSWEVR
ADIALQLLQLVDALREKDPDWVLFFLDVSFQNFAVDSRGWVRLIDLDDVMVIDRRTVV
NQEQTEMCNEQCYTDFQKKLYSDEYHCDDIFKYAPMMYASICARLLSNLQKHPERRKW
GEIREYQEGQSIELDEPPVKGFLHNPPDEIREALEGALTQCVHETLPRGRLGAVLRLQ
EILGTT
