# Supplementary figures and images for: Characterization of the Deleted in Autism 1 Protein Family: Implications for Studying Cognitive Disorders
Source: PLoS One. 2011 Jan 19;6(1):e14547. doi: 10.1371/journal.pone.0014547 (PMC3023760; doi:10.1371/journal.pone.0014547)

Fig. S11

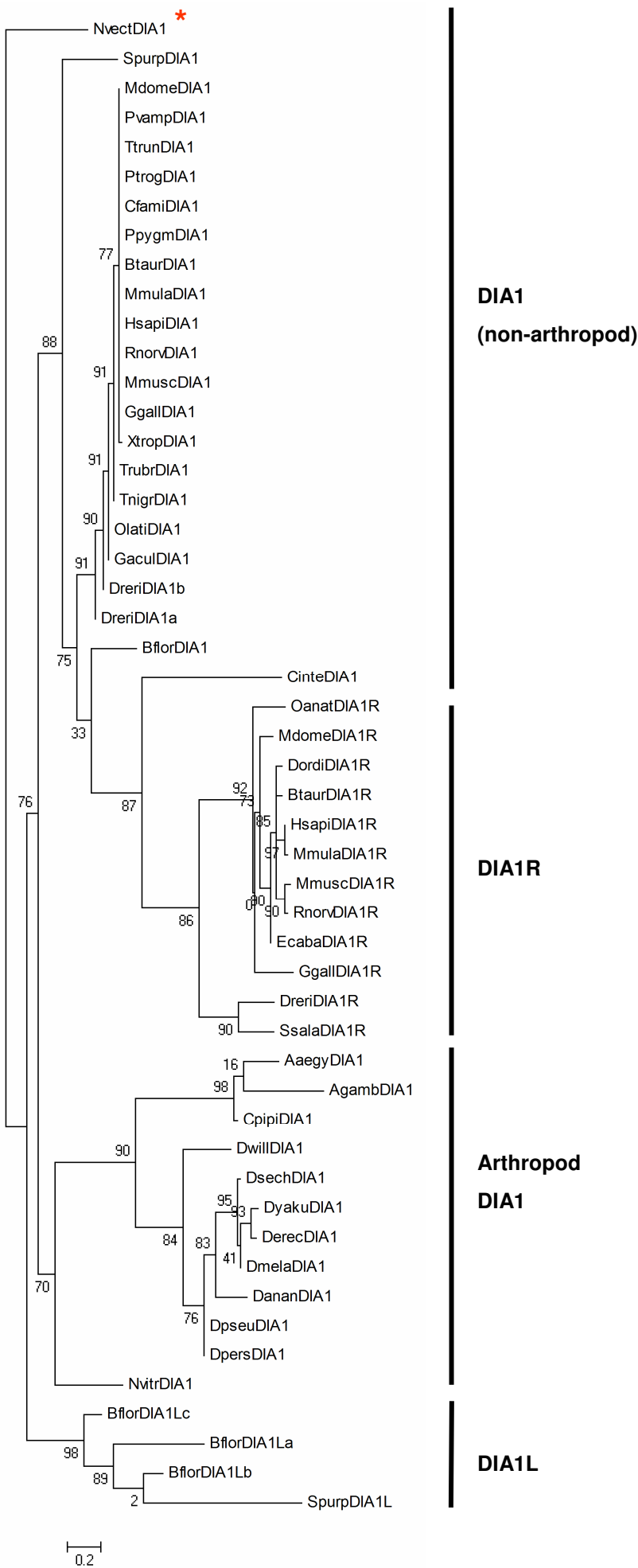

Supplement: Figure S11 — Maximum-likelihood tree of DIA1-family proteins. Proteins encoded by each full-length DIA1-family gene were aligned using CLUSTALW [47] and subjected to maximum-likelihood analysis [151] using PhyML phylogeny software [135]. Approximate likelihood-ratio test for branch-support statistics [156] was carried out, and percentage values are shown next to branches. Branch lengths are proportional to the number of amino acid substitutions per site (see scale bar). G-blocks were used to eliminate poorly aligned positions and divergent regions, since they may not be homologous or may have been saturated by multiple substitutions [148]. The tree was rooted on the cnidarian N. vectensis DIA1 sequence (NvectDIA1), as highlighted with an asterisk. Organism abbreviations use the first letter of the genus name, followed by the first four letters of the species. Full species names and accession numbers can be found in Tables S1, S4 and S7. (0.12 MB PDF) [file pone.0014547.s021.pdf]

Fig. S12

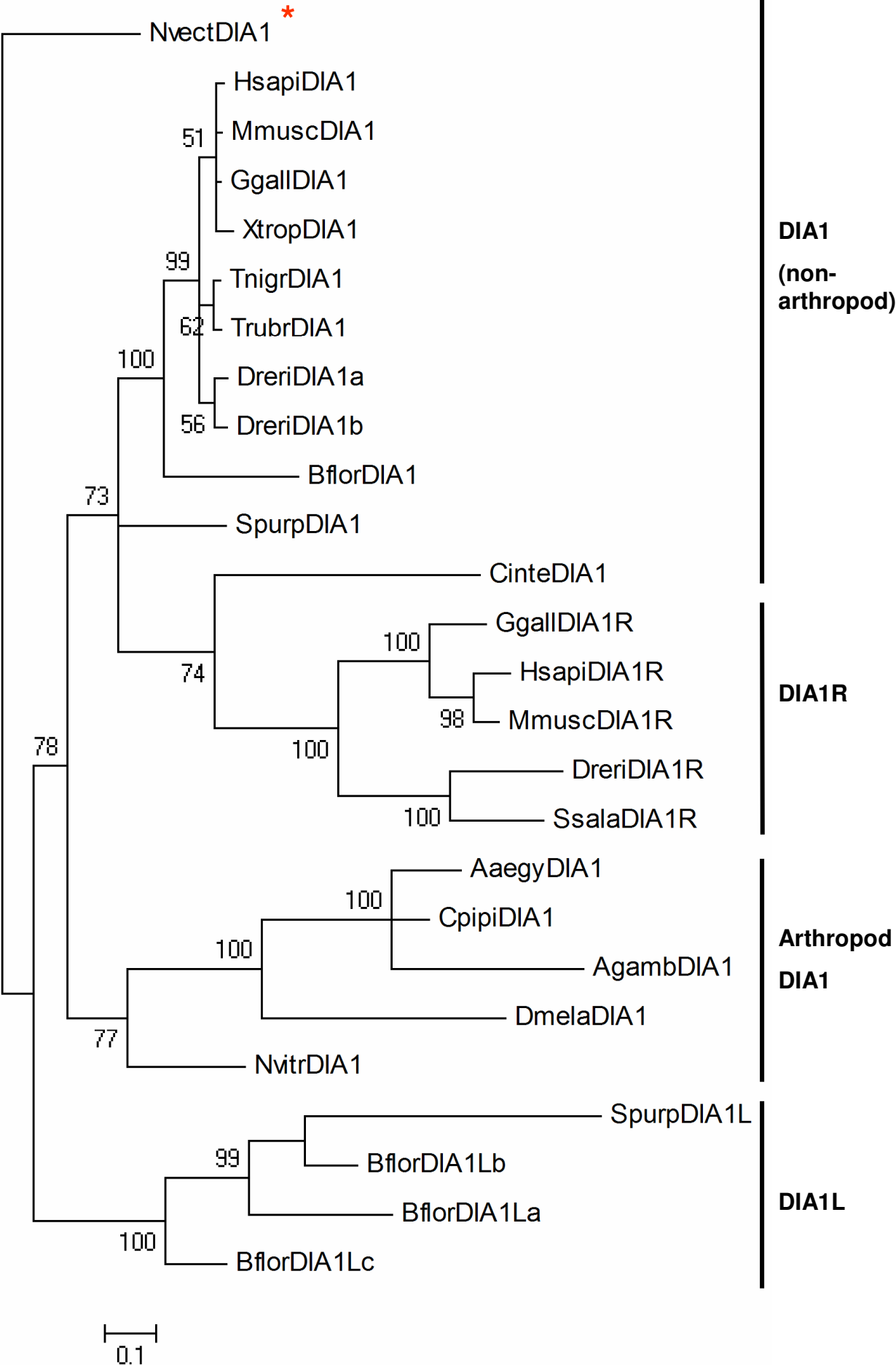

Supplement: Figure S12 — Phylogeny of the DIA1-family reconstructed using a Bayesian phylogenetic approach. A subset of DIA1-family gene products were aligned using CLUSTALW [47] and subjected to Bayesian inference of phylogeny using the MrBayes programme [153]. The number above each branch refers to the Bayesian posterior probability of the node, given as a percentage (e.g., 77 represents a posterior probability of 0.77). Branch lengths are proportional to the number of amino acid substitutions per site (see scale bar). Gblocks were used to curate the alignment [148]. The tree was rooted on the cnidarian N. vectensis DIA1 sequence (NvectDIA1), as highlighted with an asterisk. Organism abbreviations use the first letter of the genus name, followed by the first four letters of the species. Full species names and accession numbers can be found in Tables S1, S4 and S7. (0.07 MB PDF) [file pone.0014547.s022.pdf]
